# Photolysis of 5-Azido-3-Phenylisoxazole at Cryogenic Temperature: Formation and Direct Detection of a Nitrosoalkene

**DOI:** 10.3390/molecules25030543

**Published:** 2020-01-27

**Authors:** Upasana Banerjee, William L. Karney, Bruce S. Ault, Anna D. Gudmundsdottir

**Affiliations:** 1 Department of Chemistry, University of Cincinnati, PO Box 210172, Cincinnati, OH 45221-0172, USA; upasanabanerjee05@gmail.com (U.B.); bruce.ault@uc.edu (B.S.A.); 2 Department of Chemistry, University of San Francisco, 2130 Fulton Street, San Francisco, CA 94117, USA; karney@usfca.edu

**Keywords:** photoreactivity, azidoisoxazole, cryogenic matrices, nitrosoalkene, nitrenes

## Abstract

To enhance the versatility of organic azides in organic synthesis, a better understanding of their photochemistry is required. Herein, the photoreactivity of azidoisoxazole **1** was characterized in cryogenic matrices with IR and UV-Vis absorption spectroscopy. The irradiation (λ = 254 nm) of azidoisoxazole **1** in an argon matrix at 13 K and in glassy 2-methyltetrahydrofuran (mTHF) at 77 K yielded nitrosoalkene **3**. Density functional theory (DFT) and complete active space self-consistent field (CASSCF) calculations were used to aid the characterization of nitrosoalkene **3** and to support the proposed mechanism for its formation. It is likely that nitrosoalkene **3** is formed from the singlet excited state of azidoisoxazole **1** via a concerted mechanism or from cleavage of an intermediate singlet nitrene that does not undergo efficient intersystem crossing to its triplet configuration.

## 1. Introduction

Organic azides are one of the most versatile functional groups in synthesis, as they can be used to form new C–N bonds, either through electrophilic or nucleophilic substitution [1]. Additionally, their dipolar character makes them useful for forming new C–N bonds through cycloaddition. For example, the Huisgen 1,3-dipolar cycloaddition of organic azides to alkynes is commonly used to form triazole compounds, which have utility in medicinal and pharmaceutical chemistry [2,3,4]. The pursuit of sustainable chemistry has redirected efforts toward the use of photochemistry for synthetic applications because sunlight, photocatalysts, or eco-friendly light-emitting diodes (LEDs) can be used to drive photoreactions [5]. However, unlike the ground state reactivity of organic azides, their photochemistry is complex and not well understood because the reactivity is affected by both their structure and the environment. Better insight into the photoreaction mechanisms of organic azides will undoubtedly aid in their use in synthetic applications [6].

**Scheme 1:**
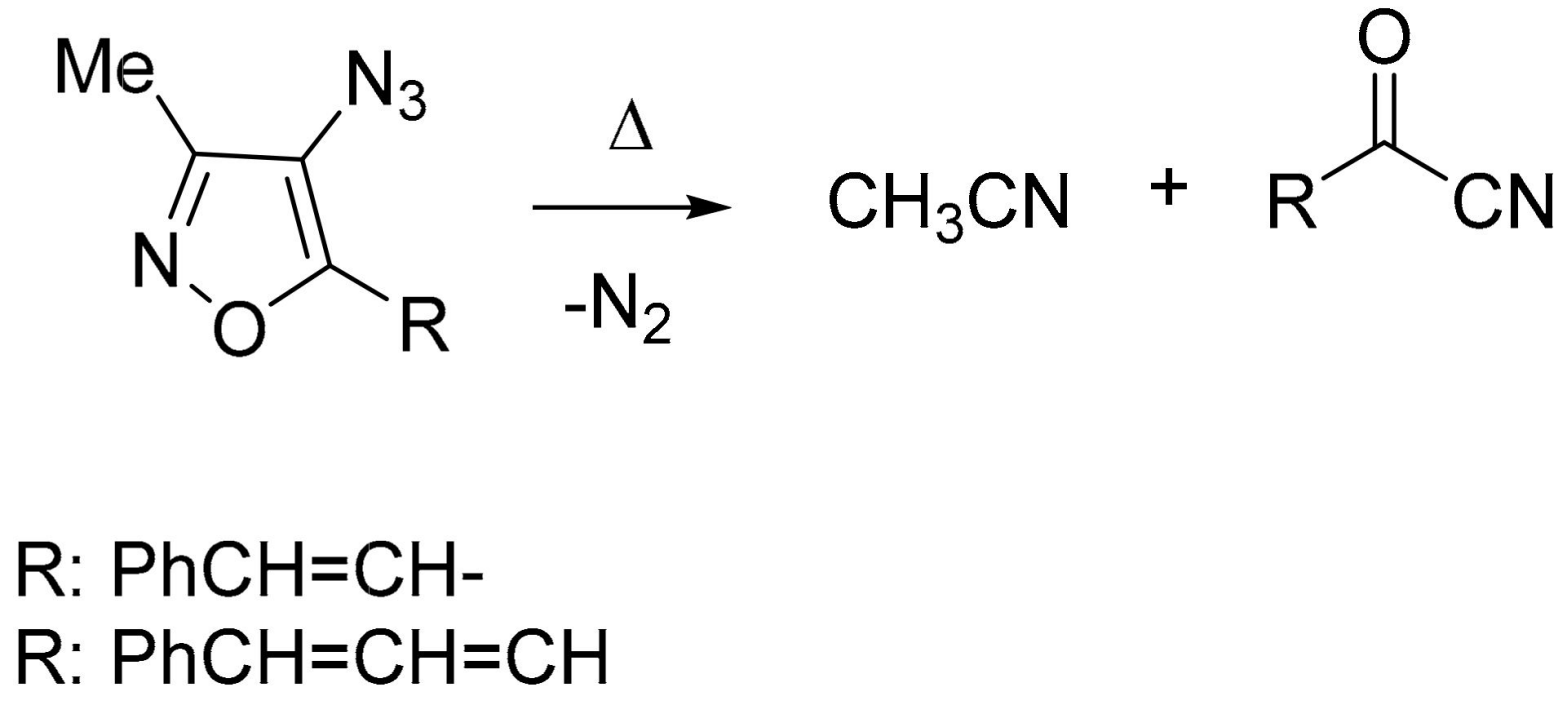
Thermal decomposition of 4-azidoisoxazole derivatives.

**Scheme 2:**
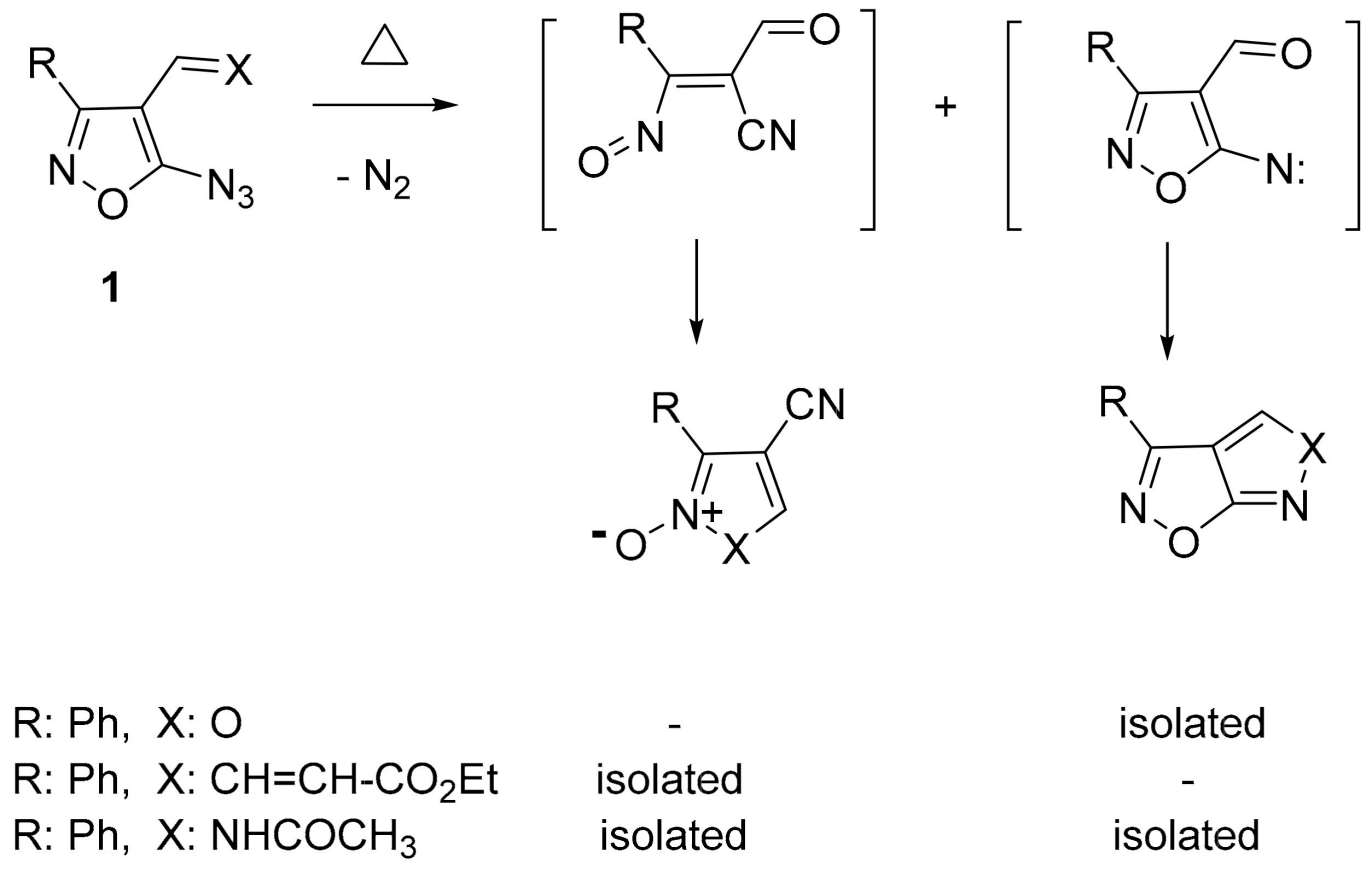
Thermal activation of 3-azidoisoxazole derivatives.

The photochemistry of vinyl azide derivatives has been studied and their reactivity has been exploited to synthesize various heterocyclic products [7,8]. However, for azidoisoxazole derivatives, another type of organic azide, the thermal decomposition behavior has been studied but the photochemistry has not been reported extensively. Kumar et al. and later L’Abbé and Godts studied the thermal decomposition of 4-azidoisoxazole derivatives and demonstrated that they undergo facile cleavage to form cyano products, as shown in Scheme 1 [9,10], presumably through a concerted reaction mechanism. In comparison, the thermal activation of 3-azidoisoxazole derivatives results in the formation of both isoxazoloisoxazole and hydroxypyrrole products (Scheme 2) [11,12]. L’Abbé and co-workers proposed that these products are formed by different reaction mechanisms, with the hydroxypyrroles formed through nitrosoalkene intermediates and the isoxazoloisoxazoles obtained from nitrene intermediates [12].

**Scheme 3:**
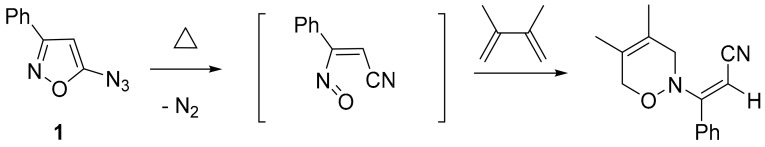
Trapping of the thermolysis product from azidoisoxazole **1**.

The thermal decomposition of a 5-azidoisoxazole in the presence of 2,3-dimethyl buta-1,3-diene yields the corresponding oxazine derivative (Scheme 3) [12]. Therefore, it was concluded that the thermal activation of 5-azidoisoxazole yields the corresponding nitrosoalkene, which can be trapped with dienes.

In this paper, we report the photoreactivity of azidoisoxazole **1** in cryogenic argon and 2-methyltetrahydrofuran (mTHF) matrices. We used IR and UV-Vis absorption spectroscopy to verify that azidoisoxazole **1** forms nitrosoalkene **3**. The proposed mechanism for the formation of nitrosoalkene **3** was supported by density functional theory (DFT) and complete active space self-consistent field (CASSCF) calculations.

## 2. Results

### 2.1. Matrix IR Spectroscopy

**Figure 1:**
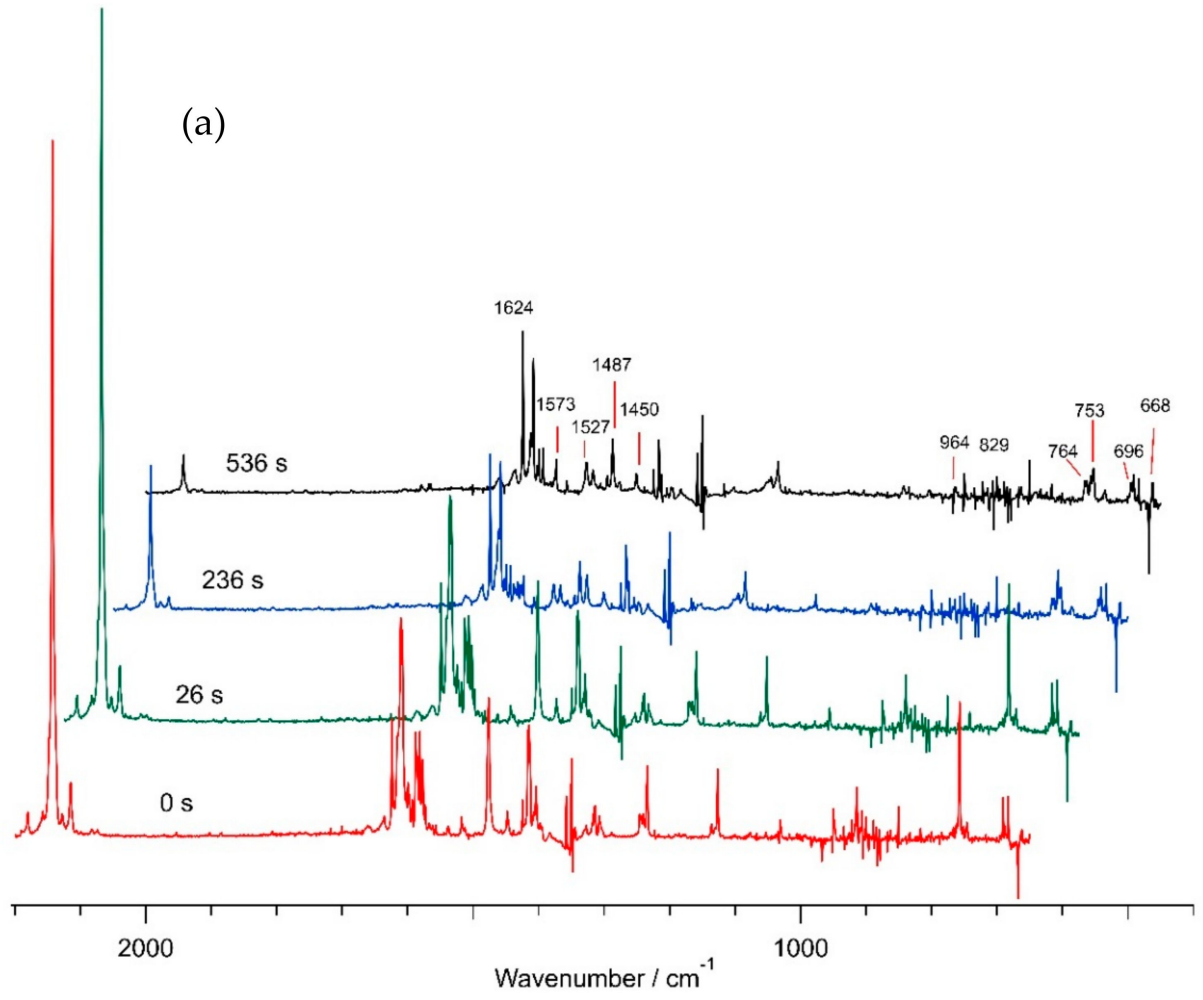

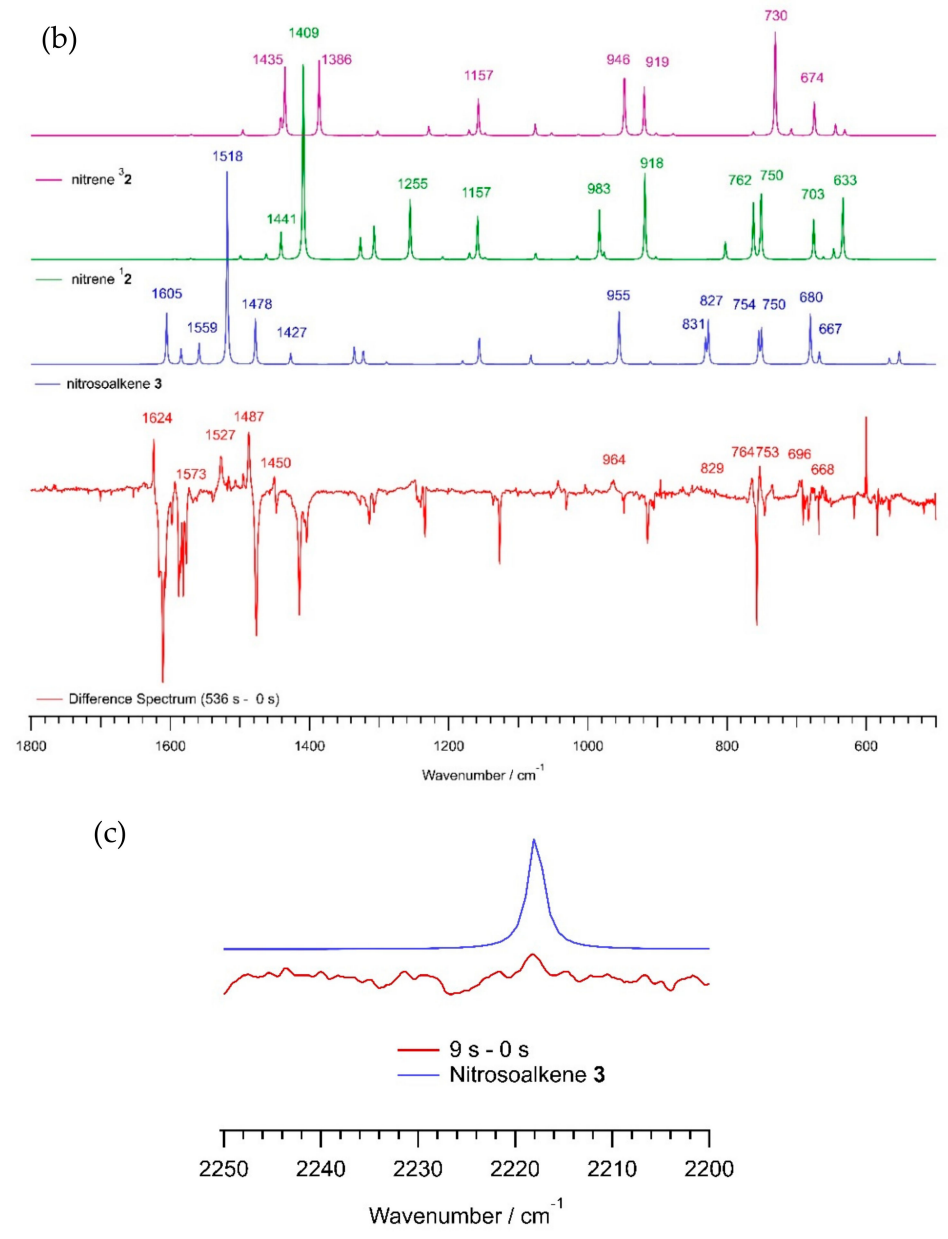
(**a**) Experimental IR spectra as a function of irradiation time (λ = 254 nm) of azidoisoxazole **1** in an argon matrix; (**b**) IR spectra of nitrenes ^3^**2** and ^1^**2** and nitrosoalkene **3** calculated using B3LYP/6-31+G(d) [14] theory and matrix IR difference spectrum (536–0 s); (**c**) Comparison of matrix IR difference spectrum (536–0 s) with calculated scaled IR spectrum of nitrosoalkene **3**.

To determine the photoreactivity of azidoisoxazole **1** at cryogenic temperatures, it was deposited into an argon matrix at 13 K and irradiated with a 254 nm UV pen. The reactivity was monitored by IR spectroscopy as a function of irradiation time. After 536 s irradiation, the bands due to azidoisoxazole **1** at 2144, 2115, 1611, 1608, 1598, 1581, 1577, 1448, 1415, 1404, 1314, 1240, 1127, 1031, 948, 915, 757, and 746 cm^−1^ were fully depleted. Concurrently, several new bands were observed at 2218, 1624, 1573, 1527, 1487, 1450, 964, 829, 764, 753, 696, and 668 cm^−1^ (Figure 1a). The intensity of the new bands increased with irradiation time, and they formed at same rate as the bands due to azidoisoxazole **1** were depleted. We assign these new IR bands to nitrosoalkene **3** based on comparison to its calculated IR spectrum. The calculated and scaled (0.9613 [13]) IR spectrum of nitrosoalkene **3** has the most significant bands at 2218 (C ≡ N stretch), 1605, 1559, 1518, 1478, 1427, 955, 827, 754, 750, 680, and 667 cm^−1^ (Figure 1b,c), which fits with the observed spectrum. It should be noted that the band at 829 cm^−1^ was considerably weaker than the other experimental bands, and thus, it is only tentatively assigned to nitrosoalkene **3**.

In contrast, the computed IR spectrum of singlet nitrene ^1^**2** does not fit the experimental IR spectra because the major calculated bands at 1462, 1441, 1409, 1157, 918, 762, 750, 703, and 633 cm^−1^ (Figure 1b) could not be reasonably matched with the observed major bands. Similarly, the calculated IR spectrum of triplet nitrene ^3^**2** does not fit well with the experimental spectra, as the major bands at 1495, 1442, 1435, 1386, 1170, 1157, 1147, 1075, 946, 919, 730, 707, 674, 670, and 630 cm^−1^ (Figure 1b) could not be easily match with the observed major bands. Thus, we conclude that the irradiation of azidoisoxazole **1** in argon matrices yields nitrosoalkene **3**.

### 2.2. Glassy Matrix UV-Vis Absorption Spectroscopy

**Figure 2:**
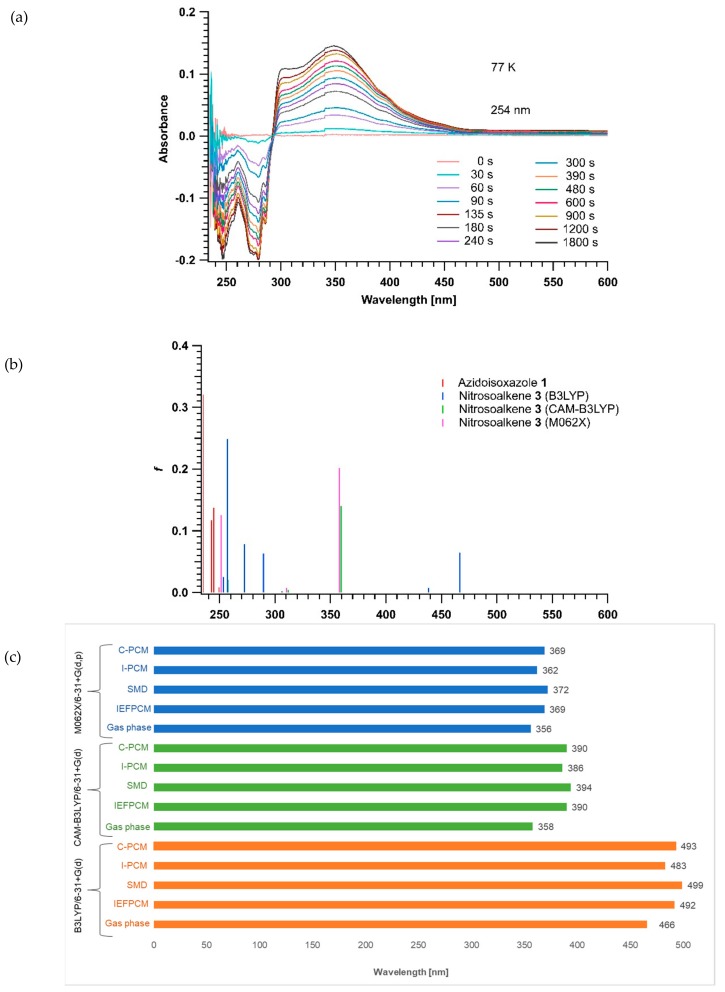
(**a**) Differential UV-Vis absorption spectra as a function of irradiation time (λ = 254 nm) of azidoisoxazole **1** in glassy 2-methyltetrahydrofuran (mTHF) at 77 K; (**b**) Calculated time-dependent density function theory (TD-DFT) absorption spectra of nitrosoalkene **3** using B3LYP/6-31+G(d), CAM-B3LYP/6-31+G(d), and M06-2X/6-31+G(d,p) using tetrahydrofuran (THF) as an implicit solvent with C-PCM and I-PCM solvation models; (**c**) Calculated TD-DFT absorption maxima of nitrosoalkene **3** above 300 nm using B3LYP/6-31+G(d), CAM-B3LYP/6-31+G(d), and M062X/6-31+G(d,p) in the gas phase and using THF as an implicit solvent with C-PCM, I-PCM, SMD, and IEFPCM solvation models.

To further characterize nitrosoalkene **3**, its UV-Vis absorption was investigated in a glassy mTHF matrix at 77 K. The absorption spectrum of azidoisoxazole **1** in the glassy mTHF matrix was recorded. Then, the matrix was irradiated with a 254 nm UV pen and the absorption difference spectra were obtained at periodic irradiation times (Figure 2a). Upon irradiation, negative absorption was observed below 300 nm due to the depletion of azidoisoxazole **1**, along with a new broad absorption band with λ_max_ at ~360 nm and a tail extending to 470 nm.

Time-dependent density function theory (TD-DFT) calculations of the absorption spectrum of nitrosoalkene **3** using the B3LYP/6-31+G(d) level of theory and basis set in the gas phase gave major electron transitions at 257 (*f* = 0.2486), 272 (*f* = 0.0781), 290 (*f* = 0.0632), 438 (*f* = 0.0077), and 466 (*f* = 0.0647) nm. The calculated bands above 300 nm overestimate the near-UV absorption band of nitrosoalkene **3** by ~100 nm. The major calculated band at 466 nm is due to a mixed electronic transition of the lone pair on the oxygen and the aromatic π-system into an antibonding π-orbital. In an attempt to obtain a better fit between this band and the observed absorption spectra, we preformed solvation calculations. TD-DFT calculations with tetrahydrofuran (THF) as an implicit solvent and employing several solvation models, such as Integral Equation Formalism version of the Polarizable Continuum Model (IEFPCM) [15], Universal Solvation Model based on Solute Electron Density (SMD) [16], Isodensity Polarizable Continuum Model (I-PCM) [17], and Conductor-like Polarizable Continuum Model (C-PCM) [18], located the major electric transition above 300 nm at 492 (*f* = 0.0818), 499 (*f* = 0.0834), 483 (*f* = 0.0666), and 493 (*f* = 0.0846) nm, respectively (Figure 2c). However, these calculations also overestimated the absorption of nitrosoalkene **3** (Figure 2a). Cramer and Truhlar have explained that dispersion forces are important in estimating short-range dielectric polarization effects in the solvation model of an electronic spectrum [19]. Furthermore, the B3LYP level of theory has significant deficiencies when estimating weak but important noncovalent interactions such as van der Waals forces [20,21].

To better model the absorption of nitrosoalkene **3**, we optimized its structure using the CAMB3LYP [22] level of theory, which has several long-range corrected functionals, with the 6-31+G(d) basis set. The TD-DFT calculations of nitrosoalkene **3**, in the gas phase and with THF as an implicit solvent, employing IEFPCM [15], SMD [16], I-PCM [17], and C-PCM [18] solvation models, resulted in the major electronic transition above 300 nm being located at 358 (*f* = 0.2017), 390 (*f* = 0.1463), 394 (*f* = 0.1484), 386 (*f* = 0.1197), and 390 (*f* = 0.1508) nm. Although most of these calculations also overestimated the most significant electronic transition for nitrosoalkene **3**, in comparison to the observed spectra, they yielded significantly better results than those obtained using the B3LYP level of theory (Figure 2b).

Finally, we opted to use the hybrid functional from Zhao and Truhlar [23] and the M062X level of theory with the 6-31+G(d,p) basis set to optimize nitrosoalkene **3** and calculate its absorption spectrum, as these hybrid functionals have shown promising results in predicting dispersion forces. The TD-DFT/M062X calculated spectrum of nitrosoalkene **3** in the gas phase and with THF as an implicit solvent with IEFPCM, SMD, I-PCM, and C-PCM solvation models exhibited major transitions above 300 nm at 356 (*f* = 0.2017), 369 (*f* = 0.1463), 372 (*f* = 0.1484), 362 (*f* = 0.1197), and 369 (*f* = 0.1508) nm, which match well with the observed spectrum of nitrosoalkene **3**. Thus, we conclude that irradiation of azidoisoxazole **1** in mTHF matrices results in the formation of nitrosoalkene **3**, as observed upon irradiation in argon matrices.

**Scheme 4:**
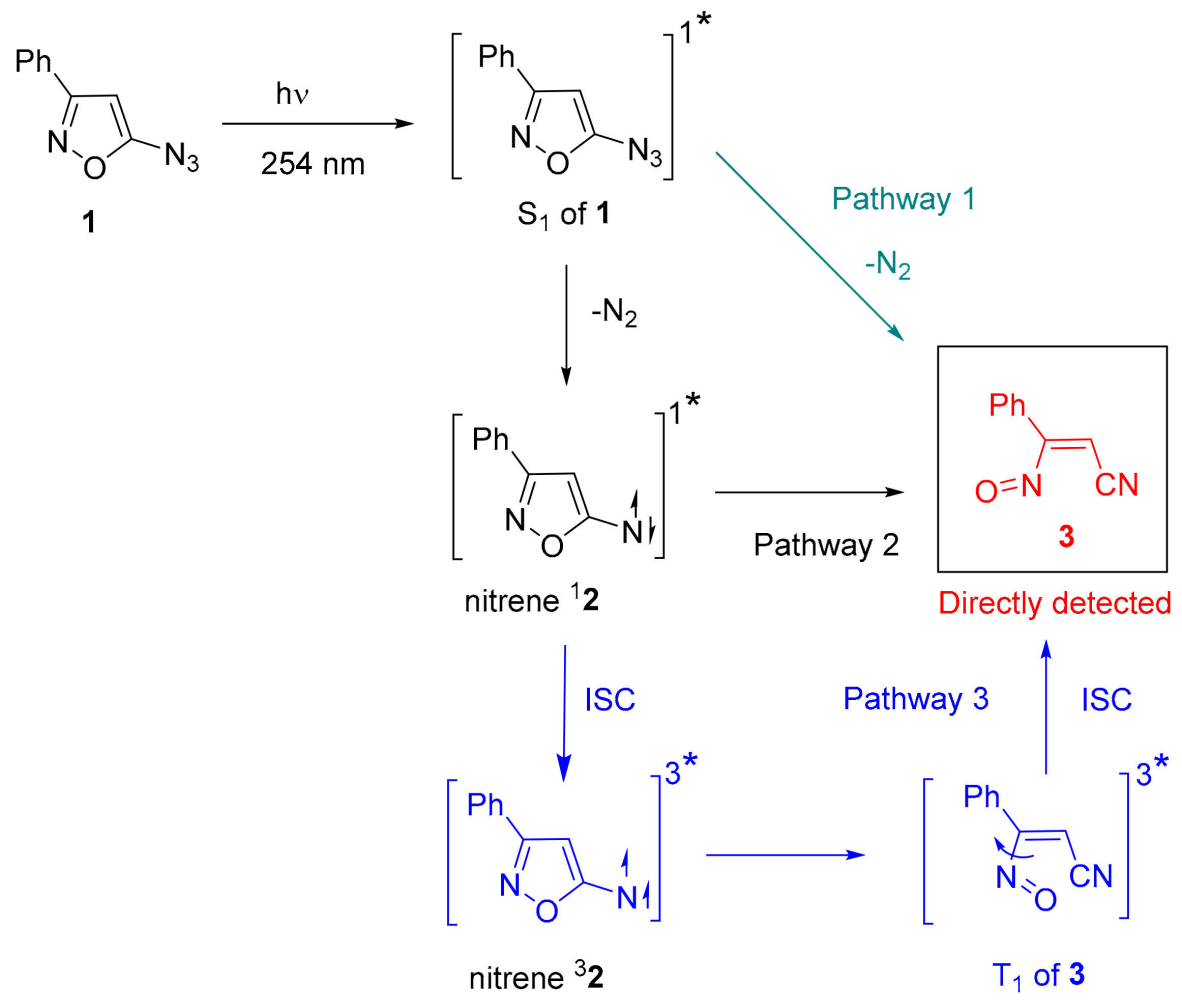
Photoreactivity of azidoisoxazole **1** in cryogenic matrices.

Based on the fact that photolysis of azidoisoxazole **1** in cryogenic matrices results in the formation of nitrosoalkene **3** and because we did not observe the formation of either singlet or triplet nitrene **2**, we theorize that azidoisoxazole **1** forms nitrosoalkene **3** in a concerted manner from the singlet excited state (S_1_) of azidoisoxazole **1** (pathway 1, Scheme 4). However, it is possible that the S_1_ of azidoisoxazole **1** forms singlet lnitrene ^1^**2**, which subsequently rearranges into nitrosoalkene **3** (pathway 2, Scheme 4). Furthermore, if nitrene ^1^**2** is formed, we cannot rule out that it intersystem crosses to its triplet configuration, nitrene ^3^**2**, which could then rearrange and intersystem cross to form nitrosoalkene **3** (pathway 3, Scheme 4).

### 2.3. Quantum Chemical Calculations

**Figure 3:**
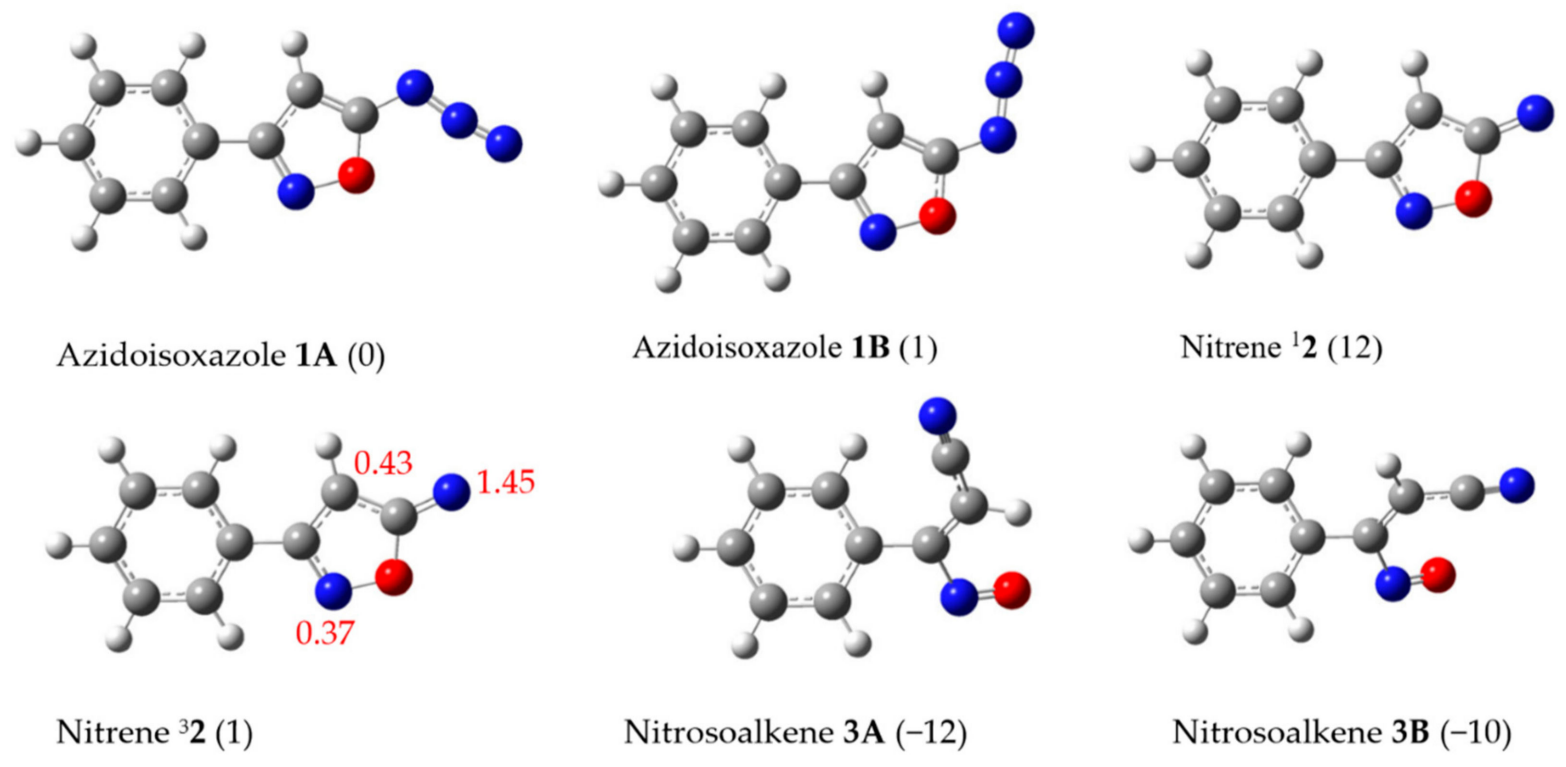
Optimized structures of azidoisoxazole **1**, nitrene ^1^**2**, nitrene ^3^**2**, and nitrosoalkene **3** using the B3LYP/6-31+G(d) level of theory. The numbers in parentheses are the energies in kcal/mol relative to the energy of conformer A of **1**. The calculated spin densities of nitrene ^3^**2** are displayed in red.

To determine the feasibility of the various pathways displayed in Scheme 4, we calculated the stationary points on the potential energy diagram using DFT calculations (Gaussian 16, B3LYP/6-31+G(d) [14,24,25]). We optimized the structure of azidoisoxazole **1** and obtained two minimal energy conformations, **A** and **B**, which differed mainly in the orientation of the azido chromophore (Figure 3). The calculated energies of azidoisoxazole **1A** and **1B** were within 1 kcal/mol of each other, with **A** being the more stable conformation. TD-DFT calculations were performed to locate the vertical excitation energies of the first singlet and triplet excited states (S_1_ and T_1_) of azidoisoxazole **1**. The S_1_ of **1** and the T_1_ of **1** were located 86 and 64 kcal/mol, respectively, above the ground state (S_0_). The open shell triplet and singlet configurations of nitrene **2** were optimized, with the singlet configuration being optimized using a broken symmetry calculation, as achieved using guess=mix as a keyword in Gaussian 16 (Figure 3). Triplet nitrene **2** was 11 kcal/mol more stable than the singlet configuration; however, it should be noted that due to spin contamination, DFT calculations do not accurately estimate the energy gap between singlet and triplet nitrenes. Furthermore, we optimized two minimal conformers of nitrosoalkene, **3A** and **3B**, with **3A** being 2 kcal/more stable than **3B** (Figure 3).

Due to the high spin contamination in the DFT calculations, the singlet and triplet configurations of nitrene **2** were also computed using the CASSCF and CASPT2 methods (Table 1). At the CASPT2(14,13)/cc-pVTZ//CASSCF(14,13)/cc-pVDZ level of theory, ^1^**2** is predicted to lie 13.8 kcal/mol above ^3^**2**.

**Figure 4:**
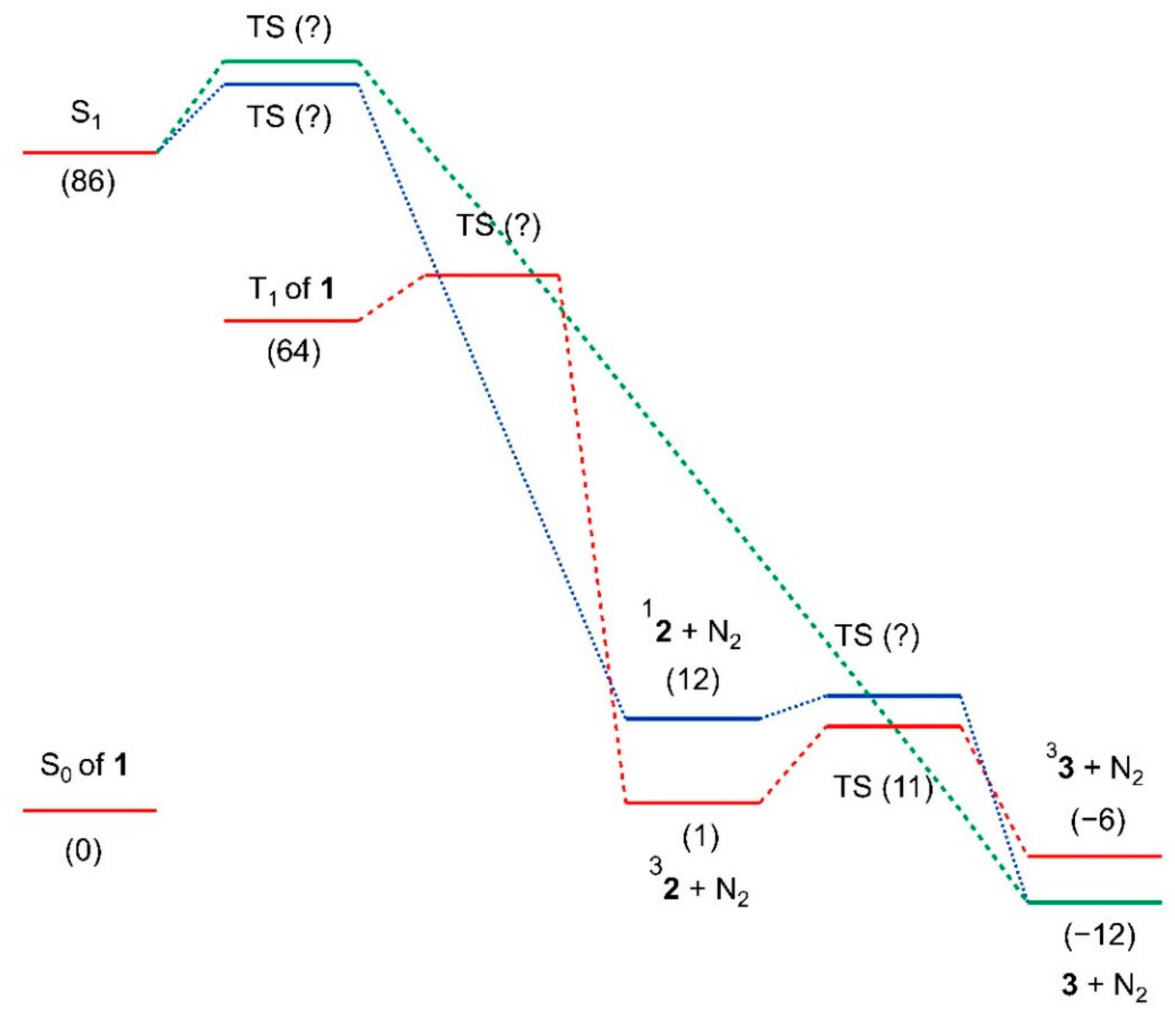
Calculated stationary points on the singlet and triplet energy surfaces of azidoisoxazole **1** using the B3LYP/6-31+G(d) level of theory. The numbers in parentheses are the energies in kcal/mol.

Finally, we calculated stationary points on the singlet and triplet excited state surfaces of azidoisoxazole **1** for the formation of nitrosoalkene **3**. However, due to spin contamination, we could not use DFT to optimize the transition state of S_1_ of **1** for the formation of nitrene ^1^**2** or that of nitrene ^1^**2** for the formation of nitrosoalkene **3**. In contrast, we calculated the transition state barrier for nitrene ^3^**2** to form nitrosoalkene ^3^**3**, which is located 10 kcal/mol above nitrene ^3^**2**. The calculated stationary points on the energy surface of azidoisoxazole **1** are displayed in Figure 4.

## 3. Discussion

Because the energy gap between the S_1_ and T_1_ of azidoisoxazole **1** was large (22 kcal/mol), intersystem crossing between these excited states was not expected to be efficient. Thus, it is reasonable that the photoreactivity of azidoisoxazole **1** occurred from its singlet excited state. The calculated energy gap between nitrenes ^1^**2** and ^3^**2** (13.8 kcal/mol) was slightly less than the singlet–triplet energy gap measured for phenylnitrene (14.9 kcal/mol) [26,27]. At cryogenic temperatures, irradiation of phenyl azide resulted in the formation of triplet phenylnitrene via intersystem crossing from singlet phenylnitrene. Thus, the reactivity of azidoisoxazole **1** at cryogenic temperatures was different from that of phenyl azide, as a stable triplet nitrene was not formed [28,29].

**Scheme 5:**
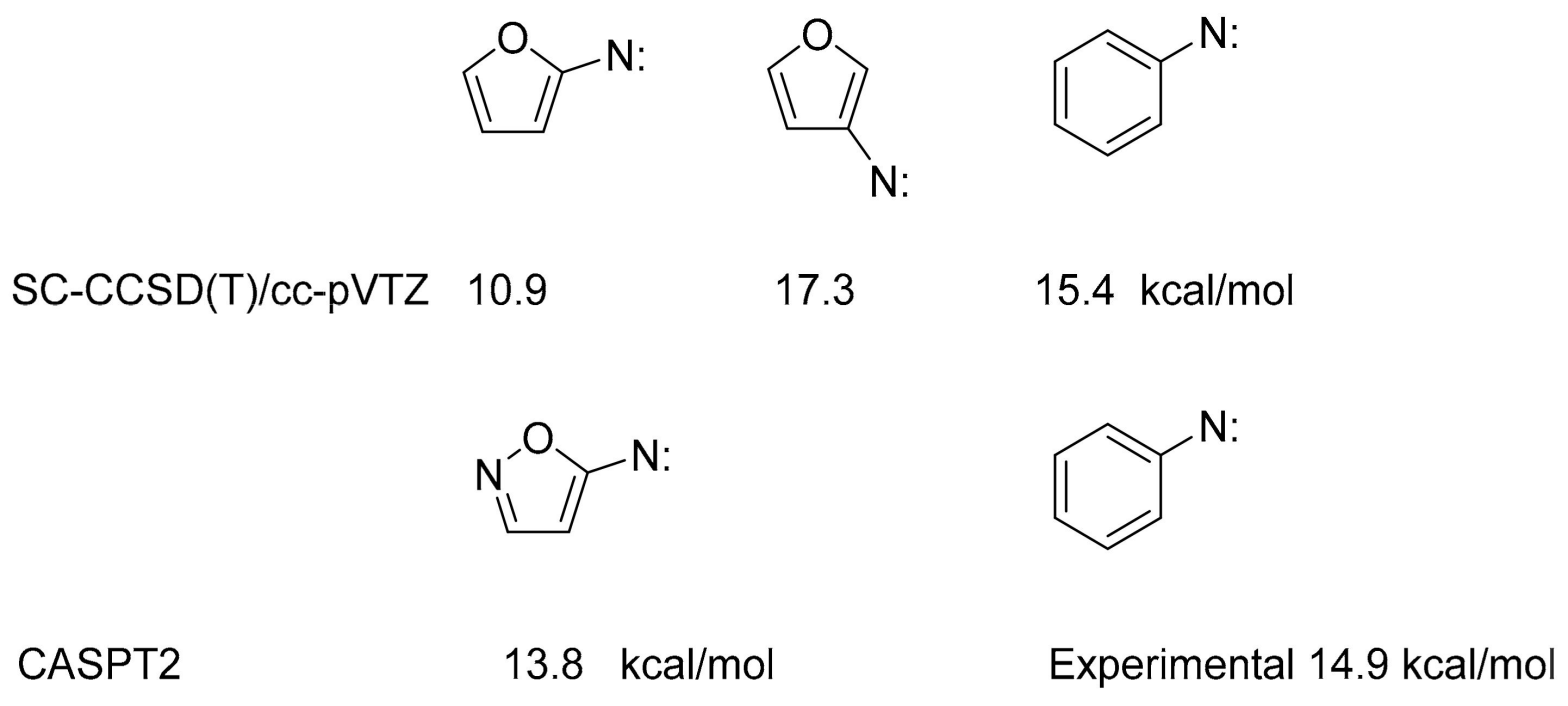
Singlet–triplet energy gaps for furanylnitrenes and phenylnitrene.

Based on computational studies of radical stabilization in furanylnitrenes using the spin-flip coupled-cluster method [30], Wenthold reported that the singlet–triplet energy gap of 2-furanylnitrene is only 10.9 kcal/mol (Scheme 5). He attributed the decreased singlet–triplet energy gap of 2-furanylnitrene to radical stabilization of its singlet state resulting from increased delocalization and decreased electron pair repulsion. In comparison, the calculated singlet–triplet energy gap of 3-furanylnitrene was larger (17.3 kcal/mol) due to a decrease in delocalization.

**Scheme 6:**
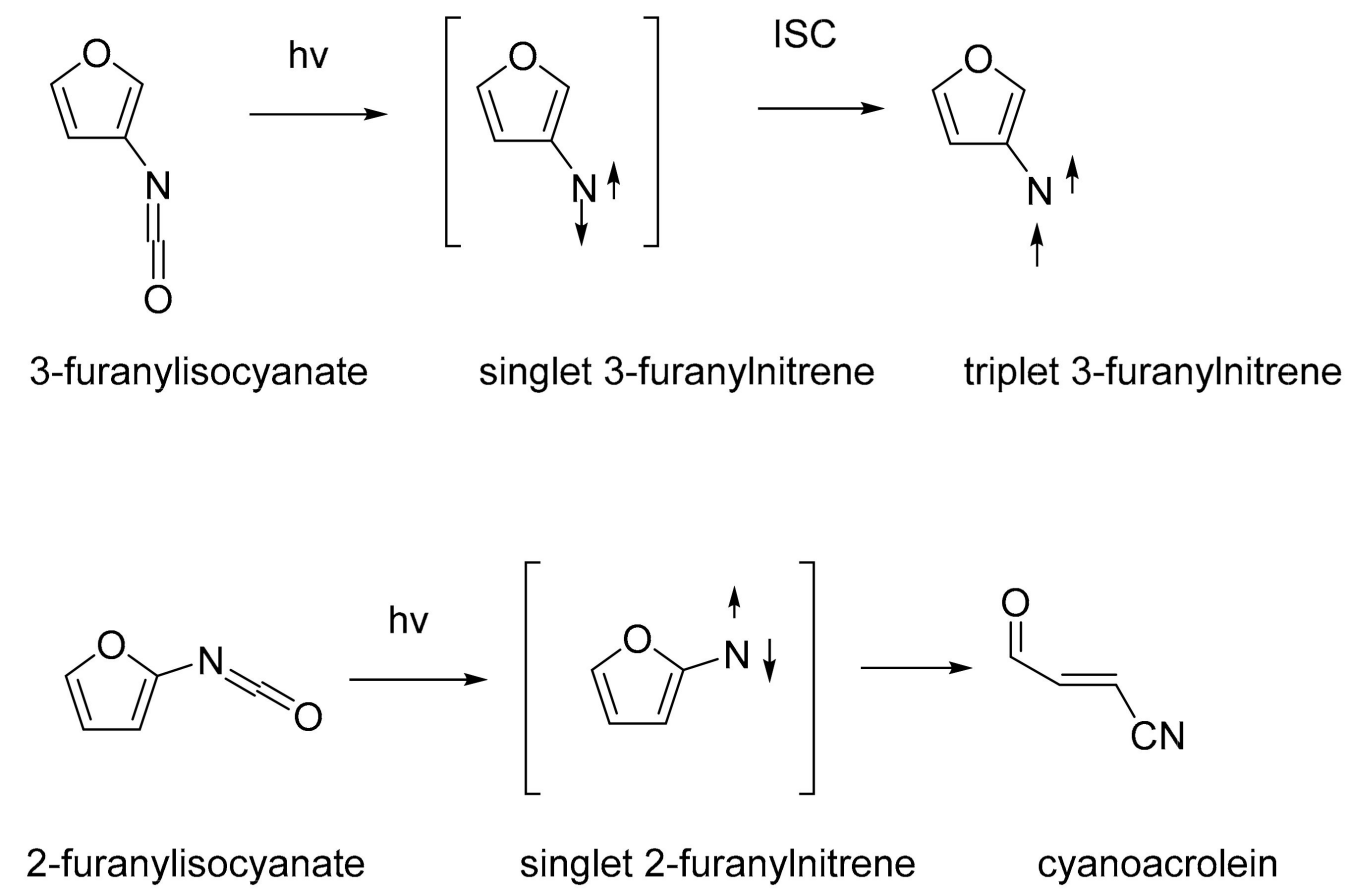
Photoreactivity of furanylisocyanates in cryogenic matrices.

Additionally, 3-furanylnitrene has been reported to be formed by direct irradiation of 3-furanylisocyanate in cryogenic matrices, presumably because intersystem crossing from the singlet to the triplet nitrene is efficient (Scheme 6) [31]. In contrast, photolysis of 2-furanylisocyanate gives cyanoacrolein rather than 2-furanylnitrene because 2-furanylisocyanate reacted in a concerted manner to form cyanoacrolein or through singlet 2-furanylnitrene, which underwent cleavage faster than it intersystem crossed to its triplet configuration. Thus, the photoreactivity of azidoisoxazole **1** resembled that observed for 2-furanylisocyanate.

**Scheme 7:**
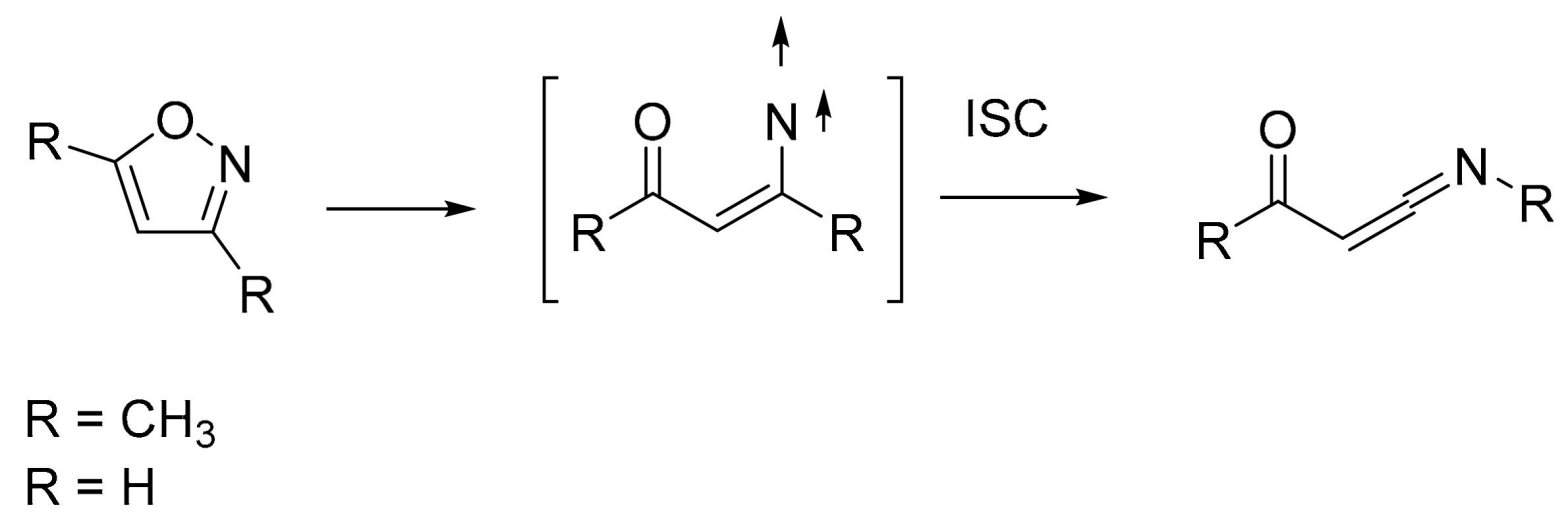
Photoreactivity of isoxazoles in cryogenic matrices.

**Scheme 8:**
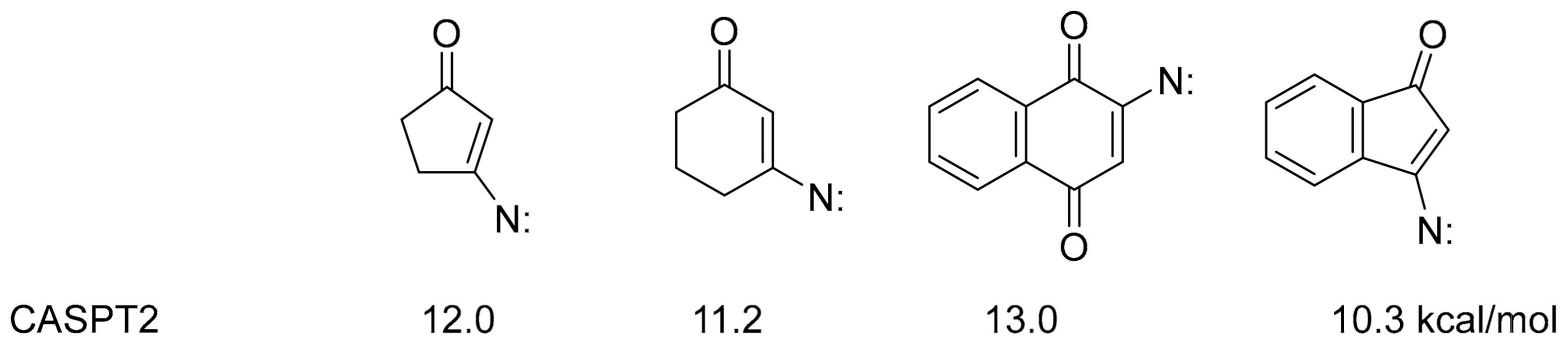
Calculated singlet–triplet energy gaps of cyclic vinylnitrenes.

Finally, the photoreactivity of azidoisoxazole **1** was compared with that of isoxazoles in cryogenic matrices. The irradiation of isoxazole derivatives at cryogenic temperatures resulted in the formation of the corresponding ketenimine. It has been theorized that the ketenimines are formed by intersystem crossing from triplet vinylnitrenes that are not stable (Scheme 7) [32,33,34]. Triplet vinylnitrenes can, however, be detected directly by laser flash photolysis of their isoxazole precursors in solution at ambient temperature [32,35]. Furthermore, they can be rendered stable at cryogenic temperatures by incorporating the vinyl bond into a cyclic structure (Scheme 8). The CASPT2 calculated singlet–triplet energy gaps of cyclic vinylnitrenes, reported to be between 10 and 13 kcal/mol, were similar to or slightly smaller than those calculated for nitrene **2** [36,37,38,39]. Thus, azidoisoxazole **1** reacts differently from isoxazole derivatives because it does not undergo cleavage of its C–O bond, as its azido moiety is more reactive.

Because we did not observe nitrene ^3^**2** in cryogenic matrices, not even at shorter irradiation times, it seems likely that the reactivity occurs on the singlet surface of azidoisoxazole **1** in a concerted manner or that nitrene ^1^**2** cleaves more efficiently to form nitrosoalkene **3** than it can intersystem cross to nitrene ^3^**2**.

## 4. Materials and Methods

### 4.1. Preparation of Azidoisoxazole 1

Azidoisoxazole **1** was synthesized by following the previously reported procedure [12]. Sodium hydride (0.041 g, 1.7 mmol) was placed a 25 mL round bottom flask, washed three times with hexane (2 mL) and once with dichloromethane (2 mL), and then vacuum dried for 1 h. Dry THF (3 mL) was added to the round bottom flask and the reaction was cooled to −30 °C using a xylene–dry ice bath. A solution of 5-amino-3-phenyl isoxazole (0.16 g, 0.99 mmol) and tosyl azide (1.5 mL, 7.5 mmol) in dry THF (3 mL) was added dropwise to the cooled suspension over 10 min under an argon atmosphere. The xylene bath temperature was raised to room temperature and the black reaction mixture was stirred overnight, diluted with water (25 mL), extracted with diethyl ether (25 mL), and dried under reduced pressure. Azidoisoxazole **1** was purified by silica gel column chromatography eluted with 5% ethyl acetate in hexane. Recrystallized azidoisoxazole **1** crystals (diethyl ether; 50 mg, 17% yield) were characterized using ^1^H-NMR and IR spectroscopy, and the obtained spectra corresponded well with the reported data [12]. ^1^H-NMR (400 MHz, CDCl_3_): 7.77–7.74 (m, 2H), 7.48–7.45 (m, 3H), 6.01 (s, 1H) ppm; IR (neat): 3129, 3059, 2121, 1598, 1570, 1469, 1368, 1168 cm^−1^.

### 4.2. Matrix IR Spectroscopy

Apparatus description: Matrix isolation of azidoisoxazole **1** in argon matrices was performed on conventional equipment [40]. This experiment was conducted in a completely stainless-steel home built vacuum system, with Nupro Teflon-seat valves (Swagelok Company, Colon, Ohio, USA). Pumping was provided by a Model 1402B Welch vacuum pump (Sargent Wlech Corporation, Buffalo Grove, Illinois, USA), and a Varían M-2 diffusion pump (Varian Corporation, Palo Alto, California, USA), with liquid nitrogen trap. Pressures near 10^−7^ mm at the gauge (cold cathode, Varían) were generally recorded at the start of an experiment. Cryogenics were supplied by a Model 21 closed cycle refrigerator (CTi, Inc., Waltham, Massachusetts, USA), operating down to 13 K. Argon was deposited on a CsI window. The sample was deposited by heating for 3.5 h at 313 K (40 °C), followed by 30 min at 315 K (42 °C) under vacuum. The vaporized sample was entrained in flowing argon (2 mmol/h), carried to the cryogenic surface and deposited.

The matrix was irradiated through a KBr window with a UV pen (λ = 254 nm). IR spectra at 1 cm^−1^ resolution were collected periodically as a function of irradiation time with a Perkin Elmer 100 Fourier transform infrared spectrometer (Perkin Elmer Corportation, Waltham, Massachusetts, USA). We used IgorPro8 software to plot and process the data.

### 4.3. Glassy Matrix UV-Vis Absorption Spectroscopy

Apparatus description: Glassy matrix UV-Vis spectroscopy was performed using the following instrumentation setup. The cryostat unit is a commercial Optistat DN2, a Variable Temperature Liquid Nitrogen Cryostat obtained from Oxford Instruments, Concord, Massachusetts, USA. (Temperature can be varied between 77 K and 500 K). **Mercury** iTC cryogenic environment controller was obtained from the same commercial source and it was used as a temperature controller. Vacuum chambers were pumped to high vacuum using KJLC RV series Rotary Vane pump to create thermal isolation. We used JASCO V-750 spectrophotometer (from JASCO Easton, Maryland, USA) for recording UV-Vis spectra. IgorPro8 software was used for plotting and processing the data. 

A stock solution of azidoisoxazole **1** (1.2 mg, 0.0001 mmol) in mTHF (0.5 mL) was prepared. Subsequently, 0.01 mL of this stock solution was added to mTHF (2 mL) and the resulting solution was placed in a quartz cuvette. After degassing the solution by bubbling with argon for 3 min, the cuvette was capped and sealed with Teflon tape. The UV-Vis absorption spectrum of **1** at ambient temperature showed a broad absorption band up to 300 nm (see Figure S1) and the maximum absorbance of the solution was below 0.6. This solution was cooled to 77 K using liquid nitrogen to form a glassy mTHF matrix. The absorption spectrum of **1** before irradiation (0 min) was recorded and then set as the baseline. Then, the glassy mTHF matrix was irradiated with a 254 nm UV pen and the absorption difference spectrum was collected periodically.

### 4.4. Quantum Chemical Calculations

Geometries of all species were optimized at the B3LYP, CAM-B3LYP, and M06-2X levels of theory with the 6-31+G(d) basis set, as implemented in the Gaussian 16 programs at the Ohio Supercomputer Center [14,23,24,41]. The absorption spectra were calculated using TD-DFT calculations [42,43] in the gas phase and with THF as an implicit solvent employing IEFPCM [15], SMD [16], I-PCM [17], and C-PCM [18] solvation models. The calculated IR spectra of the intermediates and products were obtained by frequency calculations using the parameters mentioned above, and the frequencies were scaled by a factor of 0.9613 [13]. The transition states were confirmed to have one imaginary vibrational frequency by analytical determination of the second derivative of the energy with respect to the internal coordinates. Intrinsic reaction coordinate (IRC) calculations were used to verify that the transition states were correlated with the products and the precursors [44,45].

CASSCF and CASPT2 calculations on nitrene **2** employed a 14-electron, 13-orbital active space consisting of all π/π* MOs plus the in-plane nonbonding MO on the nitrene nitrogen. The CASSCF and CASPT2 calculations were performed with Molcas 8.2 [46]. The molecular orbitals (MOs) were visualized with Molden [47].

## 5. Conclusions

IR and UV-Vis absorption spectroscopy verified that irradiation of azidoisoxazole **1** in cryogenic matrices results in the formation of nitrosoalkene **3**. Thus, both thermal and light activation of azidoisoxazole **1** yield the same product. Because the formation of nitrene ^3^**2** was not observed in cryogenic matrices, it was concluded that nitrosoalkene **3** is formed in a concerted manner from the singlet excited state of azidoisoxazole **1** or from nitrene ^1^**2**, which does not intersystem cross to its triplet configuration as efficiently as it cleaves to form nitrosoalkene **3**. Thus, azidoisoxazole **1** is an efficient precursor of nitrosoalkene **3**.

## Figures and Tables

**Table 1:** Computed singlet–triplet energy gaps (kcal/mol) for Nitrene **2**.

Method	Singlet Energy
CASSCF(14,13)/cc-pVDZ	13.9
CASPT2(14,13)/cc-pVDZ	15.2
CASPT2(14,13)/ANO-S	14.3
CASPT2(14,13)/cc-pVTZ	13.8

Energy of ^1^A” singlet state relative to ^3^A” triplet state. All energies were obtained at the CASSCF(14,13)/cc-pVDZ geometries and are corrected for differences in zero-point energies.

## Supplementary Materials

The following are available online at https://www.mdpi.com/1420-3049/25/3/543/s1, Figure S1: UV-Vis absorption spectra of azidoisoxazole **1**. Optimized structures of **1**–**3**; CASSCF and CASPT2 absolute energies of nitrenes ^1^**2** and ^3^**2**.
