# Supplementary material for: Photolysis of 5-Azido-3-Phenylisoxazole at Cryogenic Temperature: Formation and Direct Detection of a Nitrosoalkene
Source: Molecules. 2020 Jan 27;25(3):543. doi: 10.3390/molecules25030543 (PMC7037410; doi:10.3390/molecules25030543)
Supplement: Supplementary file 1 [file molecules-25-00543-s001.pdf]

Supporting Information

for

Photolysis of 5-Azido-3-phenylisoxazole at Cryogenic Temperature: Formation and Direct Detection of a Nitrosoalkene

Upasana Banerjee [1], William L. Karney [2], Bruce S. Ault [1] and Anna D. Gudmundsdottir [1,*]

[1] Department of Chemistry, University of Cincinnati, Cincinnati, PO Box 210172, Ohio 45221-0172, United States

[2] Department of Chemistry, University of San Francisco, 2130 Fulton Street, San Francisco, California 94117, United States

Contents

1. Characterization of azidoisoxazole 1 3

Figure S1. UV-Vis Spectrum of Azidoxazole 1 in mTHF

Figure S2. ^{1}H-NMR spectrum of Azidoxazole 1

Figure S3. IR spectrum of Azidoxazole 1

2. Quantum chemical calculations 5

A. Quantum chemical calculation using B3LYP 5

1. Optimization of 1A 5
2. Optimization of 1B 5
3. Optimization of T_1 of 1A 6
4. Optimization of nitrene ^{1}2 7
5. Optimization of nitrene ^{3}2 7
6. Optimization of nitrosoalkene ^{3}3A 8
7. Optimization of nitrosoalkene ^{3}3B 9
8. Optimization of nitrosoalkene 3A 10
9. Optimization of nitrosoalkene 3B 10
10. TD-DFT calculation of 1A 11
11. TD-DFT calculation of 1B 14
12. TD-DFT calculation of nitrene ^{1}2 17
13. TD-DFT calculation of nitrene ^{3}2 23
14. TD-DFT calculation of nitrosoalkene ^{3}3A 28
15. TD-DFT calculation of nitrosoalkene ^{3}3B 33

16. TD-DFT calculation of nitrosoalkene 3A in gas phase 37
17. TD-DFT calculation of nitrosoalkene 3A in IEFPCM 40
18. TD-DFT calculation of nitrosoalkene 3A in SMD 43
19. TD-DFT calculation of nitrosoalkene 3A in I-PCM 46
20. TD-DFT calculation of nitrosoalkene 3A in C-PCM 49
21. TD-DFT calculation of nitrosoalkene 3B in gas phase 53
22. TD-DFT calculation of nitrosoalkene 3B in IEFPCM 56
23. TD-DFT calculation of nitrosoalkene 3B in SMD 59
24. TD-DFT calculation of nitrosoalkene 3B in I-PCM 62
25. TD-DFT calculation of nitrosoalkene 3B in C-PCM 65
B. Quantum chemical calculations using CAM-B3LYP 71
1. Optimization of 1A 71
2. Optimization of 1B 71
3. Optimization of nitrene ^{1}2 72
4. Optimization of nitrene ^{3}2 73
5. Optimization of nitrosoalkene ^{3}3A 73
6. Optimization of nitrosoalkene ^{3}3B 74
7. Optimization of nitrosoalkene ^{1}3A 75
8. Optimization of nitrosoalkene ^{1}3B 75
9. TD-DFT calculation of 1A 76
10. TD-DFT calculation of 1B 80
11. TD-DFT calculation of nitrene ^{1}2 84
12. TD-DFT calculation nitrene ^{3}2 91
13. TD-DFT calculation nitrosoalkene ^{3}3A 96
14. TD-DFT calculation nitrosoalkene ^{3}3B 102
15. TD-DFT calculation of nitrosoalkene ^{1}3A in gas phase 108
16. TD-DFT calculation of nitrosoalkene ^{1}3A in IEFPCM 112
17. TD-DFT calculation of nitrosoalkene ^{1}3A in SMD 116
18. TD-DFT calculation of nitrosoalkene ^{1}3A in I-PCM 120
19. TD-DFT calculation of nitrosoalkene ^{1}3A in C-PCM 124
C. Quantum chemical calculation using M062X 128
1. Optimization of 1A 128
2. Optimization of 1B 129

3. Optimization of nitrene ^{3}2 130
4. Optimization of nitrosoalkene ^{3}3A 130
5. Optimization of nitrosoalkene ^{3}3B 131
6. Optimization of nitrosoalkene ^{1}3A 132
7. Optimization of nitrosoalkene ^{1}3B 132
8. TD-DFT calculation of 1A 133
9. TD-DFT calculation of 1B 137
10. TD-DFT calculation of nitrene ^{3}2 142
11. TD-DFT calculation of nitrosoalkene ^{3}3A 147
12. TD-DFT calculation of nitrosoalkene ^{3}3B 153
13. TD-DFT calculation of nitrosoalkene ^{1}3A in gas phase 159
14. TD-DFT calculation of nitrosoalkene ^{1}3A in IEFPCM 163
15. TD-DFT calculation of nitrosoalkene ^{1}3A in SMD 168
16. TD-DFT calculation of nitrosoalkene ^{1}3A in I-PCM 172
17. TD-DFT calculation of nitrosoalkene ^{1}3A in C-PCM 177

1. Characterization of azidoisoxazole 1

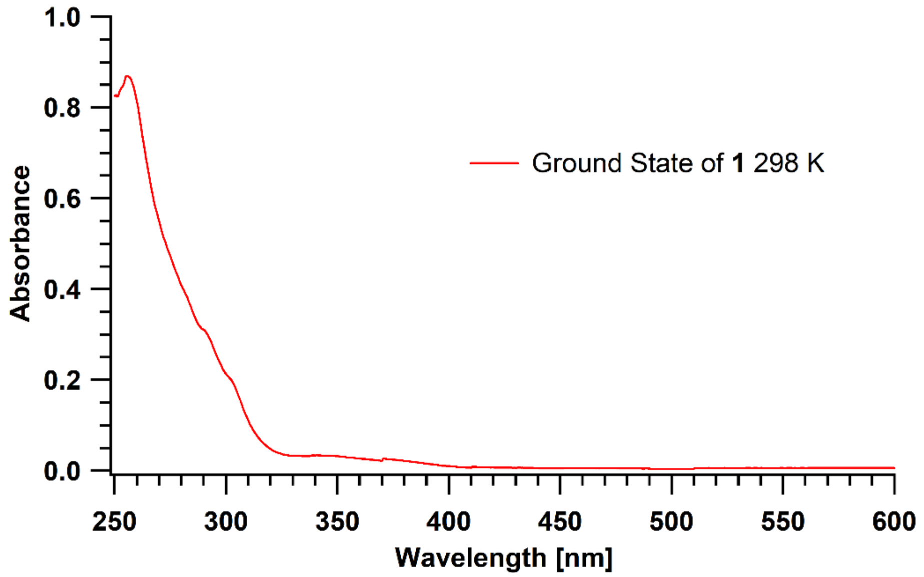

Figure S1. UV-Vis Spectrum of Azidoxazole 1 in mTHF

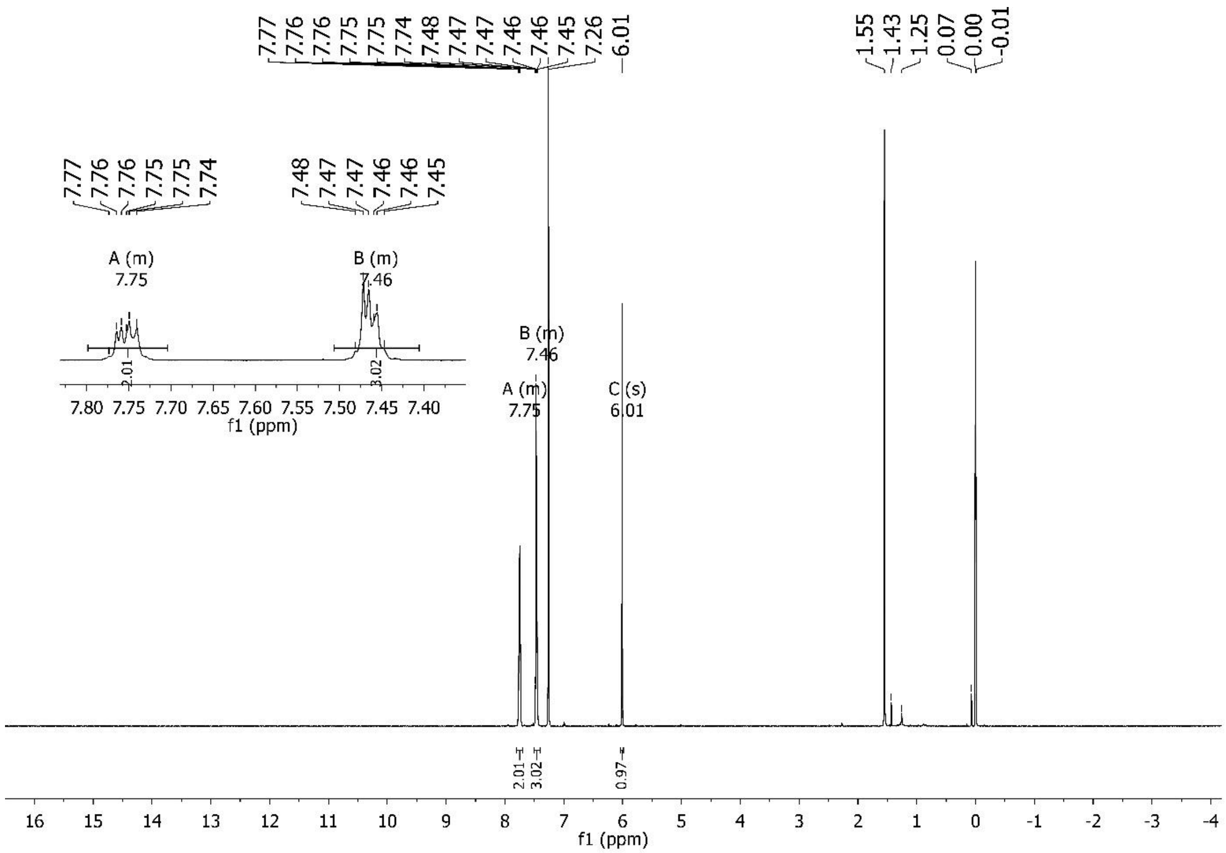

Figure S2. ^{1}H-NMR spectrum of Azidoxazole 1

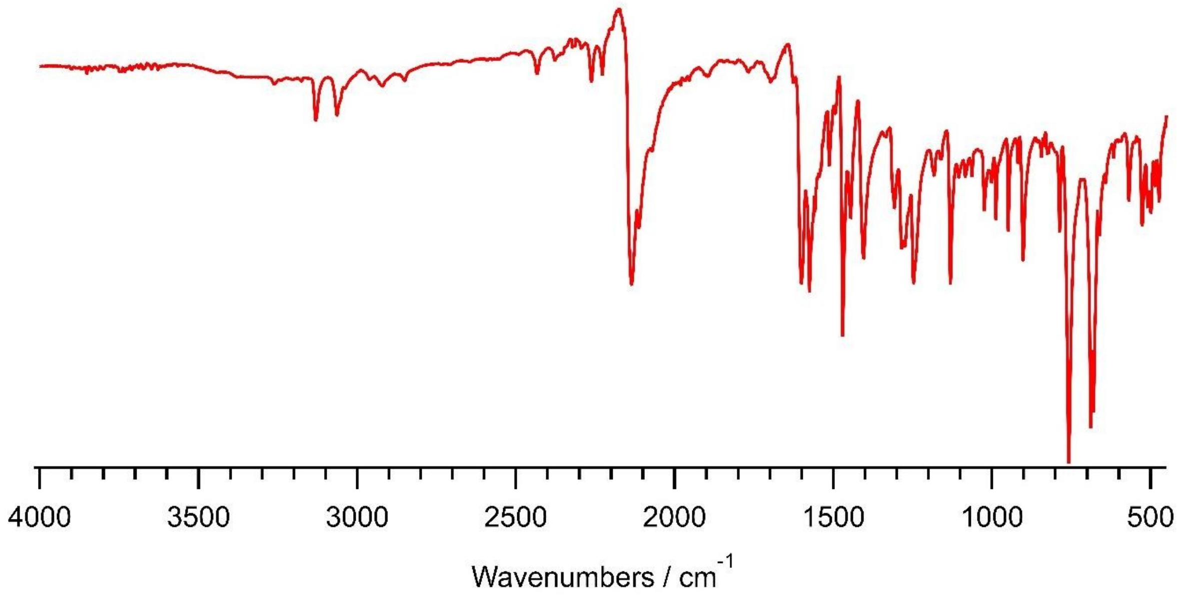

Figure S3. IR spectrum of Azidoxazole 1

2. Quantum chemical calculations

A. Quantum chemical calculation using B3LYP

1. Optimization of 1A

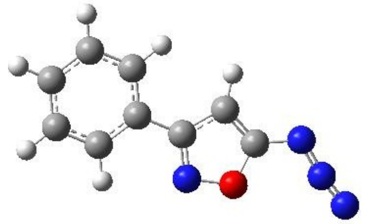

DFT/B3LYP 6-31+G(d), E = -640.706313 a.u.

Standard orientation:

Center Number	Atomic Number	Atomic Type	Coordinates (Angstroms) X	Y	Z
1	6	0	-2.024932	0.506023	-0.047962
2	6	0	-0.790301	1.092272	-0.026097
3	6	0	0.103628	-0.023778	-0.024237
4	1	0	-0.560386	2.146712	-0.013461
5	7	0	-0.557435	-1.169298	-0.043481
6	7	0	-3.338632	0.958784	-0.061467
7	7	0	-4.226565	0.096392	0.041287
8	7	0	-5.139442	-0.576784	0.126486
9	8	0	-1.918200	-0.831979	-0.058662
10	6	0	1.579010	-0.012520	-0.004361
11	6	0	2.284348	1.200777	0.016225
12	6	0	3.680428	1.210663	0.035029
13	6	0	4.390090	0.007666	0.033501
14	6	0	3.694277	-1.206262	0.013034
15	6	0	2.300844	-1.219264	-0.005762
16	1	0	1.747591	2.145254	0.017703
17	1	0	4.211342	2.158894	0.050848
18	1	0	5.476857	0.014789	0.048133
19	1	0	4.239586	-2.146451	0.011714
20	1	0	1.760784	-2.160486	-0.021640

2. Optimization of 1B

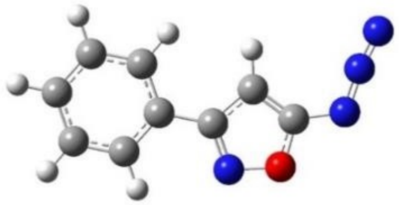

DFT/B3LYP 6-31+G(d), E = -640.704192 a.u.

Standard orientation:

Center Number	Atomic Number	Atomic Type	Coordinates (Angstroms) X	Y	Z
1	6	0	-2.117032	-0.489088	-0.058175
2	6	0	-1.096861	0.388383	0.054439
3	6	0	0.049490	-0.459351	-0.074946
4	1	0	-1.135612	1.442129	0.227053
5	7	0	-0.296790	-1.686882	-0.248333
6	7	0	-3.496614	-0.400670	-0.024691
7	7	0	-3.912964	0.761495	0.138296
8	7	0	-4.383493	1.739036	0.276699
9	8	0	-1.670163	-1.707454	-0.235232
10	6	0	1.476586	-0.071335	-0.021966
11	6	0	1.860314	1.239105	-0.284485
12	6	0	3.198155	1.603158	-0.238425
13	6	0	4.163279	0.658628	0.072908
14	6	0	3.786217	-0.651790	0.336727
15	6	0	2.451579	-1.016539	0.290561
16	1	0	1.122088	1.977872	-0.539589
17	1	0	3.482696	2.618695	-0.448053
18	1	0	5.200673	0.939471	0.110536
19	1	0	4.531131	-1.387771	0.580883
20	1	0	2.158983	-2.028658	0.497405

3. Optimization of T_1 of 1A

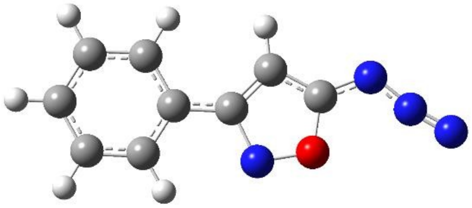

DFT/B3LYP 6-31+G(d), E = -640.604472 a.u.

Standard orientation:

Center Number	Atomic Number	Atomic Type	Coordinates (Angstroms) X	Y	Z
1	6	0	-2.040814	0.371965	-0.000013
2	6	0	-0.828104	0.994996	0.000714
3	6	0	0.104199	-0.089576	-0.000282
4	1	0	-0.645600	2.057826	0.002671
5	7	0	-0.511434	-1.260593	-0.001290
6	7	0	-3.312163	0.922090	0.000023
7	7	0	-4.210901	0.193131	0.000598
8	7	0	-5.191289	-0.380745	0.000777
9	8	0	-1.890101	-0.967160	-0.001522
10	6	0	1.578526	-0.024326	-0.000073
11	6	0	2.237513	1.214922	-0.000667
12	6	0	3.632366	1.277685	-0.000458
13	6	0	4.387072	0.102430	0.000357
14	6	0	3.737531	-1.137072	0.000956
15	6	0	2.345473	-1.202936	0.000758

16	1	0	1.664732	2.137824	-0.001424
17	1	0	4.127051	2.245425	-0.000955
18	1	0	5.472891	0.150602	0.000532
19	1	0	4.318072	-2.055950	0.001605
20	1	0	1.841604	-2.164163	0.001272

4. Optimization of nitrene [1]2

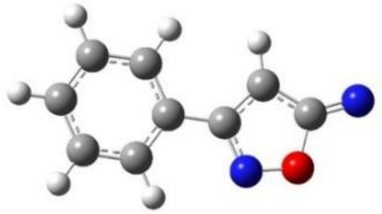

DFT/B3LYP 6-31+G(d), E =-531.157255 a.u.
Standard orientation:

Center Number	Atomic Number	Atomic Type	Coordinates (Angstroms) X	Y	Z
1	6	0	-2.895520	0.315955	0.000340
2	6	0	-1.637269	0.957865	0.000937
3	6	0	-0.693880	-0.088583	-0.000127
4	1	0	-1.478061	2.025228	0.002117
5	7	0	-1.305616	-1.279826	-0.001287
6	7	0	-4.108674	0.785294	0.000872
7	8	0	-2.667531	-1.053487	-0.001006
8	6	0	0.778891	-0.009738	-0.000045
9	6	0	1.427428	1.235489	-0.000802
10	6	0	2.820928	1.308118	-0.000697
11	6	0	3.584405	0.137805	0.000148
12	6	0	2.945231	-1.106297	0.000882
13	6	0	1.553285	-1.183077	0.000788
14	1	0	0.848441	2.154712	-0.001572
15	1	0	3.308558	2.279246	-0.001311
16	1	0	4.669685	0.194581	0.000232
17	1	0	3.532553	-2.020660	0.001550
18	1	0	1.058113	-2.148706	0.001391

5. Optimization of nitrene [3]2

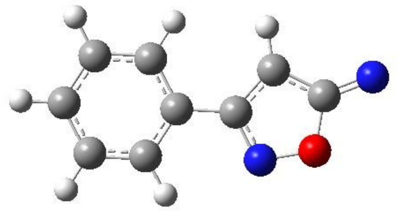

DFT/B3LYP 6-31+G(d), E = -531.175116 a.u.

Standard orientation:

Center Number	Atomic Number	Atomic Type	Coordinates (Angstroms) X	Y	Z
1	6	0	2.820716	0.309404	-0.080303
2	6	0	1.638368	0.945597	-0.224178
3	6	0	0.696184	-0.101527	0.031429
4	1	0	1.453561	1.963565	-0.491132
5	7	0	1.293555	-1.210273	0.297203
6	7	0	4.150736	0.673477	-0.183663
7	8	0	2.640602	-0.951695	0.224071
8	6	0	-0.781213	-0.019404	0.008645
9	6	0	-1.421108	1.204364	0.170950
10	6	0	-2.806074	1.281994	0.153152
11	6	0	-3.562370	0.135156	-0.029358
12	6	0	-2.929033	-1.090074	-0.192658
13	6	0	-1.547052	-1.169024	-0.174549
14	1	0	-0.845551	2.099087	0.325989
15	1	0	-3.289257	2.233573	0.284509
16	1	0	-4.636055	0.193754	-0.044933
17	1	0	-3.511424	-1.982394	-0.336749
18	1	0	-1.056628	-2.115364	-0.303802

6. Optimization of nitrosoalkene ^{3}3A

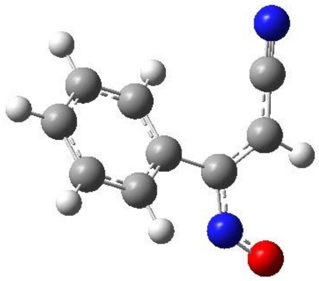

DFT/B3LYP 6-31+G(d), E = -531.186886 a.u.

Standard orientation:

Center Number	Atomic Number	Atomic Type	Coordinates (Angstroms) X	Y	Z
1	6	0	1.292884	-1.272597	-0.422602
2	6	0	0.385002	-0.274604	-0.016466
3	6	0	0.888297	0.956396	0.445973
4	6	0	2.260999	1.193539	0.476467
5	6	0	3.154936	0.202738	0.056034
6	6	0	2.666064	-1.028264	-0.389313
7	1	0	0.918080	-2.226936	-0.775201
8	1	0	0.204697	1.718256	0.810775
9	1	0	2.634210	2.146396	0.841874
10	1	0	4.225431	0.387500	0.084091
11	1	0	3.355115	-1.802958	-0.714590
12	6	0	-1.065987	-0.520208	-0.054270

13	6	0	-2.001038	0.432837	-0.309237
14	6	0	-1.816473	1.841444	-0.250865
15	7	0	-1.428000	-1.922449	0.067490
16	7	0	-1.806132	3.007186	-0.221966
17	8	0	-2.533590	-2.140446	0.519295
18	1	0	-3.017993	0.120458	-0.534306

7. Optimization of nitrosoalkene ^{3}3B

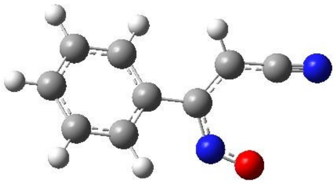

DFT/B3LYP 6-31+G(d), E = -531.186497 a.u.
Standard orientation:

Center Number	Atomic Number	Atomic Type	Coordinates (Angstroms) X	Y	Z
1	6	0	-1.529415	1.091193	-0.400072
2	6	0	-0.724118	0.001668	-0.013267
3	6	0	-1.345973	-1.197409	0.384259
4	6	0	-2.734428	-1.313454	0.370004
5	6	0	-3.526053	-0.231754	-0.031029
6	6	0	-2.919054	0.967923	-0.411849
7	1	0	-1.062803	2.021908	-0.702743
8	1	0	-0.742228	-2.030472	0.733998
9	1	0	-3.199695	-2.243341	0.685570
10	1	0	-4.608942	-0.322248	-0.037868
11	1	0	-3.528251	1.812679	-0.721711
12	6	0	0.743243	0.119513	-0.003974
13	6	0	1.598628	-0.901534	-0.274906
14	6	0	3.019320	-0.845034	-0.265518
15	7	0	1.222451	1.478402	0.185598
16	7	0	4.181385	-0.930481	-0.312428
17	8	0	2.329309	1.579904	0.674005
18	1	0	1.187695	-1.859878	-0.583360

8. Optimization of nitrosoalkene 3A

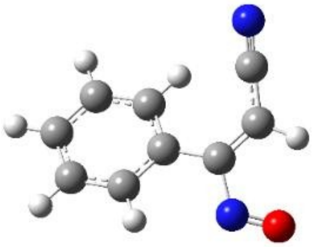

DFT/B3LYP 6-31+G(d), E = -531.195461 a.u.

Standard orientation:

Center Number	Atomic Number	Atomic Type	Coordinates (Angstroms) X	Y	Z
1	6	0	1.292884	-1.272597	-0.422602
2	6	0	0.385002	-0.274604	-0.016466
3	6	0	0.888297	0.956396	0.445973
4	6	0	2.260999	1.193539	0.476467
5	6	0	3.154936	0.202738	0.056034
6	6	0	2.666064	-1.028264	-0.389313
7	1	0	0.918080	-2.226936	-0.775201
8	1	0	0.204697	1.718256	0.810775
9	1	0	2.634210	2.146396	0.841874
10	1	0	4.225431	0.387500	0.084091
11	1	0	3.355115	-1.802958	-0.714590
12	6	0	-1.065987	-0.520208	-0.054270
13	6	0	-2.001038	0.432837	-0.309237
14	6	0	-1.816473	1.841444	-0.250865
15	7	0	-1.428000	-1.922449	0.067490
16	7	0	-1.806132	3.007186	-0.221966
17	8	0	-2.533590	-2.140446	0.519295
18	1	0	-3.017993	0.120458	-0.534306

9. Optimization of nitrosoalkene 3B

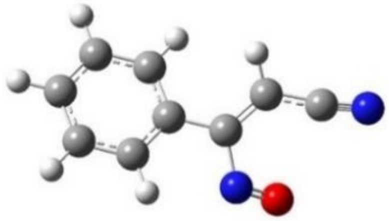

DFT/B3LYP 6-31+G(d), E = -531.191963 a.u.

Standard orientation:

Center Number	Atomic Number	Atomic Type	Coordinates (Angstroms) X	Y	Z

1	6	0	1.570069	1.180930	0.000003
2	6	0	0.793655	0.014267	0.000003
3	6	0	1.415808	-1.241459	0.000002
4	6	0	2.814375	-1.330521	-0.000003
5	6	0	3.590789	-0.163858	-0.000002
6	6	0	2.968636	1.091868	-0.000001
7	1	0	1.095041	2.139705	0.000005
8	1	0	0.822999	-2.132232	0.000003
9	1	0	3.289403	-2.289296	-0.000006
10	1	0	4.658626	-0.231860	-0.000003
11	1	0	3.561446	1.982641	-0.000002
12	6	0	-0.743232	0.112138	0.000003
13	6	0	-1.494050	-1.016064	0.000003
14	6	0	-3.030937	-0.918192	-0.000000
15	7	0	-1.395840	1.429333	-0.000001
16	7	0	-4.175219	-0.845322	-0.000003
17	8	0	-2.590221	1.505393	-0.000001
18	1	0	-1.019022	-1.974838	0.000007

10. TD-DFT calculation of 1A

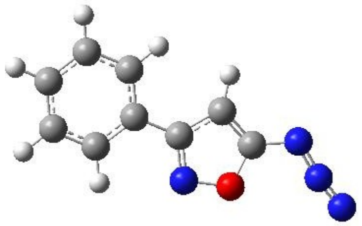

```
Excitation energies and oscillator strengths:
Excited State 1: Singlet-A 3.7234 eV 332.98 nm f=0.0002 <S**2>=0.000
43 -> 49 0.10824
47 -> 49 -0.36140
48 -> 49 0.58885
This state for optimization and/or second-order correction.
Total Energy, E(TD-HF/TD-DFT) = -640.569479353
Copying the excited state density for this state as the 1-particle RhoCI
density.

Excited State 2: Singlet-A  4.5167 eV  274.50 nm  f=0.0000 <S**2>=0.000
47 -> 49 0.59513
48 -> 49 0.37491

Excited State 3: Singlet-A  4.5947 eV  269.84 nm  f=0.0387 <S**2>=0.000
47 -> 50 0.48755
48 -> 50 0.43045
48 -> 51 -0.24991

Excited State 4: Singlet-A  4.6519 eV  266.52 nm  f=0.1847 <S**2>=0.000
45 -> 49 -0.10845
46 -> 50 -0.13782
47 -> 50 -0.40558
48 -> 50 0.52253

Excited State 5: Singlet-A  4.8161 eV  257.44 nm  f=0.0858 <S**2>=0.000
```

46 -> 50 0.59135
46 -> 51 0.16661
47 -> 50 -0.17259
47 -> 52 0.17880
48 -> 52 0.20202

Excited State 6: Singlet-A 4.9955 eV 248.19 nm f=0.0000 <S**2>=0.000
46 -> 49 0.69905

Excited State 7: Singlet-A 5.1086 eV 242.70 nm f=0.4641 <S**2>=0.000
47 -> 50 0.20350
48 -> 50 0.10359
48 -> 51 0.61775
48 -> 52 0.12531

Excited State 8: Singlet-A 5.2986 eV 233.99 nm f=0.0042 <S**2>=0.000
46 -> 50 -0.28600
46 -> 51 0.44464
47 -> 51 0.27428
47 -> 52 0.17037
48 -> 52 0.31943
Excited State 9: Singlet-A 5.4211 eV 228.71 nm f=0.0469 <S**2>=0.000
45 -> 49 -0.12783
46 -> 50 0.15376
46 -> 51 -0.22450
46 -> 52 -0.11506
47 -> 51 0.58660
47 -> 52 -0.15609

Excited State 10: Singlet-A 5.6829 eV 218.17 nm f=0.0575 <S**2>=0.000
45 -> 49 0.12802
46 -> 51 -0.16599
46 -> 52 0.12829
47 -> 51 -0.11878
47 -> 52 -0.39472
48 -> 52 0.50280

Excited State 11: Singlet-A 5.6943 eV 217.73 nm f=0.0033 <S**2>=0.000
45 -> 50 0.69543

Excited State 12: Singlet-A 5.9378 eV 208.81 nm f=0.0032 <S**2>=0.000
47 -> 53 -0.14708
47 -> 54 0.16057
48 -> 53 0.60773
48 -> 54 -0.25364

Excited State 13: Singlet-A 6.1345 eV 202.11 nm f=0.0063 <S**2>=0.000
43 -> 49 0.26628
44 -> 49 -0.25584
47 -> 53 0.36986
48 -> 53 0.28605
48 -> 54 0.34719

Excited State 14: Singlet-A 6.1410 eV 201.90 nm f=0.1652 <S**2>=0.000
43 -> 50 -0.10888
44 -> 50 0.10108
45 -> 49 0.62307

46 -> 52 -0.10092
47 -> 51 0.12753
48 -> 52 -0.11975

Excited State 15: Singlet-A 6.1562 eV 201.40 nm f=0.0014 <S**2>=0.000
43 -> 49 -0.39911
44 -> 49 0.40980
47 -> 53 0.25163
48 -> 54 0.27672

Excited State 16: Singlet-A 6.2408 eV 198.67 nm f=0.1833 <S**2>=0.000
45 -> 49 0.10831
46 -> 51 -0.40030
46 -> 59 0.10294
47 -> 52 0.47813
48 -> 52 0.20989

Excited State 17: Singlet-A 6.3448 eV 195.41 nm f=0.0000 <S**2>=0.000
46 -> 53 0.16962
47 -> 53 0.49719
47 -> 55 -0.10870
48 -> 54 -0.41363

Excited State 18: Singlet-A 6.4606 eV 191.91 nm f=0.0000 <S**2>=0.000
40 -> 50 -0.18150
42 -> 50 -0.22005
44 -> 49 0.16559
45 -> 51 0.59705

Excited State 19: Singlet-A 6.4804 eV 191.32 nm f=0.0001 <S**2>=0.000
46 -> 53 0.48418
46 -> 54 0.20686
47 -> 54 0.39998
48 -> 54 0.15807

Excited State 20: Singlet-A 6.4895 eV 191.05 nm f=0.2660 <S**2>=0.000
44 -> 50 0.46343
44 -> 51 0.10646
45 -> 49 -0.11421
46 -> 52 -0.46243

Excited State 21: Singlet-A 6.5072 eV 190.53 nm f=0.0031 <S**2>=0.000
46 -> 53 -0.34213
46 -> 54 -0.19393
47 -> 54 0.38429
48 -> 53 -0.11449
48 -> 55 0.39918

Excited State 22: Singlet-A 6.5762 eV 188.53 nm f=0.1838 <S**2>=0.000
43 -> 50 -0.16012
44 -> 50 0.40630
44 -> 51 -0.14234
46 -> 52 0.41542
47 -> 51 0.10123
47 -> 56 -0.16860
48 -> 56 -0.14679
48 -> 59 -0.10500

Excited State 23: Singlet-A 6.6155 eV 187.42 nm f=0.0000 <S**2>=0.000
43 -> 49 0.47775
44 -> 49 0.47728
45 -> 51 -0.15582

Excited State 24: Singlet-A 6.6684 eV 185.93 nm f=0.0000 <S**2>=0.000
46 -> 53 0.13360
47 -> 54 -0.33860
47 -> 55 0.20903
48 -> 55 0.52820
48 -> 57 -0.13999

Excited State 25: Singlet-A 6.7750 eV 183.00 nm f=0.0261 <S**2>=0.000
40 -> 49 -0.27794
42 -> 49 -0.22975
43 -> 50 0.47022
43 -> 51 -0.14790
44 -> 50 0.16459
44 -> 51 0.12457
48 -> 56 -0.17854
****

11. TD-DFT calculation of 1B

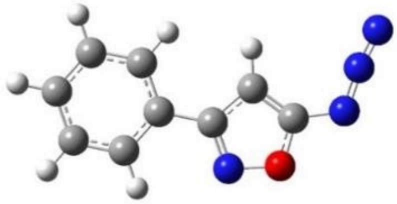

Excitation energies and oscillator strengths:

Excited State 1: Singlet-A 4.0295 eV 307.69 nm f=0.0004 <S**2>=0.000
47 -> 49 -0.40102
48 -> 49 0.56410
This state for optimization and/or second-order correction.
Total Energy, E(TD-HF/TD-KS) = -640.546570745
Copying the excited state density for this state as the 1-particle RhoCI density.

Excited State 2: Singlet-A 4.7872 eV 258.99 nm f=0.0001 <S**2>=0.000
47 -> 49 0.57006
48 -> 49 0.41192

Excited State 3: Singlet-A 4.9691 eV 249.51 nm f=0.0084 <S**2>=0.000
46 -> 50 0.15436
46 -> 51 -0.14756
47 -> 50 0.46153
48 -> 50 0.27454
48 -> 51 0.35062
48 -> 52 0.15927

Excited State 4: Singlet-A 5.0667 eV 244.70 nm f=0.1371 <S**2>=0.000
46 -> 50 -0.18652

47 -> 50 -0.24700
48 -> 50 0.59398

Excited State 5: Singlet-A 5.1122 eV 242.52 nm f=0.1173 <S**2>=0.000
46 -> 50 0.41805
46 -> 51 -0.25979
47 -> 50 -0.36569
47 -> 52 0.18826
48 -> 52 0.26274

Excited State 6: Singlet-A 5.1855 eV 239.10 nm f=0.0000 <S**2>=0.000
46 -> 49 0.69853

Excited State 7: Singlet-A 5.2697 eV 235.28 nm f=0.3205 <S**2>=0.000
46 -> 51 0.12122
47 -> 50 -0.26397
47 -> 51 -0.14707
48 -> 50 -0.19201
48 -> 51 0.56563
48 -> 52 -0.11239

Excited State 8: Singlet-A 5.4577 eV 227.17 nm f=0.0105 <S**2>=0.000
46 -> 50 0.38861
46 -> 51 0.32019
47 -> 51 0.42120
48 -> 52 -0.22658

Excited State 9: Singlet-A 5.5474 eV 223.50 nm f=0.0808 <S**2>=0.000
46 -> 50 -0.29738
46 -> 51 -0.29176
46 -> 52 0.13518
47 -> 51 0.49645
47 -> 52 0.13830
48 -> 51 0.15644

Excited State 10: Singlet-A 5.7836 eV 214.37 nm f=0.0329 <S**2>=0.000
46 -> 51 0.14766
46 -> 52 0.12801
47 -> 51 0.11485
47 -> 52 -0.42235
48 -> 52 0.48299
48 -> 53 0.13027

Excited State 11: Singlet-A 5.9326 eV 208.99 nm f=0.0052 <S**2>=0.000
47 -> 52 0.10489
47 -> 53 -0.24759
47 -> 54 -0.12199
48 -> 53 0.61798
48 -> 54 0.10359

Excited State 12: Singlet-A 6.1244 eV 202.44 nm f=0.0065 <S**2>=0.000
45 -> 50 0.67104
45 -> 51 -0.15415

Excited State 13: Singlet-A 6.1683 eV 201.00 nm f=0.0086 <S**2>=0.000
47 -> 52 0.10551
47 -> 53 0.55035

48 -> 53 0.26300
48 -> 54 -0.30206

Excited State 14: Singlet-A 6.2779 eV 197.49 nm f=0.0045 <S**2>=0.000
43 -> 49 0.12560
44 -> 49 0.22581
45 -> 49 0.63553

Excited State 15: Singlet-A 6.3316 eV 195.82 nm f=0.0222 <S**2>=0.000
42 -> 49 0.12416
43 -> 49 0.28275
44 -> 49 0.51439
45 -> 49 -0.23034
46 -> 51 0.13486
47 -> 52 0.15215

Excited State 16: Singlet-A 6.3382 eV 195.62 nm f=0.1420 <S**2>=0.000
44 -> 49 -0.16513
45 -> 49 0.16881
46 -> 50 -0.10879
46 -> 51 0.34158
47 -> 52 0.40130
48 -> 52 0.22012
48 -> 54 0.19334

Excited State 17: Singlet-A 6.4117 eV 193.37 nm f=0.0173 <S**2>=0.000
46 -> 53 0.36919
46 -> 54 -0.20161
47 -> 53 0.29335
48 -> 54 0.44270

Excited State 18: Singlet-A 6.4664 eV 191.74 nm f=0.0270 <S**2>=0.000
46 -> 52 0.15449
46 -> 53 0.47018
46 -> 54 -0.21277
47 -> 53 -0.16100
47 -> 54 -0.21687
48 -> 54 -0.33041

Excited State 19: Singlet-A 6.5773 eV 188.50 nm f=0.4787 <S**2>=0.000
44 -> 51 0.16360
46 -> 52 0.59363
46 -> 53 -0.11661
47 -> 54 0.12082

Excited State 20: Singlet-A 6.6289 eV 187.04 nm f=0.0149 <S**2>=0.000
46 -> 53 0.13543
47 -> 54 0.51971
48 -> 53 0.10423
48 -> 54 -0.12484
48 -> 55 -0.35479
48 -> 57 -0.11892

Excited State 21: Singlet-A 6.6526 eV 186.37 nm f=0.0061 <S**2>=0.000
45 -> 50 0.15376
45 -> 51 0.66704

Excited State 22: Singlet-A 6.7635 eV 183.31 nm f=0.0273 <S**2>=0.000
40 -> 49 0.12954
42 -> 49 0.17250
44 -> 50 0.49629
47 -> 54 0.16015
48 -> 55 0.21176
48 -> 56 0.23960

Excited State 23: Singlet-A 6.7838 eV 182.77 nm f=0.0042 <S**2>=0.000
44 -> 50 -0.16988
46 -> 53 0.11832
46 -> 54 0.12874
47 -> 54 0.25866
47 -> 55 0.10961
48 -> 55 0.50762
48 -> 56 -0.21435

Excited State 24: Singlet-A 6.8166 eV 181.89 nm f=0.0028 <S**2>=0.000
46 -> 53 0.26372
46 -> 54 0.57296
46 -> 55 -0.15617
47 -> 55 -0.18919
48 -> 55 -0.13725
48 -> 57 0.10294

Excited State 25: Singlet-A 6.8938 eV 179.85 nm f=0.0023 <S**2>=0.000
40 -> 50 0.11075
41 -> 49 0.11526
42 -> 49 0.17409
42 -> 50 0.13282
43 -> 49 0.46590
43 -> 50 -0.13153
44 -> 49 -0.36105
SavETr: write IOETrn= 770 NScale= 10 NData= 16 NLR=1 NState= 25 LETran= 460.

****

12. TD-DFT calculation of nitrene ^{1}2

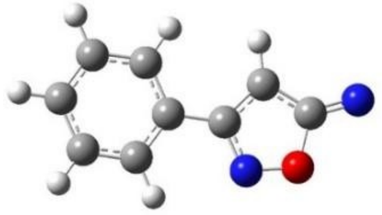

Excitation energies and oscillator strengths:
Excited State 1: 1.776-A -0.5967 eV -2077.66 nm f=-0.0000
<S**2>=0.539
40A -> 42A 0.30387
41A -> 42A 0.65651
40B -> 42B -0.30387
41B -> 42B 0.65651
41A <- 42A 0.17392
41B <- 42B 0.17392

```
 This state for optimization and/or second-order correction.
 Total Energy, E(TD-HF/TD-KS) =  -531.179185154
 Copying the excited state density for this state as the 1-particle RhoCI
density.

 Excited State   2:  1.798-A      1.3904 eV  891.72 nm  f=0.0106
<S**2>=0.558
     36A -> 42A         0.11607
     40A -> 42A         0.29885
     41A -> 42A         0.62171
     36B -> 42B         0.11607
     40B -> 42B         0.29885
     41B -> 42B        -0.62171
     41A <- 42A        -0.10389
     41B <- 42B         0.10389

 Excited State   3:  2.614-A      1.8753 eV  661.16 nm  f=0.0000
<S**2>=1.458
     37A -> 42A        -0.14736
     40A -> 42A        -0.61267
     41A -> 42A         0.29152
     37B -> 42B         0.14737
     40B -> 42B         0.61268
     41B -> 42B         0.29152

 Excited State   4:  1.912-A      2.3941 eV  517.88 nm  f=0.0059
<S**2>=0.664
     40A -> 42A         0.63603
     41A -> 42A        -0.30223
     40B -> 42B         0.63603
     41B -> 42B         0.30222

 Excited State   5:  2.604-A      2.7014 eV  458.97 nm  f=0.0000
<S**2>=1.446
     39A -> 42A        -0.69074
     39B -> 42B         0.69077

 Excited State   6:  1.909-A      2.7306 eV  454.06 nm  f=0.0047
<S**2>=0.661
     39A -> 42A         0.70371
     39B -> 42B         0.70368

 Excited State   7:  2.581-A      2.9863 eV  415.18 nm  f=0.0002
<S**2>=1.415
     36A -> 42A        -0.47401
     37A -> 42A         0.26298
     38A -> 42A         0.34951
     39A -> 42A        -0.13051
     40A -> 43A         0.10667
     41A -> 43A        -0.18069
     36B -> 42B         0.47401
     37B -> 42B        -0.26295
     38B -> 42B         0.34953
     39B -> 42B         0.13050
     40B -> 43B        -0.10667
     41B -> 43B        -0.18069
```

```
 Excited State   8:  3.391-A      3.3861 eV  366.15 nm  f=0.0001
<S**2>=2.624
     36A -> 42A        -0.10010
     37A -> 42A         0.14886
     38A -> 42A         0.13775
     39A -> 44A         0.26924
     40A -> 43A        -0.45772
     41A -> 43A         0.37969
     36B -> 42B         0.10012
     37B -> 42B        -0.14885
     38B -> 42B         0.13773
     39B -> 44B        -0.26923
     40B -> 43B         0.45773
     41B -> 43B         0.37967

 Excited State   9:  2.649-A      3.4051 eV  364.11 nm  f=0.0176
<S**2>=1.504
     35A -> 42A         0.15237
     36A -> 42A        -0.27168
     38A -> 42A        -0.57139
     38A -> 43A         0.10057
     41A -> 43A        -0.15323
     35B -> 42B        -0.15238
     36B -> 42B        -0.27168
     38B -> 42B         0.57140
     38B -> 43B        -0.10057
     41B -> 43B         0.15326

 Excited State  10:  2.003-A      3.7145 eV  333.78 nm  f=0.0038
<S**2>=0.753
     33A -> 42A        -0.13737
     35A -> 42A        -0.15275
     36A -> 42A         0.30809
     37A -> 42A        -0.17158
     38A -> 42A         0.55960
     33B -> 42B        -0.13737
     35B -> 42B        -0.15275
     36B -> 42B        -0.30809
     37B -> 42B         0.17161
     38B -> 42B         0.55959

 Excited State  11:  2.619-A      3.8853 eV  319.11 nm  f=0.0002
<S**2>=1.465
     32A -> 42A         0.11776
     36A -> 42A        -0.23258
     37A -> 42A        -0.54011
     39A -> 44A         0.11947
     40A -> 42A         0.17709
     40A -> 43A        -0.19572
     41A -> 43A        -0.16897
     32B -> 42B        -0.11776
     36B -> 42B         0.23257
     37B -> 42B         0.54012
     39B -> 44B        -0.11947
     40B -> 42B        -0.17709
     40B -> 43B         0.19572
     41B -> 43B        -0.16897
```

```
 Excited State  12:  2.567-A      4.1169 eV  301.16 nm  f=0.0082
<S**2>=1.398
     33A -> 42A         0.60494
     35A -> 42A         0.23682
     38A -> 42A         0.11180
     41A -> 43A         0.14401
     33B -> 42B        -0.60493
     35B -> 42B        -0.23681
     38B -> 42B        -0.11180
     41B -> 43B        -0.14399

 Excited State  13:  3.173-A      4.2226 eV  293.62 nm  f=0.0001
<S**2>=2.267
     33A -> 42A         0.15251
     36A -> 42A        -0.10124
     39A -> 43A         0.50481
     39A -> 44A        -0.11642
     40A -> 43A         0.19351
     40A -> 44A        -0.15338
     41A -> 43A         0.31571
     33B -> 42B         0.15252
     36B -> 42B         0.10124
     39B -> 43B        -0.50481
     39B -> 44B         0.11642
     40B -> 43B        -0.19351
     40B -> 44B         0.15338
     41B -> 43B         0.31572

 Excited State  14:  2.703-A      4.2927 eV  288.83 nm  f=0.0006
<S**2>=1.577
     33A -> 42A        -0.36547
     35A -> 42A        -0.16565
     36A -> 42A         0.10236
     37A -> 42A         0.12701
     38A -> 42A        -0.11497
     39A -> 43A         0.39327
     39A -> 44A         0.15785
     40A -> 43A        -0.10887
     41A -> 43A        -0.26732
     33B -> 42B        -0.36552
     35B -> 42B        -0.16567
     36B -> 42B        -0.10232
     37B -> 42B        -0.12686
     38B -> 42B        -0.11497
     39B -> 43B        -0.39327
     39B -> 44B        -0.15785
     40B -> 43B         0.10885
     41B -> 43B        -0.26725

 Excited State  15:  2.090-A      4.3214 eV  286.91 nm  f=0.0013
<S**2>=0.842
     33A -> 42A         0.11712
     36A -> 42A         0.14214
     37A -> 42A         0.59510
     41A -> 43A        -0.29939
     33B -> 42B        -0.11710
```

```
     36B -> 42B         0.14219
     37B -> 42B         0.59514
     41B -> 43B         0.29952

 Excited State  16:  2.391-A      4.3851 eV  282.74 nm  f=0.0013
<S**2>=1.179
     33A -> 42A         0.46406
     35A -> 42A         0.18127
     36A -> 42A         0.17310
     37A -> 42A         0.13300
     38A -> 42A         0.12792
     39A -> 43A         0.11921
     39A -> 44A         0.26408
     41A -> 43A        -0.27604
     33B -> 42B         0.46404
     35B -> 42B         0.18126
     36B -> 42B        -0.17308
     37B -> 42B        -0.13291
     38B -> 42B         0.12792
     39B -> 43B        -0.11921
     39B -> 44B        -0.26408
     41B -> 43B        -0.27599

 Excited State  17:  3.255-A      4.4835 eV  276.53 nm  f=0.0001
<S**2>=2.398
     33A -> 42A        -0.10019
     39A -> 43A        -0.10412
     39A -> 44A         0.52291
     40A -> 43A         0.40344
     41A -> 43A         0.11977
     33B -> 42B        -0.10018
     39B -> 43B         0.10412
     39B -> 44B        -0.52291
     40B -> 43B        -0.40344
     41B -> 43B         0.11977

 Excited State  18:  2.147-A      4.5382 eV  273.20 nm  f=0.0813
<S**2>=0.902
     33A -> 42A         0.12432
     36A -> 42A         0.44853
     37A -> 42A        -0.32362
     40A -> 43A        -0.12357
     41A -> 43A        -0.34870
     33B -> 42B        -0.12433
     36B -> 42B         0.44853
     37B -> 42B        -0.32361
     40B -> 43B        -0.12356
     41B -> 43B         0.34871

 Excited State  19:  3.503-A      4.8252 eV  256.95 nm  f=0.0000
<S**2>=2.818
     39A -> 43A        -0.22616
     40A -> 44A        -0.52478
     41A -> 44A         0.40101
     39B -> 43B         0.22616
     40B -> 44B         0.52479
     41B -> 44B         0.40100
```

```
 Excited State  20:  2.181-A      4.8593 eV  255.15 nm  f=0.0788
<S**2>=0.939
     36A -> 42A        -0.31200
     37A -> 42A        -0.11128
     38A -> 42A         0.26542
     39A -> 43A        -0.30503
     40A -> 43A        -0.21019
     40A -> 44A        -0.13273
     41A -> 43A        -0.32723
     41A -> 44A         0.13486
     36B -> 42B        -0.31200
     37B -> 42B        -0.11130
     38B -> 42B        -0.26541
     39B -> 43B        -0.30502
     40B -> 43B        -0.21019
     40B -> 44B        -0.13272
     41B -> 43B         0.32724
     41B -> 44B        -0.13485

 Excited State  21:  2.173-A      4.9366 eV  251.15 nm  f=0.0567
<S**2>=0.930
     36A -> 42A        -0.16726
     38A -> 42A         0.17404
     39A -> 43A         0.49378
     40A -> 43A        -0.11130
     40A -> 44A         0.27512
     41A -> 43A        -0.18667
     41A -> 44A        -0.21758
     36B -> 42B        -0.16726
     38B -> 42B        -0.17403
     39B -> 43B         0.49379
     40B -> 43B        -0.11130
     40B -> 44B         0.27514
     41B -> 43B         0.18667
     41B -> 44B         0.21758

 Excited State  22:  2.154-A      5.1128 eV  242.50 nm  f=0.3607
<S**2>=0.910
     39A -> 44A        -0.12789
     40A -> 43A         0.61013
     41A -> 43A        -0.26768
     39B -> 44B        -0.12788
     40B -> 43B         0.61013
     41B -> 43B         0.26768

 Excited State  23:  2.598-A      5.2379 eV  236.71 nm  f=0.0147
<S**2>=1.437
     33A -> 42A         0.24863
     35A -> 42A        -0.62024
     36A -> 42A        -0.12130
     41A -> 43A        -0.10081
     33B -> 42B        -0.24868
     35B -> 42B         0.62034
     36B -> 42B        -0.12133
     41B -> 43B         0.10081
```

```
 Excited State  24:  1.911-A      5.2538 eV  235.99 nm  f=0.0000
<S**2>=0.663
     33A -> 42A        -0.28872
     35A -> 42A         0.62915
     33B -> 42B        -0.28868
     35B -> 42B         0.62905

 Excited State  25:  2.609-A      5.3014 eV  233.87 nm  f=0.0080
<S**2>=1.452
     31A -> 42A         0.19084
     34A -> 42A        -0.64838
     36A -> 42A         0.14119
     31B -> 42B        -0.19085
     34B -> 42B         0.64843
     36B -> 42B         0.14119
 **********************************************************************
```

13. TD-DFT calculation of nitrene ^{3}2

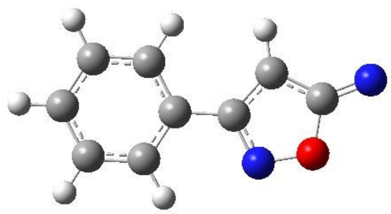

```
Excitation energies and oscillator strengths:
Excited State 1: 3.042-A 2.2871 eV 542.11 nm f=0.0066 <S**2>=2.064
42A -> 43A 0.19010
36B -> 41B 0.12998
40B -> 41B 0.96538
This state for optimization and/or second-order correction.
Total Energy, E(TD-HF/TD-KS) = -531.091067414
Copying the excited state density for this state as the 1-particle RhoCI
density.

Excited State 2: 3.045-A 2.6537 eV 467.21 nm f=0.0000 <S**2>=2.069
36B -> 42B 0.15046
40B -> 42B 0.98705

Excited State 3: 3.072-A 3.0131 eV 411.48 nm f=0.0052 <S**2>=2.110
39B -> 41B 0.98944

Excited State 4: 3.044-A 3.2942 eV 376.38 nm f=0.0001 <S**2>=2.066
36B -> 42B 0.18546
37B -> 41B -0.11045
38B -> 42B 0.62776
39B -> 42B 0.74325

Excited State 5: 3.920-A 3.3489 eV 370.22 nm f=0.0040 <S**2>=3.592
40A -> 44A 0.23309
41A -> 43A 0.46301
41A -> 46A 0.11961
42A -> 43A -0.42019
36B -> 41B -0.15084
38B -> 41B -0.29811
```

39B -> 41B 0.12553
39B -> 44B 0.24575
40B -> 41B 0.10691
40B -> 43B 0.55752

Excited State 6: 3.050-A 3.4216 eV 362.36 nm f=0.0004 <S**2>=2.076
36B -> 42B -0.19939
37B -> 41B 0.32175
38B -> 42B -0.64408
39B -> 42B 0.65366
Excited State 7: 3.093-A 3.4940 eV 354.85 nm f=0.0018 <S**2>=2.141
35B -> 41B 0.10523
37B -> 41B 0.90682
37B -> 43B -0.12615
38B -> 42B 0.30877
39B -> 42B -0.14170

Excited State 8: 3.233-A 3.6460 eV 340.05 nm f=0.0333 <S**2>=2.363
40A -> 44A 0.16457
41A -> 43A 0.27102
42A -> 43A 0.10913
42A -> 46A -0.20065
36B -> 41B 0.17663
37B -> 42B 0.19611
38B -> 41B 0.81394
39B -> 44B 0.15411
40B -> 43B 0.22801

Excited State 9: 3.061-A 3.8379 eV 323.06 nm f=0.0086 <S**2>=2.092
41A -> 43A 0.12445
42A -> 43A 0.21800
33B -> 42B 0.60736
34B -> 42B 0.15916
35B -> 42B -0.38399
37B -> 42B -0.59759

Excited State 10: 3.137-A 4.0610 eV 305.31 nm f=0.0037 <S**2>=2.210
40A -> 44A 0.12181
41A -> 43A 0.24077
42A -> 43A 0.56831
32B -> 41B 0.10733
33B -> 42B -0.22909
35B -> 42B 0.12468
36B -> 41B 0.52575
38B -> 41B -0.36716
39B -> 44B 0.13202
40B -> 41B -0.21981

Excited State 11: 4.073-A 4.2307 eV 293.06 nm f=0.0003 <S**2>=3.898
40A -> 43A 0.74485
41A -> 43A -0.10987
41A -> 44A 0.14563
42A -> 43A -0.12232
36B -> 41B 0.14199
39B -> 43B 0.51012
40B -> 44B 0.28666
Excited State 12: 4.032-A 4.4605 eV 277.96 nm f=0.0000 <S**2>=3.814
40A -> 44A 0.57038
41A -> 43A -0.27725
42A -> 44A -0.16584
33B -> 42B -0.12128
36B -> 41B -0.23959
37B -> 42B -0.14747
39B -> 44B 0.58529
40B -> 43B -0.31520

Excited State 13: 3.169-A 4.4954 eV 275.80 nm f=0.0007 <S**2>=2.260
40A -> 43A -0.13299
40A -> 44A 0.15342
41A -> 43A -0.20158
42A -> 43A -0.22271
33B -> 42B 0.45784
34B -> 42B 0.15784
35B -> 42B -0.21823
36B -> 41B 0.42142
37B -> 42B 0.56635
38B -> 41B -0.13276
39B -> 44B 0.16783

Excited State 14: 3.061-A 4.5640 eV 271.65 nm f=0.0033 <S**2>=2.092
33B -> 41B 0.79703
34B -> 41B 0.26883
35B -> 41B -0.50094

Excited State 15: 3.054-A 4.7234 eV 262.49 nm f=0.0001 <S**2>=2.082
32B -> 42B 0.13747
36B -> 42B 0.93024
38B -> 42B -0.30147
40B -> 42B -0.13806

Excited State 16: 3.874-A 4.8264 eV 256.89 nm f=0.0182 <S**2>=3.502
40A -> 43A -0.15730
41A -> 43A 0.17938
41A -> 44A 0.45859

42A -> 43A 0.23367
42A -> 44A -0.30036
36B -> 41B -0.30562
37B -> 42B 0.21756
39B -> 43B -0.12070
40B -> 44B 0.61751

Excited State 17: 3.372-A 4.8467 eV 255.81 nm f=0.0670 <S**2>=2.593
40A -> 43A -0.12411
41A -> 43A -0.20522
41A -> 44A 0.20081
42A -> 43A -0.40269
42A -> 46A -0.18265
33B -> 42B -0.17831
35B -> 42B 0.10280
36B -> 41B 0.43315
37B -> 42B -0.39757
39B -> 43B -0.34778
40B -> 44B 0.39389

Excited State 18: 3.098-A 4.9621 eV 249.86 nm f=0.0262 <S**2>=2.150
40A -> 43A -0.48476
41A -> 44A 0.43129
42A -> 44A -0.23136
36B -> 41B 0.14063
37B -> 42B -0.10563
39B -> 43B 0.63031
40B -> 44B -0.26545

Excited State 19: 3.055-A 5.1184 eV 242.23 nm f=0.3689 <S**2>=2.083
40A -> 44A -0.10178
41A -> 43A -0.60820
42A -> 43A 0.27913
42A -> 44A 0.15062
39B -> 44B 0.15653
40B -> 43B 0.67249

Excited State 20: 3.375-A 5.4442 eV 227.74 nm f=0.0002 <S**2>=2.598
37A -> 43A -0.17803
39A -> 43A 0.76941
39A -> 46A -0.14688
33B -> 41B -0.16873
34B -> 41B -0.16239
35B -> 41B -0.45881
37B -> 41B 0.18095
37B -> 43B 0.18020

Excited State 21: 3.073-A 5.5267 eV 224.34 nm f=0.0001 <S**2>=2.110
39A -> 43A 0.29017
31B -> 41B 0.27227
33B -> 41B 0.52960
34B -> 41B -0.51473
35B -> 41B 0.53143

Excited State 22: 3.093-A 5.5643 eV 222.82 nm f=0.0955 <S**2>=2.141
40A -> 44A 0.21097
41A -> 44A 0.32708
42A -> 44A 0.86559
39B -> 43B 0.10448
40B -> 43B -0.11553
40B -> 44B 0.11286

Excited State 23: 3.103-A 5.5646 eV 222.81 nm f=0.0008 <S**2>=2.158
39A -> 43A 0.42874
31B -> 41B -0.15399
34B -> 41B 0.73246
35B -> 41B 0.47296

Excited State 24: 4.050-A 5.6248 eV 220.42 nm f=0.0004 <S**2>=3.851
38A -> 43A 0.54436
40A -> 44A 0.10855
41A -> 43A 0.13602
41A -> 46A -0.30177
41A -> 51A -0.15714
42A -> 46A 0.22167
36B -> 41B 0.13192
36B -> 43B 0.45141
38B -> 43B -0.19827
40B -> 48B -0.34953
40B -> 51B -0.17740

Excited State 25: 3.061-A 5.8774 eV 210.95 nm f=0.0047 <S**2>=2.092
31B -> 42B 0.10387
33B -> 42B 0.48050
35B -> 42B 0.85859
SavETr: write IOETrn= 770 NScale= 10 NData= 16 NLR=1 NState= 25 LETran= 460.

14. TD-DFT calculation of nitrosoalkene ^{3}3A

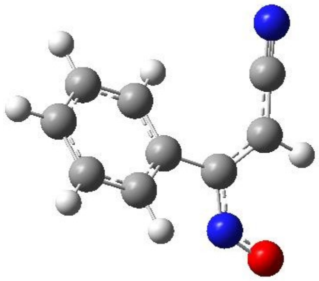

```
Excitation energies and oscillator strengths:
 Excited State   1:  3.022-A      1.8976 eV  653.37 nm  f=0.0002
<S**2>=2.033
     38B -> 41B       -0.25041
     40B -> 41B        0.96091
 This state for optimization and/or second-order correction.
 Total Energy, E(TD-HF/TD-DFT) =  -531.117149980
 Copying the excited state density for this state as the 1-particle RhoCI
density.

 Excited State   2:  3.078-A      2.3920 eV  518.33 nm  f=0.0272
<S**2>=2.119
     42A -> 43A       -0.31034
     42A -> 45A        0.10105
     38B -> 42B       -0.16003
     40B -> 42B        0.92144

 Excited State   3:  3.047-A      2.7174 eV  456.27 nm  f=0.0028
<S**2>=2.070
     39B -> 41B        0.99709

 Excited State   4:  3.094-A      3.0895 eV  401.31 nm  f=0.0007
<S**2>=2.144
     39B -> 42B        0.99460

 Excited State   5:  3.060-A      3.2998 eV  375.73 nm  f=0.0003
<S**2>=2.091
     32B -> 41B        0.17176
     38B -> 41B        0.92140
     40B -> 41B        0.26322

 Excited State   6:  3.668-A      3.5030 eV  353.94 nm  f=0.0010
<S**2>=3.113
     40A -> 44A        0.25422
     41A -> 43A        0.42356
     41A -> 45A        0.13083
     42A -> 43A        0.45223
     38B -> 41B       -0.14975
     38B -> 42B        0.45633
     39B -> 44B       -0.25513
     40B -> 42B        0.17247
     40B -> 43B       -0.39946
```

```
 Excited State   7:  3.527-A      3.7090 eV  334.28 nm  f=0.0068
<S**2>=2.860
     40A -> 44A        -0.26009
     41A -> 43A        -0.39494
     42A -> 43A         0.38147
     38B -> 42B         0.54017
     39B -> 44B         0.24343
     40B -> 42B         0.30424
     40B -> 43B         0.36469

 Excited State   8:  3.095-A      4.2456 eV  292.03 nm  f=0.0477
<S**2>=2.145
     40A -> 43A         0.15368
     42A -> 43A         0.50659
     31B -> 41B         0.13226
     37B -> 41B         0.57270
     37B -> 42B         0.37049
     38B -> 42B        -0.42722

 Excited State   9:  3.182-A      4.2625 eV  290.87 nm  f=0.0113
<S**2>=2.282
     40A -> 43A        -0.19076
     42A -> 43A        -0.23480
     37B -> 41B        -0.10075
     37B -> 42B         0.87689
     38B -> 42B         0.24643

 Excited State  10:  3.922-A      4.3174 eV  287.17 nm  f=0.0073
<S**2>=3.596
     40A -> 43A         0.76908
     40A -> 45A         0.11877
     41A -> 44A         0.11518
     42A -> 43A        -0.11809
     37B -> 41B        -0.17021
     37B -> 42B         0.14231
     38B -> 42B         0.13293
     39B -> 43B        -0.42492
     40B -> 44B        -0.28430

 Excited State  11:  4.072-A      4.4726 eV  277.21 nm  f=0.0046
<S**2>=3.896
     40A -> 44A        -0.56995
     41A -> 43A         0.39343
     37B -> 41B        -0.15793
     39B -> 44B         0.57588
     40B -> 43B        -0.33586

 Excited State  12:  3.167-A      4.5864 eV  270.33 nm  f=0.0925
<S**2>=2.257
     40A -> 44A        -0.10696
     41A -> 43A         0.17151
     42A -> 43A        -0.41828
     42A -> 45A        -0.15591
     37B -> 41B         0.72650
     37B -> 42B        -0.11567
     38B -> 42B         0.31833
     39B -> 44B         0.11277
```

```
 Excited State  13:  4.006-A      4.8752 eV  254.32 nm  f=0.0019
<S**2>=3.762
     40A -> 43A         0.30423
     41A -> 44A        -0.59508
     42A -> 44A        -0.27999
     38B -> 44B         0.12247
     40B -> 44B         0.65589

 Excited State  14:  3.214-A      4.9934 eV  248.29 nm  f=0.0103
<S**2>=2.332
     38A -> 43A         0.15011
     41A -> 43A         0.20114
     42A -> 43A         0.10236
     42A -> 44A         0.19804
     29B -> 41B         0.11306
     31B -> 41B         0.41377
     32B -> 41B        -0.12143
     32B -> 42B        -0.20774
     33B -> 41B        -0.15861
     33B -> 42B         0.26228
     34B -> 41B         0.30080
     34B -> 42B         0.30717
     35B -> 42B        -0.28766
     36B -> 41B         0.12632
     37B -> 41B        -0.18880
     38B -> 43B        -0.19445
     40B -> 43B         0.24257
     40B -> 46B         0.10578
     40B -> 47B         0.11764

 Excited State  15:  3.211-A      5.0276 eV  246.61 nm  f=0.0015
<S**2>=2.327
     39A -> 43A        -0.12277
     40A -> 43A        -0.14967
     42A -> 44A         0.70230
     42A -> 47A         0.10487
     31B -> 41B        -0.11575
     34B -> 42B        -0.10699
     35B -> 42B         0.11836
     39B -> 43B        -0.45709
     40B -> 44B         0.36058

 Excited State  16:  3.151-A      5.0810 eV  244.02 nm  f=0.0076
<S**2>=2.232
     39A -> 43A         0.12329
     40A -> 43A         0.30851
     41A -> 44A        -0.40533
     42A -> 44A         0.54722
     42A -> 47A         0.10659
     39B -> 43B         0.57895
     40B -> 44B        -0.20614

 Excited State  17:  3.098-A      5.1414 eV  241.15 nm  f=0.0187
<S**2>=2.149
     39A -> 43A         0.53353
     39A -> 45A        -0.10278
```

```
     41A -> 43A       -0.18760
     42A -> 44A        0.16467
     27B -> 41B        0.11278
     28B -> 41B       -0.15216
     31B -> 42B        0.11085
     32B -> 41B        0.22944
     33B -> 41B       -0.17296
     34B -> 41B       -0.22951
     34B -> 42B        0.22550
     35B -> 41B        0.32286
     36B -> 41B       -0.38604
     38B -> 41B       -0.13566
     40B -> 43B       -0.18335

 Excited State  18:  3.140-A      5.1488 eV  240.80 nm  f=0.1609
<S**2>=2.215
     39A -> 43A        0.30206
     40A -> 44A        0.14863
     41A -> 43A        0.51823
     41A -> 45A       -0.14981
     32B -> 41B        0.10675
     33B -> 42B       -0.10545
     34B -> 41B       -0.21810
     35B -> 41B        0.23591
     35B -> 42B        0.15414
     36B -> 41B        0.20956
     40B -> 43B        0.55541

 Excited State  19:  3.173-A      5.1822 eV  239.25 nm  f=0.0020
<S**2>=2.268
     39A -> 43A       -0.21474
     34B -> 42B        0.65988
     35B -> 42B        0.63144
     36B -> 42B        0.22022

 Excited State  20:  3.054-A      5.2225 eV  237.40 nm  f=0.0125
<S**2>=2.082
     39A -> 43A        0.67218
     39A -> 45A       -0.10390
     41A -> 43A       -0.11104
     41A -> 44A        0.10001
     28B -> 41B        0.10229
     32B -> 41B       -0.18040
     33B -> 41B        0.20957
     34B -> 41B        0.26577
     35B -> 41B       -0.36052
     35B -> 42B        0.12378
     36B -> 41B        0.35444
     38B -> 41B        0.11916
     40B -> 43B       -0.12601

 Excited State  21:  3.108-A      5.2511 eV  236.11 nm  f=0.0105
<S**2>=2.166
     39A -> 43A       -0.11605
     41A -> 43A       -0.12175
     31B -> 41B       -0.21056
     33B -> 41B        0.28110
```

```
     33B -> 42B         0.16946
     34B -> 41B        -0.37211
     34B -> 42B         0.16533
     35B -> 41B         0.26680
     35B -> 42B        -0.24548
     36B -> 41B         0.63468
     40B -> 43B        -0.10987

 Excited State  22:  3.108-A      5.3426 eV  232.07 nm  f=0.0718
<S**2>=2.164
     41A -> 43A         0.18919
     42A -> 43A         0.10662
     42A -> 45A         0.10663
     31B -> 41B        -0.28405
     33B -> 41B         0.57855
     33B -> 42B         0.19219
     34B -> 42B         0.21206
     35B -> 41B        -0.17026
     35B -> 42B        -0.12228
     36B -> 41B        -0.46862
     38B -> 42B        -0.13867
     40B -> 43B         0.20826

 Excited State  23:  3.839-A      5.3907 eV  230.00 nm  f=0.0018
<S**2>=3.434
     38A -> 43A         0.43843
     41A -> 45A         0.29562
     41A -> 46A        -0.16030
     42A -> 45A         0.24412
     42A -> 47A         0.10891
     33B -> 42B        -0.15871
     34B -> 42B        -0.22830
     35B -> 42B         0.28625
     38B -> 43B        -0.39457
     40B -> 43B         0.12706
     40B -> 46B         0.25795
     40B -> 47B         0.30101

 Excited State  24:  3.123-A      5.4528 eV  227.38 nm  f=0.0088
<S**2>=2.189
     42A -> 44A        -0.15394
     42A -> 45A         0.47876
     42A -> 46A         0.12723
     42A -> 47A         0.59251
     42A -> 48A        -0.18356
     33B -> 41B        -0.27211
     34B -> 41B        -0.26826
     35B -> 41B        -0.25620
     36B -> 41B         0.10292

 Excited State  25:  3.107-A      5.4757 eV  226.43 nm  f=0.0053
<S**2>=2.163
     42A -> 46A         0.16204
     42A -> 47A         0.38395
     42A -> 48A        -0.12819
     33B -> 41B         0.21376
     34B -> 41B         0.49847
```

```
     34B -> 42B       -0.12619
     35B -> 41B        0.63789
 SavETr:  write IOETrn=   770 NScale= 10 NData=  16 NLR=1 NState=   25
LETran=     460.
****************************************************************************
```

15. TD-DFT calculation of nitrosoalkene $^{3}3B$

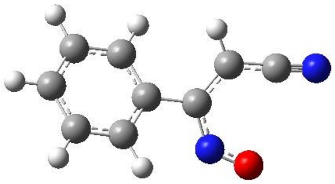

```
Excitation energies and oscillator strengths:
Excited State 1: 3.019-A 2.0298 eV 610.83 nm f=0.0001 <S**2>=2.029
38B -> 41B 0.24938
40B -> 41B 0.96210
This state for optimization and/or second-order correction.
Total Energy, E(TD-HF/TD-DFT) = -531.111904242
Copying the excited state density for this state as the 1-particle RhoCI
density.

Excited State 2: 3.080-A 2.4305 eV 510.12 nm f=0.0292 <S**2>=2.122
42A -> 43A 0.34949
42A -> 46A -0.11707
38B -> 42B 0.16788
40B -> 42B 0.90421
Excited State 3: 3.049-A 3.0271 eV 409.58 nm f=0.0026 <S**2>=2.074
39B -> 41B 0.99496

Excited State 4: 3.129-A 3.3237 eV 373.03 nm f=0.0012 <S**2>=2.197
39B -> 42B 0.98331

Excited State 5: 3.729-A 3.4736 eV 356.93 nm f=0.0029 <S**2>=3.227
40A -> 44A 0.25331
41A -> 43A 0.46933
41A -> 46A 0.13515
42A -> 43A -0.44128
38B -> 41B -0.26390
38B -> 42B -0.28318
39B -> 42B 0.12626
39B -> 44B -0.25603
40B -> 42B 0.15301
40B -> 43B 0.43953

Excited State 6: 3.098-A 3.5915 eV 345.21 nm f=0.0003 <S**2>=2.150
41A -> 43A 0.13176
42A -> 43A -0.13117
32B -> 41B 0.15408
38B -> 41B 0.89297
38B -> 42B -0.10617
40B -> 41B -0.25646
40B -> 43B 0.12164
```

Excited State 7: 3.384-A 3.7457 eV 331.00 nm f=0.0191 <S**2>=2.614
40A -> 44A 0.21615
41A -> 43A 0.34302
42A -> 43A 0.57396
37B -> 41B 0.16632
38B -> 42B 0.40856
39B -> 44B -0.20494
40B -> 42B -0.36378
40B -> 43B 0.31196

Excited State 8: 3.085-A 4.1854 eV 296.23 nm f=0.0165 <S**2>=2.129
40A -> 43A 0.10617
42A -> 43A -0.37108
31B -> 41B 0.10176
37B -> 41B -0.56742
38B -> 42B 0.68985

Excited State 9: 3.140-A 4.2323 eV 292.95 nm f=0.0001 <S**2>=2.214
37B -> 41B 0.11098
37B -> 42B 0.96811

Excited State 10: 3.960-A 4.3497 eV 285.04 nm f=0.0013 <S**2>=3.671
40A -> 43A 0.76839
40A -> 46A 0.12359
41A -> 44A 0.16974
37B -> 41B 0.12515
39B -> 43B 0.40709
40B -> 44B -0.37014

Excited State 11: 4.086-A 4.4570 eV 278.18 nm f=0.0014 <S**2>=3.924
40A -> 44A -0.57759
41A -> 43A 0.37890
42A -> 44A 0.10158
37B -> 41B 0.11200
39B -> 44B 0.58764
40B -> 43B 0.33615

Excited State 12: 3.153-A 4.6258 eV 268.03 nm f=0.0789 <S**2>=2.236
41A -> 43A -0.10348
42A -> 43A -0.40620
42A -> 46A -0.20761
37B -> 41B 0.72659
38B -> 42B 0.33822
Excited State 13: 3.785-A 4.7902 eV 258.83 nm f=0.0035 <S**2>=3.332
39A -> 43A -0.17448
40A -> 43A 0.34790
41A -> 44A -0.50205
42A -> 44A 0.48697
38B -> 44B -0.10727
39B -> 43B 0.13338
40B -> 44B 0.54879

Excited State 14: 3.322-A 4.8739 eV 254.39 nm f=0.0023 <S**2>=2.510
39A -> 43A 0.10917
40A -> 43A -0.10286
41A -> 43A -0.12816

41A -> 44A 0.21799
42A -> 44A 0.78879
31B -> 41B -0.14633
34B -> 42B -0.12549
35B -> 42B 0.11203
39B -> 43B -0.13379
40B -> 43B 0.11457
40B -> 44B -0.35612

Excited State 15: 3.192-A 4.9036 eV 252.84 nm f=0.0005 <S**2>=2.297
39A -> 43A 0.85634
39A -> 46A -0.15223
41A -> 44A -0.11226
31B -> 41B -0.11265
34B -> 41B 0.13239
34B -> 42B -0.12920
35B -> 41B -0.13429
35B -> 42B 0.12661
39B -> 43B 0.18421
40B -> 44B 0.19635

Excited State 16: 3.193-A 4.9187 eV 252.07 nm f=0.0095 <S**2>=2.299
38A -> 43A 0.12916
39A -> 43A 0.22315
41A -> 44A 0.10062
42A -> 44A 0.27110
31B -> 41B 0.39099
32B -> 41B 0.14295
32B -> 42B 0.13480
33B -> 41B -0.22438
34B -> 41B 0.24654
34B -> 42B 0.39982
35B -> 41B -0.11624
35B -> 42B -0.37456
36B -> 41B -0.21944
38B -> 43B -0.15398
40B -> 47B -0.11277

Excited State 17: 3.064-A 5.0493 eV 245.54 nm f=0.0091 <S**2>=2.096
39A -> 43A -0.27630
27B -> 41B -0.10981
28B -> 41B -0.17319
31B -> 41B -0.12713
32B -> 41B 0.20701
34B -> 41B 0.57968
35B -> 41B -0.57105
36B -> 41B 0.11600
36B -> 42B 0.12612
38B -> 41B -0.18099
39B -> 43B -0.10236
40B -> 43B 0.10876

Excited State 18: 3.154-A 5.0759 eV 244.26 nm f=0.0200 <S**2>=2.237
39A -> 43A -0.15634
40A -> 43A -0.30159
41A -> 43A -0.17925
41A -> 44A 0.46122

39B -> 43B 0.66988
40B -> 43B 0.18313
40B -> 44B 0.33659

Excited State 19: 3.165-A 5.0901 eV 243.58 nm f=0.0023 <S**2>=2.254
33B -> 42B -0.13156
34B -> 42B 0.13779
35B -> 41B 0.10982
35B -> 42B 0.10054
36B -> 41B 0.12683
36B -> 42B 0.92957

Excited State 20: 3.114-A 5.1314 eV 241.62 nm f=0.2559 <S**2>=2.175
40A -> 43A 0.10053
40A -> 44A -0.13361
41A -> 43A -0.55938
41A -> 44A -0.14683
41A -> 46A 0.10494
42A -> 44A -0.11734
34B -> 41B -0.13331
34B -> 42B 0.23478
35B -> 42B -0.23480
39B -> 43B -0.16835
40B -> 43B 0.61398

Excited State 21: 3.090-A 5.2608 eV 235.67 nm f=0.0390 <S**2>=2.137
41A -> 43A 0.12000
31B -> 41B -0.10030
33B -> 41B 0.11965
34B -> 41B 0.11773
34B -> 42B 0.16848
35B -> 41B 0.13868
35B -> 42B -0.22839
36B -> 41B 0.85993
36B -> 42B -0.11874
38B -> 42B 0.10661
40B -> 43B -0.14661

Excited State 22: 3.122-A 5.2997 eV 233.94 nm f=0.0035 <S**2>=2.186
42A -> 44A -0.15719
42A -> 45A 0.68221
42A -> 46A -0.22369
42A -> 47A 0.49025
42A -> 48A -0.12502
42A -> 49A -0.32990

Excited State 23: 3.883-A 5.3749 eV 230.67 nm f=0.0107 <S**2>=3.519
38A -> 43A 0.45042
41A -> 43A -0.14199
41A -> 46A 0.34631
42A -> 46A -0.13262
42A -> 47A -0.10463
34B -> 42B -0.26526
35B -> 42B 0.27388
38B -> 43B -0.42167
40B -> 46B 0.16178
40B -> 47B -0.38383

```
Excited State 24: 3.132-A 5.4521 eV 227.41 nm f=0.0548 <S**2>=2.203
 38A -> 43A 0.19815
 28B -> 42B 0.10346
 31B -> 41B -0.37946
 33B -> 41B 0.64508
 34B -> 41B -0.12561
 34B -> 42B 0.12511
 35B -> 41B -0.17162
 35B -> 42B -0.22640
 36B -> 41B -0.30977
 38B -> 42B 0.16534
 40B -> 43B -0.16069

Excited State 25: 3.051-A 5.5185 eV 224.67 nm f=0.0254 <S**2>=2.078
42A -> 46A 0.11048
33B -> 41B 0.21935
34B -> 41B 0.58126
35B -> 41B 0.70526
36B -> 41B -0.19447
SavETr: write IOETrn= 770 NScale= 10 NData= 16 NLR=1 NState= 25 LETran= 460.
```

****

16. TD-DFT calculation of nitrosoalkene 3A in gas phase

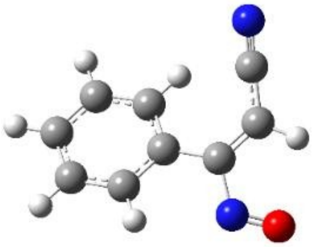

```
Excited State 1: Singlet-A 1.3200 eV 939.26 nm f=0.0003 <S**2>=0.000
40 -> 42 -0.17073
41 -> 42 0.67877
41 -> 43 0.11263
This state for optimization and/or second-order correction.
Total Energy, E(TD-HF/TD-DFT) = -531.139692730
Copying the excited state density for this state as the 1-particle RhoCI
density.

Excited State 2: Singlet-A  2.6578 eV  466.50 nm  f=0.0647 <S**2>=0.000
40 -> 42 0.67206
41 -> 42 0.17973

Excited State 3: Singlet-A  2.8291 eV  438.25 nm  f=0.0077 <S**2>=0.000
39 -> 42 0.69926

Excited State 4: Singlet-A  4.0461 eV  306.43 nm  f=0.0023 <S**2>=0.000
40 -> 43 -0.19003
41 -> 42 -0.10434
41 -> 43 0.65247
```

Excited State 5: Singlet-A 4.2816 eV 289.57 nm f=0.0632 <S**2>=0.000
37 -> 42 0.35939
38 -> 42 0.58081

Excited State 6: Singlet-A 4.5536 eV 272.28 nm f=0.0781 <S**2>=0.000
37 -> 42 0.58069
38 -> 42 -0.30830
40 -> 43 -0.14618
41 -> 43 -0.13465

Excited State 7: Singlet-A 4.8206 eV 257.20 nm f=0.2486 <S**2>=0.000
37 -> 42 0.10272
38 -> 42 -0.15754
39 -> 43 -0.14541
40 -> 43 0.62391
41 -> 43 0.16248

Excited State 8: Singlet-A 4.8954 eV 253.26 nm f=0.0254 <S**2>=0.000
39 -> 43 0.59919
40 -> 43 0.12492
40 -> 44 -0.30778
41 -> 44 -0.13408

Excited State 9: Singlet-A 5.3416 eV 232.11 nm f=0.0004 <S**2>=0.000
36 -> 42 0.69822

Excited State 10: Singlet-A 5.4725 eV 226.56 nm f=0.0040 <S**2>=0.000
40 -> 44 -0.20865
41 -> 44 0.66187

Excited State 11: Singlet-A 5.6330 eV 220.10 nm f=0.0104 <S**2>=0.000
34 -> 42 -0.14224
35 -> 42 0.66074

Excited State 12: Singlet-A 5.7057 eV 217.30 nm f=0.0559 <S**2>=0.000
34 -> 42 -0.14200
39 -> 43 0.28050
39 -> 45 0.24803
40 -> 44 0.50198
41 -> 44 0.10786
41 -> 45 -0.20475

Excited State 13: Singlet-A 5.7776 eV 214.60 nm f=0.0066 <S**2>=0.000
33 -> 42 0.59381
34 -> 42 0.35663

Excited State 14: Singlet-A 5.8562 eV 211.71 nm f=0.0177 <S**2>=0.000
33 -> 42 -0.33620
34 -> 42 0.54564
35 -> 42 0.18303

Excited State 15: Singlet-A 5.9116 eV 209.73 nm f=0.0186 <S**2>=0.000
39 -> 45 0.10211
40 -> 44 0.11212
40 -> 45 -0.25041
41 -> 45 0.57794
41 -> 46 0.15683

Excited State 16: Singlet-A 6.0320 eV 205.54 nm f=0.0068 <S**2>=0.000
39 -> 44 -0.14546
39 -> 45 0.10635
40 -> 45 0.46972
40 -> 46 -0.41231
41 -> 45 0.16130

Excited State 17: Singlet-A 6.1417 eV 201.87 nm f=0.0071 <S**2>=0.000
32 -> 42 -0.18705
38 -> 43 -0.27841
39 -> 44 -0.14044
40 -> 45 0.25846
40 -> 46 0.27395
41 -> 46 0.43312

Excited State 18: Singlet-A 6.2108 eV 199.63 nm f=0.0331 <S**2>=0.000
31 -> 42 0.16882
32 -> 42 0.16910
38 -> 43 0.34548
39 -> 44 -0.17764
40 -> 46 -0.13789
41 -> 45 -0.20967
41 -> 46 0.43496

Excited State 19: Singlet-A 6.2229 eV 199.24 nm f=0.0529 <S**2>=0.000
31 -> 42 0.15973
32 -> 42 0.40511
39 -> 44 -0.28385
40 -> 45 0.15526
40 -> 46 0.32620
41 -> 46 -0.21639

Excited State 20: Singlet-A 6.3645 eV 194.81 nm f=0.0216 <S**2>=0.000
32 -> 42 0.19668
38 -> 43 -0.25211
39 -> 44 0.13724
39 -> 45 0.11865
40 -> 47 0.40465
41 -> 47 0.39764

Excited State 21: Singlet-A 6.4039 eV 193.61 nm f=0.1347 <S**2>=0.000
31 -> 42 0.19714
32 -> 42 0.26127
38 -> 43 -0.26187
39 -> 44 0.36759
40 -> 46 -0.12988
40 -> 47 -0.28301
41 -> 46 0.13840
41 -> 47 -0.17363

Excited State 22: Singlet-A 6.4609 eV 191.90 nm f=0.0121 <S**2>=0.000
40 -> 47 -0.42686
40 -> 48 -0.10019
41 -> 47 0.47315
41 -> 48 0.15804
41 -> 49 -0.14804

Excited State 23: Singlet-A 6.6130 eV 187.49 nm f=0.0322 <S**2>=0.000
31 -> 42 0.35372
32 -> 42 -0.26019
37 -> 43 0.39035
39 -> 45 0.23148
39 -> 47 -0.13924
40 -> 46 0.12168

Excited State 24: Singlet-A 6.6513 eV 186.41 nm f=0.0620 <S**2>=0.000
31 -> 42 -0.24112
32 -> 42 0.16359
38 -> 44 -0.12367
39 -> 45 0.40328
39 -> 46 -0.31279
39 -> 47 -0.23378
40 -> 44 -0.11859
40 -> 48 0.11338
Excited State 25: Singlet-A 6.6820 eV 185.55 nm f=0.0065 <S**2>=0.000
31 -> 42 -0.31440
32 -> 42 0.17383
37 -> 43 0.53124
39 -> 45 -0.12359
39 -> 46 0.15807
****

17. TD-DFT calculation of nitrosoalkene 3A in IEFPCM

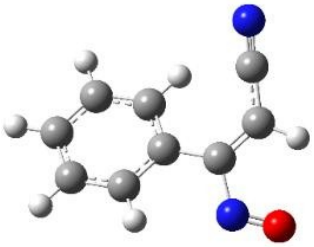

Excitation energies and oscillator strengths:
Excited State 1: Singlet-A 1.3544 eV 915.42 nm f=0.0004 <S**2>=0.000
40 -> 42 0.56485
41 -> 42 0.40681
This state for optimization and/or second-order correction.
Total Energy, E(TD-HF/TD-DFT) = -531.145270237
Copying the excited state density for this state as the 1-particle RhoCI density.

Excited State 2: Singlet-A 2.5191 eV 492.18 nm f=0.0818 <S**2>=0.000
40 -> 42 -0.39914
41 -> 42 0.57479

Excited State 3: Singlet-A 2.7052 eV 458.32 nm f=0.0088 <S**2>=0.000
39 -> 42 0.69716
40 -> 42 -0.10982

Excited State 4: Singlet-A 4.1327 eV 300.00 nm f=0.0020 <S**2>=0.000

38 -> 42 -0.11752
40 -> 43 0.56154
41 -> 43 0.37466

Excited State 5: Singlet-A 4.2264 eV 293.36 nm f=0.1154 <S**2>=0.000
37 -> 42 0.24856
38 -> 42 0.62694
41 -> 43 0.14084

Excited State 6: Singlet-A 4.5537 eV 272.27 nm f=0.0895 <S**2>=0.000
37 -> 42 0.61901
38 -> 42 -0.18022
41 -> 43 -0.23952

Excited State 7: Singlet-A 4.7163 eV 262.89 nm f=0.2977 <S**2>=0.000
37 -> 42 0.17235
38 -> 42 -0.18326
39 -> 43 -0.10244
40 -> 43 -0.38250
41 -> 43 0.50992

Excited State 8: Singlet-A 4.8413 eV 256.10 nm f=0.0297 <S**2>=0.000
39 -> 43 0.62703
40 -> 43 -0.10020
40 -> 44 0.13506
41 -> 44 -0.26195

Excited State 9: Singlet-A 5.1903 eV 238.87 nm f=0.0009 <S**2>=0.000
36 -> 42 0.69683

Excited State 10: Singlet-A 5.5341 eV 224.04 nm f=0.0108 <S**2>=0.000
34 -> 42 -0.10791
35 -> 42 0.67488
40 -> 44 0.10588

Excited State 11: Singlet-A 5.6208 eV 220.58 nm f=0.0275 <S**2>=0.000
39 -> 43 0.16990
39 -> 45 0.14852
40 -> 44 0.31079
41 -> 44 0.56094

Excited State 12: Singlet-A 5.6657 eV 218.83 nm f=0.0646 <S**2>=0.000
39 -> 43 -0.19151
39 -> 45 -0.17048
40 -> 44 0.58099
41 -> 44 -0.18029
41 -> 45 0.14436

Excited State 13: Singlet-A 5.7916 eV 214.08 nm f=0.0289 <S**2>=0.000
34 -> 42 0.66399
35 -> 42 0.10600
39 -> 44 -0.10253

Excited State 14: Singlet-A 5.9443 eV 208.58 nm f=0.0049 <S**2>=0.000
39 -> 45 0.12082
39 -> 46 -0.10218
40 -> 45 -0.32488

40 -> 46 -0.21531
41 -> 45 0.34195
41 -> 46 0.40055

Excited State 15: Singlet-A 5.9968 eV 206.75 nm f=0.0068 <S**2>=0.000
33 -> 42 0.67119

Excited State 16: Singlet-A 6.0330 eV 205.51 nm f=0.0149 <S**2>=0.000
32 -> 42 0.11654
33 -> 42 -0.14438
38 -> 43 0.10120
39 -> 45 0.10747
40 -> 45 0.49021
40 -> 46 -0.22590
41 -> 45 0.33278

Excited State 17: Singlet-A 6.1192 eV 202.62 nm f=0.0080 <S**2>=0.000
32 -> 42 0.11884
38 -> 43 0.25169
39 -> 44 0.27547
40 -> 45 0.20522
41 -> 45 -0.30305
41 -> 46 0.43237

Excited State 18: Singlet-A 6.1978 eV 200.05 nm f=0.0878 <S**2>=0.000
31 -> 42 0.21006
32 -> 42 0.36941
38 -> 43 0.30997
39 -> 44 -0.33255
40 -> 45 -0.18599
41 -> 45 -0.12636
41 -> 46 -0.10448

Excited State 19: Singlet-A 6.2361 eV 198.82 nm f=0.0361 <S**2>=0.000
32 -> 42 0.23329
38 -> 43 -0.14642
40 -> 45 0.10293
40 -> 46 0.54875
41 -> 45 0.20918
41 -> 46 0.19734

Excited State 20: Singlet-A 6.3581 eV 195.00 nm f=0.1976 <S**2>=0.000
31 -> 42 0.22848
32 -> 42 0.34176
38 -> 43 -0.32295
39 -> 44 0.36552
40 -> 46 -0.12773
41 -> 46 -0.14533

Excited State 21: Singlet-A 6.4118 eV 193.37 nm f=0.0077 <S**2>=0.000
39 -> 45 0.18464
40 -> 47 -0.22004
41 -> 47 0.61009

Excited State 22: Singlet-A 6.5412 eV 189.54 nm f=0.0401 <S**2>=0.000
31 -> 42 -0.13957
32 -> 42 0.18701

37 -> 43 -0.11072
38 -> 43 -0.20176
39 -> 45 0.20420
39 -> 46 0.41426
40 -> 46 -0.14456
40 -> 47 -0.25352
41 -> 45 -0.11335
41 -> 47 -0.20044

Excited State 23: Singlet-A 6.5614 eV 188.96 nm f=0.0070 <S**2>=0.000
31 -> 42 -0.27805
32 -> 42 0.17908
39 -> 45 0.13121
39 -> 46 0.11481
40 -> 47 0.52344
40 -> 49 0.13377
41 -> 47 0.11550
Excited State 24: Singlet-A 6.5768 eV 188.52 nm f=0.0265 <S**2>=0.000
31 -> 42 0.38128
32 -> 42 -0.21202
37 -> 43 0.14647
39 -> 45 0.38026
39 -> 46 0.22285
40 -> 47 0.17424

Excited State 25: Singlet-A 6.6691 eV 185.91 nm f=0.1300 <S**2>=0.000
31 -> 42 -0.23997
32 -> 42 0.11294
37 -> 43 0.33539
38 -> 44 -0.13727
39 -> 45 0.28827
39 -> 46 -0.25794
39 -> 47 -0.17790
40 -> 47 -0.10543
41 -> 44 -0.14086
41 -> 46 -0.11810
41 -> 47 -0.14578

18. TD-DFT calculation of nitrosoalkene 3A in SMD

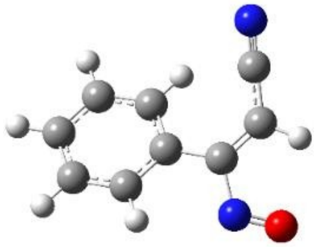

Excitation energies and oscillator strengths:
Excited State 1: Singlet-A 1.3568 eV 913.83 nm f=0.0004 <S**2>=0.000
40 -> 42 0.60926
41 -> 42 0.33409

This state for optimization and/or second-order correction.
Total Energy, E(TD-HF/TD-DFT) = -531.150840774

Copying the excited state density for this state as the 1-particle RhoCI density.

Excited State 2: Singlet-A 2.4849 eV 498.96 nm f=0.0834 <S**2>=0.000
40 -> 42 -0.32599
41 -> 42 0.62012

Excited State 3: Singlet-A 2.6831 eV 462.09 nm f=0.0089 <S**2>=0.000
39 -> 42 0.69617
40 -> 42 -0.11642

Excited State 4: Singlet-A 4.1493 eV 298.81 nm f=0.0019 <S**2>=0.000
38 -> 42 -0.14818
40 -> 43 0.60080
41 -> 43 0.29745

Excited State 5: Singlet-A 4.2151 eV 294.15 nm f=0.1147 <S**2>=0.000
37 -> 42 0.24840
38 -> 42 0.62169
41 -> 43 0.15783

Excited State 6: Singlet-A 4.5487 eV 272.57 nm f=0.0984 <S**2>=0.000
37 -> 42 0.61063
38 -> 42 -0.16892
41 -> 43 -0.26803

Excited State 7: Singlet-A 4.6887 eV 264.43 nm f=0.2970 <S**2>=0.000
37 -> 42 0.19687
38 -> 42 -0.19057
39 -> 43 -0.10497
40 -> 43 -0.31770
41 -> 43 0.54224

Excited State 8: Singlet-A 4.8278 eV 256.81 nm f=0.0330 <S**2>=0.000
39 -> 43 0.62916
40 -> 43 -0.10481
40 -> 44 0.10033
41 -> 44 -0.26872

Excited State 9: Singlet-A 5.1249 eV 241.92 nm f=0.0012 <S**2>=0.000
36 -> 42 0.69507

Excited State 10: Singlet-A 5.4979 eV 225.51 nm f=0.0119 <S**2>=0.000
35 -> 42 0.68155

Excited State 11: Singlet-A 5.6177 eV 220.70 nm f=0.0679 <S**2>=0.000
39 -> 43 0.23249
39 -> 45 0.19147
39 -> 46 -0.12341
41 -> 44 0.58260
41 -> 45 -0.10833

Excited State 12: Singlet-A 5.6715 eV 218.61 nm f=0.0299 <S**2>=0.000
40 -> 44 0.66555
41 -> 45 0.11398

Excited State 13: Singlet-A 5.7800 eV 214.50 nm f=0.0298 <S**2>=0.000

34 -> 42 0.66550
39 -> 44 -0.10357

Excited State 14: Singlet-A 5.9183 eV 209.49 nm f=0.0063 <S**2>=0.000
39 -> 45 0.11442
39 -> 46 -0.11487
40 -> 45 -0.26891
40 -> 46 -0.15089
41 -> 45 0.41244
41 -> 46 0.40050

Excited State 15: Singlet-A 6.0413 eV 205.23 nm f=0.0172 <S**2>=0.000
32 -> 42 0.17183
33 -> 42 0.24702
38 -> 43 0.15187
40 -> 45 0.48559
40 -> 46 -0.26496
41 -> 45 0.17268

Excited State 16: Singlet-A 6.0654 eV 204.41 nm f=0.0041 <S**2>=0.000
33 -> 42 0.58004
39 -> 44 0.10533
40 -> 45 -0.18541
41 -> 45 -0.24104
41 -> 46 0.17694

Excited State 17: Singlet-A 6.1050 eV 203.09 nm f=0.0080 <S**2>=0.000
33 -> 42 -0.25531
38 -> 43 0.18531
39 -> 44 0.31417
40 -> 45 0.20503
41 -> 45 -0.25461
41 -> 46 0.41754

Excited State 18: Singlet-A 6.1890 eV 200.33 nm f=0.0959 <S**2>=0.000
31 -> 42 0.20514
32 -> 42 0.35095
33 -> 42 -0.15200
38 -> 43 0.34166
39 -> 44 -0.30697
40 -> 45 -0.19338
41 -> 45 -0.14917

Excited State 19: Singlet-A 6.2281 eV 199.07 nm f=0.0369 <S**2>=0.000
32 -> 42 0.23702
38 -> 43 -0.11339
40 -> 45 0.15850
40 -> 46 0.56372
41 -> 45 0.18867
41 -> 46 0.12961

Excited State 20: Singlet-A 6.3383 eV 195.61 nm f=0.2081 <S**2>=0.000
31 -> 42 0.24068
32 -> 42 0.34395
38 -> 43 -0.29394
39 -> 44 0.36277
40 -> 46 -0.12585

41 -> 46 -0.14957

Excited State 21: Singlet-A 6.3611 eV 194.91 nm f=0.0020 <S**2>=0.000
38 -> 43 -0.14278
39 -> 45 0.15997
40 -> 47 -0.15771
41 -> 47 0.62628

Excited State 22: Singlet-A 6.5089 eV 190.49 nm f=0.0283 <S**2>=0.000
32 -> 42 -0.15013
38 -> 43 0.19718
39 -> 45 -0.19121
39 -> 46 -0.36115
40 -> 46 0.13379
40 -> 47 0.39094
40 -> 49 0.13204
41 -> 47 0.17952
Excited State 23: Singlet-A 6.5255 eV 190.00 nm f=0.0103 <S**2>=0.000
31 -> 42 -0.20977
32 -> 42 0.17244
39 -> 45 0.28540
39 -> 46 0.22557
40 -> 47 0.47454
40 -> 49 0.12702

Excited State 24: Singlet-A 6.5489 eV 189.32 nm f=0.0349 <S**2>=0.000
29 -> 42 -0.10105
31 -> 42 0.43156
32 -> 42 -0.25762
37 -> 43 0.16232
39 -> 45 0.35924
39 -> 46 0.13085

Excited State 25: Singlet-A 6.6365 eV 186.82 nm f=0.1543 <S**2>=0.000
31 -> 42 0.24240
32 -> 42 -0.11400
37 -> 43 -0.24519
38 -> 44 0.12817
39 -> 45 -0.27924
39 -> 46 0.31053
39 -> 47 0.21418
40 -> 47 0.13657
41 -> 44 0.15373
41 -> 46 0.13091
41 -> 47 0.13453

19. TD-DFT calculation of nitrosoalkene 3A in I-PCM

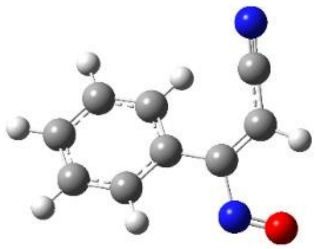

Excitation energies and oscillator strengths:

```
Excited State 1: Singlet-A 1.3200 eV 939.26 nm f=0.0003 <S**2>=0.000
40 -> 42 -0.17073
41 -> 42 0.67877
41 -> 43 0.11262
This state for optimization and/or second-order correction.
Total Energy, E(TD-HF/TD-DFT) = -531.139692729
Copying the excited state density for this state as the 1-particle RhoCI
density.

Excited State 2: Singlet-A 2.6578 eV 466.50 nm f=0.0647 <S**2>=0.000
40 -> 42 0.67205
41 -> 42 0.17973

Excited State 3: Singlet-A 2.8291 eV 438.25 nm f=0.0077 <S**2>=0.000
39 -> 42 0.69926

Excited State 4: Singlet-A 4.0461 eV 306.43 nm f=0.0023 <S**2>=0.000
40 -> 43 -0.19003
41 -> 42 -0.10434
41 -> 43 0.65247

Excited State 5: Singlet-A 4.2816 eV 289.57 nm f=0.0633 <S**2>=0.000
37 -> 42 0.35938
38 -> 42 0.58082

Excited State 6: Singlet-A 4.5536 eV 272.28 nm f=0.0781 <S**2>=0.000
37 -> 42 0.58069
38 -> 42 -0.30830
40 -> 43 -0.14618
41 -> 43 -0.13465

Excited State 7: Singlet-A 4.8206 eV 257.20 nm f=0.2486 <S**2>=0.000
37 -> 42 0.10272
38 -> 42 -0.15754
39 -> 43 -0.14539
40 -> 43 0.62391
41 -> 43 0.16248

Excited State 8: Singlet-A 4.8954 eV 253.26 nm f=0.0254 <S**2>=0.000
39 -> 43 0.59920
40 -> 43 0.12491
40 -> 44 -0.30778
41 -> 44 -0.13408

Excited State 9: Singlet-A 5.3416 eV 232.11 nm f=0.0004 <S**2>=0.000
36 -> 42 0.69822

Excited State 10: Singlet-A 5.4725 eV 226.56 nm f=0.0040 <S**2>=0.000
40 -> 44 -0.20865
41 -> 44 0.66187

Excited State 11: Singlet-A 5.6330 eV 220.10 nm f=0.0104 <S**2>=0.000
34 -> 42 -0.14224
35 -> 42 0.66074

Excited State 12: Singlet-A 5.7057 eV 217.30 nm f=0.0559 <S**2>=0.000
34 -> 42 -0.14200
```

39 -> 43 0.28050
39 -> 45 0.24803
40 -> 44 0.50198
41 -> 44 0.10786
41 -> 45 -0.20475

Excited State 13: Singlet-A 5.7776 eV 214.60 nm f=0.0066 <S**2>=0.000
33 -> 42 0.59380
34 -> 42 0.35665

Excited State 14: Singlet-A 5.8562 eV 211.71 nm f=0.0177 <S**2>=0.000
33 -> 42 -0.33622
34 -> 42 0.54563
35 -> 42 0.18303

Excited State 15: Singlet-A 5.9116 eV 209.73 nm f=0.0186 <S**2>=0.000
39 -> 45 0.10211
40 -> 44 0.11212
40 -> 45 -0.25041
41 -> 45 0.57794
41 -> 46 0.15683

Excited State 16: Singlet-A 6.0320 eV 205.54 nm f=0.0068 <S**2>=0.000
39 -> 44 -0.14547
39 -> 45 0.10634
40 -> 45 0.46972
40 -> 46 -0.41231
41 -> 45 0.16130

Excited State 17: Singlet-A 6.1417 eV 201.87 nm f=0.0071 <S**2>=0.000
32 -> 42 -0.18705
38 -> 43 -0.27841
39 -> 44 -0.14044
40 -> 45 0.25846
40 -> 46 0.27395
41 -> 46 0.43312

Excited State 18: Singlet-A 6.2108 eV 199.63 nm f=0.0331 <S**2>=0.000
31 -> 42 0.16884
32 -> 42 0.16917
38 -> 43 0.34548
39 -> 44 -0.17770
40 -> 46 -0.13782
41 -> 45 -0.20967
41 -> 46 0.43492

Excited State 19: Singlet-A 6.2229 eV 199.24 nm f=0.0529 <S**2>=0.000
31 -> 42 0.15969
32 -> 42 0.40507
39 -> 44 -0.28382
40 -> 45 0.15525
40 -> 46 0.32623
41 -> 46 -0.21648

Excited State 20: Singlet-A 6.3645 eV 194.81 nm f=0.0216 <S**2>=0.000
32 -> 42 0.19670
38 -> 43 -0.25212

```
39 -> 44 0.13726
39 -> 45 0.11865
40 -> 47 0.40463
41 -> 47 0.39762

Excited State 21: Singlet-A 6.4039 eV 193.61 nm f=0.1347 <S**2>=0.000
31 -> 42 0.19714
32 -> 42 0.26127
38 -> 43 -0.26185
39 -> 44 0.36758
40 -> 46 -0.12988
40 -> 47 -0.28303
41 -> 46 0.13840
41 -> 47 -0.17366

Excited State 22: Singlet-A 6.4609 eV 191.90 nm f=0.0121 <S**2>=0.000
40 -> 47 -0.42686
40 -> 48 -0.10020
41 -> 47 0.47315
41 -> 48 0.15804
41 -> 49 -0.14804

Excited State 23: Singlet-A 6.6130 eV 187.49 nm f=0.0322 <S**2>=0.000
31 -> 42 0.35376
32 -> 42 -0.26021
37 -> 43 0.39033
39 -> 45 0.23145
39 -> 47 -0.13920
40 -> 46 0.12167

Excited State 24: Singlet-A 6.6513 eV 186.41 nm f=0.0620 <S**2>=0.000
31 -> 42 -0.24113
32 -> 42 0.16359
38 -> 44 -0.12368
39 -> 45 0.40329
39 -> 46 -0.31274
39 -> 47 -0.23380
40 -> 44 -0.11860
40 -> 48 0.11338

Excited State 25: Singlet-A 6.6820 eV 185.55 nm f=0.0065 <S**2>=0.000
31 -> 42 -0.31437
32 -> 42 0.17381
37 -> 43 0.53122
39 -> 45 -0.12364
39 -> 46 0.15814
****************************************************************
```

20. TD-DFT calculation of nitrosoalkene 3A in C-PCM

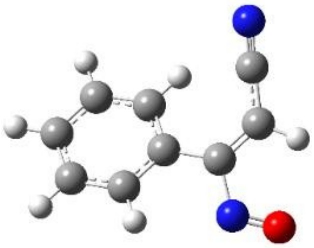

```
Excitation energies and oscillator strengths:
Excited State 1: Singlet-A 1.3564 eV 914.07 nm f=0.0005 <S**2>=0.000
40 -> 42 0.56916
41 -> 42 0.40062
This state for optimization and/or second-order correction.
Total Energy, E(TD-HF/TD-DFT) = -531.145676119
Copying the excited state density for this state as the 1-particle
RhoCI density.

Excited State 2: Singlet-A 2.5163 eV 492.73 nm f=0.0846 <S**2>=0.000
40 -> 42 -0.39385
41 -> 42 0.57919

Excited State 3: Singlet-A 2.7112 eV 457.31 nm f=0.0088 <S**2>=0.000
39 -> 42 0.69771
40 -> 42 -0.10647

Excited State 4: Singlet-A 4.1354 eV 299.81 nm f=0.0021 <S**2>=0.000
38 -> 42 -0.12042
40 -> 43 0.56534
41 -> 43 0.36832

Excited State 5: Singlet-A 4.2223 eV 293.64 nm f=0.1228 <S**2>=0.000
37 -> 42 0.24020
38 -> 42 0.63003
41 -> 43 0.14322

Excited State 6: Singlet-A 4.5534 eV 272.29 nm f=0.0918 <S**2>=0.000
37 -> 42 0.62090
38 -> 42 -0.17170
41 -> 43 -0.24191

Excited State 7: Singlet-A 4.7095 eV 263.26 nm f=0.3053 <S**2>=0.000
37 -> 42 0.17774
38 -> 42 -0.18229
40 -> 43 -0.37874
41 -> 43 0.51342

Excited State 8: Singlet-A 4.8421 eV 256.06 nm f=0.0305 <S**2>=0.000
39 -> 43 0.62864
40 -> 44 0.13169
41 -> 44 -0.26266

Excited State 9: Singlet-A 5.1918 eV 238.81 nm f=0.0009 <S**2>=0.000
36 -> 42 0.69680

Excited State 10: Singlet-A 5.5359 eV 223.96 nm f=0.0113 <S**2>=0.000
34 -> 42 -0.10490
35 -> 42 0.67472
40 -> 44 0.10900

Excited State 11: Singlet-A 5.6184 eV 220.67 nm f=0.0345 <S**2>=0.000
```

39 -> 43 0.18107
39 -> 45 0.15485
40 -> 44 0.28490
41 -> 44 0.56955

Excited State 12: Singlet-A 5.6616 eV 218.99 nm f=0.0667 <S**2>=0.000
35 -> 42 -0.10500
39 -> 43 -0.18371
39 -> 45 -0.15956
40 -> 44 0.59610
41 -> 44 -0.15910
41 -> 45 0.13643

Excited State 13: Singlet-A 5.7951 eV 213.95 nm f=0.0299 <S**2>=0.000
34 -> 42 0.66453
35 -> 42 0.10390
39 -> 44 -0.10498

Excited State 14: Singlet-A 5.9444 eV 208.57 nm f=0.0052 <S**2>=0.000
39 -> 45 0.12020
40 -> 45 -0.31801
40 -> 46 -0.21746
41 -> 45 0.34172
41 -> 46 0.40556

Excited State 15: Singlet-A 6.0056 eV 206.45 nm f=0.0080 <S**2>=0.000
32 -> 42 0.10581
33 -> 42 0.65776
40 -> 45 0.13056
40 -> 46 -0.10272

Excited State 16: Singlet-A 6.0341 eV 205.47 nm f=0.0148 <S**2>=0.000
32 -> 42 0.10630
33 -> 42 -0.19102
39 -> 45 0.10170
40 -> 45 0.48660
40 -> 46 -0.21624
41 -> 45 0.33103

Excited State 17: Singlet-A 6.1172 eV 202.68 nm f=0.0079 <S**2>=0.000
32 -> 42 0.11134
38 -> 43 0.24722
39 -> 44 0.28358
40 -> 45 0.20714
41 -> 45 -0.30706
41 -> 46 0.42619

Excited State 18: Singlet-A 6.1954 eV 200.12 nm f=0.0970 <S**2>=0.000
31 -> 42 0.20476
32 -> 42 0.37313
38 -> 43 0.31387
39 -> 44 -0.33913

40 -> 45 -0.17804
41 -> 45 -0.12392

Excited State 19: Singlet-A 6.2376 eV 198.77 nm f=0.0368 <S**2>=0.000
32 -> 42 0.22191
38 -> 43 -0.15081
40 -> 45 0.10976
40 -> 46 0.55211
41 -> 45 0.20784
41 -> 46 0.20514

Excited State 20: Singlet-A 6.3535 eV 195.14 nm f=0.2011 <S**2>=0.000
31 -> 42 0.22982
32 -> 42 0.35079
38 -> 43 -0.31154
39 -> 44 0.36340
40 -> 46 -0.12819
41 -> 46 -0.15065

Excited State 21: Singlet-A 6.4112 eV 193.39 nm f=0.0069 <S**2>=0.000
38 -> 43 -0.10576
39 -> 45 0.18325
40 -> 47 -0.21622
41 -> 47 0.61216

Excited State 22: Singlet-A 6.5415 eV 189.53 nm f=0.0492 <S**2>=0.000
31 -> 42 -0.17289
32 -> 42 0.20505
37 -> 43 -0.12489
38 -> 43 -0.20778
39 -> 45 0.17022
39 -> 46 0.39506
40 -> 46 -0.14877
40 -> 47 -0.25587
41 -> 45 -0.11820
41 -> 47 -0.19344

Excited State 23: Singlet-A 6.5625 eV 188.93 nm f=0.0081 <S**2>=0.000
31 -> 42 -0.30123
32 -> 42 0.18745
39 -> 45 0.10524
40 -> 47 0.51628
40 -> 49 0.13721
41 -> 47 0.11790

Excited State 24: Singlet-A 6.5760 eV 188.54 nm f=0.0251 <S**2>=0.000
31 -> 42 0.34368
32 -> 42 -0.17976
37 -> 43 0.13028
39 -> 45 0.41682
39 -> 46 0.24815
40 -> 47 0.18387

```
Excited State 25: Singlet-A 6.6616 eV 186.12 nm f=0.1677 <S**2>=0.000
31 -> 42 -0.24228
32 -> 42 0.11238
37 -> 43 0.26623
38 -> 44 -0.14724
39 -> 45 0.30408
39 -> 46 -0.29892
39 -> 47 -0.15087
40 -> 47 -0.12007
41 -> 44 -0.15346
41 -> 46 -0.12391
41 -> 47 -0.16038

**********************************************************************
```

21. TD-DFT calculation of nitrosoalkene 3B in gas phase

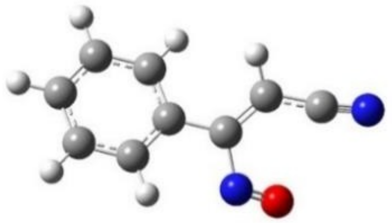

```
Excitation energies and oscillator strengths:
Excited State 1: Singlet-A 1.3182 eV 940.58 nm f=0.0006 <S**2>=0.000
41 -> 42 0.69469
41 -> 43 -0.11985
This state for optimization and/or second-order correction.
Total Energy, E(TD-HF/TD-KS) = -531.143521437
Copying the excited state density for this state as the 1-particle
RhoCI density.

Excited State 2: Singlet-A 2.9114 eV 425.85 nm f=0.1040 <S**2>=0.000
40 -> 42 0.68894
40 -> 43 -0.10885

Excited State 3: Singlet-A 3.1516 eV 393.40 nm f=0.0036 <S**2>=0.000
39 -> 42 0.70415

Excited State 4: Singlet-A 3.8616 eV 321.07 nm f=0.0073 <S**2>=0.000
37 -> 42 0.10541
40 -> 43 -0.11028
41 -> 42 0.10983
41 -> 43 0.66246

Excited State 5: Singlet-A 4.3131 eV 287.46 nm f=0.0220 <S**2>=0.000
37 -> 42 -0.38019
38 -> 42 0.57960

Excited State 6: Singlet-A 4.6589 eV 266.12 nm f=0.0517 <S**2>=0.000
```

37 -> 42 0.54683
38 -> 42 0.31642
40 -> 43 -0.22601
41 -> 43 -0.15663

Excited State 7: Singlet-A 4.9072 eV 252.66 nm f=0.3862 <S**2>=0.000
37 -> 42 0.18161
38 -> 42 0.16011
39 -> 43 -0.11846
40 -> 42 0.10847
40 -> 43 0.61402
40 -> 44 0.11319

Excited State 8: Singlet-A 4.9373 eV 251.12 nm f=0.0480 <S**2>=0.000
39 -> 43 0.55599
40 -> 43 0.16094
40 -> 44 -0.30575
41 -> 44 -0.24216

Excited State 9: Singlet-A 5.2036 eV 238.27 nm f=0.0016 <S**2>=0.000
39 -> 43 0.16791
40 -> 44 -0.20918
41 -> 44 0.64962

Excited State 10: Singlet-A 5.4801 eV 226.24 nm f=0.0051 <S**2>=0.000
35 -> 42 0.62270
36 -> 42 -0.31689

Excited State 11: Singlet-A 5.5666 eV 222.73 nm f=0.0008 <S**2>=0.000
35 -> 42 0.32216
36 -> 42 0.62297

Excited State 12: Singlet-A 5.7612 eV 215.20 nm f=0.0301 <S**2>=0.000
39 -> 43 0.17413
40 -> 44 0.24502
41 -> 45 0.21146
41 -> 46 0.56122

Excited State 13: Singlet-A 5.8003 eV 213.75 nm f=0.0561 <S**2>=0.000
33 -> 42 0.27476
39 -> 43 0.26670
39 -> 46 0.17855
40 -> 44 0.42977
41 -> 46 -0.32244

Excited State 14: Singlet-A 5.8281 eV 212.73 nm f=0.0095 <S**2>=0.000
33 -> 42 0.30742
34 -> 42 0.59393
40 -> 44 -0.11191
Excited State 15: Singlet-A 5.9045 eV 209.98 nm f=0.0126 <S**2>=0.000
33 -> 42 0.11379
34 -> 42 -0.14793

40 -> 44 -0.10071
41 -> 45 0.60682
41 -> 46 -0.15748
41 -> 47 0.15506

Excited State 16: Singlet-A 5.9461 eV 208.51 nm f=0.0364 <S**2>=0.000
33 -> 42 0.51699
34 -> 42 -0.31186
39 -> 43 -0.10664
40 -> 44 -0.14972
41 -> 45 -0.17849
41 -> 46 0.13861

Excited State 17: Singlet-A 6.1308 eV 202.23 nm f=0.0023 <S**2>=0.000
39 -> 44 -0.10786
40 -> 45 0.64728
40 -> 47 0.13684
41 -> 47 -0.10723

Excited State 18: Singlet-A 6.1969 eV 200.07 nm f=0.0070 <S**2>=0.000
38 -> 43 0.53809
40 -> 45 -0.13747
40 -> 46 -0.41366

Excited State 19: Singlet-A 6.2745 eV 197.60 nm f=0.2224 <S**2>=0.000
31 -> 42 -0.11642
32 -> 42 0.28229
38 -> 43 -0.18960
39 -> 44 0.45349
40 -> 46 -0.29082
41 -> 47 0.15386

Excited State 20: Singlet-A 6.3338 eV 195.75 nm f=0.0482 <S**2>=0.000
32 -> 42 -0.33982
40 -> 47 0.12789
41 -> 45 -0.14941
41 -> 47 0.51917
41 -> 49 -0.18466

Excited State 21: Singlet-A 6.4264 eV 192.93 nm f=0.0795 <S**2>=0.000
31 -> 42 -0.19015
32 -> 42 0.44308
38 -> 43 0.15893
39 -> 44 -0.27046
40 -> 46 0.16857
40 -> 47 0.14162
41 -> 47 0.29344

Excited State 22: Singlet-A 6.5111 eV 190.42 nm f=0.0651 <S**2>=0.000
39 -> 46 -0.10469
40 -> 45 -0.16995
40 -> 47 0.56643

40 -> 49 -0.18617
41 -> 47 -0.10167
41 -> 49 0.15594

Excited State 23: Singlet-A 6.5899 eV 188.14 nm f=0.0010 <S**2>=0.000
39 -> 45 0.66008
39 -> 46 -0.15281
39 -> 47 -0.13574

Excited State 24: Singlet-A 6.6602 eV 186.16 nm f=0.0093 <S**2>=0.000
31 -> 42 -0.12104
37 -> 43 0.61680
37 -> 46 0.11125
40 -> 47 0.14255
41 -> 47 -0.10966
41 -> 49 -0.14045

Excited State 25: Singlet-A 6.6863 eV 185.43 nm f=0.0355 <S**2>=0.000
29 -> 42 0.14261
31 -> 42 0.40488
32 -> 42 0.12647
37 -> 43 0.27110
39 -> 44 0.10701
41 -> 47 0.16590
41 -> 48 -0.15874
41 -> 49 0.32130
****

22. TD-DFT calculation of nitrosoalkene 3B in IEFPCM

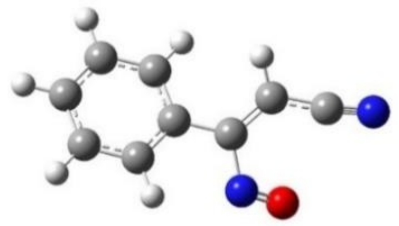

Excitation energies and oscillator strengths:
Excited State 1: Singlet-A 1.3581 eV 912.94 nm f=0.0008 <S**2>=0.000
40 -> 42 -0.30785
41 -> 42 0.62787
41 -> 43 0.10019
This state for optimization and/or second-order correction.
Total Energy, E(TD-HF/TD-DFT) = -531.151228670
Copying the excited state density for this state as the 1-particle RhoCI density.

Excited State 2: Singlet-A 2.6735 eV 463.75 nm f=0.1258 <S**2>=0.000
40 -> 42 0.62484
41 -> 42 0.31501

Excited State 3: Singlet-A 2.8588 eV 433.69 nm f=0.0054 <S**2>=0.000
39 -> 42 0.70258

Excited State 4: Singlet-A 4.0031 eV 309.72 nm f=0.0108 <S**2>=0.000
38 -> 42 0.15094
40 -> 43 -0.30772
41 -> 43 0.59424

Excited State 5: Singlet-A 4.2690 eV 290.43 nm f=0.0594 <S**2>=0.000
37 -> 42 0.21924
38 -> 42 0.63866
41 -> 43 -0.13413

Excited State 6: Singlet-A 4.6917 eV 264.26 nm f=0.3109 <S**2>=0.000
37 -> 42 0.40224
40 -> 43 0.47040
41 -> 43 0.30665

Excited State 7: Singlet-A 4.8378 eV 256.28 nm f=0.1275 <S**2>=0.000
37 -> 42 -0.32124
38 -> 42 0.10309
39 -> 43 0.51093
40 -> 43 0.23367
40 -> 44 0.14832
41 -> 44 0.16655

Excited State 8: Singlet-A 4.8462 eV 255.84 nm f=0.1585 <S**2>=0.000
37 -> 42 0.39946
38 -> 42 -0.18523
39 -> 43 0.37405
40 -> 43 -0.32244
40 -> 44 0.16122
41 -> 44 0.12740

Excited State 9: Singlet-A 5.2469 eV 236.30 nm f=0.0034 <S**2>=0.000
36 -> 42 0.69824

Excited State 10: Singlet-A 5.4780 eV 226.33 nm f=0.0116 <S**2>=0.000
33 -> 42 -0.11073
35 -> 42 0.65502
41 -> 44 0.18445

Excited State 11: Singlet-A 5.5308 eV 224.17 nm f=0.0118 <S**2>=0.000
35 -> 42 -0.20130
39 -> 43 -0.12159
40 -> 44 -0.24226
41 -> 44 0.60867

Excited State 12: Singlet-A 5.7552 eV 215.43 nm f=0.0824 <S**2>=0.000
34 -> 42 0.18472
39 -> 43 -0.23974
39 -> 45 -0.22636
40 -> 44 0.55725
41 -> 44 0.13150

Excited State 13: Singlet-A 5.8447 eV 212.13 nm f=0.0069 <S**2>=0.000
33 -> 42 0.40403
34 -> 42 0.53889

Excited State 14: Singlet-A 5.8776 eV 210.94 nm f=0.0124 <S**2>=0.000

33 -> 42 0.56104
34 -> 42 -0.36492
35 -> 42 0.10120

Excited State 15: Singlet-A 5.9859 eV 207.13 nm f=0.0117 <S**2>=0.000
39 -> 44 0.15801
40 -> 45 -0.17344
41 -> 45 0.52716
41 -> 46 -0.36177

Excited State 16: Singlet-A 6.0659 eV 204.39 nm f=0.0040 <S**2>=0.000
32 -> 42 0.11097
40 -> 46 0.19808
41 -> 45 0.32078
41 -> 46 0.55042

Excited State 17: Singlet-A 6.1548 eV 201.44 nm f=0.0154 <S**2>=0.000
32 -> 42 0.14810
38 -> 43 -0.30069
39 -> 44 0.18562
40 -> 45 0.19027
40 -> 46 0.47788
40 -> 47 0.13503
41 -> 45 -0.13070

Excited State 18: Singlet-A 6.1864 eV 200.41 nm f=0.1755 <S**2>=0.000
29 -> 42 -0.10473
31 -> 42 -0.14528
32 -> 42 0.52315
38 -> 43 -0.15238
39 -> 44 0.15215
40 -> 45 -0.14553
40 -> 46 -0.28852
41 -> 45 -0.11362

Excited State 19: Singlet-A 6.2145 eV 199.51 nm f=0.0220 <S**2>=0.000
32 -> 42 0.14524
38 -> 43 0.36299
39 -> 44 0.25990
40 -> 45 0.49529
41 -> 45 0.12372

Excited State 20: Singlet-A 6.3238 eV 196.06 nm f=0.2701 <S**2>=0.000
32 -> 42 -0.30154
34 -> 42 0.11214
38 -> 43 -0.33882
39 -> 44 0.41013
40 -> 46 -0.19101
41 -> 46 0.15871
41 -> 47 -0.10987

Excited State 21: Singlet-A 6.4677 eV 191.70 nm f=0.0755 <S**2>=0.000
39 -> 44 0.16856
39 -> 45 -0.13663
40 -> 47 0.18198
41 -> 47 0.59238
41 -> 48 0.15001

Excited State 22: Singlet-A 6.5832 eV 188.33 nm f=0.0268 <S**2>=0.000
31 -> 42 -0.15122
39 -> 46 0.14641
40 -> 46 -0.16600
40 -> 47 0.53440
40 -> 48 0.19244
41 -> 48 -0.21883

Excited State 23: Singlet-A 6.6379 eV 186.78 nm f=0.0041 <S**2>=0.000
31 -> 42 -0.18493
39 -> 45 0.17370
39 -> 46 0.55087
39 -> 47 -0.20697
40 -> 47 -0.12356
41 -> 48 0.12294

Excited State 24: Singlet-A 6.6498 eV 186.45 nm f=0.0185 <S**2>=0.000
26 -> 42 -0.12202
29 -> 42 0.23000
31 -> 42 0.49116
32 -> 42 0.19432
39 -> 45 0.16803
39 -> 46 0.21732
39 -> 47 -0.12513
40 -> 47 0.11332

Excited State 25: Singlet-A 6.7427 eV 183.88 nm f=0.1900 <S**2>=0.000
31 -> 42 -0.11780
38 -> 44 -0.23074
39 -> 45 0.50110
39 -> 46 -0.20824
40 -> 44 0.17630
41 -> 44 0.12269
41 -> 47 0.12433

23. TD-DFT calculation of nitrosoalkene 3B in SMD

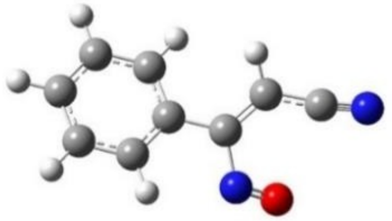

Excitation energies and oscillator strengths:
Excited State 1: Singlet-A 1.3617 eV 910.49 nm f=0.0008 <S**2>=0.000
40 -> 42 -0.35535
41 -> 42 0.60185
This state for optimization and/or second-order correction.
Total Energy, E(TD-HF/TD-DFT) = -531.157229276
Copying the excited state density for this state as the 1-particle RhoCI density.

Excited State 2: Singlet-A 2.6371 eV 470.15 nm f=0.1270 <S**2>=0.000

40 -> 42 0.59866
41 -> 42 0.36311

Excited State 3: Singlet-A 2.8301 eV 438.10 nm f=0.0060 <S**2>=0.000
39 -> 42 0.70193

Excited State 4: Singlet-A 4.0177 eV 308.60 nm f=0.0111 <S**2>=0.000
38 -> 42 0.16616
40 -> 43 -0.34982
41 -> 43 0.56780
Excited State 5: Singlet-A 4.2664 eV 290.61 nm f=0.0636 <S**2>=0.000
37 -> 42 0.19962
38 -> 42 0.63866
41 -> 43 -0.15726

Excited State 6: Singlet-A 4.6816 eV 264.83 nm f=0.4175 <S**2>=0.000
37 -> 42 0.28357
40 -> 43 0.51371
41 -> 43 0.35356

Excited State 7: Singlet-A 4.8227 eV 257.08 nm f=0.0258 <S**2>=0.000
37 -> 42 -0.10689
39 -> 43 0.63235
40 -> 44 0.18643
41 -> 44 0.21336

Excited State 8: Singlet-A 4.8599 eV 255.12 nm f=0.1640 <S**2>=0.000
36 -> 42 -0.17984
37 -> 42 0.54656
38 -> 42 -0.20305
40 -> 43 -0.29957

Excited State 9: Singlet-A 5.1676 eV 239.93 nm f=0.0036 <S**2>=0.000
36 -> 42 0.67126
37 -> 42 0.20739

Excited State 10: Singlet-A 5.4268 eV 228.46 nm f=0.0141 <S**2>=0.000
35 -> 42 0.68066
37 -> 42 -0.10649

Excited State 11: Singlet-A 5.5569 eV 223.12 nm f=0.0183 <S**2>=0.000
39 -> 43 -0.14193
40 -> 44 -0.24677
41 -> 44 0.62781

Excited State 12: Singlet-A 5.7457 eV 215.79 nm f=0.0861 <S**2>=0.000
34 -> 42 0.14409
39 -> 43 -0.22544
39 -> 45 -0.15469
39 -> 46 0.16575
40 -> 44 0.58001
41 -> 44 0.12653

Excited State 13: Singlet-A 5.8552 eV 211.75 nm f=0.0109 <S**2>=0.000
34 -> 42 0.65774

Excited State 14: Singlet-A 5.9356 eV 208.88 nm f=0.0052 <S**2>=0.000

33 -> 42 0.68417

Excited State 15: Singlet-A 5.9925 eV 206.90 nm f=0.0080 <S**2>=0.000
39 -> 44 -0.17698
40 -> 45 0.14182
40 -> 46 -0.10560
41 -> 45 -0.19181
41 -> 46 0.60341

Excited State 16: Singlet-A 6.0426 eV 205.18 nm f=0.0039 <S**2>=0.000
32 -> 42 0.11568
40 -> 46 0.24889
41 -> 45 0.57587
41 -> 46 0.23965

Excited State 17: Singlet-A 6.1418 eV 201.87 nm f=0.0095 <S**2>=0.000
38 -> 43 -0.25972
39 -> 44 0.18468
40 -> 45 0.46158
40 -> 46 0.29333
40 -> 47 0.14495
41 -> 45 -0.17172

Excited State 18: Singlet-A 6.1780 eV 200.69 nm f=0.1666 <S**2>=0.000
29 -> 42 -0.11570
31 -> 42 -0.14881
32 -> 42 0.51660
38 -> 43 -0.23294
39 -> 44 0.13114
40 -> 45 -0.26175
41 -> 45 -0.10947
41 -> 46 0.11550

Excited State 19: Singlet-A 6.2011 eV 199.94 nm f=0.0603 <S**2>=0.000
32 -> 42 -0.18606
38 -> 43 -0.32103
39 -> 44 -0.29453
40 -> 45 -0.32115
40 -> 46 0.37167
41 -> 45 -0.12517

Excited State 20: Singlet-A 6.3024 eV 196.72 nm f=0.2594 <S**2>=0.000
32 -> 42 -0.31306
34 -> 42 0.12058
38 -> 43 -0.36273
39 -> 44 0.38433
40 -> 46 -0.17263
41 -> 46 0.11797
41 -> 47 -0.12674

Excited State 21: Singlet-A 6.4302 eV 192.81 nm f=0.0855 <S**2>=0.000
39 -> 44 0.18285
39 -> 45 -0.11161
40 -> 47 0.15212
41 -> 47 0.59637
41 -> 48 0.13886

Excited State 22: Singlet-A 6.5440 eV 189.46 nm f=0.0266 <S**2>=0.000
31 -> 42 -0.10995
39 -> 46 0.11345
40 -> 45 -0.13300
40 -> 47 0.55079
40 -> 48 0.21084
41 -> 48 -0.22472

Excited State 23: Singlet-A 6.6014 eV 187.82 nm f=0.0028 <S**2>=0.000
39 -> 45 0.48864
39 -> 46 0.39291
39 -> 47 -0.23673
41 -> 48 0.10433

Excited State 24: Singlet-A 6.6275 eV 187.08 nm f=0.0201 <S**2>=0.000
26 -> 42 0.13579
29 -> 42 0.25353
31 -> 42 0.51544
32 -> 42 0.19890
39 -> 45 0.15721
40 -> 46 0.11617
40 -> 47 0.10728

Excited State 25: Singlet-A 6.7079 eV 184.83 nm f=0.2156 <S**2>=0.000
31 -> 42 0.14078
38 -> 44 0.19960
39 -> 45 -0.31246
39 -> 46 0.45295
40 -> 44 -0.16349
40 -> 46 -0.11387
41 -> 44 -0.13355
41 -> 47 -0.12882
****

24. TD-DFT calculation of nitrosoalkene 3B in I-PCM

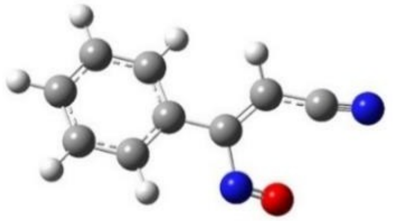

Excitation energies and oscillator strengths:
Excited State 1: Singlet-A 1.3182 eV 940.58 nm f=0.0006 <S**2>=0.000
41 -> 42 0.69469
41 -> 43 -0.11985
This state for optimization and/or second-order correction.
Total Energy, E(TD-HF/TD-DFT) = -531.143518122
Copying the excited state density for this state as the 1-particle RhoCI density.

Excited State 2: Singlet-A 2.9114 eV 425.85 nm f=0.1040 <S**2>=0.000
40 -> 42 0.68894
40 -> 43 -0.10886

Excited State 3: Singlet-A 3.1516 eV 393.40 nm f=0.0036 <S**2>=0.000
39 -> 42 0.70415

Excited State 4: Singlet-A 3.8617 eV 321.06 nm f=0.0073 <S**2>=0.000
37 -> 42 0.10541
40 -> 43 -0.11028
41 -> 42 0.10983
41 -> 43 0.66246

Excited State 5: Singlet-A 4.3131 eV 287.46 nm f=0.0220 <S**2>=0.000
37 -> 42 -0.38020
38 -> 42 0.57959

Excited State 6: Singlet-A 4.6589 eV 266.12 nm f=0.0517 <S**2>=0.000
37 -> 42 0.54683
38 -> 42 0.31643
40 -> 43 -0.22602
41 -> 43 -0.15663

Excited State 7: Singlet-A 4.9073 eV 252.65 nm f=0.3863 <S**2>=0.000
37 -> 42 0.18160
38 -> 42 0.16012
39 -> 43 -0.11851
40 -> 42 0.10847
40 -> 43 0.61400
40 -> 44 0.11322

Excited State 8: Singlet-A 4.9373 eV 251.12 nm f=0.0480 <S**2>=0.000
39 -> 43 0.55594
40 -> 43 0.16100
40 -> 44 -0.30577
41 -> 44 -0.24222

Excited State 9: Singlet-A 5.2035 eV 238.27 nm f=0.0016 <S**2>=0.000
39 -> 43 0.16794
40 -> 44 -0.20922
41 -> 44 0.64960

Excited State 10: Singlet-A 5.4802 eV 226.24 nm f=0.0051 <S**2>=0.000
35 -> 42 0.62273
36 -> 42 -0.31684

Excited State 11: Singlet-A 5.5666 eV 222.73 nm f=0.0008 <S**2>=0.000
35 -> 42 0.32211
36 -> 42 0.62300

Excited State 12: Singlet-A 5.7613 eV 215.20 nm f=0.0302 <S**2>=0.000
39 -> 43 0.17454
40 -> 44 0.24559
41 -> 45 0.21093
41 -> 46 0.56097

Excited State 13: Singlet-A 5.8003 eV 213.75 nm f=0.0561 <S**2>=0.000
33 -> 42 0.27443
39 -> 43 0.26658
39 -> 46 0.17850
40 -> 44 0.42954

```
41 -> 46 -0.32310

Excited State 14: Singlet-A 5.8281 eV 212.73 nm f=0.0095 <S**2>=0.000
33 -> 42 0.30753
34 -> 42 0.59395
40 -> 44 -0.11164

Excited State 15: Singlet-A 5.9049 eV 209.97 nm f=0.0126 <S**2>=0.000
33 -> 42 0.11531
34 -> 42 -0.14863
40 -> 44 -0.10089
41 -> 45 0.60644
41 -> 46 -0.15672
41 -> 47 0.15516

Excited State 16: Singlet-A 5.9461 eV 208.51 nm f=0.0363 <S**2>=0.000
33 -> 42 0.51672
34 -> 42 -0.31145
39 -> 43 -0.10646
40 -> 44 -0.14943
41 -> 45 -0.18011
41 -> 46 0.13895

Excited State 17: Singlet-A 6.1311 eV 202.22 nm f=0.0022 <S**2>=0.000
39 -> 44 -0.10801
40 -> 45 0.64707
40 -> 47 0.13705
41 -> 47 -0.10745

Excited State 18: Singlet-A 6.1969 eV 200.07 nm f=0.0070 <S**2>=0.000
38 -> 43 0.53800
40 -> 45 -0.13792
40 -> 46 -0.41363

Excited State 19: Singlet-A 6.2745 eV 197.60 nm f=0.2225 <S**2>=0.000
31 -> 42 -0.11642
32 -> 42 0.28228
38 -> 43 -0.18958
39 -> 44 0.45347
40 -> 46 -0.29075
41 -> 47 0.15384

Excited State 20: Singlet-A 6.3338 eV 195.75 nm f=0.0482 <S**2>=0.000
32 -> 42 -0.33991
40 -> 47 0.12788
41 -> 45 -0.14960
41 -> 47 0.51905
41 -> 49 -0.18459

Excited State 21: Singlet-A 6.4264 eV 192.93 nm f=0.0796 <S**2>=0.000
31 -> 42 -0.19017
32 -> 42 0.44302
38 -> 43 0.15889
39 -> 44 -0.27058
40 -> 46 0.16852
40 -> 47 0.14151
41 -> 47 0.29351
```

```
Excited State 22: Singlet-A 6.5111 eV 190.42 nm f=0.0651 <S**2>=0.000
39 -> 46 -0.10464
40 -> 45 -0.17020
40 -> 47 0.56646
40 -> 49 -0.18610
41 -> 47 -0.10164
41 -> 49 0.15580

Excited State 23: Singlet-A 6.5901 eV 188.14 nm f=0.0010 <S**2>=0.000
39 -> 45 0.66011
39 -> 46 -0.15279
39 -> 47 -0.13569

Excited State 24: Singlet-A 6.6602 eV 186.16 nm f=0.0092 <S**2>=0.000
31 -> 42 -0.12094
37 -> 43 0.61711
37 -> 46 0.11130
40 -> 47 0.14236
41 -> 47 -0.10938
41 -> 49 -0.13969

Excited State 25: Singlet-A 6.6864 eV 185.43 nm f=0.0354 <S**2>=0.000
29 -> 42 0.14302
31 -> 42 0.40604
32 -> 42 0.12726
37 -> 43 0.27041
39 -> 44 0.10690
41 -> 47 0.16561
41 -> 48 -0.15919
41 -> 49 0.31984
**********************************************************************
```

25. TD-DFT calculation of nitrosoalkene 3B in C-PCM

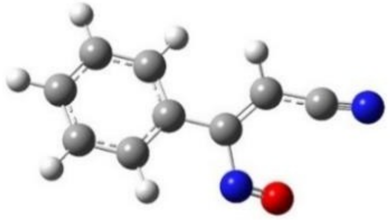

```
Excitation energies and oscillator strengths:
Excited State 1: Singlet-A 1.3608 eV 911.10 nm f=0.0009 <S**2>=0.000
40 -> 42 -0.31767
41 -> 42 0.62276
This state for optimization and/or second-order correction.
Total Energy, E(TD-HF/TD-DFT) = -531.151729953
Copying the excited state density for this state as the 1-particle RhoCI
density.

Excited State 2: Singlet-A 2.6655 eV 465.14 nm f=0.1284 <S**2>=0.000
40 -> 42 0.62016
41 -> 42 0.32494

Excited State 3: Singlet-A 2.8578 eV 433.85 nm f=0.0055 <S**2>=0.000
39 -> 42 0.70260
```

Excited State 4: Singlet-A 4.0085 eV 309.30 nm f=0.0112 <S**2>=0.000
38 -> 42 0.15306
40 -> 43 -0.31589
41 -> 43 0.58948

Excited State 5: Singlet-A 4.2631 eV 290.83 nm f=0.0640 <S**2>=0.000
37 -> 42 0.21167
38 -> 42 0.64070
41 -> 43 -0.13849
Excited State 6: Singlet-A 4.6879 eV 264.48 nm f=0.3382 <S**2>=0.000
37 -> 42 0.38284
40 -> 43 0.48107
41 -> 43 0.31639

Excited State 7: Singlet-A 4.8355 eV 256.40 nm f=0.1296 <S**2>=0.000
37 -> 42 -0.35608
38 -> 42 0.11153
39 -> 43 0.48967
40 -> 43 0.23519
40 -> 44 0.13674
41 -> 44 0.16026

Excited State 8: Singlet-A 4.8433 eV 255.99 nm f=0.1418 <S**2>=0.000
37 -> 42 0.39147
38 -> 42 -0.17531
39 -> 43 0.40339
40 -> 43 -0.29814
40 -> 44 0.16490
41 -> 44 0.13692

Excited State 9: Singlet-A 5.2414 eV 236.55 nm f=0.0035 <S**2>=0.000
36 -> 42 0.69764

Excited State 10: Singlet-A 5.4753 eV 226.44 nm f=0.0121 <S**2>=0.000
33 -> 42 -0.10884
35 -> 42 0.66343
41 -> 44 0.15754

Excited State 11: Singlet-A 5.5354 eV 223.99 nm f=0.0140 <S**2>=0.000
35 -> 42 -0.17286
39 -> 43 -0.12707
40 -> 44 -0.24241
41 -> 44 0.61620

Excited State 12: Singlet-A 5.7497 eV 215.64 nm f=0.0904 <S**2>=0.000
34 -> 42 0.17076
39 -> 43 -0.23859
39 -> 45 -0.22725
40 -> 44 0.56575
41 -> 44 0.13115

Excited State 13: Singlet-A 5.8484 eV 212.00 nm f=0.0087 <S**2>=0.000
33 -> 42 0.31632
34 -> 42 0.59006

Excited State 14: Singlet-A 5.8819 eV 210.79 nm f=0.0108 <S**2>=0.000

33 -> 42 0.61523
34 -> 42 -0.28364
35 -> 42 0.10626

Excited State 15: Singlet-A 5.9895 eV 207.00 nm f=0.0109 <S**2>=0.000
39 -> 44 0.16288
40 -> 45 -0.17940
41 -> 45 0.56639
41 -> 46 -0.29475

Excited State 16: Singlet-A 6.0718 eV 204.20 nm f=0.0038 <S**2>=0.000
32 -> 42 0.11811
40 -> 45 -0.12128
40 -> 46 0.19620
41 -> 45 0.24482
41 -> 46 0.58233

Excited State 17: Singlet-A 6.1567 eV 201.38 nm f=0.0301 <S**2>=0.000
31 -> 42 -0.10119
32 -> 42 0.19712
38 -> 43 -0.31862
39 -> 44 0.20934
40 -> 45 0.12481
40 -> 46 0.45451
40 -> 47 0.12562
41 -> 45 -0.13901
41 -> 46 -0.10889

Excited State 18: Singlet-A 6.1848 eV 200.47 nm f=0.1741 <S**2>=0.000
31 -> 42 -0.13059
32 -> 42 0.51115
38 -> 43 -0.10187
39 -> 44 0.14473
40 -> 46 -0.35710
41 -> 45 -0.10145

Excited State 19: Singlet-A 6.2126 eV 199.57 nm f=0.0209 <S**2>=0.000
32 -> 42 0.11444
38 -> 43 0.36404
39 -> 44 0.26155
40 -> 45 0.50505
41 -> 45 0.12967

Excited State 20: Singlet-A 6.3191 eV 196.21 nm f=0.2790 <S**2>=0.000
32 -> 42 -0.30933
34 -> 42 0.11668
38 -> 43 -0.33784
39 -> 44 0.40717
40 -> 46 -0.18685
41 -> 46 0.16812

Excited State 21: Singlet-A 6.4683 eV 191.68 nm f=0.0703 <S**2>=0.000
39 -> 44 0.16067
39 -> 45 -0.13712
40 -> 47 0.17641
41 -> 47 0.59690
41 -> 48 0.14645

Excited State 22: Singlet-A 6.5857 eV 188.26 nm f=0.0251 <S**2>=0.000
31 -> 42 -0.15752
39 -> 46 0.13211
40 -> 46 -0.17059
40 -> 47 0.53474
40 -> 48 0.18914
41 -> 48 -0.22033

Excited State 23: Singlet-A 6.6415 eV 186.68 nm f=0.0139 <S**2>=0.000
29 -> 42 -0.18127
31 -> 42 -0.38714
32 -> 42 -0.14488
39 -> 46 0.39547
39 -> 47 -0.13054
40 -> 45 0.11785
40 -> 47 -0.16243
41 -> 47 0.10033
41 -> 48 0.12196

Excited State 24: Singlet-A 6.6498 eV 186.45 nm f=0.0091 <S**2>=0.000
29 -> 42 0.16479
31 -> 42 0.34582
32 -> 42 0.13606
39 -> 45 0.18066
39 -> 46 0.46752
39 -> 47 -0.21495

Excited State 25: Singlet-A 6.7296 eV 184.24 nm f=0.2077 <S**2>=0.000
31 -> 42 -0.13267
38 -> 44 -0.21782
39 -> 45 0.52904
39 -> 46 -0.14880
40 -> 44 0.17182
40 -> 45 -0.10729
41 -> 44 0.12386
41 -> 47 0.11865
****

26. TS calculation to nitrosoalkene ^{3}3

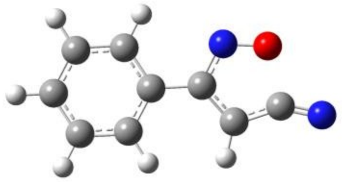

DFT/B3LYP 6-31+G(d), E = -531.158414 a.u.

#Imaginary Frequency=1
Standard orientation:

Center Number	Atomic Number	Atomic Type	Coordinates (Angstroms) X	Y	Z

1	6	0	2.960895	-0.515147	-0.053111
2	6	0	1.623172	-0.960404	-0.094294
3	6	0	0.694611	0.081906	0.011833
4	1	0	1.387394	-2.008227	-0.213969
5	7	0	1.303113	1.282695	0.125932
6	7	0	4.154043	-0.773046	-0.082854
7	8	0	2.589319	1.221020	0.114307
8	6	0	-0.774795	0.000510	0.005423
9	6	0	-1.428857	-1.240144	0.088804
10	6	0	-2.821936	-1.308225	0.076429
11	6	0	-3.582383	-0.139083	-0.016557
12	6	0	-2.939289	1.099995	-0.095939
13	6	0	-1.547164	1.172337	-0.085336
14	1	0	-0.854527	-2.158121	0.174380
15	1	0	-3.312665	-2.275392	0.143892
16	1	0	-4.667680	-0.193278	-0.025366
17	1	0	-3.522880	2.013836	-0.168096
18	1	0	-1.049804	2.135005	-0.150350

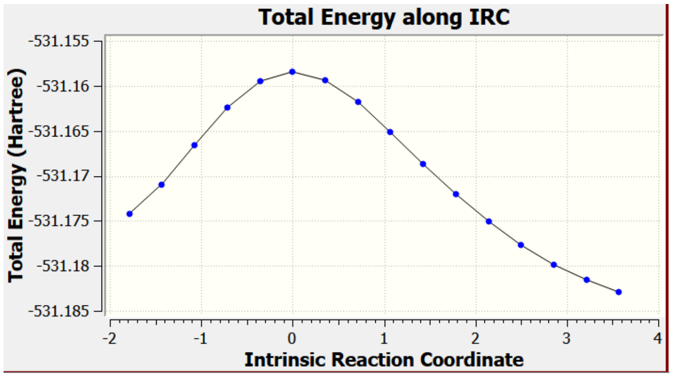

27. Spin density calculation of T_1 of 1A

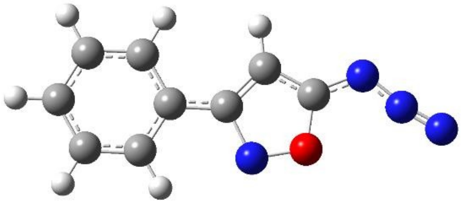

Atom	Number	Alpha spin	Beta spin	Spin density
C	1	0.06044	0.39226	-0.33182
C	2	-0.12835	-0.18156	0.05321
C	3	-0.05029	0.13923	-0.18952
H	4	0.13428	0.13467	-0.00039
N	5	-0.40048	0.38808	-0.78856
N	6	-0.14061	-0.15024	0.00963
N	7	0.04792	0.11685	-0.06893
N	8	-0.16284	0.16188	-0.32472
O	9	-0.25852	-0.12838	-0.13014
C	10	-0.03937	-0.05654	0.01717
C	11	-0.14495	-0.06068	-0.08427
C	12	-0.10242	-0.13563	0.03321
C	13	-0.18179	-0.04588	-0.13591
C	14	-0.09265	-0.14616	0.05351
C	15	-0.16395	-0.04306	-0.12089
H	16	0.12021	0.11744	0.00277
H	17	0.12279	0.1237	-0.00091
H	18	0.12465	0.12041	0.00424
H	19	0.1231	0.1246	-0.0015
H	20	0.13282	0.12903	0.00379

28. Spin density calculation of nitrene ^{3}2

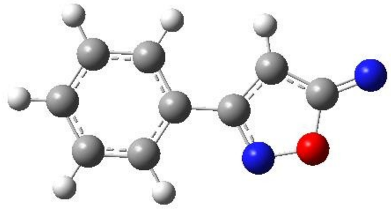

Atom	Number	Alpha spin	Beta spin	Spin density
C	1	0.25316	0.06185	0.19131
C	2	-0.34531	0.08504	-0.43035
C	3	0.12805	-0.00132	0.12937
H	4	0.13912	0.12588	0.01324
N	5	-0.21839	0.15465	-0.37304
N	6	-0.78043	0.66692	-1.44735
O	7	-0.20943	-0.11122	-0.09821
C	8	-0.06137	-0.03342	-0.02795
C	9	-0.09186	-0.11636	0.0245
C	10	-0.12458	-0.11081	-0.01377
C	11	-0.10069	-0.1242	0.02351
C	12	-0.1232	-0.11004	-0.01316

C	13	-0.08741	-0.11082	0.02341
H	14	0.11896	0.11979	-0.00083
H	15	0.12426	0.12384	0.00042
H	16	0.12339	0.12412	-0.00073
H	17	0.12474	0.12446	0.00028
H	18	0.13101	0.13163	-0.00062

B. Quantum chemical calculations using CAM-B3LYP

1. Optimization of 1A

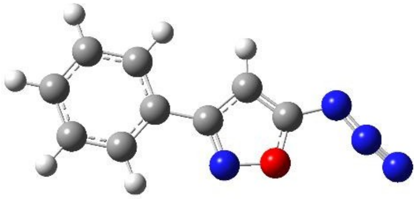

DFT/CAM-B3LYP 6-31+G(d), E = -640.388943 a.u.
Standard orientation:

Center Number	Atomic Number	Atomic Type	Coordinates (Angstroms) X	Y	Z
1	6	0	-2.020756	0.405551	0.000245
2	6	0	-0.811561	1.018385	0.001043
3	6	0	0.107523	-0.071792	-0.000191
4	1	0	-0.620302	2.078837	0.002482
5	7	0	-0.515978	-1.227038	-0.001046
6	7	0	-3.291342	0.953694	0.000530
7	7	0	-4.241078	0.151524	0.000270
8	7	0	-5.200304	-0.439202	-0.000049
9	8	0	-1.879441	-0.925665	-0.001290
10	6	0	1.581619	-0.021852	-0.000066
11	6	0	2.248365	1.204565	-0.000724
12	6	0	3.638357	1.252737	-0.000592
13	6	0	4.376713	0.074255	0.000206
14	6	0	3.717312	-1.153166	0.000877
15	6	0	2.329986	-1.203414	0.000755
16	1	0	1.684400	2.131875	-0.001432
17	1	0	4.143261	2.213995	-0.001132
18	1	0	5.461961	0.110730	0.000320
19	1	0	4.287662	-2.077157	0.001525
20	1	0	1.814114	-2.157411	0.001319

2. Optimization of 1B

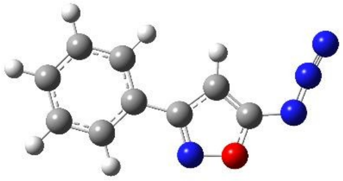

DFT/CAM-B3LYP 6-31+G(d), E = -640.386991 a.u.
Standard orientation:

Center Number	Atomic Number	Atomic Type	Coordinates (Angstroms) X	Y	Z
1	6	0	2.114650	-0.496945	-0.000015
2	6	0	1.084153	0.389890	0.000010
3	6	0	-0.065865	-0.454450	-0.000017
4	1	0	1.135990	1.467326	0.000051
5	7	0	0.275218	-1.721370	-0.000057
6	7	0	3.498787	-0.402408	-0.000007
7	7	0	3.954108	0.750045	0.000032
8	7	0	4.491543	1.741848	0.000065
9	8	0	1.662088	-1.751158	-0.000055
10	6	0	-1.487186	-0.061893	-0.000005
11	6	0	-1.851260	1.285771	-0.000076
12	6	0	-3.192347	1.655157	-0.000065
13	6	0	-4.183904	0.679790	0.000018
14	6	0	-3.826999	-0.667257	0.000090
15	6	0	-2.489000	-1.037975	0.000079
16	1	0	-1.088022	2.057693	-0.000147
17	1	0	-3.460631	2.707315	-0.000123
18	1	0	-5.231158	0.966914	0.000027
19	1	0	-4.596071	-1.433836	0.000156
20	1	0	-2.207856	-2.085477	0.000136

3. Optimization of nitrene [1]2

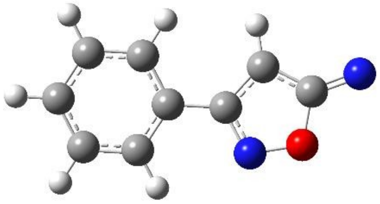

DFT/CAM-B3LYP 6-31+G(d), E = -530.881882 a.u.
Standard orientation:

Center Number	Atomic Number	Atomic Type	Coordinates (Angstroms) X	Y	Z
1	6	0	2.877721	0.309443	-0.077446
2	6	0	1.641978	0.927861	-0.212640
3	6	0	0.692316	-0.088304	0.028220
4	1	0	1.487294	1.963234	-0.473102
5	7	0	1.309031	-1.229873	0.287846
6	7	0	4.100958	0.753031	-0.193383
7	8	0	2.639779	-1.019877	0.227994
8	6	0	-0.779500	-0.011521	0.010381
9	6	0	-1.425786	1.214969	0.176125
10	6	0	-2.814160	1.287921	0.153098
11	6	0	-3.569943	0.134827	-0.032808

12	6	0	-2.930264	-1.092068	-0.194622
13	6	0	-1.543803	-1.167816	-0.173933
14	1	0	-0.849644	2.119926	0.342735
15	1	0	-3.304498	2.247198	0.287216
16	1	0	-4.654089	0.191258	-0.051488
17	1	0	-3.514379	-1.995286	-0.342251
18	1	0	-1.044186	-2.121294	-0.306546

4. Optimization of nitrene ^{3}2

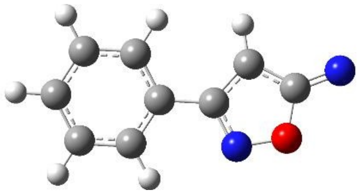

DFT/CAM-B3LYP 6-31+G(d), E =-530.904425 a.u.
Standard orientation:

Center Number	Atomic Number	Atomic Type	Coordinates (Angstroms) X	Y	Z
1	6	0	2.879984	0.317994	-0.036469
2	6	0	1.641387	0.945750	-0.098116
3	6	0	0.691915	-0.091207	0.013363
4	1	0	1.485019	2.006475	-0.218286
5	7	0	1.308344	-1.257802	0.133640
6	7	0	4.102083	0.768915	-0.090002
7	8	0	2.637278	-1.045162	0.105845
8	6	0	-0.779491	-0.011754	0.004618
9	6	0	-1.420768	1.226260	0.082596
10	6	0	-2.809165	1.301303	0.072283
11	6	0	-3.569607	0.139369	-0.014895
12	6	0	-2.934807	-1.098305	-0.091328
13	6	0	-1.548176	-1.176518	-0.082215
14	1	0	-0.839325	2.139950	0.159994
15	1	0	-3.295945	2.269656	0.135271
16	1	0	-4.653793	0.197455	-0.023243
17	1	0	-3.522802	-2.008382	-0.160549
18	1	0	-1.051996	-2.139007	-0.144448

5. Optimization of nitrosoalkene ^{3}3A

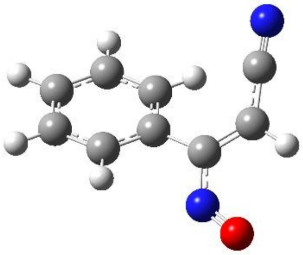

DFT/CAM-B3LYP 6-31+G(d), E =-530.912016 a.u.
Standard orientation:

Center Number	Atomic Number	Atomic Type	Coordinates (Angstroms) X	Y	Z
1	6	0	1.263920	-1.185193	-0.547628
2	6	0	0.400713	-0.239244	0.023314
3	6	0	0.940948	0.908319	0.616846
4	6	0	2.314182	1.114433	0.621191
5	6	0	3.164913	0.178680	0.038417
6	6	0	2.636393	-0.969852	-0.544033
7	1	0	0.856176	-2.081192	-1.000636
8	1	0	0.290603	1.636860	1.087974
9	1	0	2.720187	2.007441	1.085824
10	1	0	4.238222	0.342865	0.042241
11	1	0	3.294966	-1.703532	-0.998478
12	6	0	-1.050595	-0.471658	0.014410
13	6	0	-2.057731	0.411557	-0.105207
14	6	0	-1.899723	1.811630	-0.305967
15	7	0	-1.409386	-1.888913	0.048976
16	7	0	-1.815108	2.955222	-0.468657
17	8	0	-2.503598	-2.132168	0.490702
18	1	0	-3.078037	0.038715	-0.072833

6. Optimization of nitrosoalkene ^{3}3B

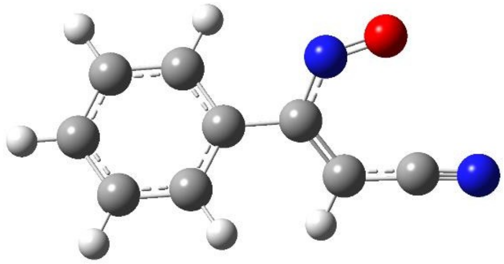

DFT/CAM-B3LYP 6-31+G(d), E =-530.911449 a.u.
Standard orientation:

Center Number	Atomic Number	Atomic Type	Coordinates (Angstroms) X	Y	Z
1	6	0	-1.529266	1.090757	-0.401950
2	6	0	-0.724064	0.002060	-0.012703
3	6	0	-1.345815	-1.196453	0.386662
4	6	0	-2.734268	-1.312745	0.371951
5	6	0	-3.525832	-0.231928	-0.031626
6	6	0	-2.918866	0.967167	-0.414384
7	1	0	-1.062705	2.021005	-0.706104
8	1	0	-0.741805	-2.028847	0.737639
9	1	0	-3.199559	-2.242174	0.688798
10	1	0	-4.608690	-0.322659	-0.039060
11	1	0	-3.528051	1.811170	-0.726283

12	6	0	0.743281	0.119778	-0.003609
13	6	0	1.598202	-0.901210	-0.276059
14	6	0	3.018874	-0.845161	-0.267107
15	7	0	1.222764	1.478383	0.187413
16	7	0	4.180915	-0.930502	-0.314467
17	8	0	2.329364	1.578963	0.676610
18	1	0	1.186673	-1.858949	-0.585543

7. Optimization of nitrosoalkene [1]3A

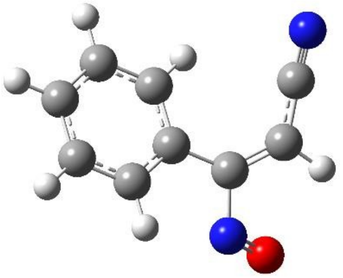

DFT/CAM-B3LYP 6-31+G(d), E =-530.919939 a.u.
Standard orientation:

Center Number	Atomic Number	Atomic Type	Coordinates (Angstroms) X	Y	Z
1	6	0	1.286718	-1.191639	-0.523645
2	6	0	0.407665	-0.233614	0.023213
3	6	0	0.943989	0.938703	0.590417
4	6	0	2.320297	1.154957	0.592351
5	6	0	3.183856	0.206834	0.033610
6	6	0	2.662516	-0.964628	-0.522336
7	1	0	0.887341	-2.101327	-0.957412
8	1	0	0.288291	1.674880	1.043210
9	1	0	2.718992	2.063190	1.035669
10	1	0	4.256831	0.379363	0.035797
11	1	0	3.327824	-1.705420	-0.957432
12	6	0	-1.041151	-0.479656	0.022508
13	6	0	-2.069596	0.400067	-0.084435
14	6	0	-1.947475	1.797754	-0.292988
15	7	0	-1.384396	-1.905013	0.045996
16	7	0	-1.895228	2.949951	-0.464260
17	8	0	-2.490045	-2.175202	0.466662
18	1	0	-3.082466	0.003690	-0.037451

8. Optimization of nitrosoalkene [1]3B

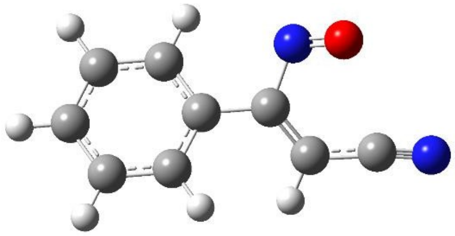

DFT/CAM-B3LYP 6-31+G(d), E =-530.916497 a.u.
Standard orientation:

Center Number	Atomic Number	Atomic Type	Coordinates (Angstroms) X	Y	Z
1	6	0	-1.529266	1.090757	-0.401950
2	6	0	-0.724064	0.002060	-0.012703
3	6	0	-1.345815	-1.196453	0.386662
4	6	0	-2.734268	-1.312745	0.371951
5	6	0	-3.525832	-0.231928	-0.031626
6	6	0	-2.918866	0.967167	-0.414384
7	1	0	-1.062705	2.021005	-0.706104
8	1	0	-0.741805	-2.028847	0.737639
9	1	0	-3.199559	-2.242174	0.688798
10	1	0	-4.608690	-0.322659	-0.039060
11	1	0	-3.528051	1.811170	-0.726283
12	6	0	0.743281	0.119778	-0.003609
13	6	0	1.598202	-0.901210	-0.276059
14	6	0	3.018874	-0.845161	-0.267107
15	7	0	1.222764	1.478383	0.187413
16	7	0	4.180915	-0.930502	-0.314467
17	8	0	2.329364	1.578963	0.676610
18	1	0	1.186673	-1.858949	-0.585543

9. TD-DFT calculation of 1A

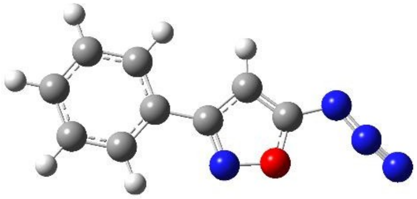

Excitation energies and oscillator strengths:

```
 Excited State   1: Singlet-A   4.1181 eV  301.07 nm  f=0.0003  <S**2>=0.000
      43 -> 50   0.22736
      46 -> 50         0.10969
      47 -> 50        -0.41511
      48 -> 50         0.49822
 This state for optimization and/or second-order correction.
 Total Energy, E(TD-HF/TD-DFT) =  -640.237606840
 Copying the excited state density for this state as the 1-particle RhoCI
density.

 Excited State   2: Singlet-A   5.0868 eV  243.74 nm  f=0.2892  <S**2>=0.000
      40 -> 50         0.12488
      45 -> 50        -0.11792
      47 -> 49        -0.38998
      48 -> 49         0.52053

 Excited State   3: Singlet-A  5.1833 eV  239.20 nm  f=0.0058  <S**2>=0.000
      46 -> 49         0.36553
      46 -> 51         0.34584
```

```
     47 -> 49         0.17426
     47 -> 52        -0.27404
     48 -> 49         0.12189
     48 -> 51        -0.16344
     48 -> 52        -0.28594

 Excited State   4: Singlet-A  5.3440 eV  232.01 nm  f=0.4320  <S**2>=0.000
     46 -> 49        -0.17019
     46 -> 52         0.12352
     47 -> 49         0.45189
     47 -> 51         0.21467
     48 -> 49         0.35286
     48 -> 51         0.24319

 Excited State   5: Singlet-A 5.7829 eV  214.40 nm  f=0.0418  <S**2>=0.000
     40 -> 50        -0.10908
     44 -> 49         0.14523
     46 -> 51         0.15533
     47 -> 49        -0.21576
     47 -> 51        -0.12621
     47 -> 60         0.11080
     48 -> 51         0.54409
     48 -> 52        -0.14562

 Excited State   6: Singlet-A  6.1819 eV  200.56 nm  f=0.0005  <S**2>=0.000
     44 -> 50        -0.16431
     45 -> 49        -0.17616
     47 -> 50         0.50397
     48 -> 50         0.40600

 Excited State   7:  Singlet-A   6.1820 eV  200.56 nm  f=0.0126  <S**2>=0.000
     40 -> 50         0.11382
     46 -> 49         0.12901
     46 -> 51         0.11318
     46 -> 52         0.16698
     47 -> 51         0.52831
     48 -> 49        -0.22630
     48 -> 51         0.11460
     48 -> 60         0.10533

 Excited State   8:  Singlet-A   6.1897 eV  200.31 nm  f=0.0040  <S**2>=0.000
     45 -> 49         0.63462
     45 -> 60        -0.11450
     47 -> 50         0.12592
     48 -> 50         0.14034

 Excited State   9: Singlet-A  6.2718 eV  197.68 nm  f=0.0061  <S**2>=0.000
     47 -> 53        -0.28368
     47 -> 54         0.24437
     48 -> 53         0.43253
     48 -> 54        -0.32700

 Excited State  10: Singlet-A  6.4904 eV  191.03 nm  f=0.3798  <S**2>=0.000
     46 -> 49         0.47318
     46 -> 52        -0.24342
     47 -> 49         0.12897
     47 -> 52         0.15861
```

```
      48 -> 51         0.13171
      48 -> 52         0.34782

 Excited State  11: Singlet-A 6.6756 eV  185.73 nm  f=0.0052  <S**2>=0.000
      47 -> 53         0.42650
      47 -> 54         0.14878
      48 -> 53         0.40465
      48 -> 54         0.27533

 Excited State  12: Singlet-A6.7508 eV  183.66 nm  f=0.6363  <S**2>=0.000
      40 -> 50        -0.18461
      42 -> 50         0.13543
      43 -> 49        -0.12620
      43 -> 51         0.11473
      45 -> 50         0.20401
      46 -> 51         0.19705
      46 -> 52         0.27995
      47 -> 52         0.36831
      48 -> 51        -0.21467
      48 -> 58        -0.10425

 Excited State  13: Singlet-A 6.8368 eV  181.35 nm  f=0.0630  <S**2>=0.000
      40 -> 50         0.10118
      43 -> 49         0.11656
      44 -> 51         0.13229
      45 -> 50        -0.12152
      46 -> 51         0.25732
      46 -> 52         0.32583
      47 -> 51        -0.27415
      47 -> 52        -0.10503
      48 -> 52         0.34045
      48 -> 58         0.10389

 Excited State  14: Singlet-A  6.8446 eV  181.14 nm  f=0.0000  <S**2>=0.000
      40 -> 49         0.34090
      40 -> 51        -0.18095
      42 -> 49        -0.26100
      43 -> 50         0.24660
      44 -> 50        -0.12503
      45 -> 51         0.33235
      48 -> 50        -0.11209

 Excited State  15: Singlet-A   6.9025 eV  179.62 nm  f=0.0004  <S**2>=0.000
      46 -> 50         0.14329
      46 -> 53         0.44824
      46 -> 54         0.29605
      47 -> 53         0.23834
      47 -> 55        -0.11859
      48 -> 54        -0.19276

 Excited State  16: Singlet-A 6.9964 eV  177.21 nm  f=0.0007  <S**2>=0.000
      40 -> 49        -0.14108
      42 -> 49         0.12348
      43 -> 50         0.31402
      44 -> 50        -0.17051
      45 -> 51        -0.16484
      46 -> 50         0.29519
```

```
      46 -> 53        -0.24957
      46 -> 54        -0.19176
      47 -> 55        -0.11955
      48 -> 50        -0.15223
      48 -> 55         0.14552

 Excited State  17: Singlet-A   7.0192 eV  176.64 nm  f=0.0938  <S**2>=0.000
      44 -> 51        -0.10316
      46 -> 49        -0.24795
      46 -> 51         0.35377
      46 -> 52        -0.34053
      46 -> 58         0.10204
      47 -> 51         0.13994
      47 -> 52        -0.12360
      48 -> 52         0.28324

 Excited State  18: Singlet-A 7.0486 eV  175.90 nm  f=0.0004  <S**2>=0.000
      46 -> 50         0.47041
      46 -> 53         0.14110
      47 -> 53        -0.22152
      47 -> 55         0.20500
      47 -> 56         0.11377
      48 -> 53         0.10199
      48 -> 54         0.21386
      48 -> 55        -0.18901

 Excited State  19: Singlet-A  7.1290 eV  173.91 nm  f=0.0034  <S**2>=0.000
      43 -> 50         0.26490
      44 -> 50        -0.15251
      46 -> 50        -0.28400
      47 -> 53        -0.16255
      47 -> 54         0.32934
      47 -> 55         0.14024
      48 -> 54         0.22863
      48 -> 55         0.13907

 Excited State  20:  Singlet-A   7.1510 eV  173.38 nm  f=0.0035  <S**2>=0.000
      43 -> 50        -0.28775
      44 -> 50         0.14616
      46 -> 50         0.26065
      47 -> 54         0.29210
      48 -> 53        -0.19470
      48 -> 55         0.26199
      48 -> 56         0.18548
      48 -> 59         0.12880

 Excited State  21: Singlet-A 7.2003 eV  172.19 nm  f=0.1664  <S**2>=0.000
      40 -> 50         0.10565
      43 -> 49         0.17357
      43 -> 51        -0.11065
      44 -> 49        -0.15576
      45 -> 50        -0.18333
      46 -> 51         0.25745
      46 -> 52        -0.18561
      47 -> 51        -0.11046
      47 -> 52         0.42852
      48 -> 52        -0.21486
```

```
 Excited State  22: Singlet-A 7.2882 eV  170.12 nm  f=0.0000  <S**2>=0.000
      47 -> 54        -0.12537
      47 -> 55         0.31420
      47 -> 56        -0.10110
      48 -> 55         0.44374
      48 -> 56        -0.28409
      48 -> 57         0.15439
      48 -> 59        -0.16059

 Excited State  23: Singlet-A  7.4505 eV  166.41 nm  f=0.0296  <S**2>=0.000
      46 -> 53        -0.15859
      46 -> 54         0.10505
      46 -> 55         0.49436
      46 -> 56        -0.19819
      47 -> 55         0.24849
      48 -> 54        -0.13620
      48 -> 56         0.13575

 Excited State  24: Singlet-A 7.4863 eV  165.62 nm  f=0.0137  <S**2>=0.000
      43 -> 49        -0.13568
      44 -> 49         0.33063
      46 -> 52        -0.10261
      47 -> 52         0.14408
      47 -> 58        -0.29748
      47 -> 62        -0.10772
      48 -> 58         0.40161
      48 -> 62         0.15282

 Excited State  25: Singlet-A 7.5718 eV  163.75 nm  f=0.0007  <S**2>=0.000
      40 -> 49         0.15037
      42 -> 49         0.15936
      42 -> 51         0.21385
      45 -> 51        -0.11499
      46 -> 54         0.10281
      46 -> 55         0.24858
      47 -> 55        -0.24525
      47 -> 57        -0.11977
      47 -> 59         0.14966
      48 -> 57         0.31997
      48 -> 59        -0.14808
**********************************************************************
```

10. TD-DFT calculation of 1B

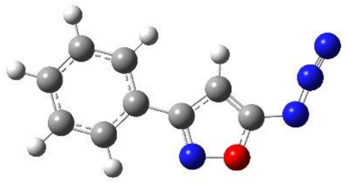

```
Excitation energies and oscillator strengths:

 Excited State   1: Singlet-A  4.0688 eV  304.72 nm  f=0.0004  <S**2>=0.000
      42 -> 49        -0.22731
      46 -> 49        -0.17041
      47 -> 49         0.47635
```

```
      48 -> 49         -0.42241
 This state for optimization and/or second-order correction.
 Total Energy, E(TD-HF/TD-DFT) =  -640.237465931
 Copying the excited state density for this state as the 1-particle RhoCI
density.

 Excited State   2: Singlet-A 5.1540 eV  240.56 nm  f=0.2899  <S**2>=0.000
      43 -> 49          0.19211
      46 -> 50          0.14671
      47 -> 50         -0.42141
      47 -> 51          0.15519
      48 -> 50          0.43201
      48 -> 51         -0.15045

 Excited State   3:  Singlet-A  5.1904 eV  238.87 nm  f=0.0058  <S**2>=0.000
      46 -> 50          0.28374
      46 -> 51          0.38556
      47 -> 50          0.23768
      47 -> 51          0.10254
      47 -> 53          0.21258
      48 -> 51         -0.13910
      48 -> 53          0.34510

 Excited State   4: Singlet-A  5.3753 eV  230.66 nm  f=0.3792  <S**2>=0.000
      46 -> 50         -0.13999
      46 -> 53         -0.12657
      47 -> 50          0.27912
      47 -> 51          0.17761
      48 -> 50          0.40580
      48 -> 51          0.41156

 Excited State   5: Singlet-A 5.7399 eV  216.00 nm  f=0.0061  <S**2>=0.000
      44 -> 50          0.14254
      46 -> 50          0.14297
      46 -> 51          0.19853
      47 -> 50         -0.33039
      47 -> 51         -0.22329
      47 -> 60          0.12203
      48 -> 51          0.43856
      48 -> 53          0.11449

 Excited State   6: Singlet-A 6.0581 eV  204.66 nm  f=0.0000  <S**2>=0.000
      44 -> 49         -0.16547
      47 -> 49          0.45721
      48 -> 49          0.50906

 Excited State   7: Singlet-A  6.1163 eV  202.71 nm  f=0.0203  <S**2>=0.000
      43 -> 49          0.11472
      46 -> 53         -0.13066
      47 -> 51          0.52439
      48 -> 50         -0.31366
      48 -> 51          0.15733
      48 -> 53         -0.11347

 Excited State   8: Singlet-A  6.2440 eV  198.56 nm  f=0.0107  <S**2>=0.000
      46 -> 52          0.12529
      47 -> 52         -0.40337
```

```
     47 -> 54        -0.19442
     48 -> 52         0.44847
     48 -> 54         0.15983

Excited State   9: Singlet-A   6.3404 eV  195.55 nm  f=0.0033  <S**2>=0.000
     45 -> 50         0.61920
     45 -> 51         0.22483
     45 -> 60        -0.16430

Excited State  10: Singlet-A  6.5126 eV  190.37 nm  f=0.3488  <S**2>=0.000
     46 -> 50         0.47573
     46 -> 53         0.25434
     47 -> 50         0.17371
     48 -> 51         0.11255
     48 -> 53        -0.36425

Excited State  11: Singlet-A  6.6336 eV  186.90 nm  f=0.0049  <S**2>=0.000
     47 -> 52         0.37062
     47 -> 54        -0.12230
     48 -> 52         0.41619
     48 -> 54        -0.34625

Excited State  12: Singlet-A  6.6766 eV  185.70 nm  f=0.0023  <S**2>=0.000
     40 -> 50         0.18094
     40 -> 51        -0.11702
     42 -> 49         0.30428
     43 -> 50         0.40323
     43 -> 51        -0.22873
     44 -> 49        -0.18967
     45 -> 51         0.13213
     46 -> 49         0.11171
     47 -> 49         0.12081
     48 -> 49        -0.16045

Excited State  13: Singlet-A  6.7272 eV  184.30 nm  f=0.6164  <S**2>=0.000
     43 -> 49         0.16535
     46 -> 51        -0.31668
     46 -> 53         0.33359
     47 -> 53         0.30117
     48 -> 51         0.17321
     48 -> 53         0.26331

Excited State  14: Singlet-A 6.7965 eV  182.42 nm  f=0.0134  <S**2>=0.000
     40 -> 49         0.14443
     42 -> 50         0.21488
     42 -> 51        -0.12684
     43 -> 49         0.30553
     44 -> 51         0.16789
     45 -> 49        -0.11020
     46 -> 51         0.16032
     46 -> 53        -0.23001
     47 -> 51        -0.23959
     47 -> 53         0.14180
     47 -> 58        -0.12447
     48 -> 53        -0.16054
     48 -> 58         0.13659
     48 -> 60         0.11568
```

```
Excited State  15:   Singlet-A 6.8197 eV  181.80 nm  f=0.0016  <S**2>=0.000
     40 -> 50         -0.11597
     42 -> 49          0.20042
     43 -> 50         -0.26627
     43 -> 51          0.14594
     44 -> 49         -0.13729
     46 -> 49          0.48719
     46 -> 52          0.11702
     47 -> 49          0.15733
     48 -> 49         -0.13663

Excited State  16: Singlet-A   6.8960 eV  179.79 nm  f=0.0010  <S**2>=0.000
     42 -> 49         -0.23513
     43 -> 50          0.13543
     44 -> 49          0.15623
     46 -> 52          0.39029
     46 -> 54         -0.34428
     47 -> 52          0.17795
     48 -> 54          0.17398

Excited State  17: Singlet-A  6.9298 eV  178.92 nm  f=0.0007  <S**2>=0.000
     42 -> 49         -0.30848
     43 -> 50          0.15108
     44 -> 49          0.20184
     46 -> 49          0.45602
     46 -> 52         -0.16811
     46 -> 54          0.17316
     47 -> 52         -0.13368
     48 -> 54         -0.10971

Excited State  18: Singlet-A  6.9521 eV  178.34 nm  f=0.1770  <S**2>=0.000
     46 -> 50         -0.31461
     46 -> 51          0.34725
     46 -> 53          0.33069
     46 -> 58          0.10916
     47 -> 51          0.11342
     47 -> 53          0.20386
     48 -> 53         -0.24841

Excited State  19: Singlet-A   7.0635 eV  175.53 nm  f=0.0014  <S**2>=0.000
     44 -> 52         -0.10628
     46 -> 52         -0.18533
     46 -> 54          0.13547
     47 -> 52          0.21524
     47 -> 54          0.29270
     47 -> 55         -0.16761
     48 -> 52          0.16799
     48 -> 54          0.41703
     48 -> 57          0.16170

Excited State  20: Singlet-A  7.1916 eV  172.40 nm  f=0.0035  <S**2>=0.000
     47 -> 54         -0.20474
     47 -> 55         -0.12960
     47 -> 56         -0.13785
     47 -> 57         -0.12625
     48 -> 52         -0.18257
```

```
      48 -> 55          0.52087
      48 -> 56          0.10315
      48 -> 57          0.12638

 Excited State   21: Singlet-A    7.1928 eV  172.37 nm  f=0.0128  <S**2>=0.000
      42 -> 50         -0.15804
      42 -> 51          0.10794
      43 -> 49         -0.14464
      44 -> 50          0.19870
      46 -> 51         -0.18085
      46 -> 53         -0.23973
      47 -> 53          0.47908
      48 -> 53         -0.20162

 Excited State   22: Singlet-A    7.2908 eV  170.06 nm  f=0.0018  <S**2>=0.000
      46 -> 54          0.13433
      47 -> 52          0.11954
      47 -> 54         -0.20615
      47 -> 55         -0.26592
      48 -> 55         -0.26669
      48 -> 56          0.37075
      48 -> 57         -0.21277
      48 -> 59          0.18638

 Excited State   23: Singlet-A    7.4400 eV  166.65 nm  f=0.0253  <S**2>=0.000
      46 -> 52         -0.26096
      46 -> 54         -0.25218
      46 -> 55          0.46994
      46 -> 57         -0.11231
      47 -> 55          0.25537
      48 -> 54          0.11516

 Excited State   24: Singlet-A 7.4975 eV  165.37 nm  f=0.0010  <S**2>=0.000
      41 -> 50          0.30511
      41 -> 51          0.31702
      45 -> 50         -0.14422
      45 -> 51          0.48050

 Excited State   25: Singlet-A  7.5239 eV  164.79 nm  f=0.0151  <S**2>=0.000
      42 -> 50         -0.11886
      44 -> 50          0.25345
      46 -> 53          0.12842
      46 -> 58          0.11570
      47 -> 53         -0.18666
      47 -> 58         -0.31435
      47 -> 62         -0.12929
      48 -> 58          0.43781
**********************************************************************
```

11. TD-DFT calculation of nitrene [1]2

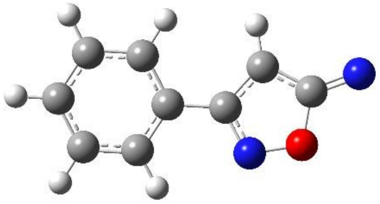

```
Excitation energies and oscillator strengths:

 Excited State   1:  1.672-A -0.7671 eV    -1616.32 nm  f=-0.0000
<S**2>=0.449
      39A -> 42A       -0.16825
      40A -> 42A        0.54809
      41A -> 42A        0.41176
      39B -> 42B        0.21022
      40B -> 42B       -0.54500
      41B -> 42B       -0.40320
      40A <- 42A        0.11514
      40B <- 42B       -0.11010
 This state for optimization and/or second-order correction.
 Total Energy, E(TD-HF/TD-DFT) =  -530.910071725
 Copying the excited state density for this state as the 1-particle RhoCI
density.

 Excited State   2:  1.741-A 1.5130 eV  819.44 nm  f=0.0126  <S**2>=0.508
      36A -> 42A       -0.15678
      39A -> 42A       -0.16540
      40A -> 42A        0.52217
      41A -> 42A        0.38866
      36B -> 42B       -0.15838
      38B -> 42B        0.10358
      39B -> 42B       -0.19726
      40B -> 42B        0.52164
      41B -> 42B        0.36704

 Excited State   3:  2.736-A   2.5336 eV  489.35 nm  f=0.0001  <S**2>=1.621
      36A -> 42A       -0.16591
      37A -> 42A        0.22205
      38A -> 42A       -0.15425
      40A -> 42A        0.32340
      41A -> 42A       -0.48193
      41A -> 43A        0.19256
      36B -> 42B        0.14728
      37B -> 42B       -0.26399
      40B -> 42B       -0.30999
      41B -> 42B        0.49227
      41B -> 43B       -0.19243

 Excited State   4:  2.596-A   2.9329 eV  422.74 nm  f=0.0001  <S**2>=1.435
      34A -> 42A        0.17760
      36A -> 42A       -0.40362
      37A -> 42A       -0.34723
      38A -> 42A       -0.28523
      40A -> 42A       -0.16735
      41A -> 43A        0.18638
      34B -> 42B        0.15173
      35B -> 42B       -0.13417
      36B -> 42B        0.41550
      37B -> 42B        0.23376
      38B -> 42B       -0.36972
      40B -> 42B        0.15180
      41B -> 43B       -0.18542

 Excited State   5:  2.845-A   3.3516 eV  369.92 nm  f=0.0048  <S**2>=1.773
```

```
     37A -> 42A        -0.15087
     39A -> 44A         0.25454
     40A -> 42A        -0.24401
     40A -> 43A         0.21771
     41A -> 42A         0.29677
     41A -> 43A        -0.31013
     36B -> 42B         0.11591
     37B -> 42B        -0.11127
     38B -> 42B        -0.11058
     39B -> 44B         0.24692
     40B -> 42B        -0.28557
     40B -> 43B        -0.15091
     41B -> 42B         0.46030
     41B -> 43B         0.35106

 Excited State   6:  2.735-A  3.3774 eV  367.10 nm  f=0.0055  <S**2>=1.620
     36A -> 42A         0.12136
     38A -> 42A         0.14328
     39A -> 44A        -0.23561
     40A -> 42A        -0.30573
     40A -> 43A        -0.13624
     41A -> 42A         0.47463
     41A -> 43A         0.32773
     37B -> 42B        -0.14113
     39B -> 44B        -0.23306
     40B -> 42B        -0.26008
     40B -> 43B         0.21042
     41B -> 42B         0.33310
     41B -> 43B        -0.27964

 Excited State   7:  2.686-A 3.7732 eV  328.59 nm  f=0.0131  <S**2>=1.554
     33A -> 42A         0.26262
     34A -> 42A        -0.15400
     35A -> 42A         0.23436
     36A -> 42A         0.20444
     38A -> 42A        -0.47744
     38A -> 43A         0.11581
     33B -> 42B        -0.27108
     34B -> 42B         0.15143
     35B -> 42B        -0.25398
     36B -> 42B         0.16454
     38B -> 42B         0.48672
     38B -> 43B        -0.11685

 Excited State   8:  1.989-A  4.1237 eV  300.66 nm  f=0.0074  <S**2>=0.739
     33A -> 42A        -0.35948
     34A -> 42A         0.13669
     35A -> 42A        -0.24265
     36A -> 42A        -0.20901
     38A -> 42A         0.41552
     39A -> 42A         0.23876
     33B -> 42B        -0.38634
     34B -> 42B         0.13381
     35B -> 42B        -0.26751
     36B -> 42B         0.17865
     37B -> 42B         0.12135
     38B -> 42B         0.38999
```

```
 Excited State   9:  2.747-A  4.1782 eV  296.74 nm  f=0.0002  <S**2>=1.637
     33A -> 42A         0.14036
     39A -> 42A         0.62796
     39A -> 44A         0.11878
     40A -> 42A         0.14435
     41A -> 43A         0.14519
     39B -> 42B        -0.62666
     39B -> 44B         0.11498
     40B -> 42B        -0.18089
     41B -> 42B        -0.10332
     41B -> 43B        -0.14200

 Excited State  10:  1.969-A 4.2106 eV  294.46 nm  f=0.0055  <S**2>=0.719
     39A -> 42A         0.63463
     40A -> 42A         0.17272
     33B -> 42B         0.12815
     39B -> 42B         0.66732
     40B -> 42B         0.20772

Excited State  11:  2.649-A  4.2714 eV  290.27 nm  f=0.0121  <S**2>=1.504
     33A -> 42A 0.57550
     38A -> 42A         0.26229
     38A -> 43A        -0.12256
     33B -> 42B        -0.57451
     38B -> 42B        -0.27106
     38B -> 43B         0.11876

 Excited State  12:  3.472-A   4.5104 eV  274.89 nm  f=0.0001  <S**2>=2.764
     33A -> 42A         0.12816
     39A -> 43A         0.54705
     39A -> 44A         0.17801
     39A -> 51A         0.11800
     40A -> 43A         0.16465
     40A -> 44A         0.15327
     41A -> 43A         0.17938
     41A -> 44A        -0.17399
     33B -> 42B         0.10804
     39B -> 43B        -0.53957
     39B -> 44B         0.17348
     39B -> 51B        -0.11608
     40B -> 43B        -0.19896
     40B -> 44B         0.16600
     41B -> 43B        -0.19168
     41B -> 44B        -0.16482

 Excited State  13:  2.982-A   4.5622 eV  271.76 nm  f=0.0002  <S**2>=1.973
     33A -> 42A         0.30093
     38A -> 42A         0.19205
     39A -> 42A        -0.20588
     39A -> 43A        -0.22403
     39A -> 44A         0.36640
     40A -> 42A        -0.13144
     41A -> 43A         0.26697
     41A -> 44A         0.16559
     33B -> 42B         0.26702
     38B -> 42B         0.19839
```

```
     39B -> 42B        0.13918
     39B -> 43B        0.23201
     39B -> 44B        0.36564
     40B -> 42B        0.11729
     41B -> 43B       -0.22974
     41B -> 44B        0.17040

 Excited State  14:  2.625-A  4.6427 eV  267.05 nm  f=0.0002  <S**2>=1.473
     33A -> 42A        0.40464
     34A -> 42A        0.10117
     36A -> 42A       -0.11163
     38A -> 42A        0.29667
     39A -> 42A        0.11303
     39A -> 44A       -0.32081
     40A -> 43A        0.16307
     41A -> 43A       -0.14464
     33B -> 42B        0.42376
     36B -> 42B        0.11121
     38B -> 42B        0.30457
     39B -> 42B       -0.13063
     39B -> 44B       -0.30937
     40B -> 43B       -0.16762
     41B -> 43B        0.15855

 Excited State  15:  2.105-A    4.8487 eV  255.71 nm  f=0.1490  <S**2>=0.858
     34A -> 42A       -0.19004
     36A -> 42A        0.38712
     37A -> 42A        0.50057
     38A -> 42A        0.13865
     41A -> 42A        0.21175
     34B -> 42B        0.18383
     35B -> 42B       -0.15643
     36B -> 42B        0.43873
     37B -> 42B        0.13364
     38B -> 42B       -0.27299
     39B -> 42B       -0.10813
     40B -> 42B        0.14575

 Excited State  16:  2.865-A 4.8601 eV  255.11 nm  f=0.0199  <S**2>=1.803
     32A -> 42A       -0.13623
     36A -> 42A        0.21482
     37A -> 42A       -0.25721
     37A -> 43A        0.10355
     38A -> 42A        0.21812
     39A -> 44A        0.13449
     40A -> 42A        0.18449
     40A -> 43A       -0.23830
     40A -> 44A        0.14866
     41A -> 42A       -0.16073
     41A -> 44A       -0.16702
     32B -> 42B        0.13520
     33B -> 42B        0.12710
     36B -> 42B        0.11512
     37B -> 42B        0.50321
     37B -> 43B       -0.10278
     39B -> 44B        0.12010
     40B -> 42B       -0.11258
```

```
    40B -> 43B         0.24075
    40B -> 44B         0.17280
    41B -> 42B         0.26277
    41B -> 44B        -0.17312

Excited State  17:  3.376-A  5.0516 eV  245.43 nm  f=0.0000  <S**2>=2.599
    37A -> 42A        -0.14797
    37A -> 44A         0.10267
    39A -> 43A         0.25362
    40A -> 44A        -0.26525
    41A -> 42A        -0.11383
    41A -> 44A         0.50800
    37B -> 42B         0.16698
    37B -> 44B         0.10845
    39B -> 43B        -0.25576
    40B -> 44B        -0.26693
    41B -> 42B         0.11469
    41B -> 44B         0.50547

Excited State  18:  2.160-A  5.2218 eV  237.44 nm  f=0.0072  <S**2>=0.917
    36A -> 42A        -0.12590
    37A -> 42A         0.15998
    39A -> 43A         0.52815
    40A -> 44A        -0.16576
    41A -> 44A         0.33996
    39B -> 43B         0.46260
    40B -> 43B         0.24760
    40B -> 44B         0.19786
    41B -> 44B        -0.37458

Excited State  19:  2.630-A  5.2340 eV  236.88 nm  f=0.0057  <S**2>=1.479
    36A -> 42A 0.12791
    37A -> 42A        -0.12882
    40A -> 43A         0.37520
    41A -> 44A         0.11746
    41A -> 51A        -0.12665
    26B -> 42B         0.11276
    32B -> 42B         0.17510
    33B -> 42B        -0.11068
    34B -> 42B        -0.11153
    35B -> 42B         0.11879
    36B -> 42B        -0.25995
    36B -> 43B        -0.10841
    37B -> 42B         0.39720
    39B -> 43B         0.21958
    40B -> 42B        -0.11960
    40B -> 43B        -0.37491
    41B -> 43B        -0.25262
    41B -> 51B         0.14867

Excited State  20:  2.138-A 5.2962 eV  234.10 nm  f=0.1380  <S**2>=0.893
    32A -> 42A         0.19958
    34A -> 42A         0.10950
    36A -> 42A        -0.25572
    37A -> 42A         0.45772
    40A -> 42A        -0.12478
    40A -> 43A        -0.12551
```

```
     41A -> 42A         0.11831
     41A -> 43A        -0.45023
     32B -> 42B         0.12211
     36B -> 42B        -0.12791
     37B -> 42B         0.34104
     39B -> 43B        -0.13625
     41B -> 43B        -0.38024

 Excited State  21:  2.102-A  5.5204 eV  224.59 nm  f=0.1960  <S**2>=0.855
     32A -> 42A         0.10977
     36A -> 42A        -0.16299
     37A -> 42A         0.28942
     38A -> 42A        -0.11669
     39A -> 44A        -0.14335
     40A -> 43A        -0.31959
     41A -> 43A         0.46625
     36B -> 42B        -0.12483
     37B -> 42B         0.29362
     39B -> 44B         0.14332
     40B -> 43B        -0.28335
     41B -> 43B         0.48032

 Excited State  22:  2.459-A  5.8530 eV  211.83 nm  f=0.1241  <S**2>=1.262
     37A -> 42A         0.13529
     38A -> 42A        -0.24137
     39A -> 43A        -0.13254
     40A -> 43A         0.55645
     40A -> 51A        -0.11363
     41A -> 43A         0.19719
     37B -> 42B         0.21196
     38B -> 42B         0.19262
     39B -> 43B        -0.16902
     40B -> 43B         0.52372
     40B -> 51B        -0.10235
     41B -> 43B         0.18736

 Excited State  23:  3.326-A   5.9417 eV  208.67 nm  f=0.0001  <S**2>=2.516
     37A -> 43A        -0.44476
     38A -> 43A         0.18521
     40A -> 43A        -0.24247
     40A -> 51A         0.16102
     41A -> 50A         0.12968
     41A -> 51A        -0.23663
     37B -> 43B         0.47518
     40B -> 43B         0.25064
     40B -> 51B        -0.16003
     41B -> 50B         0.10723
     41B -> 51B         0.22365

 Excited State  24:  2.707-A  6.1535 eV  201.49 nm  f=0.0083  <S**2>=1.582
     29A -> 42A        -0.20148
     31A -> 42A        -0.51581
     33A -> 42A        -0.10240
     34A -> 42A         0.28949
     35A -> 42A         0.17729
     29B -> 42B         0.20621
     31B -> 42B         0.53654
```

```
     33B -> 42B        0.10294
     34B -> 42B       -0.30082
     35B -> 42B       -0.19542

 Excited State  25:  1.976-A  6.3078 eV  196.56 nm  f=0.0001  <S**2>=0.727
     29A -> 42A        0.18704
     31A -> 42A        0.52020
     33A -> 42A        0.10203
     34A -> 42A       -0.34742
     35A -> 42A       -0.23385
     29B -> 42B        0.17805
     31B -> 42B        0.50659
     34B -> 42B       -0.33420
     35B -> 42B       -0.23744
*************************************************************************
```

12. TD-DFT calculation nitrene $^{3}2$

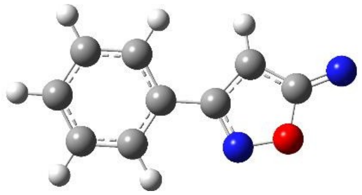

```
Excitation energies and oscillator strengths:

 Excited State   1:  3.127-A  2.9137 eV  425.52 nm  f=0.0082  <S**2>=2.195
     42A -> 43A       -0.27189
     36B -> 41B       -0.33122
     40B -> 41B        0.86470
     40B -> 43B       -0.15008
 This state for optimization and/or second-order correction.
 Total Energy, E(TD-HF/TD-DFT) =  -530.797349292
 Copying the excited state density for this state as the 1-particle RhoCI
density.

 Excited State   2:  4.031-A   3.3148 eV  374.03 nm  f=0.0027  <S**2>=3.812
     40A -> 44A        0.31692
     41A -> 43A        0.39908
     41A -> 44A        0.12347
     42A -> 43A        0.36691
     38B -> 41B       -0.22429
     39B -> 41B        0.11018
     39B -> 44B       -0.35018
     40B -> 41B        0.21717
     40B -> 43B        0.53361
     39B <- 44B       -0.10029
     40B <- 43B        0.10571

 Excited State   3:  3.038-A  3.5071 eV  353.53 nm  f=0.0002  <S**2>=2.057
     26B -> 42B       -0.11031
     36B -> 42B       -0.29257
     38B -> 42B        0.92042
     40B -> 42B        0.17485

 Excited State   4:  3.126-A  3.7547 eV  330.21 nm  f=0.0574  <S**2>=2.194
```

```
    40A -> 44A        0.15123
    41A -> 43A        0.17854
    42A -> 43A       -0.10030
    42A -> 48A        0.17107
    36B -> 41B       -0.14813
    38B -> 41B        0.86785
    39B -> 44B       -0.13817
    40B -> 43B        0.11218

Excited State   5:  3.178-A 3.8018 eV  326.12 nm  f=0.0031  <S**2>=2.275
    35B -> 41B        0.16998
    37B -> 41B        0.91378
    37B -> 43B       -0.20484
    37B -> 52B       -0.12013

Excited State   6:  3.057-A  3.9271 eV  315.71 nm  f=0.0052  <S**2>=2.086
    33B -> 42B        0.80007
    34B -> 42B        0.11312
    35B -> 42B       -0.32550
    37B -> 42B       -0.32890
    40B -> 42B       -0.26564

Excited State   7:  3.075-A 3.9865 eV  311.01 nm  f=0.0008  <S**2>=2.114
    33B -> 42B        0.23880
    36B -> 42B       -0.28378
    38B -> 42B       -0.25485
    40B -> 42B        0.87645

Excited State   8:  3.357-A 4.3552 eV  284.68 nm  f=0.0030  <S**2>=2.568
    40A -> 44A        0.20422
    41A -> 43A       -0.12920
    42A -> 43A       -0.23814
    39B -> 41B        0.86579
    39B -> 44B       -0.24937
    40B -> 41B       -0.11180
    40B -> 43B       -0.16894

Excited State   9:  4.099-A  4.4860 eV  276.38 nm  f=0.0002  <S**2>=3.950
    40A -> 43A        0.67820
    40A -> 51A       -0.10801
    41A -> 43A        0.25600
    41A -> 44A        0.13170
    42A -> 43A       -0.13827
    42A -> 44A        0.11182
    39B -> 43B        0.49973
    40B -> 44B       -0.31747

Excited State  10:  3.824-A  4.6341 eV  267.55 nm  f=0.0015  <S**2>=3.406
    40A -> 44A       -0.47145
    41A -> 43A        0.10723
    41A -> 44A       -0.10674
    42A -> 43A        0.29659
    42A -> 44A        0.15792
    38B -> 41B        0.16939
    39B -> 41B        0.46769
    39B -> 44B        0.48037
    40B -> 41B        0.12231
```

```
    40B -> 43B        0.30019

 Excited State  11:  3.225-A  4.8248 eV  256.97 nm  f=0.0054  <S**2>=2.350
    40A -> 44A       -0.10626
    41A -> 43A        0.26271
    41A -> 44A       -0.11304
    42A -> 43A       -0.23286
    33B -> 41B        0.70742
    34B -> 41B        0.13680
    35B -> 41B       -0.24702
    36B -> 41B       -0.24218
    38B -> 41B       -0.13479
    39B -> 44B        0.12108
    40B -> 41B       -0.19668
    40B -> 43B        0.12454
    40B -> 44B        0.13779

 Excited State  12:  3.328-A  4.8737 eV  254.39 nm  f=0.0074  <S**2>=2.520
    41A -> 43A       -0.35640
    41A -> 44A        0.17353
    42A -> 43A        0.35670
    42A -> 48A       -0.11476
    32B -> 41B        0.12235
    33B -> 41B        0.52353
    34B -> 41B        0.10643
    35B -> 41B       -0.19234
    36B -> 41B        0.22026
    37B -> 42B        0.10330
    38B -> 41B        0.17617
    39B -> 44B       -0.12514
    40B -> 41B        0.25250
    40B -> 43B       -0.14044
    40B -> 44B       -0.23923

 Excited State  13:  3.974-A 5.0429 eV  245.86 nm  f=0.0082  <S**2>=3.697
    40A -> 43A        0.30104
    41A -> 44A       -0.38717
    42A -> 43A        0.21865
    42A -> 44A       -0.33117
    36B -> 44B        0.12251
    37B -> 42B        0.11096
    39B -> 43B        0.28077
    40B -> 41B        0.13324
    40B -> 44B        0.62787

 Excited State  14:  3.128-A 5.2512 eV  236.11 nm  f=0.0033  <S**2>=2.197
    40A -> 43A       -0.37473
    41A -> 43A       -0.14084
    41A -> 44A        0.39193
    42A -> 43A        0.13431
    42A -> 44A        0.32133
    36B -> 41B       -0.11718
    39B -> 42B        0.27054
    39B -> 43B        0.60224
    40B -> 44B        0.26193

 Excited State  15:  3.094-A 5.2993 eV  233.96 nm  f=0.0001  <S**2>=2.143
```

```
     41A -> 44A       -0.10356
     36B -> 41B        0.11967
     39B -> 42B        0.94722
     39B -> 43B       -0.17247

 Excited State  16:  3.124-A 5.4123 eV  229.08 nm  f=0.0489  <S**2>=2.189
     40A -> 43A       -0.19854
     41A -> 43A        0.27737
     42A -> 43A       -0.14044
     32B -> 41B        0.22683
     33B -> 42B       -0.17064
     36B -> 41B        0.66527
     37B -> 42B       -0.37703
     39B -> 42B       -0.13450
     39B -> 43B        0.11323
     40B -> 41B        0.19773
     40B -> 43B       -0.15157
     40B -> 44B        0.16771

 Excited State  17:  3.081-A  5.4768 eV  226.38 nm  f=0.3740  <S**2>=2.122
     40A -> 44A       -0.13035
     41A -> 43A       -0.49898
     42A -> 43A       -0.40882
     36B -> 41B        0.15275
     37B -> 42B       -0.11490
     39B -> 44B       -0.17875
     40B -> 43B        0.65684

 Excited State  18:  3.146-A  5.7517 eV  215.56 nm  f=0.0446  <S**2>=2.224
     40A -> 43A       -0.12719
     41A -> 43A        0.18258
     42A -> 43A       -0.27558
     42A -> 48A       -0.15098
     33B -> 42B        0.28052
     36B -> 41B        0.27136
     37B -> 42B        0.74263
     38B -> 43B       -0.12617

 Excited State  19:  4.025-A  5.9658 eV  207.82 nm  f=0.0009  <S**2>=3.800
     38A -> 43A 0.51652
     41A -> 43A       -0.19013
     41A -> 48A        0.19856
     41A -> 51A       -0.15513
     41A -> 53A       -0.10798
     42A -> 48A        0.14172
     42A -> 51A       -0.11034
     36B -> 41B        0.18898
     36B -> 43B        0.44330
     37B -> 42B        0.11662
     38B -> 43B        0.12505
     40B -> 51B       -0.18991
     40B -> 52B       -0.30543
     40B -> 53B        0.12871

 Excited State  20:  3.605-A  6.1542 eV  201.46 nm  f=0.0009  <S**2>=2.999
     35A -> 43A       -0.15347
     37A -> 43A       -0.28410
```

```
      39A -> 43A         0.78448
      39A -> 48A        -0.25293
      39A -> 49A         0.11400
      39A -> 51A         0.14484
      31B -> 41B         0.13695
      37B -> 41B         0.19200
      37B -> 43B         0.17968

 Excited State  21:  3.106-A   6.2359 eV  198.82 nm  f=0.0000  <S**2>=2.162
      26B -> 42B         0.12418
      32B -> 42B         0.24751
      36B -> 42B         0.84347
      38B -> 42B         0.23728
      40B -> 42B         0.34751

 Excited State  22:  3.108-A  6.3193 eV  196.20 nm  f=0.0000  <S**2>=2.165
      39A -> 43A        -0.13314
      29B -> 41B        -0.18071
      31B -> 41B         0.75253
      33B -> 41B         0.19546
      34B -> 41B        -0.51147
      35B -> 41B         0.17478

 Excited State  23:  3.123-A  6.5554 eV  189.13 nm  f=0.2313  <S**2>=2.188
      40A -> 43A         0.29783
      41A -> 43A         0.11219
      41A -> 44A         0.13764
      41A -> 48A        -0.12560
      42A -> 44A         0.54951
      42A -> 45A        -0.11161
      42A -> 48A         0.17698
      42A -> 49A        -0.11297
      42A -> 53A         0.12231
      26B -> 41B        -0.10525
      38B -> 43B         0.14496
      39B -> 43B        -0.35379
      40B -> 44B         0.40758

 Excited State  24:  3.107-A  6.6032 eV  187.76 nm  f=0.0102  <S**2>=2.163
      40A -> 45A         0.10272
      40A -> 46A        -0.15685
      41A -> 44A         0.12310
      41A -> 45A        -0.35643
      41A -> 46A         0.26433
      41A -> 47A        -0.11335
      42A -> 45A         0.60937
      42A -> 46A        -0.36536
      42A -> 47A         0.28395
      42A -> 48A         0.11770
      42A -> 49A         0.14565
      40B -> 44B         0.10283

 Excited State  25:  3.706-A  6.6315 eV  186.96 nm  f=0.0303  <S**2>=3.183
      41A -> 44A         0.10171
      42A -> 44A        -0.10175
      36B -> 46B         0.10188
      40B -> 45B         0.89588
```

```
     40B -> 46B         0.16458
     40B -> 51B        -0.14211
```

****

13. TD-DFT calculation nitrosoalkene [3]3A

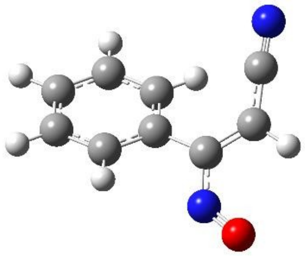

```
Excitation energies and oscillator strengths:

 Excited State   1:  3.031-A  2.5047 eV  495.01 nm  f=0.0004  <S**2>=2.047
     32B -> 41B         0.10736
     33B -> 41B        -0.15122
     35B -> 41B         0.10159
     38B -> 41B         0.45562
     40B -> 41B         0.84860
 This state for optimization and/or second-order correction.
 Total Energy, E(TD-HF/TD-DFT) =  -530.819971079
 Copying the excited state density for this state as the 1-particle RhoCI
density.

 Excited State   2:  3.166-A  2.7888 eV  444.57 nm  f=0.0318  <S**2>=2.255
     41A -> 43A         0.14061
     42A -> 43A         0.32070
     42A -> 47A         0.13668
     38B -> 42B         0.34269
     40B -> 42B         0.82998

 Excited State   3:  4.025-A  3.4634 eV  357.98 nm  f=0.0015  <S**2>=3.800
     40A -> 44A         0.39195
     40A -> 45A        -0.10222
     41A -> 43A         0.48036
     41A -> 47A        -0.18316
     42A -> 43A        -0.19334
     38B -> 42B        -0.19544
     38B -> 43B        -0.13474
     39B -> 41B         0.13889
     39B -> 44B        -0.38455
     39B -> 45B         0.10723
     40B -> 43B         0.46753
     40A <- 44A         0.10214

 Excited State   4:  3.266-A 4.1288 eV  300.29 nm  f=0.0019  <S**2>=2.416
     40A -> 43A        -0.19144
     41A -> 43A        -0.12412
     38B -> 41B         0.17675
     39B -> 41B         0.79338
     39B -> 42B        -0.42772
     39B -> 43B        -0.17939
```

```
    40B -> 41B       -0.10203

Excited State   5:  3.301-A  4.2165 eV  294.04 nm  f=0.0010  <S**2>=2.474
    40A -> 43A        0.22376
    41A -> 43A       -0.12046
    32B -> 41B        0.11438
    38B -> 41B        0.22904
    39B -> 41B        0.41187
    39B -> 42B        0.76804
    39B -> 43B        0.20062
    40B -> 41B       -0.14229

Excited State   6:  3.171-A 4.2818 eV  289.56 nm  f=0.0034  <S**2>=2.263
    40A -> 44A       -0.14566
    42A -> 43A       -0.11233
    26B -> 41B        0.12445
    28B -> 41B       -0.13716
    32B -> 41B        0.24800
    38B -> 41B        0.67286
    39B -> 41B       -0.31967
    39B -> 42B       -0.11334
    39B -> 44B        0.13885
    40B -> 41B       -0.46274
    40B -> 44B        0.10132

Excited State   7:  3.175-A  4.4273 eV  280.04 nm  f=0.0409  <S**2>=2.271
    40A -> 44A        0.19572
    42A -> 43A        0.55142
    42A -> 47A        0.15891
    32B -> 42B        0.10793
    38B -> 42B        0.47403
    39B -> 41B       -0.17567
    39B -> 44B       -0.17155
    40B -> 42B       -0.50613

Excited State   8:  3.769-A   4.6402 eV  267.20 nm  f=0.0024  <S**2>=3.302
    40A -> 43A        0.66142
    40A -> 44A       -0.11045
    40A -> 47A       -0.17392
    41A -> 43A        0.14506
    41A -> 44A        0.11388
    42A -> 43A        0.11500
    37B -> 42B       -0.23065
    39B -> 42B       -0.33684
    39B -> 43B        0.32727
    40B -> 44B       -0.31019

Excited State   9:  3.955-A  4.7038 eV  263.58 nm  f=0.0178  <S**2>=3.660
    40A -> 43A       -0.15530
    40A -> 44A       -0.46193
    40A -> 45A        0.10867
    41A -> 43A        0.43813
    42A -> 43A        0.15728
    38B -> 41B       -0.10512
    39B -> 41B        0.16305
    39B -> 42B        0.13654
    39B -> 44B        0.46800
```

```
     39B -> 45B        -0.12288
     40B -> 42B        -0.12024
     40B -> 43B         0.36498

 Excited State  10:  3.236-A      4.8208 eV  257.19 nm  f=0.0203
<S**2>=2.367
     40A -> 43A         0.12307
     42A -> 43A         0.27006
     31B -> 41B        -0.12782
     35B -> 42B         0.12826
     37B -> 41B        -0.19440
     37B -> 42B         0.79473
     37B -> 49B         0.16550
     38B -> 42B        -0.19387
     39B -> 42B        -0.20572

 Excited State  11:  3.122-A    4.8829 eV  253.92 nm  f=0.1150  <S**2>=2.186
     40A -> 43A         0.11726
     42A -> 43A        -0.52274
     31B -> 41B         0.24051
     32B -> 42B         0.10698
     33B -> 42B         0.12753
     37B -> 41B         0.10281
     37B -> 42B         0.32963
     38B -> 42B         0.60067
     39B -> 42B        -0.10228

 Excited State  12:  4.079-A  5.0916 eV  243.51 nm  f=0.0010  <S**2>=3.909
     38A -> 44A         0.10359
     40A -> 43A         0.32093
     41A -> 44A        -0.59653
     41A -> 45A         0.13997
     38B -> 44B        -0.17701
     39B -> 43B         0.15851
     40B -> 44B         0.60651
     40B -> 45B        -0.14266

 Excited State  13:  3.415-A  5.1487 eV  240.81 nm  f=0.0234  <S**2>=2.666
     37A -> 52A        -0.10960
     38A -> 43A        -0.18494
     41A -> 43A         0.11939
     41A -> 47A        -0.11283
     42A -> 43A         0.12431
     29B -> 41B         0.16869
     31B -> 41B         0.48815
     32B -> 42B         0.17493
     33B -> 42B        -0.35775
     34B -> 41B         0.10058
     35B -> 41B        -0.15992
     35B -> 42B         0.22456
     37B -> 41B         0.20027
     38B -> 42B        -0.11349
     38B -> 43B        -0.22088
     38B -> 49B        -0.16475
     40B -> 43B        -0.13999
     40B -> 49B        -0.19883
```

```
Excited State  14:  3.181-A  5.4044 eV  229.41 nm  f=0.0016  <S**2>=2.279
    40A -> 43A        -0.29861
    41A -> 44A         0.43076
    39B -> 42B        -0.13946
    39B -> 43B         0.69460
    39B -> 49B        -0.13431
    40B -> 44B         0.37505

Excited State  15:  3.255-A 5.5896 eV  221.81 nm  f=0.1859  <S**2>=2.398
    38A -> 43A        -0.14850
    40A -> 44A        -0.18887
    41A -> 43A        -0.60160
    41A -> 47A        -0.14917
    38B -> 42B         0.13558
    38B -> 43B        -0.10327
    39B -> 44B        -0.11458
    40B -> 43B         0.61152
    40B -> 49B        -0.10992

Excited State  16:  3.278-A  5.6921 eV  217.82 nm  f=0.1259  <S**2>=2.437
    37A -> 52A         0.13761
    39A -> 43A         0.13148
    41A -> 43A        -0.20204
    42A -> 43A         0.12832
    26B -> 42B         0.10457
    29B -> 41B         0.14279
    31B -> 41B         0.46303
    31B -> 42B         0.18791
    32B -> 42B        -0.16922
    33B -> 41B        -0.17423
    33B -> 42B         0.29789
    35B -> 41B        -0.18261
    35B -> 42B        -0.26737
    37B -> 52B        -0.14293
    38B -> 42B        -0.28112
    40B -> 43B         0.19458

Excited State  17:  3.207-A  5.7480 eV  215.70 nm  f=0.0027  <S**2>=2.320
    38A -> 52A        -0.10711
    39A -> 43A         0.60559
    39A -> 47A         0.18447
    39A -> 48A        -0.10050
    42A -> 44A         0.10882
    42A -> 45A        -0.13114
    42A -> 46A        -0.18443
    42A -> 49A         0.15119
    42A -> 50A        -0.13328
    42A -> 51A        -0.10351
    42A -> 52A        -0.21501
    26B -> 41B        -0.11344
    28B -> 41B         0.10963
    31B -> 41B        -0.14448
    31B -> 42B         0.30486
    31B -> 43B        -0.10632
    33B -> 41B         0.11419
    33B -> 42B        -0.10784
    34B -> 42B         0.16159
```

```
    37B -> 41B         0.10708
    37B -> 42B         0.13464
    38B -> 41B         0.11038

Excited State  18:  3.350-A 5.8388 eV  212.35 nm  f=0.0005  <S**2>=2.555
    37A -> 52A        -0.12734
    38A -> 43A         0.10613
    38A -> 52A         0.13973
    39A -> 43A         0.26398
    41A -> 52A         0.11438
    42A -> 44A        -0.17341
    42A -> 45A         0.23923
    42A -> 46A         0.35513
    42A -> 49A        -0.26817
    42A -> 50A         0.19843
    42A -> 51A         0.16573
    42A -> 52A         0.34820
    31B -> 42B         0.10330
    34B -> 42B         0.13649
    37B -> 41B         0.36049
    37B -> 52B         0.16057
    38B -> 43B         0.10750

Excited State  19:  3.521-A 5.8743 eV  211.06 nm  f=0.0323  <S**2>=2.849
    37A -> 52A        -0.17084
    38A -> 43A         0.17319
    39A -> 43A        -0.34043
    39A -> 47A        -0.10502
    41A -> 47A         0.13452
    42A -> 43A         0.10443
    42A -> 44A         0.11220
    42A -> 45A        -0.10029
    42A -> 46A        -0.15206
    42A -> 49A         0.13460
    42A -> 52A        -0.17943
    33B -> 42B         0.14907
    37B -> 41B         0.51442
    37B -> 42B         0.12515
    37B -> 48B        -0.10727
    37B -> 52B         0.23379
    38B -> 42B        -0.10508
    38B -> 43B         0.15181
    40B -> 49B         0.16853

Excited State  20:  3.322-A 5.9171 eV  209.54 nm  f=0.0072  <S**2>=2.508
    38A -> 43A        -0.19347
    39A -> 43A        -0.42898
    39A -> 47A        -0.11855
    41A -> 47A        -0.12616
    31B -> 41B        -0.12121
    31B -> 42B         0.27908
    34B -> 41B        -0.10318
    34B -> 42B         0.61798
    34B -> 49B         0.12868
    35B -> 42B        -0.19386
    38B -> 43B        -0.13660
    40B -> 43B        -0.12761
```

```
     40B -> 49B       -0.15330

 Excited State  21:  3.634-A 5.9493 eV  208.40 nm  f=0.0092  <S**2>=3.051
     32A -> 43A       -0.10278
     33A -> 43A        0.10077
     38A -> 43A       -0.32772
     39A -> 43A        0.10055
     41A -> 47A       -0.23302
     41A -> 48A        0.10172
     31B -> 41B       -0.10862
     31B -> 42B       -0.13257
     33B -> 42B        0.39782
     34B -> 42B       -0.38173
     35B -> 42B       -0.19112
     37B -> 41B        0.19624
     37B -> 52B        0.10791
     38B -> 43B       -0.24658
     40B -> 43B       -0.23399
     40B -> 49B       -0.24110

 Excited State  22:  3.179-A 6.1219 eV  202.52 nm  f=0.0094  <S**2>=2.276
     39A -> 43A        0.26189
     42A -> 44A       -0.20509
     42A -> 45A       -0.18437
     42A -> 46A       -0.24085
     26B -> 41B        0.27620
     28B -> 41B       -0.28558
     31B -> 42B       -0.33565
     32B -> 41B        0.15897
     33B -> 41B       -0.18824
     33B -> 42B        0.16723
     34B -> 42B        0.38542
     34B -> 49B        0.12339
     35B -> 41B        0.14591
     35B -> 42B        0.17157
     38B -> 41B       -0.23617

 Excited State  23:  3.107-A  6.1375 eV  202.01 nm  f=0.0017  <S**2>=2.164
     39A -> 43A        0.12313
     42A -> 44A        0.64301
     42A -> 45A        0.39862
     42A -> 46A        0.38464
     42A -> 52A       -0.21268
     31B -> 42B       -0.24011
     33B -> 42B        0.12884
     34B -> 42B        0.17457
     35B -> 42B        0.10174

 Excited State  24:  3.138-A  6.1902 eV  200.29 nm  f=0.0008  <S**2>=2.212
     42A -> 44A        0.18839
     26B -> 41B        0.33695
     28B -> 41B       -0.36383
     29B -> 42B        0.10057
     30B -> 41B       -0.12299
     31B -> 42B        0.42066
     32B -> 41B        0.21059
     33B -> 41B       -0.17946
```

```
     33B -> 42B       -0.16500
     34B -> 42B       -0.24157
     35B -> 41B        0.26146
     35B -> 42B       -0.23702
     38B -> 41B       -0.28898

 Excited State  25:  3.189-A 6.5029 eV  190.66 nm  f=0.0110  <S**2>=2.292
     42A -> 43A       -0.12706
     42A -> 44A        0.58473
     42A -> 45A       -0.39304
     42A -> 46A       -0.25088
     42A -> 47A        0.22442
     42A -> 48A       -0.16048
     42A -> 49A       -0.17050
     42A -> 50A        0.15252
     42A -> 51A        0.12614
     42A -> 52A        0.31572
     42A -> 53A        0.11054
*************************************************************************
```

14. TD-DFT calculation nitrosoalkene ^{3}3B

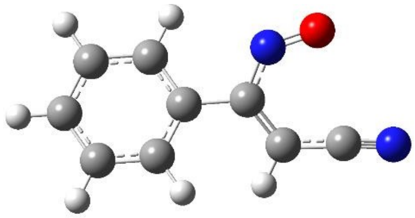

```
Excitation energies and oscillator strengths:

 Excited State   1:  3.029-A   2.6132 eV  474.45 nm  f=0.0004  <S**2>=2.044
     34B -> 41B       -0.16692
     38B -> 41B        0.44014
     40B -> 41B        0.85492
     40B -> 42B        0.10890
 This state for optimization and/or second-order correction.
 Total Energy, E(TD-HF/TD-DFT) =  -530.815415231
 Copying the excited state density for this state as the 1-particle RhoCI
density.

 Excited State   2:  3.168-A  2.8100 eV  441.22 nm  f=0.0312  <S**2>=2.259
     41A -> 43A        0.14523
     42A -> 43A        0.34397
     42A -> 48A       -0.16148
     38B -> 42B        0.33103
     40B -> 42B        0.81837

 Excited State   3:  4.025-A  3.4525 eV  359.12 nm  f=0.0018  <S**2>=3.800
     40A -> 44A        0.39607
     41A -> 43A        0.49793
     41A -> 48A        0.16594
     42A -> 43A       -0.20804
     38B -> 42B       -0.17573
```

```
    38B -> 43B        -0.13963
    39B -> 44B        -0.39105
    40B -> 43B         0.47799
    40A <- 44A         0.10430
    39B <- 44B        -0.10235

Excited State   4:  3.655-A    4.3732 eV  283.51 nm  f=0.0052  <S**2>=3.091
    40A -> 43A        -0.43570
    40A -> 48A        -0.11537
    41A -> 44A        -0.13067
    42A -> 43A         0.17421
    38B -> 41B        -0.15837
    39B -> 41B        -0.36944
    39B -> 42B         0.60492
    39B -> 43B         0.31885
    40B -> 41B         0.12077
    40B -> 44B        -0.14431

Excited State   5:  3.274-A    4.3944 eV  282.14 nm  f=0.0539  <S**2>=2.429
    40A -> 44A         0.24478
    42A -> 43A         0.62205
    42A -> 48A        -0.17454
    37B -> 41B        -0.12492
    38B -> 42B         0.18231
    39B -> 41B         0.42931
    39B -> 44B        -0.23829
    40B -> 42B        -0.39021

Excited State   6:  3.182-A    4.4864 eV  276.36 nm  f=0.0191  <S**2>=2.282
    40A -> 43A        -0.14145
    41A -> 43A        -0.22848
    42A -> 43A        -0.31504
    42A -> 48A         0.10184
    32B -> 41B         0.17749
    38B -> 41B         0.35687
    39B -> 41B         0.54101
    39B -> 42B         0.41600
    39B -> 43B         0.12010
    40B -> 41B        -0.27098
    40B -> 42B         0.18265
    40B -> 43B        -0.11816

Excited State   7:  3.370-A   4.5693 eV  271.34 nm  f=0.0082  <S**2>=2.588
    40A -> 44A        -0.24685
    41A -> 43A         0.22359
    42A -> 43A         0.19963
    27B -> 41B        -0.10853
    28B -> 41B        -0.14463
    32B -> 41B         0.20622
    34B -> 41B        -0.15174
    38B -> 41B         0.53010
    38B -> 42B         0.11600
    39B -> 41B        -0.35326
    39B -> 44B         0.24294
    40B -> 41B        -0.33067
    40B -> 42B        -0.17821
    40B -> 43B         0.20241
```

```
    40B -> 44B        0.15784

 Excited State   8:  3.478-A  4.6675 eV  265.64 nm  f=0.0058  <S**2>=2.775
    40A -> 43A        0.34738
    40A -> 44A        0.16631
    41A -> 43A       -0.12284
    41A -> 44A        0.12937
    42A -> 43A       -0.16855
    37B -> 41B       -0.10924
    37B -> 42B        0.67799
    38B -> 42B        0.13486
    39B -> 41B       -0.21371
    39B -> 42B        0.17946
    39B -> 43B       -0.14552
    39B -> 44B       -0.15605
    40B -> 43B       -0.10995
    40B -> 44B        0.25920

 Excited State   9:  3.532-A  4.7053 eV  263.50 nm  f=0.0079  <S**2>=2.869
    40A -> 43A       -0.15948
    40A -> 44A       -0.29465
    41A -> 43A        0.23860
    41A -> 44A       -0.10980
    42A -> 43A        0.12988
    37B -> 41B       -0.17448
    37B -> 42B        0.55597
    38B -> 41B       -0.14970
    38B -> 42B       -0.11689
    39B -> 41B        0.38592
    39B -> 44B        0.29492
    40B -> 41B        0.11951
    40B -> 43B        0.20345
    40B -> 44B       -0.21348

 Excited State  10:  3.435-A  4.8066 eV  257.95 nm  f=0.0033  <S**2>=2.700
    40A -> 43A        0.36093
    40A -> 44A       -0.24458
    41A -> 43A        0.18328
    37B -> 42B       -0.25620
    38B -> 41B       -0.24407
    39B -> 41B        0.17583
    39B -> 42B        0.57281
    39B -> 44B        0.24237
    40B -> 41B        0.16730
    40B -> 43B        0.19243
    40B -> 44B        0.27760

 Excited State  11:  3.253-A 4.9400 eV  250.98 nm  f=0.0305  <S**2>=2.395
    38A -> 43A       -0.13523
    40A -> 43A       -0.21415
    41A -> 43A        0.12031
    42A -> 43A       -0.29149
    31B -> 41B       -0.13597
    32B -> 42B        0.10782
    34B -> 42B        0.22241
    37B -> 41B        0.13669
    38B -> 41B       -0.12283
```

```
    38B -> 42B         0.71092
    39B -> 42B        -0.12187
    40B -> 42B        -0.21385

Excited State  12:  3.997-A  5.0495 eV  245.54 nm  f=0.0018  <S**2>=3.743
    38A -> 44A         0.11975
    40A -> 43A        -0.41892
    41A -> 44A         0.58657
    38B -> 42B        -0.14443
    38B -> 44B        -0.16933
    39B -> 42B        -0.12620
    39B -> 43B         0.17704
    40B -> 44B         0.53059

Excited State  13:  3.292-A   5.1179 eV  242.26 nm  f=0.0108  <S**2>=2.460
    38A -> 43A        -0.14479
    41A -> 43A        -0.14969
    28B -> 42B         0.11409
    29B -> 41B        -0.12406
    31B -> 41B         0.52198
    31B -> 42B         0.12841
    32B -> 41B         0.14534
    32B -> 42B        -0.14379
    33B -> 41B         0.14071
    33B -> 42B         0.10356
    34B -> 41B        -0.11126
    34B -> 42B         0.44166
    35B -> 41B        -0.19970
    36B -> 42B        -0.13816
    38B -> 43B         0.20050
    40B -> 42B         0.11957
    40B -> 43B         0.10713
    40B -> 49B         0.10122
    40B -> 51B         0.10348

Excited State  14:  3.196-A  5.4203 eV  228.74 nm  f=0.0028  <S**2>=2.304
    40A -> 43A         0.22950
    41A -> 44A        -0.44860
    39B -> 42B        -0.19267
    39B -> 43B         0.67718
    39B -> 51B        -0.10510
    40B -> 44B         0.39499

Excited State  15:  3.318-A  5.5086 eV  225.07 nm  f=0.0079  <S**2>=2.503
    37A -> 52A 0.13646
    37A -> 53A        -0.13668
    39A -> 43A         0.41824
    39A -> 48A        -0.12250
    41A -> 43A        -0.20315
    42A -> 43A         0.10635
    31B -> 41B         0.21788
    31B -> 42B         0.14931
    34B -> 42B        -0.11688
    37B -> 41B         0.53157
    37B -> 52B         0.16406
    37B -> 53B         0.16842
    38B -> 42B         0.15255
```

```
    40B -> 43B        0.25015

 Excited State  16:  3.172-A  5.5237 eV  224.46 nm  f=0.0019  <S**2>=2.266
    39A -> 43A        0.72517
    39A -> 48A       -0.26602
    31B -> 41B       -0.12769
    31B -> 42B        0.13168
    34B -> 41B       -0.10372
    37B -> 41B       -0.32301
    37B -> 42B       -0.17761
    38B -> 42B       -0.10909

 Excited State  17:  3.144-A   5.6028 eV  221.29 nm  f=0.3215  <S**2>=2.221
    39A -> 43A       -0.12707
    40A -> 44A       -0.17054
    41A -> 43A       -0.57825
    42A -> 44A        0.10801
    31B -> 41B       -0.12534
    37B -> 41B       -0.24660
    39B -> 44B       -0.14536
    40B -> 43B        0.61345

 Excited State  18:  3.254-A   5.6833 eV  218.15 nm  f=0.0190  <S**2>=2.397
    38A -> 52A       -0.12034
    38A -> 53A        0.11593
    39A -> 43A        0.16068
    41A -> 52A        0.10907
    42A -> 44A       -0.23071
    42A -> 45A       -0.39148
    42A -> 46A       -0.33600
    42A -> 48A        0.15435
    42A -> 49A        0.17226
    42A -> 51A       -0.25532
    42A -> 52A        0.42216
    42A -> 53A       -0.36828
    37B -> 41B       -0.13238
    37B -> 42B        0.10947

 Excited State  19:  3.253-A   5.7894 eV  214.16 nm  f=0.0021  <S**2>=2.395
    39A -> 43A        0.19523
    31B -> 42B       -0.33130
    31B -> 43B        0.10964
    32B -> 42B       -0.14586
    33B -> 42B        0.22886
    35B -> 41B       -0.11879
    35B -> 42B        0.74603
    36B -> 42B        0.15807

 Excited State  20:  3.427-A  5.7931 eV  214.02 nm  f=0.0405  <S**2>=2.687
    33A -> 43A       -0.12029
    38A -> 43A        0.18021
    41A -> 48A        0.14437
    42A -> 43A        0.10711
    31B -> 41B       -0.22329
    31B -> 42B       -0.11164
    32B -> 42B       -0.10910
    34B -> 41B       -0.10562
```

```
      34B -> 42B         0.60083
      35B -> 41B         0.17670
      36B -> 42B        -0.20946
      37B -> 41B         0.27191
      38B -> 42B        -0.17351
      38B -> 43B        -0.20313
      40B -> 49B        -0.10320
      40B -> 50B         0.10128
      40B -> 51B        -0.12192

 Excited State  21:   3.642-A    5.8732 eV  211.10 nm  f=0.0288  <S**2>=3.066
      38A -> 43A         0.41075
      38A -> 48A        -0.10924
      41A -> 48A         0.28658
      42A -> 43A        -0.12175
      31B -> 41B         0.26749
      31B -> 42B         0.21041
      32B -> 42B         0.11001
      33B -> 42B         0.11784
      35B -> 41B        -0.18752
      37B -> 41B        -0.24760
      38B -> 41B        -0.11732
      38B -> 42B         0.22268
      38B -> 43B        -0.30402
      40B -> 43B        -0.19663
      40B -> 49B        -0.18498
      40B -> 50B         0.17175
      40B -> 51B        -0.19397
      40B -> 52B        -0.10159

 Excited State  22:   3.105-A   5.9115 eV  209.73 nm  f=0.0114  <S**2>=2.160
      39A -> 43A         0.16676
      26B -> 41B        -0.10658
      27B -> 41B         0.21671
      28B -> 41B         0.30747
      31B -> 41B         0.21074
      31B -> 42B        -0.13689
      32B -> 41B        -0.12892
      33B -> 41B         0.19737
      33B -> 42B        -0.20509
      34B -> 41B         0.53659
      35B -> 41B         0.13171
      35B -> 42B        -0.19341
      36B -> 41B        -0.14510
      37B -> 41B        -0.10665
      38B -> 41B         0.34103
      38B -> 42B         0.18508

 Excited State  23:   3.122-A 6.0028 eV  206.54 nm  f=0.0062  <S**2>=2.187
      42A -> 45A         0.11862
      27B -> 41B         0.10763
      28B -> 41B         0.13636
      29B -> 42B        -0.13766
      31B -> 41B        -0.17583
      31B -> 42B         0.58011
      31B -> 43B        -0.17207
      32B -> 41B        -0.12570
```

```
     33B -> 42B        0.46781
     34B -> 41B        0.28619
     35B -> 41B        0.11043
     35B -> 42B        0.12551
     36B -> 42B       -0.13464
     38B -> 41B        0.17890
     38B -> 42B       -0.10268

 Excited State  24:  3.079-A  6.0876 eV  203.67 nm  f=0.0107  <S**2>=2.121
     42A -> 44A 0.76017
     42A -> 45A       -0.41526
     42A -> 46A       -0.26316
     42A -> 47A        0.24334
     42A -> 50A       -0.16580
     42A -> 52A       -0.11171

 Excited State  25:  3.191-A   6.3736 eV  194.53 nm  f=0.0066  <S**2>=2.295
     42A -> 44A        0.54254
     42A -> 45A        0.45310
     42A -> 46A        0.17101
     42A -> 47A       -0.23709
     42A -> 48A        0.22608
     42A -> 51A       -0.16173
     42A -> 52A        0.31415
     42A -> 53A       -0.24031
*********************************************************************
```

15. TD-DFT calculation of nitrosoalkene ^{1}3A in gas phase

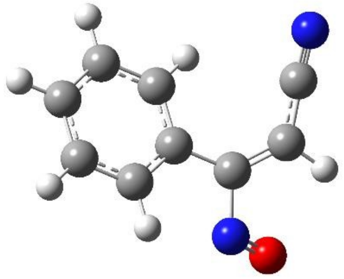

```
Excitation energies and oscillator strengths:

 Excited State   1:   Singlet-A   1.4067 eV  881.40 nm  f=0.0003  <S**2>=0.000
      39 -> 42        -0.30901
      40 -> 42         0.53811
      40 -> 43         0.16444
      41 -> 42        -0.25868
 This state for optimization and/or second-order correction.
 Total Energy, E(TD-HF/TD-DFT) =  -530.867521254
 Copying the excited state density for this state as the 1-particle RhoCI
density.

 Excited State   2: Singlet-A  3.4505 eV  359.32 nm  f=0.1404  <S**2>=0.000
      39 -> 42        -0.10653
      40 -> 42         0.23692
      41 -> 42         0.64606

 Excited State   3: Singlet-A  3.9746 eV  311.94 nm  f=0.0041  <S**2>=0.000
      39 -> 42         0.60225
```

```
      40 -> 42         0.34598

 Excited State   4: Singlet-A  4.8162 eV  257.43 nm  f=0.0205  <S**2>=0.000
      37 -> 42        -0.16588
      39 -> 42         0.14381
      39 -> 43        -0.25432
      40 -> 42        -0.16221
      40 -> 43         0.47130
      40 -> 47        -0.11732
      41 -> 43        -0.28791

 Excited State   5: Singlet-A  4.9819 eV  248.87 nm  f=0.1283  <S**2>=0.000
      32 -> 42         0.11621
      37 -> 42         0.11760
      38 -> 42         0.62896
      40 -> 43         0.15486
      41 -> 43         0.14813

 Excited State   6: Singlet-A  5.3004 eV  233.92 nm  f=0.1952  <S**2>=0.000
      37 -> 42        -0.35081
      39 -> 43        -0.13696
      40 -> 43         0.12044
      41 -> 43         0.53954

 Excited State   7: Singlet-A 5.3459 eV  231.92 nm  f=0.0057  <S**2>=0.000
      39 -> 43         0.45618
      39 -> 47         0.10758
      40 -> 43         0.22789
      41 -> 44         0.40001

 Excited State   8: Singlet-A  5.4483 eV  227.56 nm  f=0.1159  <S**2>=0.000
      37 -> 42         0.50987
      37 -> 43        -0.11716
      38 -> 42        -0.21778
      40 -> 43         0.24803
      41 -> 43         0.24456

 Excited State   9:  Singlet-A   6.3906 eV  194.01 nm  f=0.1407  <S**2>=0.00 0
      32 -> 42         0.10996
      38 -> 43         0.16709
      39 -> 43        -0.33438
      39 -> 44         0.22515
      40 -> 43        -0.14995
      40 -> 44         0.23139
      41 -> 44         0.35401
      41 -> 46        -0.10641
      41 -> 47        -0.16387

 Excited State  10:  Singlet-A  6.4694 eV  191.65 nm  f=0.0985  <S**2>=0.000
      31 -> 42        -0.10273
      35 -> 42        -0.12334
      36 -> 42         0.40079
      38 -> 43        -0.13308
      39 -> 43        -0.16764
      39 -> 44        -0.20619
      40 -> 44        -0.18216
      41 -> 44         0.30369
```

```
     41 -> 45         0.10197
     41 -> 47         0.13653

 Excited State  11: Singlet-A   6.5216 eV  190.11 nm  f=0.0686  <S**2>=0.000
     28 -> 42         0.10636
     31 -> 42         0.14662
     34 -> 42         0.11884
     35 -> 42         0.12080
     36 -> 42         0.50792
     39 -> 43         0.12717
     39 -> 44         0.14456
     40 -> 43         0.10096
     41 -> 44        -0.22170

 Excited State  12:   Singlet-A  6.5765 eV  188.53 nm  f=0.1048  <S**2>=0.000
     29 -> 42        -0.13536
     31 -> 42         0.27867
     33 -> 42        -0.13752
     35 -> 42         0.37310
     36 -> 42        -0.10665
     39 -> 44        -0.26533
     40 -> 44        -0.13276
     41 -> 47         0.14779

 Excited State  13: Singlet-A  6.7003 eV  185.04 nm  f=0.0043  <S**2>=0.000
     31 -> 42         0.10674
     34 -> 42         0.11446
     35 -> 42         0.10753
     38 -> 46         0.11649
     38 -> 49        -0.11518
     38 -> 50        -0.11193
     38 -> 52         0.11180
     41 -> 45         0.34670
     41 -> 46         0.22684
     41 -> 48        -0.14422
     41 -> 49        -0.19822
     41 -> 50        -0.21970
     41 -> 52         0.15414

 Excited State  14: Singlet-A 6.7286 eV  184.26 nm  f=0.0164  <S**2>=0.000
     27 -> 42        -0.10547
     28 -> 42        -0.10605
     29 -> 42         0.20038
     32 -> 42        -0.12995
     34 -> 42        -0.33629
     35 -> 42         0.40905
     38 -> 43        -0.14425
     40 -> 44         0.14605

 Excited State  15: Singlet-A  6.8499 eV  181.00 nm  f=0.0378  <S**2>=0.000
     34 -> 42        -0.10420
     39 -> 43         0.10046
     39 -> 44         0.14577
     40 -> 46        -0.15340
     41 -> 45         0.50525
     41 -> 46        -0.11771
     41 -> 49         0.18462
```

```
      41 -> 52         -0.14846

 Excited State  16: Singlet-A   6.8802 eV  180.20 nm  f=0.0096  <S**2>=0.000
      39 -> 44        -0.28412
      40 -> 44         0.49347
      40 -> 45         0.20216
      40 -> 46         0.15115
      41 -> 45         0.16232

 Excited State  17: Singlet-A   6.9178 eV  179.22 nm  f=0.0231  <S**2>=0.000
      32 -> 42        -0.19532
      33 -> 42         0.38808
      34 -> 42         0.40397
      38 -> 43        -0.18479

 Excited State  18: Singlet-A   7.0397 eV  176.12 nm  f=0.0318  <S**2>=0.000
      39 -> 44         0.16626
      39 -> 46        -0.17567
      40 -> 43         0.12672
      40 -> 44        -0.23746
      40 -> 45         0.41222
      40 -> 46         0.23177
      40 -> 47         0.15461
      41 -> 46        -0.18619

 Excited State  19:  Singlet-A   7.0858 eV  174.98 nm  f=0.0416  <S**2>=0.000
      39 -> 44         0.20253
      39 -> 45         0.45753
      39 -> 46        -0.10753
      40 -> 45         0.23469
      40 -> 46        -0.15676
      41 -> 46         0.18660
      41 -> 47         0.23939

 Excited State  20: Singlet-A  7.1334 eV  173.81 nm  f=0.1282  <S**2>=0.000
      33 -> 42         0.16672
      38 -> 43         0.28186
      39 -> 44         0.15726
      39 -> 45        -0.28608
      40 -> 45        -0.12154
      40 -> 46         0.12873
      41 -> 46         0.19084
      41 -> 47         0.35692
      41 -> 48         0.13091
      41 -> 50         0.11639
**********************************************************************
```

16. TD-DFT calculation of nitrosoalkene ^{1}3A in IEFPCM

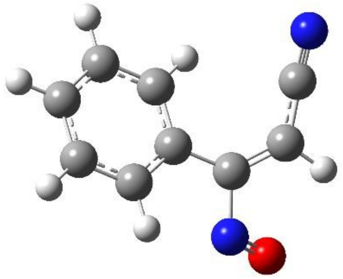

```
Excitation energies and oscillator strengths:

 Excited State   1: Singlet-A 1.3988 eV  886.33 nm  f=0.0004  <S**2>=0.000
      39 -> 42         0.59175
      39 -> 43         0.18206
      40 -> 42        -0.31283
 This state for optimization and/or second-order correction.
 Total Energy, E(TD-HF/TD-DFT) =  -530.867747553
 Copying the excited state density for this state as the 1-particle RhoCI
density.

 Excited State   2: Singlet-A3.1812 eV  389.74 nm  f=0.1463  <S**2>=0.000
      41 -> 42         0.69572

 Excited State   3: Singlet-A  3.7734 eV  328.57 nm  f=0.0066  <S**2>=0.000
      39 -> 42         0.32015
      40 -> 42         0.62130

 Excited State   4: Singlet-A  4.8297 eV  256.71 nm  f=0.1564  <S**2>=0.000
      37 -> 42         0.14779
      38 -> 42         0.50719
      39 -> 42        -0.15704
      39 -> 43         0.27200
      40 -> 43        -0.10774
      41 -> 43         0.22907

 Excited State   5: Singlet-A  4.9308 eV  251.45 nm  f=0.0420  <S**2>=0.000
      38 -> 42        -0.33848
      39 -> 42        -0.13323
      39 -> 43         0.45010
      39 -> 47        -0.11791
      40 -> 43        -0.30811
      41 -> 43        -0.12609

 Excited State   6:  Singlet-A  5.1966 eV  238.59 nm  f=0.2707  <S**2>=0.000
      37 -> 42        -0.21318
      38 -> 42        -0.21787
      41 -> 43         0.60188

 Excited State   7: Singlet-A   5.3082 eV  233.57 nm  f=0.0053  <S**2>=0.000
      37 -> 42         0.35410
      39 -> 43        -0.30253
      40 -> 43        -0.30053
      40 -> 47        -0.13175
      41 -> 44        -0.33959
```

```
 Excited State   8:   Singlet-A  5.3199 eV  233.06 nm  f=0.0797  <S**2>=0.000
      35 -> 42         0.10212
      36 -> 42        -0.10691
      37 -> 42         0.45278
      38 -> 42        -0.18327
      40 -> 43         0.31459
      41 -> 43         0.19276
      41 -> 44         0.23287

 Excited State   9: Singlet-A   6.2265 eV  199.12 nm  f=0.1458  <S**2>=0.000
      38 -> 43         0.10880
      39 -> 43        -0.18759
      40 -> 43        -0.32080
      40 -> 44         0.18655
      41 -> 44         0.42765
      41 -> 45        -0.17166
      41 -> 46         0.15269

 Excited State  10:      Singlet-A      6.2948 eV  196.96 nm  f=0.0145
<S**2>=0.000
      29 -> 42        -0.10200
      31 -> 42        -0.16144
      36 -> 42         0.60130
      37 -> 42         0.13757
      40 -> 44        -0.10456
      41 -> 44         0.10844

 Excited State  11: Singlet-A 6.4165 eV  193.23 nm  f=0.1712  <S**2>=0.000
      35 -> 42        -0.11216
      36 -> 42         0.20415
      39 -> 43         0.10296
      39 -> 44         0.12966
      40 -> 43         0.20523
      40 -> 44         0.34085
      41 -> 44        -0.26818
      41 -> 45        -0.26015
      41 -> 46         0.18812
      41 -> 47        -0.11508

 Excited State  12: Singlet-A 6.4467 eV  192.32 nm  f=0.1549  <S**2>=0.000
      38 -> 46         0.11377
      39 -> 44        -0.17616
      40 -> 44        -0.32148
      41 -> 45        -0.16740
      41 -> 46         0.20082
      41 -> 47         0.34004
      41 -> 48        -0.18277
      41 -> 49         0.10015

 Excited State  13: Singlet-A  6.5423 eV  189.51 nm  f=0.0414  <S**2>=0.000
      28 -> 42        -0.15224
      31 -> 42        -0.27057
      34 -> 42         0.11310
      35 -> 42         0.48300
      37 -> 42        -0.12636
      41 -> 45        -0.17675
```

```
     41 -> 46         0.11384

Excited State  14:   Singlet-A   6.7416 eV  183.91 nm  f=0.0132  <S**2>=0.000
     26 -> 42         0.12905
     28 -> 42         0.15965
     29 -> 42         0.13444
     31 -> 42         0.14094
     32 -> 42         0.26000
     33 -> 42         0.13198
     34 -> 42        -0.22936
     35 -> 42         0.31142
     38 -> 43         0.23663
     41 -> 45         0.13450

Excited State  15: Singlet-A  6.8268 eV  181.61 nm  f=0.1068  <S**2>=0.000
     40 -> 44         0.21498
     41 -> 45         0.50161
     41 -> 46         0.30771
     41 -> 47         0.11892
     41 -> 53        -0.11791

Excited State  16: Singlet-A  6.9675 eV  177.95 nm  f=0.1651  <S**2>=0.000
     38 -> 43         0.11298
     39 -> 45        -0.19816
     39 -> 49        -0.10872
     40 -> 44         0.26726
     40 -> 45         0.23499
     41 -> 46        -0.26864
     41 -> 47         0.37716
     41 -> 50         0.10731

Excited State  17: Singlet-A   7.0362 eV  176.21 nm  f=0.0696  <S**2>=0.000
     33 -> 42         0.11069
     34 -> 42         0.19755
     39 -> 44         0.45028
     39 -> 45         0.22209
     39 -> 49         0.14417
     40 -> 45        -0.10557
     41 -> 47         0.24471

Excited State  18:   Singlet-A  7.0782 eV  175.16 nm  f=0.1217  <S**2>=0.000
     33 -> 42         0.18623
     34 -> 42         0.23845
     39 -> 44         0.16061
     39 -> 45        -0.25271
     39 -> 49        -0.10419
     40 -> 44        -0.17939
     40 -> 45         0.26609
     40 -> 47         0.11360
     41 -> 46         0.21246
     41 -> 47        -0.13380

Excited State  19: Singlet-A 7.0849 eV  175.00 nm  f=0.0129  <S**2>=0.000
     26 -> 42        -0.13166
     28 -> 42        -0.12349
     29 -> 42        -0.11931
     31 -> 42        -0.14377
```

```
      32 -> 42         0.12371
      33 -> 42        -0.27946
      34 -> 42        -0.24852
      38 -> 43         0.16740
      39 -> 44         0.29888
      39 -> 45        -0.10492
      40 -> 44        -0.17087
      40 -> 45         0.15001

 Excited State   20: Singlet-A   7.1283 eV  173.93 nm  f=0.0094  <S**2>=0.000
      34 -> 42        -0.12144
      38 -> 43        -0.13044
      39 -> 45         0.33445
      39 -> 47         0.11190
      40 -> 45         0.38853
      40 -> 46         0.32740
      40 -> 48         0.10419

 Excited State   21: Singlet-A   7.1833 eV  172.60 nm  f=0.0170  <S**2>=0.000
      31 -> 42        -0.18900
      33 -> 42         0.51000
      33 -> 43        -0.12288
      34 -> 42        -0.27620

 Excited State   22: Singlet-A   7.2532 eV  170.94 nm  f=0.0909  <S**2>=0.000
      31 -> 42        -0.12321
      33 -> 42         0.13390
      34 -> 42         0.18256
      38 -> 43         0.28540
      39 -> 44        -0.27492
      39 -> 45         0.15847
      40 -> 46        -0.14937
      40 -> 47         0.29417
      41 -> 44        -0.11063
      41 -> 46         0.13338
      41 -> 48         0.10472

 Excited State   23: Singlet-A 7.3015 eV  169.81 nm  f=0.1180  <S**2>=0.000
      34 -> 42        -0.15581
      35 -> 42         0.11330
      38 -> 43        -0.26140
      39 -> 46        -0.21611
      39 -> 47         0.22971
      40 -> 46        -0.31364
      40 -> 47         0.32441
      41 -> 44        -0.11216

 Excited State  24:      Singlet-A      7.3660 eV  168.32 nm  f=0.0270
<S**2>=0.000
      38 -> 45        -0.11728
      39 -> 47         0.10909
      40 -> 47        -0.15245
      41 -> 45        -0.10530
      41 -> 46         0.25144
      41 -> 47         0.20965
      41 -> 48         0.40766
      41 -> 49        -0.29684
```

```
 Excited State  25:      Singlet-A      7.4064 eV  167.40 nm  f=0.0006
<S**2>=0.000
      32 -> 42         0.31350
      34 -> 42         0.22631
      36 -> 43        -0.11968
      37 -> 43        -0.13786
      39 -> 43         0.11324
      39 -> 46        -0.10811
      39 -> 47         0.35242
      39 -> 49        -0.12127
      40 -> 47        -0.21583
*****************************************************************************
```

17. TD-DFT calculation of nitrosoalkene [1]3A in SMD

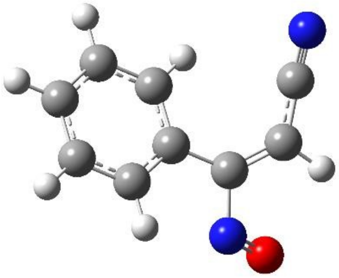

```
Excitation energies and oscillator strengths:

 Excited State   1: Singlet-A      1.4003 eV  885.39 nm  f=0.0004
<S**2>=0.000
      39 -> 42         0.61019
      39 -> 43         0.18574
      40 -> 42        -0.27648
 This state for optimization and/or second-order correction.
 Total Energy, E(TD-HF/TD-DFT) =  -530.873500907
 Copying the excited state density for this state as the 1-particle RhoCI
density.

 Excited State   2:   Singlet-A  3.1473 eV  393.93 nm  f=0.1484  <S**2>=0.000
      41 -> 42         0.69595

 Excited State   3: Singlet-A  3.7574 eV  329.97 nm  f=0.0070  <S**2>=0.000
      39 -> 42         0.28207
      40 -> 42         0.63972

 Excited State   4: Singlet-A  4.8236 eV  257.04 nm  f=0.1745  <S**2>=0.000
      37 -> 42         0.12906
      38 -> 42         0.54768
      39 -> 42        -0.14056
      39 -> 43         0.21116
      41 -> 43         0.24900

 Excited State   5: Singlet-A 4.9440 eV  250.78 nm  f=0.0253  <S**2>=0.000
      37 -> 42         0.12451
      38 -> 42        -0.26064
      39 -> 42        -0.15453
      39 -> 43         0.49972
      39 -> 47        -0.12735
```

```
     40 -> 43        -0.29303
     41 -> 43        -0.10514

Excited State   6: Singlet-A  5.1722 eV  239.71 nm  f=0.2895  <S**2>=0.000
     37 -> 42        -0.16533
     38 -> 42        -0.23815
     41 -> 43         0.61158

Excited State   7: Singlet-A  5.3011 eV  233.88 nm  f=0.0046  <S**2>=0.000
     37 -> 42        -0.16312
     39 -> 43         0.28294
     40 -> 43         0.40984
     40 -> 47         0.15165
     41 -> 44         0.39552

Excited State   8: Singlet-A     5.3185 eV  233.12 nm  f=0.0708  <S**2>=0.000
     35 -> 42         0.13446
     36 -> 42        -0.14720
     37 -> 42         0.55862
     37 -> 43        -0.12223
     38 -> 42        -0.16277
     40 -> 43         0.18561
     41 -> 43         0.15626

Excited State   9:   Singlet-A   6.1941 eV  200.16 nm  f=0.1733  <S**2>=0.000
     38 -> 43         0.10229
     39 -> 43        -0.17186
     40 -> 43        -0.34112
     40 -> 44         0.17785
     41 -> 44         0.44593
     41 -> 45        -0.16298
     41 -> 46         0.13073

Excited State  10: Singlet-A  6.2406 eV  198.67 nm  f=0.0065  <S**2>=0.000
     31 -> 42        -0.14588
     36 -> 42         0.61519
     37 -> 42         0.16262

Excited State  11: Singlet-A   6.3812 eV  194.30 nm  f=0.1926  <S**2>=0.000
     35 -> 42        -0.11443
     36 -> 42         0.16092
     39 -> 44         0.12767
     40 -> 43         0.19020
     40 -> 44         0.39272
     41 -> 44        -0.26138
     41 -> 45        -0.24262
     41 -> 46         0.16579
     41 -> 47        -0.17123

Excited State  12: Singlet-A  6.4205 eV  193.11 nm  f=0.1456  <S**2>=0.000
     38 -> 46        -0.12086
     39 -> 44         0.14485
     40 -> 44         0.29601
     41 -> 45         0.20701
     41 -> 46        -0.24225
     41 -> 47        -0.28059
     41 -> 48         0.21631
```

```
     41 -> 49         -0.11496

Excited State  13:   Singlet-A   6.5185 eV  190.20 nm  f=0.0381  <S**2>=0.000
     28 -> 42         -0.15518
     31 -> 42         -0.26150
     35 -> 42          0.50014
     37 -> 42         -0.13615
     41 -> 45         -0.17811
     41 -> 46          0.10872

Excited State  14: Singlet-A  6.7385 eV  183.99 nm  f=0.0144  <S**2>=0.000
     26 -> 42          0.12312
     28 -> 42          0.15806
     29 -> 42          0.12455
     31 -> 42          0.14039
     32 -> 42          0.24508
     33 -> 42          0.11894
     34 -> 42         -0.21303
     35 -> 42          0.27104
     38 -> 43          0.22730
     41 -> 45          0.24327

Excited State  15: Singlet-A  6.7756 eV  182.99 nm  f=0.1104  <S**2>=0.000
     40 -> 44          0.21803
     41 -> 45          0.44965
     41 -> 46          0.30641
     41 -> 47          0.10324
     41 -> 53         -0.11672

Excited State  16: Singlet-A   6.9305 eV  178.90 nm  f=0.1503  <S**2>=0.000
     39 -> 45         -0.23561
     39 -> 46          0.10213
     39 -> 49         -0.13363
     40 -> 44          0.25574
     40 -> 45          0.23763
     41 -> 46         -0.22280
     41 -> 47          0.36287
     41 -> 48         -0.11648
     41 -> 50          0.11348

Excited State  17: Singlet-A   7.0048 eV  177.00 nm  f=0.1445  <S**2>=0.000
     39 -> 44          0.30635
     39 -> 45          0.31623
     39 -> 49          0.18267
     40 -> 45         -0.16424
     41 -> 46         -0.17357
     41 -> 47          0.31224

Excited State  18: Singlet-A 7.0673 eV  175.43 nm  f=0.0968  <S**2>=0.000
     31 -> 42          0.10986
     33 -> 42          0.10759
     34 -> 42          0.28198
     39 -> 44          0.30628
     40 -> 44         -0.15375
     40 -> 45          0.31809
     40 -> 47          0.18985
     41 -> 46          0.17293
```

```
Excited State  19: Singlet-A 7.0849 eV  175.00 nm  f=0.0105  <S**2>=0.000
     26 -> 42         0.11615
     28 -> 42         0.11830
     31 -> 42         0.14581
     32 -> 42        -0.11007
     33 -> 42         0.13701
     34 -> 42         0.33946
     39 -> 44        -0.13939
     39 -> 45        -0.18282
     40 -> 45        -0.30940
     40 -> 46        -0.19242

Excited State  20: Singlet-A 7.0942 eV  174.77 nm  f=0.0029  <S**2>=0.000
     31 -> 42        -0.12135
     35 -> 42        -0.11126
     38 -> 43         0.20704
     39 -> 44         0.30971
     39 -> 45        -0.28748
     39 -> 47        -0.15170
     40 -> 44        -0.18159
     40 -> 45        -0.15870
     40 -> 46        -0.25435

Excited State  21: Singlet-A  7.2070 eV  172.03 nm  f=0.0457  <S**2>=0.000
     32 -> 42        -0.11516
     33 -> 42         0.42252
     34 -> 42        -0.25748
     38 -> 43        -0.25668
     39 -> 44         0.21090
     39 -> 45        -0.10161
     40 -> 47        -0.15317

Excited State  22:  Singlet-A 7.2401 eV  171.25 nm  f=0.0613  <S**2>=0.000
     31 -> 42        -0.16535
     33 -> 42         0.39673
     38 -> 43         0.13239
     39 -> 44        -0.22703
     39 -> 45         0.11223
     40 -> 46        -0.17162
     40 -> 47         0.29872

Excited State  23:  Singlet-A  7.2732 eV  170.47 nm  f=0.1008  <S**2>=0.000
     33 -> 42        -0.11339
     34 -> 42        -0.18601
     35 -> 42         0.12599
     38 -> 43        -0.29358
     39 -> 46        -0.17181
     39 -> 47         0.21548
     40 -> 46        -0.26786
     40 -> 47         0.30961

Excited State  24: Singlet-A 7.3222 eV  169.33 nm  f=0.0273  <S**2>=0.000
     38 -> 45        -0.12014
     40 -> 47        -0.10962
     41 -> 45        -0.11981
     41 -> 46         0.27231
```

```
      41 -> 47         0.22232
      41 -> 48         0.39146
      41 -> 49        -0.31300

 Excited State  25: Singlet-A  7.3966 eV  167.62 nm  f=0.0026  <S**2>=0.000
      32 -> 42         0.31845
      34 -> 42         0.22326
      36 -> 43        -0.13913
      37 -> 43        -0.18502
      39 -> 43         0.11113
      39 -> 47         0.34957
      39 -> 49        -0.11124
      40 -> 47        -0.19553
****************************************************************************
```

18. TD-DFT calculation of nitrosoalkene ^{1}3A in I-PCM

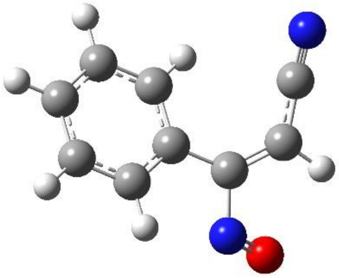

```
Excitation energies and oscillator strengths:

 Excited State   1: Singlet-A  1.3680 eV  906.31 nm  f=0.0004  <S**2>=0.000
      39 -> 42        -0.35189
      39 -> 43        -0.11056
      40 -> 42         0.56801
      40 -> 43         0.17480
 This state for optimization and/or second-order correction.
 Total Energy, E(TD-HF/TD-DFT) =  -530.861903706
 Copying the excited state density for this state as the 1-particle RhoCI
density.

 Excited State   2: Singlet-A 3.3139 eV  374.13 nm  f=0.1180  <S**2>=0.000
      41 -> 42         0.69439

 Excited State   3: Singlet-A   3.8744 eV  320.01 nm   f=0.0052   <S**2>=0.000
      39 -> 42         0.58807
      40 -> 42         0.37431

 Excited State   4: Singlet-A  4.8054 eV  258.01 nm  f=0.0237  <S**2>=0.000
      37 -> 42        -0.19615
      38 -> 42        -0.16058
      39 -> 42         0.13752
      39 -> 43        -0.29558
      40 -> 42        -0.17452
      40 -> 43         0.47949
      40 -> 47        -0.11756
      41 -> 43        -0.10241

 Excited State   5:  Singlet-A  4.9540 eV  250.27 nm   f=0.1126   <S**2>=0.000
      32 -> 42         0.10671
```

```
     38 -> 42         0.58258
     40 -> 43         0.24697
     41 -> 43         0.21154

Excited State   6: Singlet-A  5.2255 eV  237.27 nm  f=0.0935  <S**2>=0.000
     37 -> 42         0.53307
     37 -> 43        -0.11634
     40 -> 43         0.10512
     41 -> 43        -0.37160

Excited State   7: Singlet-A 5.3106 eV  233.46 nm  f=0.1145  <S**2>=0.000
     37 -> 42         0.24633
     38 -> 42        -0.17845
     39 -> 43        -0.33502
     41 -> 43         0.40627
     41 -> 44        -0.25393

Excited State   8: Singlet-A  5.3469 eV  231.88 nm  f=0.0852  <S**2>=0.000
     37 -> 42         0.18913
     38 -> 42        -0.23291
     39 -> 43         0.29666
     40 -> 43         0.23444
     41 -> 43         0.32195
     41 -> 44         0.34416

Excited State   9:  Singlet-A  6.3012 eV  196.76 nm  f=0.0655  <S**2>=0.000
     31 -> 42         0.13578
     32 -> 42         0.10230
     38 -> 43         0.14684
     39 -> 43        -0.28259
     39 -> 44         0.19423
     40 -> 43        -0.15355
     40 -> 44         0.19152
     41 -> 44         0.31878
     41 -> 45        -0.19416
     41 -> 46        -0.17602
     41 -> 47        -0.10493
     41 -> 49         0.10637

Excited State  10:  Singlet-A  6.3992 eV  193.75 nm  f=0.0562  <S**2>=0.000
     29 -> 42         0.10669
     31 -> 42         0.18919
     35 -> 42         0.12640
     36 -> 42         0.51294
     39 -> 43         0.19091
     41 -> 44        -0.24328

Excited State  11:  Singlet-A 6.4811 eV  191.30 nm  f=0.0985  <S**2>=0.000
     36 -> 42         0.32072
     39 -> 43        -0.21485
     39 -> 44        -0.19305
     40 -> 43        -0.13613
     40 -> 44        -0.13866
     41 -> 44         0.29651
     41 -> 45         0.27519
     41 -> 46         0.17592
```

```
Excited State  12: Singlet-A  6.5344 eV  189.74 nm  f=0.1718  <S**2>=0.000
     31 -> 42         0.11368
     35 -> 42         0.11523
     39 -> 44        -0.33759
     40 -> 44        -0.21165
     41 -> 46        -0.12023
     41 -> 47         0.33801
     41 -> 48         0.13627
     41 -> 49         0.13176
     41 -> 50        -0.13382
     41 -> 52        -0.13233

Excited State  13:  Singlet-A  6.5872 eV  188.22 nm  f=0.0210  <S**2>=0.000
     28 -> 42        -0.13624
     31 -> 42         0.27375
     34 -> 42        -0.17002
     35 -> 42         0.37472
     36 -> 42        -0.23392
     41 -> 45         0.22135
     41 -> 46         0.13239

Excited State  14:  Singlet-A  6.7159 eV  184.61 nm  f=0.0061  <S**2>=0.000
     26 -> 42        -0.10732
     28 -> 42         0.10214
     29 -> 42        -0.13429
     32 -> 42        -0.22273
     33 -> 42        -0.19409
     34 -> 42         0.20913
     35 -> 42         0.41582
     38 -> 43        -0.20287

Excited State  15:  Singlet-A 6.8398 eV  181.27 nm  f=0.0555  <S**2>=0.000
     40 -> 44         0.24635
     41 -> 45         0.48673
     41 -> 46        -0.28935
     41 -> 48         0.11895
     41 -> 49         0.12884

Excited State  16: Singlet-A  6.8802 eV  180.20 nm  f=0.0037  <S**2>=0.000
     39 -> 44        -0.25746
     39 -> 45        -0.12523
     39 -> 46        -0.11143
     40 -> 44         0.40350
     40 -> 45         0.30548
     40 -> 46         0.15129
     40 -> 49         0.10785
     41 -> 45        -0.11276
     41 -> 46         0.14170

Excited State  17: Singlet-A 6.9356 eV  178.77 nm  f=0.0125  <S**2>=0.000
     32 -> 42        -0.12246
     33 -> 42         0.60811
     33 -> 43        -0.12767
     34 -> 42         0.17447
     38 -> 43        -0.11627

Excited State  18: Singlet-A  6.9818 eV  177.58 nm  f=0.0273  <S**2>=0.000
```

```
      35 -> 42         0.10625
      39 -> 44         0.30674
      39 -> 45        -0.11347
      39 -> 46        -0.12909
      40 -> 43         0.11216
      40 -> 44        -0.25763
      40 -> 45         0.36973
      40 -> 46         0.16036
      40 -> 47         0.14237
      41 -> 47         0.15962

 Excited State  19: Singlet-A  7.0670 eV  175.44 nm  f=0.1837  <S**2>=0.000
      38 -> 43         0.15413
      39 -> 44         0.19510
      39 -> 45         0.29192
      40 -> 44         0.18364
      40 -> 46        -0.19309
      41 -> 46         0.29111
      41 -> 47         0.35657

 Excited State  20: Singlet-A  7.1035 eV  174.54 nm  f=0.0654  <S**2>=0.000
      34 -> 42        -0.11006
      38 -> 43        -0.18035
      39 -> 45         0.40765
      39 -> 46        -0.21031
      40 -> 45         0.30345
      40 -> 46        -0.20792
      41 -> 46        -0.14829
      41 -> 47        -0.16530

 Excited State  21: Singlet-A 7.1620 eV  173.11 nm  f=0.0074  <S**2>=0.000
      26 -> 42         0.17987
      28 -> 42        -0.13944
      29 -> 42         0.18994
      31 -> 42         0.26346
      33 -> 42        -0.15623
      34 -> 42         0.42387
      35 -> 42        -0.13830

 Excited State  22: Singlet-A 7.2424 eV  171.19 nm  f=0.0442  <S**2>=0.000
      38 -> 43         0.24213
      39 -> 44        -0.19124
      39 -> 45         0.16459
      40 -> 47         0.30579
      40 -> 49        -0.11382
      41 -> 46        -0.28551
      41 -> 48        -0.16663
      41 -> 49        -0.21418

 Excited State  23:      Singlet-A      7.2852 eV  170.19 nm  f=0.0536
<S**2>=0.000
      39 -> 44        -0.14608
      39 -> 46        -0.15836
      39 -> 47        -0.28249
      40 -> 43         0.12142
      40 -> 47         0.29967
      41 -> 46         0.20060
```

```
      41 -> 47         -0.24058
      41 -> 48          0.14288
      41 -> 49          0.23906

 Excited State  24: Singlet-A  7.3755 eV  168.10 nm  f=0.0871  <S**2>=0.000
      34 -> 42         -0.10941
      35 -> 42         -0.10983
      38 -> 43         -0.26485
      39 -> 46          0.29657
      39 -> 47          0.25201
      40 -> 46          0.13635
      40 -> 47          0.36964
      41 -> 44         -0.13735

 Excited State  25: Singlet-A  7.4153 eV  167.20 nm  f=0.0749  <S**2>=0.000
      32 -> 42  0.18825
      34 -> 42          0.26873
      38 -> 43          0.28039
      39 -> 44         -0.12372
      39 -> 46          0.17702
      39 -> 47          0.24595
      39 -> 49         -0.10472
      40 -> 49          0.10165
      41 -> 47         -0.12498
      41 -> 48          0.11673
      41 -> 49          0.16653
 SavETr:  write IOETrn=   770 NScale= 10 NData=  16 NLR=1 NState=   25
LETran=     460.
*****************************************************************************
```

19. TD-DFT calculation of nitrosoalkene ^{1}3A in C-PCM

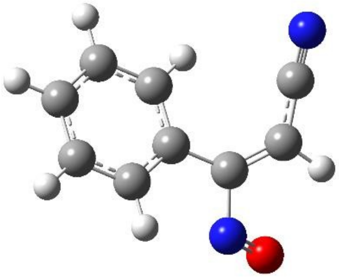

```
Excitation energies and oscillator strengths:

 Excited State   1: Singlet-A  1.4006 eV  885.20 nm  f=0.0005  <S**2>=0.000
      39 -> 42          0.58821
      39 -> 43          0.18115
      40 -> 42         -0.31926
 This state for optimization and/or second-order correction.
 Total Energy, E(TD-HF/TD-DFT) =  -530.868182187
 Copying the excited state density for this state as the 1-particle RhoCI
density.

 Excited State   2: Singlet-A  3.1762 eV  390.35 nm  f=0.1508  <S**2>=0.000
      41 -> 42          0.69574

 Excited State   3: Singlet-A   3.7810 eV  327.91 nm  f=0.0070  <S**2>=0.000
```

```
     39 -> 42         0.32691
     40 -> 42         0.61765

Excited State   4:   Singlet-A  4.8264 eV  256.89 nm  f=0.1689  <S**2>=0.000
     37 -> 42         0.14025
     38 -> 42         0.52031
     39 -> 42        -0.15187
     39 -> 43         0.25455
     40 -> 43        -0.10167
     41 -> 43         0.23263

Excited State   5:  Singlet-A  4.9321 eV  251.38 nm  f=0.0387  <S**2>=0.000
     37 -> 42         0.10737
     38 -> 42        -0.31896
     39 -> 42        -0.13822
     39 -> 43         0.45570
     39 -> 47        -0.11792
     40 -> 43        -0.31578
     41 -> 43        -0.12170

Excited State   6:  Singlet-A  5.1920 eV  238.80 nm  f=0.2814  <S**2>=0.000
     37 -> 42        -0.19733
     38 -> 42        -0.22464
     41 -> 43         0.60638

Excited State   7: Singlet-A   5.3087 eV  233.55 nm  f=0.0029  <S**2>=0.000
     37 -> 42        -0.32844
     39 -> 43         0.30850
     40 -> 43         0.31386
     40 -> 47         0.13309
     41 -> 44         0.35311

Excited State   8: Singlet-A 5.3192 eV  233.09 nm  f=0.0799  <S**2>=0.000
     35 -> 42         0.10703
     36 -> 42        -0.11301
     37 -> 42         0.47880
     37 -> 43        -0.10413
     38 -> 42        -0.17570
     40 -> 43         0.29842
     41 -> 43         0.18304
     41 -> 44         0.21130

Excited State   9: Singlet-A  6.2143 eV  199.52 nm  f=0.1713  <S**2>=0.000
     38 -> 43         0.10442
     39 -> 43        -0.19606
     40 -> 43        -0.32701
     40 -> 44         0.17629
     41 -> 44         0.44292
     41 -> 45        -0.15830
     41 -> 46         0.14574

Excited State  10: Singlet-A 6.2964 eV  196.91 nm  f=0.0162  <S**2>=0.000
     29 -> 42        -0.10247
     31 -> 42        -0.16487
     36 -> 42         0.59526
     37 -> 42         0.13487
     40 -> 44        -0.12222
```

```
     41 -> 44         0.10342

 Excited State  11: Singlet-A 6.4088 eV  193.46 nm  f=0.1868  <S**2>=0.000
     35 -> 42        -0.10099
     36 -> 42         0.22268
     39 -> 44         0.15012
     40 -> 43         0.18632
     40 -> 44         0.37055
     41 -> 44        -0.24871
     41 -> 45        -0.24503
     41 -> 46         0.18502
     41 -> 47        -0.13314

 Excited State  12: Singlet-A  6.4419 eV  192.46 nm  f=0.1490  <S**2>=0.000
     38 -> 46         0.11833
     39 -> 44        -0.16981
     40 -> 44        -0.29198
     41 -> 45        -0.18686
     41 -> 46         0.21705
     41 -> 47         0.32805
     41 -> 48        -0.18915
     41 -> 49         0.10480

 Excited State  13:   Singlet-A  6.5435 eV  189.48 nm   f=0.0426   <S**2>=0.000
     28 -> 42        -0.15339
     31 -> 42        -0.27129
     34 -> 42         0.11138
     35 -> 42         0.48222
     36 -> 42        -0.10020
     37 -> 42        -0.12674
     41 -> 45        -0.17077
     41 -> 46         0.11579

 Excited State  14:   Singlet-A  6.7425 eV  183.89 nm   f=0.0159   <S**2>=0.000
     26 -> 42         0.12864
     28 -> 42         0.15818
     29 -> 42         0.13348
     31 -> 42         0.13855
     32 -> 42         0.26095
     33 -> 42         0.12968
     34 -> 42        -0.23120
     35 -> 42         0.31209
     38 -> 43         0.23912
     41 -> 45         0.12625

 Excited State  15: Singlet-A 6.8200 eV  181.79 nm  f=0.1170  <S**2>=0.000
     40 -> 44         0.21780
     41 -> 45         0.50975
     41 -> 46         0.28863
     41 -> 47         0.14313
     41 -> 53        -0.12126

 Excited State  16:   Singlet-A  6.9604 eV  178.13 nm  f=0.1852   <S**2>=0.000
     38 -> 43         0.10997
     39 -> 45        -0.16333
     40 -> 44         0.26130
     40 -> 45         0.21555
```

```
      41 -> 46        -0.30335
      41 -> 47         0.39317
      41 -> 50         0.11523

 Excited State  17: Singlet-A 7.0366 eV  176.20 nm  f=0.0549  <S**2>=0.000
      34 -> 42         0.18068
      39 -> 44         0.45717
      39 -> 45         0.23471
      39 -> 49         0.15425
      40 -> 45        -0.13255
      41 -> 47         0.21451

 Excited State  18: Singlet-A 7.0736 eV  175.28 nm  f=0.1282  <S**2>=0.000
      33 -> 42         0.10838
      34 -> 42         0.17798
      39 -> 43        -0.10342
      39 -> 44         0.22873
      39 -> 45        -0.25756
      39 -> 46         0.11599
      39 -> 49        -0.10356
      40 -> 44        -0.20803
      40 -> 45         0.31425
      40 -> 47         0.14818
      41 -> 46         0.21180
      41 -> 47        -0.10846

 Excited State  19: Singlet-A   7.0859 eV  174.97 nm  f=0.0020  <S**2>=0.000
      26 -> 42         0.14865
      28 -> 42         0.14555
      29 -> 42         0.13485
      31 -> 42         0.16731
      32 -> 42        -0.13886
      33 -> 42         0.30812
      34 -> 42         0.30226
      35 -> 42         0.10805
      38 -> 43        -0.18209
      39 -> 44        -0.23860
      40 -> 44         0.12028

 Excited State  20: Singlet-A   7.1259 eV  173.99 nm  f=0.0092  <S**2>=0.000
      34 -> 42        -0.11163
      38 -> 43        -0.12592
      39 -> 45         0.34954
      39 -> 47         0.11838
      40 -> 45         0.38258
      40 -> 46         0.31624
      40 -> 48         0.10647

 Excited State  21:      Singlet-A      7.1873 eV  172.50 nm  f=0.0208
<S**2>=0.000
      31 -> 42        -0.17769
      33 -> 42         0.50759
      33 -> 43        -0.12225
      34 -> 42        -0.28188
      38 -> 43        -0.11489

 Excited State  22: Singlet-A   7.2449 eV  171.13 nm  f=0.0861  <S**2>=0.000
```

```
      31 -> 42         -0.13309
      33 -> 42          0.16531
      34 -> 42          0.17388
      38 -> 43          0.28319
      39 -> 44         -0.27039
      39 -> 45          0.15794
      40 -> 46         -0.16608
      40 -> 47          0.29433
      41 -> 44         -0.10636
      41 -> 46          0.10767

 Excited State  23: Singlet-A  7.2932 eV  170.00 nm  f=0.1132  <S**2>=0.000
      34 -> 42          0.16034
      35 -> 42         -0.11533
      38 -> 43          0.27086
      39 -> 46          0.22198
      39 -> 47         -0.22561
      40 -> 46          0.31665
      40 -> 47         -0.31257
      41 -> 44          0.10482

 Excited State  24:      Singlet-A      7.3696 eV  168.24 nm  f=0.0246
<S**2>=0.000
      38 -> 45         -0.12209
      39 -> 47          0.10201
      40 -> 47         -0.13136
      41 -> 45         -0.10227
      41 -> 46          0.24794
      41 -> 47          0.21605
      41 -> 48          0.41440
      41 -> 49         -0.30127

 Excited State  25: Singlet-A  7.4081 eV  167.36 nm  f=0.0007  <S**2>=0.000
      32 -> 42          0.30419
      34 -> 42          0.22611
      36 -> 43         -0.12143
      37 -> 43         -0.13757
      39 -> 43          0.11381
      39 -> 46         -0.11980
      39 -> 47          0.34902
      39 -> 49         -0.12859
      40 -> 47         -0.22096
************************************************************
```

C. Quantum chemical calculation using M062X

1. Optimization of 1A

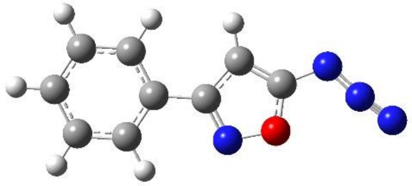

```
DFT/M062X 6-31+G(d,p), E =-640.452351 a.u.
Standard orientation:
```

Center Number	Atomic Number	Atomic Type	Coordinates (Angstroms) X	Y	Z
1	6	0	-2.019231	0.423038	-0.000131
2	6	0	-0.810873	1.038702	0.000459
3	6	0	0.099033	-0.060454	-0.000347
4	1	0	-0.618354	2.098406	0.002014
5	7	0	-0.524243	-1.216769	-0.001816
6	7	0	-3.286884	0.973123	0.000138
7	7	0	-4.223815	0.156209	0.000650
8	7	0	-5.139983	-0.496479	0.001197
9	8	0	-1.882625	-0.909147	-0.001301
10	6	0	1.571663	-0.019470	-0.000058
11	6	0	2.243746	1.204940	-0.000602
12	6	0	3.635327	1.243786	-0.000376
13	6	0	4.366627	0.058855	0.000410
14	6	0	3.699092	-1.166265	0.000944
15	6	0	2.310273	-1.208272	0.000714
16	1	0	1.683335	2.134757	-0.001340
17	1	0	4.145970	2.201189	-0.000836
18	1	0	5.451382	0.088463	0.000594
19	1	0	4.263321	-2.093168	0.001560
20	1	0	1.785876	-2.158219	0.001151

2. Optimization of 1B

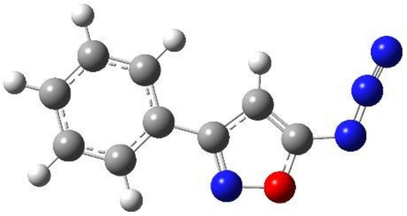

DFT/M062X 6-31+G(d,p), E = -640.450719 a.u.

Standard orientation:

Center Number	Atomic Number	Atomic Type	Coordinates (Angstroms) X	Y	Z
1	6	0	2.118107	-0.499207	-0.000020
2	6	0	1.091711	0.395881	0.000014
3	6	0	-0.058433	-0.452857	-0.000024
4	1	0	1.148935	1.472552	0.000069
5	7	0	0.283631	-1.719653	-0.000078
6	7	0	3.505574	-0.412273	-0.000009
7	7	0	3.937773	0.752179	0.000046
8	7	0	4.438579	1.758766	0.000093
9	8	0	1.660488	-1.750288	-0.000075
10	6	0	-1.481169	-0.062504	-0.000008
11	6	0	-1.844272	1.286492	-0.000087
12	6	0	-3.187823	1.654211	-0.000071
13	6	0	-4.178623	0.675417	0.000024
14	6	0	-3.820203	-0.673423	0.000102

15	6	0	-2.480171	-1.043498	0.000087
16	1	0	-1.079585	2.057275	-0.000168
17	1	0	-3.458459	2.705162	-0.000134
18	1	0	-5.225734	0.960900	0.000037
19	1	0	-4.588713	-1.439754	0.000178
20	1	0	-2.193994	-2.090040	0.000150

3. Optimization of nitrene ^{3}2

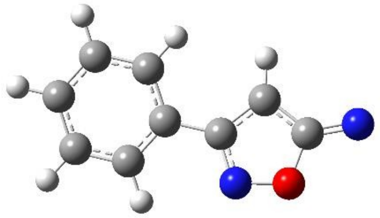

DFT/M062X 6-31+G(d,p), E =-530.955350 a.u.
Standard orientation:

Center Number	Atomic Number	Atomic Type	Coordinates (Angstroms) X	Y	Z
1	6	0	-2.895520	0.315955	0.000340
2	6	0	-1.637269	0.957865	0.000937
3	6	0	-0.693880	-0.088583	-0.000127
4	1	0	-1.478061	2.025228	0.002117
5	7	0	-1.305616	-1.279826	-0.001287
6	7	0	-4.108674	0.785294	0.000872
7	8	0	-2.667531	-1.053487	-0.001006
8	6	0	0.778891	-0.009738	-0.000045
9	6	0	1.427428	1.235489	-0.000802
10	6	0	2.820928	1.308118	-0.000697
11	6	0	3.584405	0.137805	0.000148
12	6	0	2.945231	-1.106297	0.000882
13	6	0	1.553285	-1.183077	0.000788
14	1	0	0.848441	2.154712	-0.001572
15	1	0	3.308558	2.279246	-0.001311
16	1	0	4.669685	0.194581	0.000232
17	1	0	3.532553	-2.020660	0.001550
18	1	0	1.058113	-2.148706	0.001391

4. Optimization of nitrosoalkene ^{3}3A

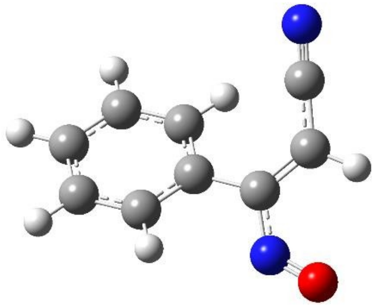

DFT/M062X 6-31+G(d,p), E =-530.958258 a.u.
Standard orientation:

Center Number	Atomic Number	Atomic Type	Coordinates (Angstroms) X	Y	Z
1	6	0	1.178772	-1.226629	-0.531812
2	6	0	0.377521	-0.247637	0.070793
3	6	0	0.978144	0.879277	0.641968
4	6	0	2.362101	1.029560	0.596717
5	6	0	3.155413	0.056936	-0.009389
6	6	0	2.560888	-1.073580	-0.570476
7	1	0	0.713825	-2.100627	-0.979234
8	1	0	0.372151	1.632913	1.134036
9	1	0	2.819254	1.907189	1.043133
10	1	0	4.234057	0.177878	-0.043056
11	1	0	3.173171	-1.834333	-1.045081
12	6	0	-1.090557	-0.430515	0.112829
13	6	0	-2.059936	0.538899	-0.018668
14	6	0	-1.774988	1.897222	-0.305870
15	7	0	-1.490271	-1.732889	0.284091
16	7	0	-1.584383	3.019591	-0.539051
17	8	0	-2.601054	-2.199460	0.210649
18	1	0	-3.105608	0.264540	0.073175

5. Optimization of nitrosoalkene ^{3}3B

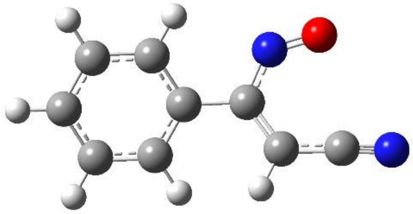

DFT/M062X 6-31+G(d,p), E =-530.965381 a.u.
Standard orientation:

Center Number	Atomic Number	Atomic Type	Coordinates (Angstroms) XY	Z	
1	6	0	-2.998868	-0.890864	-0.254671
2	6	0	-1.578890	-0.918674	-0.285162
3	6	0	-0.750081	0.120145	0.036181
4	1	0	-1.131713	-1.847917	-0.618936
5	7	0	-1.205098	1.370123	0.325079
6	7	0	-4.158643	-0.946758	-0.255388
7	8	0	-2.350755	1.702152	0.475575
8	6	0	0.725614	0.004884	0.006714
9	6	0	1.342732	-1.196754	0.369006
10	6	0	2.729687	-1.310205	0.337328
11	6	0	3.512378	-0.223016	-0.046907
12	6	0	2.902099	0.981305	-0.394884
13	6	0	1.515952	1.098671	-0.366866
14	1	0	0.739660	-2.034890	0.705227
15	1	0	3.198748	-2.245001	0.626330
16	1	0	4.593671	-0.311675	-0.068412

17	1	0	3.506273	1.831725	-0.693500
18	1	0	1.041855	2.034043	-0.647566

6. Optimization of nitrosoalkene [1]3A

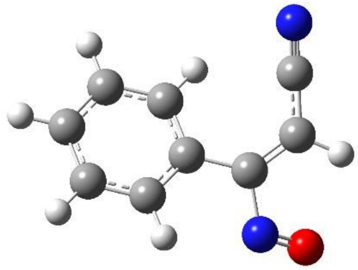

DFT/M062X 6-31+G(d,p), E =-530.972512 a.u.
Standard orientation:

Center Number	Atomic Number	Atomic Type	Coordinates (Angstroms) X	Y	Z
1	6	0	1.238520	-1.204906	-0.548719
2	6	0	0.388280	-0.247327	0.026033
3	6	0	0.939937	0.894274	0.624608
4	6	0	2.317289	1.084472	0.627681
5	6	0	3.157227	0.138699	0.040024
6	6	0	2.615303	-1.004489	-0.545523
7	1	0	0.817784	-2.094504	-1.004617
8	1	0	0.294837	1.626085	1.100479
9	1	0	2.734920	1.970789	1.094914
10	1	0	4.232423	0.290608	0.043194
11	1	0	3.265923	-1.744040	-1.001979
12	6	0	-1.065745	-0.460141	0.013640
13	6	0	-2.055385	0.442629	-0.111225
14	6	0	-1.844203	1.840864	-0.311091
15	7	0	-1.447746	-1.879902	0.048935
16	7	0	-1.705803	2.979841	-0.472305
17	8	0	-2.541458	-2.094113	0.490842
18	1	0	-3.086724	0.099944	-0.087704

7. Optimization of nitrosoalkene [1]3B

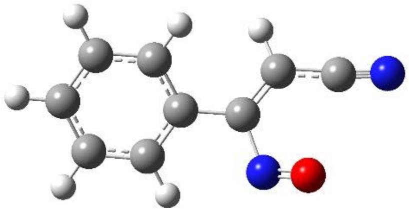

DFT/M062X 6-31+G(d,p), E =-530.976651 a.u.
Standard orientation:

Center Number	Atomic Number	Atomic Type	Coordinates (Angstroms) X	Y	Z
1	6	0	3.019759	0.497469	-0.035027

2	6	0	1.592484	0.916261	-0.048171
3	6	0	0.674271	-0.012749	0.006012
4	1	0	1.402501	1.980903	-0.120805
5	7	0	1.290265	-1.309392	0.031266
6	7	0	4.102045	0.796098	-0.007265
7	8	0	2.498085	-1.241608	0.052939
8	6	0	-0.786734	0.024172	-0.001537
9	6	0	-1.420919	1.256927	0.029296
10	6	0	-2.803784	1.304052	0.033581
11	6	0	-3.534136	0.124116	-0.001867
12	6	0	-2.891087	-1.103485	-0.033597
13	6	0	-1.506552	-1.158011	-0.027841
14	1	0	-0.815863	2.159511	0.073079
15	1	0	-3.313392	2.257538	0.064349
16	1	0	-4.615233	0.162524	-0.006209
17	1	0	-3.467134	-2.018278	-0.064824
18	1	0	-0.981547	-2.108790	-0.062203

8. TD-DFT calculation of 1A

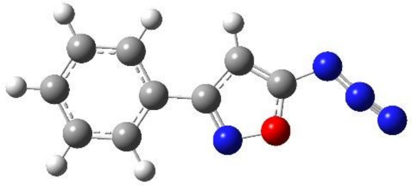

```
Excitation energies and oscillator strengths:

 Excited State   1:   Singlet-A   4.0211 eV  308.33 nm  f=0.0002  <S**2>=0.000
      42 -> 50         0.25481
      47 -> 50        -0.35446
      48 -> 50         0.51089
 This state for optimization and/or second-order correction.
 Total Energy, E(TD-HF/TD-DFT) =  -640.304577638
 Copying the excited state density for this state as the 1-particle RhoCI
density.

 Excited State   2: Singlet-A  5.1535 eV  240.58 nm  f=0.2596  <S**2>=0.000
      40 -> 50         0.11274
      47 -> 49        -0.32783
      47 -> 51        -0.10390
      48 -> 49         0.56208
      48 -> 51         0.11140

 Excited State   3: Singlet-A   5.2344 eV  236.86 nm  f=0.0034  <S**2>=0.000
      46 -> 49         0.39658
      46 -> 51        -0.29185
      47 -> 49         0.18456
      47 -> 53         0.29615
      48 -> 49         0.12330
      48 -> 51         0.20889
      48 -> 53         0.24534

 Excited State   4: Singlet-A  5.4447 eV  227.71 nm  f=0.4930  <S**2>=0.000
```

```
      46 -> 49        -0.17732
      47 -> 49         0.51734
      47 -> 51        -0.17110
      48 -> 49         0.31781
      48 -> 51        -0.20220

 Excited State   5: Singlet-A  5.8191 eV  213.06 nm  f=0.0731  <S**2>=0.000
      40 -> 50         0.11836
      44 -> 49        -0.11856
      46 -> 49        -0.10301
      46 -> 51         0.13464
      47 -> 49         0.19851
      47 -> 51        -0.12950
      48 -> 49        -0.10073
      48 -> 51         0.53992
      48 -> 53        -0.18379

 Excited State   6: Singlet-A  6.0715 eV  204.21 nm  f=0.0012  <S**2>=0.000
      45 -> 49        -0.24950
      47 -> 52        -0.24220
      47 -> 54         0.15027
      48 -> 50         0.13809
      48 -> 52         0.47192
      48 -> 54        -0.25415

 Excited State   7: Singlet-A 6.1226 eV  202.50 nm  f=0.0072  <S**2>=0.000
      45 -> 49         0.59683
      45 -> 64        -0.13745
      47 -> 52        -0.10404
      48 -> 52         0.19939
      48 -> 54        -0.12082

 Excited State   8: Singlet-A 6.2610 eV  198.03 nm  f=0.0299  <S**2>=0.000
      46 -> 49        -0.19040
      46 -> 51         0.14241
      46 -> 53         0.13866
      47 -> 51         0.56263
      48 -> 49         0.16655
      48 -> 51         0.14531

 Excited State   9: Singlet-A  6.3535 eV  195.14 nm  f=0.0001  <S**2>=0.000
      44 -> 50        -0.12637
      47 -> 50         0.55831
      48 -> 50         0.39856

 Excited State  10:  Singlet-A  6.5236 eV  190.05 nm  f=0.3797  <S**2>=0.000
      46 -> 49         0.43318
      46 -> 51         0.16001
      46 -> 53         0.22795
      47 -> 49         0.13239
      47 -> 51         0.12068
      47 -> 53        -0.14027
      48 -> 51        -0.13837
      48 -> 53        -0.37847

 Excited State  11:      Singlet-A      6.6048 eV  187.72 nm  f=0.0031
<S**2>=0.000
```

```
     47 -> 52         0.43877
     47 -> 54         0.19264
     48 -> 52         0.34425
     48 -> 54         0.29626

Excited State  12:   Singlet-A  6.7581 eV  183.46 nm  f=0.0000  <S**2>=0.000
     40 -> 49        -0.23430
     40 -> 51        -0.16392
     42 -> 50        -0.22706
     43 -> 49        -0.13058
     44 -> 50         0.10646
     45 -> 51        -0.19672
     47 -> 50        -0.12646
     47 -> 52        -0.11414
     47 -> 55         0.15225
     48 -> 50         0.13738
     48 -> 54         0.29688
     48 -> 55        -0.19368
     48 -> 57        -0.10026

Excited State  13:   Singlet-A  6.7765 eV  182.96 nm  f=0.0922  <S**2>=0.000
     40 -> 50         0.16021
     45 -> 50         0.14093
     46 -> 49        -0.16744
     47 -> 51         0.13283
     47 -> 53         0.43087
     48 -> 51        -0.17422
     48 -> 53        -0.30011
     48 -> 58        -0.10241
     48 -> 63         0.14170

Excited State  14:   Singlet-A  6.8225 eV  181.73 nm  f=0.4568  <S**2>=0.000
     46 -> 51         0.39052
     46 -> 53         0.36204
     47 -> 51        -0.16937
     47 -> 53         0.22312
     48 -> 53         0.28022

Excited State  15:   Singlet-A   6.8261 eV  181.63 nm   f=0.0010   <S**2>=0.000
     40 -> 49        -0.21475
     40 -> 51        -0.15236
     42 -> 50        -0.11266
     43 -> 49        -0.12894
     45 -> 51        -0.19141
     46 -> 52         0.20449
     46 -> 54         0.17033
     47 -> 52         0.28840
     47 -> 54         0.13016
     47 -> 55        -0.13716
     48 -> 52        -0.12159
     48 -> 54        -0.22898
     48 -> 55         0.12652

Excited State  16:   Singlet-A   6.9219 eV  179.12 nm   f=0.0031   <S**2>=0.000
     46 -> 50         0.11399
     46 -> 52         0.41727
     46 -> 54         0.38077
```

```
     48 -> 55         -0.28425

 Excited State  17: Singlet-A 6.9924 eV  177.31 nm  f=0.0039  <S**2>=0.000
     47 -> 52         -0.21017
     47 -> 54          0.45121
     47 -> 55          0.15473
     48 -> 52         -0.15430
     48 -> 54          0.20554
     48 -> 55          0.22880
     48 -> 56          0.16029
     48 -> 57         -0.12643
     48 -> 59          0.12144

 Excited State  18: Singlet-A 7.0863 eV  174.96 nm  f=0.0456  <S**2>=0.000
     40 -> 50         -0.10699
     42 -> 49         -0.10333
     44 -> 51          0.10998
     46 -> 49         -0.19359
     46 -> 51         -0.29031
     46 -> 53          0.43188
     47 -> 51         -0.15561
     48 -> 53         -0.19728
     48 -> 58          0.10947
     48 -> 63         -0.10542

 Excited State  19: Singlet-A  7.1145 eV  174.27 nm  f=0.0001  <S**2>=0.000
     45 -> 51         -0.10940
     46 -> 50          0.51940
     47 -> 54         -0.12606
     47 -> 55          0.16998
     48 -> 55          0.23049
     48 -> 56         -0.16941
     48 -> 57          0.10251
     48 -> 59         -0.10075

 Excited State  20: Singlet-A  7.1180 eV  174.18 nm  f=0.0000  <S**2>=0.000
     46 -> 50          0.36983
     46 -> 54         -0.14756
     47 -> 54          0.13153
     47 -> 55         -0.29717
     48 -> 55         -0.31825
     48 -> 56          0.22891
     48 -> 57         -0.10306
     48 -> 59          0.12888

 Excited State  21:  Singlet-A 7.2324 eV  171.43 nm  f=0.2460  <S**2>=0.000
     40 -> 50         -0.16958
     42 -> 49         -0.14746
     42 -> 51         -0.11819
     43 -> 50         -0.10153
     45 -> 50         -0.19132
     46 -> 51          0.27513
     46 -> 53         -0.22744
     47 -> 51         -0.10812
     47 -> 53          0.34044
     48 -> 53         -0.16931
     48 -> 63         -0.14984
```

```
 Excited State   22:   Singlet-A  7.2732 eV  170.47 nm  f=0.0006  <S**2>=0.000
      42 -> 50          0.30930
      43 -> 49         -0.21230
      44 -> 50         -0.15687
      45 -> 51         -0.21153
      46 -> 50         -0.21767
      47 -> 52         -0.13814
      47 -> 55         -0.20791
      47 -> 59          0.10930
      48 -> 50         -0.11067
      48 -> 54          0.11006
      48 -> 57          0.21344

 Excited State   23: Singlet-A 7.3182 eV  169.42 nm  f=0.0187  <S**2>=0.000
      42 -> 50          0.23140
      43 -> 49         -0.10194
      44 -> 50         -0.11270
      46 -> 55          0.33969
      46 -> 56         -0.15267
      47 -> 54         -0.10460
      47 -> 55          0.30500
      48 -> 54         -0.10504
      48 -> 56          0.19353
      48 -> 57         -0.16833

 Excited State   24: Singlet-A 7.3495 eV  168.70 nm  f=0.0049  <S**2>=0.000
      42 -> 49          0.10304
      47 -> 58         -0.22953
      47 -> 61          0.11780
      48 -> 58          0.59488
      48 -> 61         -0.13337

 Excited State   25: Singlet-A 7.4020 eV  167.50 nm  f=0.0058  <S**2>=0.000
      42 -> 50         -0.16192
      43 -> 49          0.13743
      43 -> 51         -0.12851
      46 -> 52         -0.10504
      46 -> 55          0.40207
      46 -> 56         -0.15104
      47 -> 55         -0.12298
      47 -> 57         -0.10237
      48 -> 57          0.29675
      48 -> 59         -0.19042
**********************************************************************
```

9. TD-DFT calculation of 1B

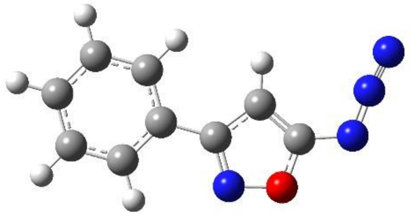

Excitation energies and oscillator strengths:

```
 Excited State   1: Singlet-A 3.9685 eV  312.42 nm  f=0.0003  <S**2>=0.000
      42 -> 50   0.25947
      46 -> 50         0.13979
      47 -> 50        -0.43349
      48 -> 50         0.44093
 This state for optimization and/or second-order correction.
 Total Energy, E(TD-HF/TD-DFT) =  -640.304881009
 Copying the excited state density for this state as the 1-particle RhoCI
density.

 Excited State   2: Singlet-A  5.2378 eV  236.71 nm  f=0.0347  <S**2>=0.000
      46 -> 49         0.36717
      46 -> 51        -0.27681
      47 -> 53        -0.24405
      48 -> 49         0.19328
      48 -> 51         0.24984
      48 -> 53        -0.29902

 Excited State   3: Singlet-A  5.2530 eV  236.03 nm  f=0.2381  <S**2>=0.000
      40 -> 50         0.12775
      43 -> 50         0.12218
      46 -> 51         0.16185
      47 -> 49        -0.41941
      47 -> 51        -0.18078
      48 -> 49         0.42020
      48 -> 51         0.13428
      48 -> 53         0.13362

 Excited State   4: Singlet-A  5.4648 eV  226.88 nm  f=0.4379  <S**2>=0.000
      46 -> 49        -0.13078
      47 -> 49         0.33521
      48 -> 49         0.44071
      48 -> 51        -0.36634

 Excited State   5: Singlet-A 5.7696 eV  214.89 nm  f=0.0075  <S**2>=0.000
      44 -> 49        -0.11759
      46 -> 49        -0.17958
      46 -> 51         0.15825
      47 -> 49         0.38322
      47 -> 51        -0.22308
      48 -> 51         0.40446
      48 -> 53         0.13554

 Excited State   6:    Singlet-A 6.0925 eV  203.50 nm  f=0.0084  <S**2>=0.000
      46 -> 52         0.10191
      47 -> 52        -0.37377
      47 -> 54        -0.14692
      48 -> 52         0.50973
      48 -> 54         0.14268

 Excited State   7:   Singlet-A  6.1898 eV  200.30 nm  f=0.0342  <S**2>=0.000
      46 -> 49        -0.15247
      46 -> 53        -0.11551
      47 -> 51         0.53814
      48 -> 49         0.23855
      48 -> 51         0.23826
```

```
 Excited State   8: Singlet-A 6.2183 eV  199.38 nm  f=0.0026  <S**2>=0.000
      45 -> 49         0.51117
      45 -> 51        -0.10550
      45 -> 61        -0.10414
      45 -> 63         0.10388
      47 -> 50         0.27761
      48 -> 50         0.28926

 Excited State   9: Singlet-A 6.2294 eV  199.03 nm  f=0.0012  <S**2>=0.000
      44 -> 50        -0.10306
      45 -> 49        -0.36324
      47 -> 50         0.40160
      48 -> 50         0.39441

 Excited State  10: Singlet-A  6.5485 eV  189.33 nm  f=0.0036  <S**2>=0.000
      47 -> 52         0.41959
      47 -> 54        -0.12962
      48 -> 52         0.37843
      48 -> 54        -0.33672

 Excited State  11: Singlet-A 6.5497 eV  189.30 nm  f=0.3532  <S**2>=0.000
      46 -> 49         0.44637
      46 -> 53        -0.23263
      47 -> 49         0.15113
      47 -> 51         0.12640
      48 -> 53         0.40373

 Excited State  12: Singlet-A  6.6088 eV  187.60 nm  f=0.0020  <S**2>=0.000
      40 -> 49         0.23680
      40 -> 51         0.23493
      42 -> 50         0.29725
      43 -> 49         0.24301
      43 -> 51         0.22161
      44 -> 50        -0.17103
      45 -> 51        -0.10286
      47 -> 50         0.17401
      47 -> 52        -0.11395
      48 -> 50        -0.18759

 Excited State  13: Singlet-A 6.7675 eV  183.21 nm  f=0.4597  <S**2>=0.000
      46 -> 51         0.41543
      46 -> 53        -0.34994
      47 -> 51        -0.10333
      47 -> 53        -0.21642
      48 -> 51        -0.13080
      48 -> 53        -0.30525

 Excited State  14: Singlet-A  6.8217 eV  181.75 nm  f=0.0031  <S**2>=0.000
      46 -> 50        -0.19365
      46 -> 52         0.35026
      46 -> 54        -0.31477
      47 -> 52         0.25347
      47 -> 55        -0.11856
      48 -> 54         0.32262

 Excited State  15: Singlet-A  6.8346 eV  181.41 nm  f=0.1152  <S**2>=0.000
      40 -> 50        -0.19439
```

```
      42 -> 49        -0.11954
      42 -> 51        -0.12245
      43 -> 50        -0.18991
      46 -> 49         0.17258
      46 -> 51         0.10276
      47 -> 51        -0.15992
      47 -> 53         0.42147
      47 -> 58        -0.11346
      48 -> 51         0.12856
      48 -> 53        -0.18347
      48 -> 58         0.12868
      48 -> 63         0.11386

 Excited State  16:      Singlet-A      6.9245 eV  179.05 nm  f=0.0000
<S**2>=0.000
      46 -> 50         0.39739
      46 -> 52        -0.20229
      46 -> 54         0.12708
      47 -> 52         0.20368
      47 -> 54         0.20143
      47 -> 55        -0.13543
      48 -> 54         0.34615
      48 -> 57         0.12911

 Excited State  17: Singlet-A 6.9542 eV  178.29 nm  f=0.0001  <S**2>=0.000
      40 -> 49        -0.11602
      40 -> 51        -0.11121
      43 -> 49        -0.12623
      43 -> 51        -0.10848
      46 -> 50         0.39425
      46 -> 52         0.24482
      46 -> 54        -0.25435
      47 -> 50         0.11949
      48 -> 55        -0.25086
      48 -> 57        -0.13071

 Excited State  18: Singlet-A 6.9832 eV  177.55 nm  f=0.0028  <S**2>=0.000
      46 -> 50         0.28636
      47 -> 54        -0.31504
      48 -> 52        -0.19476
      48 -> 55         0.44321
      48 -> 56         0.10361

 Excited State  19:   Singlet-A 7.0266 eV  176.45 nm  f=0.0982  <S**2>=0.000
      44 -> 51        -0.12524
      46 -> 49         0.22604
      46 -> 51         0.29923
      46 -> 53         0.41356
      47 -> 51         0.15980
      47 -> 53         0.18178
      48 -> 53        -0.17567

 Excited State  20: Singlet-A 7.1180 eV  174.19 nm  f=0.0003  <S**2>=0.000
      40 -> 51        -0.10164
      42 -> 50         0.22774
      43 -> 49        -0.12717
      44 -> 50        -0.13845
```

```
     46 -> 50        -0.10219
     47 -> 54         0.16905
     47 -> 55         0.17633
     48 -> 55         0.30469
     48 -> 56        -0.29838
     48 -> 57         0.19009
     48 -> 59        -0.14994

Excited State  21: Singlet-A 7.1476 eV  173.46 nm  f=0.0022  <S**2>=0.000
     40 -> 49        -0.12611
     40 -> 51        -0.11626
     42 -> 50         0.35330
     43 -> 49        -0.13686
     43 -> 51        -0.11485
     44 -> 50        -0.23080
     46 -> 50        -0.16561
     46 -> 54         0.14168
     47 -> 54        -0.18317
     47 -> 55        -0.16680
     48 -> 56         0.23502
     48 -> 57        -0.10870
     48 -> 59         0.11174

Excited State  22: Singlet-A 7.1987 eV  172.23 nm  f=0.0604  <S**2>=0.000
     40 -> 50         0.18417
     42 -> 49         0.14446
     42 -> 51         0.15164
     43 -> 50         0.18601
     44 -> 49        -0.14308
     46 -> 51        -0.24223
     46 -> 53        -0.26357
     47 -> 53         0.38072
     48 -> 53        -0.15472

Excited State  23: Singlet-A 7.3195 eV  169.39 nm  f=0.0151  <S**2>=0.000
     46 -> 52        -0.27812
     46 -> 54        -0.23187
     46 -> 55         0.36886
     46 -> 57        -0.13008
     47 -> 55         0.33611
     48 -> 54         0.13237
     48 -> 57        -0.13286

Excited State  24:  Singlet-A 7.3670 eV  168.30 nm  f=0.0030  <S**2>=0.000
     46 -> 52        -0.10767
     46 -> 54        -0.17812
     46 -> 55         0.26596
     47 -> 55        -0.23773
     47 -> 56         0.10333
     47 -> 57        -0.14382
     47 -> 59         0.12303
     48 -> 55        -0.17071
     48 -> 57         0.39926
     48 -> 59        -0.13388

Excited State  25: Singlet-A 7.4004 eV  167.54 nm  f=0.0205  <S**2>=0.000
     47 -> 58        -0.25076
```

```
      47 -> 61         -0.10079
      48 -> 58          0.59087
*****************************************************************************
```

10. TD-DFT calculation of nitrene ^{3}2

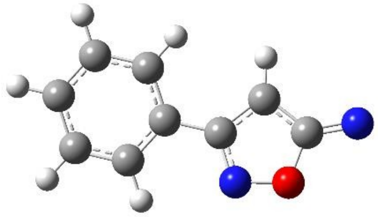

```
Excitation energies and oscillator strengths:

 Excited State   1:  3.109-A  2.9341 eV  422.57 nm  f=0.0095  <S**2>=2.166
     42A -> 43A         0.25524
     36B -> 41B         0.29641
     37B -> 41B        -0.16479
     40B -> 41B         0.87744
 This state for optimization and/or second-order correction.
 Total Energy, E(TD-HF/TD-DFT) =  -530.847524513
 Copying the excited state density for this state as the 1-particle RhoCI
density.

 Excited State   2:  3.042-A  3.4355 eV  360.89 nm  f=0.0084  <S**2>=2.063
     29B -> 42B        -0.10797
     32B -> 42B         0.11813
     33B -> 42B         0.83347
     35B -> 42B        -0.31160
     36B -> 42B        -0.14825
     37B -> 42B        -0.22128
     38B -> 41B         0.13904

 Excited State   3:  3.035-A 3.4762 eV  356.67 nm  f=0.0003  <S**2>=2.053
     26B -> 42B        -0.10759
     36B -> 42B        -0.19522
     37B -> 41B         0.15154
     38B -> 42B         0.90701
     40B -> 42B        -0.23545

 Excited State   4:  3.130-A  3.5892 eV  345.43 nm  f=0.0494  <S**2>=2.200
     42A -> 43A        -0.26430
     42A -> 50A        -0.15561
     42A -> 51A        -0.11941
     33B -> 42B        -0.13033
     37B -> 41B         0.11736
     38B -> 41B         0.84675
     39B -> 41B        -0.10419
     40B -> 41B         0.11855
     40B -> 43B         0.16869

 Excited State   5:  3.173-A 3.6423 eV  340.40 nm  f=0.0038  <S**2>=2.267
     33B -> 41B        -0.14005
```

35B -> 41B 0.24964
36B -> 41B 0.43610
36B -> 43B -0.10410
37B -> 41B 0.74320
37B -> 43B -0.17761
38B -> 41B -0.12188
38B -> 42B -0.17311

Excited State 6: 3.996-A 4.0038 eV 309.66 nm f=0.0093
<S**2>=3.742
40A -> 44A -0.24758
41A -> 43A -0.50686
42A -> 43A -0.29158
38B -> 41B -0.30701
39B -> 41B -0.10766
39B -> 44B -0.24403
40B -> 41B 0.14105
40B -> 43B 0.57611

Excited State 7: 3.090-A 4.2209 eV 293.74 nm f=0.0002 <S**2>=2.137
36B -> 42B 0.24315
37B -> 42B -0.11551
38B -> 42B 0.29418
40B -> 42B 0.89851

Excited State 8: 3.146-A 4.3291 eV 286.40 nm f=0.0027 <S**2>=2.224
41A -> 43A -0.10443
42A -> 43A -0.14506
39B -> 41B 0.95293
39B -> 44B 0.10958

Excited State 9: 3.098-A 4.3831 eV 282.87 nm f=0.0026 <S**2>=2.149
33B -> 41B 0.85775
34B -> 41B 0.14337
35B -> 41B -0.24339
37B -> 41B 0.20400
37B -> 43B -0.10083

Excited State 10: 4.097-A 4.6988 eV 263.87 nm f=0.0001 <S**2>=3.947
40A -> 43A 0.71485
40A -> 51A -0.11597
41A -> 43A 0.12654
41A -> 44A 0.14668
42A -> 43A -0.13421
39B -> 43B 0.51267
40B -> 44B -0.29962

Excited State 11: 3.430-A 4.7615 eV 260.39 nm f=0.0144 <S**2>=2.691
40A -> 44A -0.31041
41A -> 43A -0.22278
42A -> 43A 0.55284
42A -> 50A 0.12849
42A -> 51A 0.11316
32B -> 41B 0.11165
36B -> 41B 0.27039
37B -> 41B -0.10784
38B -> 41B 0.22648

```
     39B -> 41B         0.19033
     39B -> 44B        -0.30940
     40B -> 41B        -0.32548
     40B -> 44B        -0.14120

 Excited State  12:  3.931-A  4.8989 eV  253.08 nm  f=0.0042  <S**2>=3.612
     40A -> 44A         0.52228
     41A -> 43A        -0.36579
     42A -> 43A         0.19252
     42A -> 44A        -0.11926
     36B -> 41B         0.16287
     39B -> 41B        -0.12735
     39B -> 44B         0.51605
     39B -> 45B         0.11754
     40B -> 41B        -0.15625
     40B -> 43B         0.30023

 Excited State  13:  4.067-A 5.1215 eV  242.08 nm  f=0.0060  <S**2>=3.885
     40A -> 43A         0.24819
     41A -> 44A        -0.46603
     42A -> 43A         0.15514
     42A -> 44A        -0.26043
     36B -> 44B        -0.10688
     39B -> 43B         0.30099
     40B -> 44B         0.65939
     40B -> 45B         0.14432

 Excited State  14:  3.136-A  5.2962 eV  234.10 nm  f=0.0014  <S**2>=2.208
     40A -> 43A        -0.37275
     41A -> 43A        -0.11291
     41A -> 44A         0.46403
     42A -> 43A         0.17590
     42A -> 44A         0.26602
     36B -> 41B        -0.21415
     37B -> 41B         0.11676
     39B -> 42B         0.16983
     39B -> 43B         0.57609
     40B -> 44B         0.22667

 Excited State  15:  3.131-A  5.3742 eV  230.70 nm  f=0.0299  <S**2>=2.200
     40A -> 43A        -0.17798
     41A -> 43A         0.17251
     42A -> 43A        -0.26171
     32B -> 41B         0.18813
     36B -> 41B         0.55792
     37B -> 41B        -0.31237
     37B -> 42B        -0.16382
     39B -> 42B         0.49950
     40B -> 41B        -0.17646
     40B -> 44B         0.17657

 Excited State  16:  3.101-A 5.4134 eV  229.03 nm  f=0.0112  <S**2>=2.155
     40A -> 43A         0.14066
     42A -> 43A         0.15612
     36B -> 41B        -0.29017
     37B -> 41B         0.16212
     39B -> 42B         0.83969
```

```
     39B -> 43B        -0.20893
     40B -> 44B        -0.16868

 Excited State  17:   3.058-A   5.5889 eV  221.84 nm  f=0.4112  <S**2>=2.088
     40A -> 44A         0.12244
     41A -> 43A         0.61399
     42A -> 43A         0.27396
     39B -> 44B        -0.15239
     40B -> 43B         0.68308

 Excited State  18:   3.151-A   5.9481 eV  208.44 nm  f=0.0088  <S**2>=2.232
     38A -> 43A         0.10013
     41A -> 43A         0.10696
     42A -> 43A        -0.22270
     42A -> 44A        -0.10821
     42A -> 50A         0.24199
     42A -> 51A         0.14132
     42A -> 58A         0.11069
     33B -> 42B         0.26522
     34B -> 42B         0.11277
     36B -> 42B         0.26748
     37B -> 42B         0.72960

 Excited State  19:   3.570-A   6.1143 eV  202.78 nm  f=0.0015  <S**2>=2.936
     35A -> 43A         0.24689
     37A -> 43A        -0.21642
     39A -> 43A         0.78500
     39A -> 50A         0.26644
     39A -> 51A         0.21090
     37B -> 41B         0.14787
     37B -> 43B         0.12004

 Excited State  20:   4.008-A   6.1963 eV  200.09 nm  f=0.0062  <S**2>=3.766
     38A -> 43A         0.50701
     41A -> 43A         0.11411
     41A -> 50A         0.21043
     41A -> 51A         0.21767
     41A -> 54A         0.10801
     41A -> 56A        -0.10325
     42A -> 50A         0.12163
     42A -> 51A         0.11878
     36B -> 41B        -0.17599
     36B -> 43B        -0.39459
     37B -> 42B        -0.16243
     37B -> 43B         0.23180
     40B -> 54B         0.29711
     40B -> 55B        -0.19246

 Excited State  21:   3.110-A   6.3105 eV  196.47 nm  f=0.0002  <S**2>=2.168
     29B -> 41B         0.25435
     31B -> 41B         0.64966
     33B -> 41B         0.20314
     34B -> 41B        -0.58524
     35B -> 41B         0.24997

 Excited State  22:   3.123-A   6.3809 eV  194.30 nm  f=0.0020  <S**2>=2.188
     42A -> 45A        -0.16644
```

```
     26B -> 42B         0.10552
     32B -> 42B         0.20654
     36B -> 42B         0.76541
     37B -> 42B        -0.34381
     38B -> 42B         0.14182
     40B -> 42B        -0.31698
     40B -> 45B         0.18236

 Excited State  23:  3.099-A   6.4021 eV  193.66 nm  f=0.0205  <S**2>=2.151
     41A -> 44A         0.10605
     41A -> 45A        -0.23403
     41A -> 46A         0.17155
     42A -> 44A        -0.37002
     42A -> 45A         0.63864
     42A -> 46A        -0.35649
     42A -> 47A         0.26886
     42A -> 48A         0.14836
     36B -> 42B         0.13366
     37B -> 42B        -0.12643

 Excited State  24:  3.114-A   6.5162 eV  190.27 nm  f=0.2511  <S**2>=2.175
     40A -> 43A         0.23301
     41A -> 45A        -0.11458
     41A -> 50A         0.13829
     42A -> 44A         0.57145
     42A -> 45A         0.28859
     42A -> 46A        -0.12501
     42A -> 50A        -0.23286
     42A -> 51A        -0.13411
     42A -> 54A         0.10994
     36B -> 42B         0.21897
     37B -> 42B         0.16384
     38B -> 41B        -0.13452
     39B -> 43B        -0.25642
     40B -> 44B         0.28448

 Excited State  25:  3.469-A   6.5862 eV  188.25 nm  f=0.1032  <S**2>=2.758
     40A -> 43A        -0.14860
     41A -> 44A        -0.28707
     42A -> 50A        -0.15068
     37B -> 42B         0.17153
     39B -> 43B         0.17526
     40B -> 44B        -0.35797
     40B -> 45B         0.73465
     40B -> 46B         0.15194
     40B -> 51B        -0.12588
*******************************************************************
```

11. TD-DFT calculation of nitrosoalkene ^{3}3A

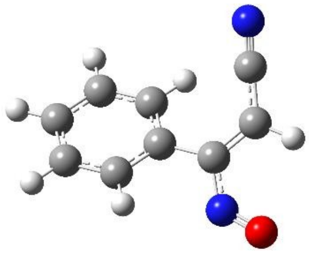

```
Excitation energies and oscillator strengths:

 Excited State   1:  3.027-A  2.4967 eV  496.60 nm  f=0.0003  <S**2>=2.041
     26B -> 41B       -0.13995
     28B -> 41B        0.12882
     32B -> 41B       -0.14117
     34B -> 41B        0.16568
     38B -> 41B        0.47182
     40B -> 41B        0.80214
     40B -> 42B        0.12422
 This state for optimization and/or second-order correction.
 Total Energy, E(TD-HF/TD-DFT) =  -530.866506784
 Copying the excited state density for this state as the 1-particle RhoCI
density.

 Excited State   2:  3.186-A  2.7825 eV  445.59 nm  f=0.0247  <S**2>=2.288
     42A -> 43A       -0.39075
     42A -> 48A       -0.17237
     38B -> 42B        0.32980
     40B -> 41B       -0.12602
     40B -> 42B        0.80953

 Excited State   3:  3.668-A  4.0206 eV  308.37 nm  f=0.0071  <S**2>=3.113
     40A -> 44A       -0.21265
     41A -> 43A        0.44366
     41A -> 48A       -0.11583
     42A -> 43A       -0.28726
     42A -> 48A       -0.10452
     32B -> 41B        0.14444
     38B -> 41B       -0.33473
     38B -> 42B        0.23828
     39B -> 41B       -0.26518
     39B -> 44B        0.20854
     40B -> 41B        0.26164
     40B -> 42B       -0.17279
     40B -> 43B       -0.39184

 Excited State   4:  3.338-A  4.1599 eV  298.05 nm  f=0.0015  <S**2>=2.536
     40A -> 43A        0.20210
     42A -> 43A        0.13162
     37B -> 42B       -0.10665
     38B -> 41B       -0.10434
     38B -> 42B       -0.10131
     39B -> 41B       -0.58120
     39B -> 42B        0.67797
     39B -> 43B        0.20161
```

```
     40B -> 42B          0.10430

 Excited State   5:   3.209-A   4.2473 eV  291.91 nm  f=0.0069  <S**2>=2.324
     41A -> 43A          0.22364
     42A -> 43A         -0.24452
     26B -> 41B         -0.16868
     28B -> 41B          0.13362
     32B -> 41B         -0.20466
     38B -> 41B          0.45057
     38B -> 42B          0.25329
     39B -> 41B          0.15850
     39B -> 42B          0.43261
     39B -> 43B          0.10213
     40B -> 41B         -0.37393
     40B -> 42B         -0.25114
     40B -> 43B         -0.14684

 Excited State   6:   3.159-A   4.3020 eV  288.20 nm  f=0.0124  <S**2>=2.246
     40A -> 44A          0.14834
     42A -> 43A         -0.16919
     26B -> 41B          0.10943
     32B -> 41B          0.11169
     38B -> 41B         -0.32569
     39B -> 41B          0.66955
     39B -> 42B          0.44835
     39B -> 44B         -0.14183
     40B -> 41B          0.31175

 Excited State   7:   3.394-A   4.4126 eV  280.98 nm  f=0.0894  <S**2>=2.629
     40A -> 44A         -0.18905
     41A -> 43A          0.37867
     42A -> 43A          0.58056
     42A -> 48A          0.13978
     38B -> 42B         -0.14263
     39B -> 41B          0.26629
     39B -> 42B          0.11137
     39B -> 44B          0.16036
     40B -> 42B          0.42227
     40B -> 43B         -0.28789

 Excited State   8:   3.707-A 4.7884 eV  258.93 nm  f=0.0024  <S**2>=3.185
     40A -> 43A          0.65821
     40A -> 44A         -0.11999
     40A -> 48A         -0.15832
     42A -> 43A         -0.10669
     37B -> 42B          0.41001
     38B -> 42B         -0.18800
     39B -> 41B          0.10231
     39B -> 42B         -0.16047
     39B -> 43B          0.29987
     40B -> 44B         -0.25158

 Excited State   9:   3.344-A   4.8235 eV  257.04 nm  f=0.0438  <S**2>=2.546
     40A -> 44A          0.25007
     42A -> 43A          0.31656
     31B -> 41B         -0.14152
     35B -> 42B          0.10458
```

```
     37B -> 41B        -0.23722
     37B -> 42B         0.61266
     38B -> 42B         0.41161
     39B -> 42B         0.10547
     39B -> 44B        -0.22991

 Excited State  10:  3.665-A      4.8910 eV  253.49 nm  f=0.0192
<S**2>=3.109
     40A -> 43A        -0.36934
     40A -> 44A        -0.37737
     40A -> 45A         0.11922
     40A -> 48A         0.10935
     41A -> 43A        -0.14572
     42A -> 43A        -0.15190
     37B -> 42B         0.44634
     38B -> 42B        -0.26586
     39B -> 42B         0.25192
     39B -> 44B         0.35722
     39B -> 45B        -0.14970
     40B -> 43B         0.15317
     40B -> 44B         0.19462

 Excited State  11:  3.518-A  4.9557 eV  250.18 nm  f=0.0651  <S**2>=2.843
     40A -> 43A         0.12580
     40A -> 44A        -0.31435
     40A -> 45A         0.10553
     41A -> 43A        -0.36535
     42A -> 43A         0.27658
     31B -> 41B        -0.11771
     32B -> 42B        -0.10543
     37B -> 42B        -0.16559
     38B -> 41B        -0.10974
     38B -> 42B         0.54176
     39B -> 41B         0.14144
     39B -> 44B         0.33224
     39B -> 45B        -0.12904
     40B -> 42B        -0.12740
     40B -> 43B         0.23051

 Excited State  12:  4.078-A  5.2032 eV  238.28 nm  f=0.0019  <S**2>=3.907
     40A -> 43A         0.30311
     41A -> 44A         0.61090
     41A -> 45A        -0.19437
     38B -> 44B        -0.16166
     39B -> 43B         0.11151
     40B -> 44B         0.58676
     40B -> 45B        -0.21273

 Excited State  13:  3.290-A  5.3403 eV  232.17 nm  f=0.0222  <S**2>=2.456
     37A -> 53A        -0.10684
     38A -> 43A         0.13669
     41A -> 43A         0.11642
     42A -> 43A         0.10031
     24B -> 41B         0.11306
     26B -> 42B         0.11767
     29B -> 41B        -0.20422
     29B -> 42B        -0.10754
```

```
     31B -> 41B         0.52835
     31B -> 42B         0.19224
     32B -> 42B         0.12411
     33B -> 41B         0.10736
     34B -> 42B        -0.32081
     35B -> 41B        -0.20206
     37B -> 41B         0.29149
     38B -> 42B         0.15603
     38B -> 43B         0.17521
     38B -> 52B         0.10106
     40B -> 43B         0.14968
     40B -> 51B        -0.11545
     40B -> 52B         0.12086

 Excited State  14:  3.195-A 5.4938 eV  225.68 nm  f=0.0008  <S**2>=2.303
     40A -> 43A        -0.22115
     41A -> 44A        -0.40663
     41A -> 45A         0.12344
     31B -> 42B        -0.11459
     39B -> 42B        -0.14245
     39B -> 43B         0.69913
     40B -> 44B         0.37217
     40B -> 45B        -0.12385

 Excited State  15:  3.135-A  5.5505 eV  223.37 nm  f=0.0005  <S**2>=2.207
     39A -> 43A        -0.13977
     26B -> 41B         0.26042
     28B -> 41B        -0.20174
     29B -> 42B        -0.25096
     31B -> 42B         0.53046
     31B -> 43B        -0.17618
     31B -> 52B        -0.11310
     32B -> 42B        -0.10940
     33B -> 42B         0.25967
     34B -> 41B        -0.11743
     35B -> 42B        -0.28236
     38B -> 41B         0.33920
     39B -> 43B         0.13864

 Excited State  16:  3.296-A  5.6425 eV  219.73 nm  f=0.0147  <S**2>=2.465
     38A -> 53A        -0.20839
     39A -> 43A        -0.22766
     41A -> 43A        -0.15090
     41A -> 53A         0.11339
     42A -> 44A        -0.30667
     42A -> 45A         0.21958
     42A -> 46A        -0.27858
     42A -> 48A        -0.17066
     42A -> 49A        -0.20787
     42A -> 50A         0.12854
     42A -> 51A         0.12655
     42A -> 53A         0.57870
     42A -> 54A         0.17948
     40B -> 43B        -0.17698

 Excited State  17:  3.207-A  5.6912 eV  217.85 nm  f=0.2352  <S**2>=2.321
     38A -> 43A        -0.12173
```

```
     39A -> 43A        -0.11515
     40A -> 44A        -0.15212
     41A -> 43A         0.57303
     41A -> 48A         0.15651
     42A -> 53A         0.16244
     40B -> 43B         0.66697

 Excited State  18:  3.127-A  5.8026 eV  213.67 nm  f=0.0015  <S**2>=2.195
     37A -> 43A        -0.10086
     39A -> 43A         0.83048
     39A -> 48A         0.28630
     42A -> 44A        -0.12243
     42A -> 53A         0.17369

 Excited State  19:  3.344-A  5.8143 eV  213.24 nm  f=0.0590  <S**2>=2.545
     37A -> 53A 0.13637
     38A -> 43A        -0.15755
     39A -> 43A        -0.15230
     42A -> 48A         0.14612
     24B -> 41B         0.12760
     29B -> 41B        -0.11545
     31B -> 41B         0.40676
     32B -> 42B        -0.18307
     33B -> 42B        -0.11269
     34B -> 41B        -0.17217
     34B -> 42B         0.45639
     35B -> 41B        -0.21459
     35B -> 42B        -0.17151
     37B -> 54B        -0.11971
     38B -> 41B        -0.18674
     38B -> 42B         0.18513

 Excited State  20:  3.091-A  5.9447 eV  208.56 nm  f=0.0051  <S**2>=2.139
     42A -> 44A         0.77067
     42A -> 45A         0.38644
     42A -> 46A        -0.43453

 Excited State  21:  3.287-A  6.0644 eV  204.45 nm  f=0.0022  <S**2>=2.451
     42A -> 44A        -0.10877
     26B -> 41B        -0.19433
     28B -> 41B         0.15716
     29B -> 41B         0.11599
     33B -> 41B        -0.19813
     33B -> 42B         0.67286
     33B -> 51B        -0.11686
     33B -> 52B         0.13503
     34B -> 42B         0.12612
     37B -> 41B         0.38030
     38B -> 41B        -0.20346

 Excited State  22:  3.463-A   6.1067 eV  203.03 nm  f=0.0034  <S**2>=2.748
     37A -> 53A        -0.12813
     38A -> 43A        -0.17814
     41A -> 48A         0.17463
     42A -> 48A        -0.11790
     29B -> 41B         0.11530
     31B -> 41B        -0.11648
```

```
     33B -> 42B       -0.40865
     37B -> 41B        0.60202
     37B -> 42B        0.16090
     37B -> 54B        0.16569
     38B -> 43B       -0.22072
     40B -> 51B        0.13144
     40B -> 52B       -0.13076

 Excited State  23:  3.617-A   6.1867 eV  200.41 nm  f=0.0129  <S**2>=3.021
     33A -> 43A        0.10645
     38A -> 43A        0.33728
     41A -> 48A       -0.29834
     42A -> 48A       -0.13160
     26B -> 41B       -0.13214
     31B -> 41B       -0.18754
     31B -> 42B        0.18491
     33B -> 42B       -0.20897
     34B -> 42B        0.28407
     35B -> 41B        0.10888
     35B -> 42B       -0.31878
     37B -> 41B        0.14813
     38B -> 43B        0.28898
     40B -> 43B        0.20571
     40B -> 51B       -0.19775
     40B -> 52B        0.19319

 Excited State  24:  3.157-A      6.2875 eV  197.19 nm  f=0.0045
<S**2>=2.242
     38A -> 53A        0.11912
     42A -> 44A       -0.48478
     42A -> 45A        0.58764
     42A -> 46A       -0.31411
     42A -> 49A        0.12117
     42A -> 53A       -0.40880
     42A -> 54A       -0.11283

 Excited State  25:  3.210-A  6.3096 eV  196.50 nm  f=0.0050  <S**2>=2.326
     38A -> 43A       -0.15763
     39A -> 43A        0.13218
     41A -> 48A        0.12292
     26B -> 41B       -0.32960
     28B -> 41B        0.24838
     29B -> 42B       -0.10345
     31B -> 42B        0.38389
     32B -> 41B       -0.19919
     33B -> 42B       -0.14926
     34B -> 41B        0.25188
     34B -> 42B       -0.29143
     35B -> 41B       -0.13263
     35B -> 42B       -0.24203
     36B -> 41B        0.13042
     37B -> 41B       -0.21833
     38B -> 41B       -0.25010

**********************************************************************
```

12. TD-DFT calculation of nitrosoalkene ^{3}3B

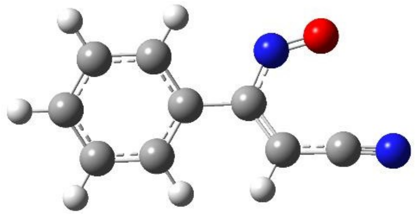

```
Excitation energies and oscillator strengths:


 Excited State   1:  3.025-A  2.6270 eV  471.97 nm  f=0.0005  <S**2>=2.037
     27B -> 42B       -0.12836
     28B -> 42B       -0.13685
     32B -> 42B        0.11788
     34B -> 42B       -0.19478
     38B -> 41B        0.13005
     38B -> 42B        0.44369
     40B -> 41B        0.25402
     40B -> 42B        0.78387
 This state for optimization and/or second-order correction.
 Total Energy, E(TD-HF/TD-DFT) =  -530.868841743
 Copying the excited state density for this state as the 1-particle RhoCI
density.

 Excited State   2:  3.181-A  2.8014 eV  442.58 nm  f=0.0255  <S**2>=2.280
     42A -> 43A        0.42136
     42A -> 48A       -0.17554
     38B -> 41B        0.29748
     38B -> 42B       -0.11038
     40B -> 41B        0.76875
     40B -> 42B       -0.25082

 Excited State   3:  3.773-A  4.0332 eV  307.41 nm  f=0.0134  <S**2>=3.309
     40A -> 44A       -0.23572
     41A -> 43A        0.52052
     42A -> 43A       -0.40103
     42A -> 48A        0.12520
     38B -> 41B       -0.26090
     38B -> 43B       -0.10494
     39B -> 42B        0.10807
     39B -> 44B        0.23229
     40B -> 41B        0.27526
     40B -> 43B        0.43737

 Excited State   4:  3.326-A 4.3378 eV  285.82 nm  f=0.1154  <S**2>=2.515
     40A -> 44A       -0.21192
     41A -> 43A        0.25126
     42A -> 43A        0.67109
     42A -> 48A       -0.14864
     37B -> 42B        0.12573
     39B -> 41B       -0.14002
     39B -> 42B        0.16192
     39B -> 44B        0.20087
```

```
    40B -> 41B       -0.40492
    40B -> 42B        0.16160
    40B -> 43B        0.24641

Excited State   5:  3.474-A  4.4015 eV  281.69 nm  f=0.0011  <S**2>=2.767
    40A -> 43A        0.30865
    41A -> 44A       -0.10717
    32B -> 42B       -0.10595
    38B -> 42B       -0.19732
    39B -> 41B        0.80104
    39B -> 43B        0.25517
    40B -> 42B        0.19298

Excited State   6:  3.190-A 4.4958 eV  275.78 nm  f=0.0039  <S**2>=2.294
    41A -> 43A        0.22254
    27B -> 42B       -0.21714
    28B -> 42B       -0.17668
    31B -> 41B       -0.12042
    32B -> 42B        0.23649
    34B -> 42B       -0.16930
    38B -> 42B        0.50093
    39B -> 41B        0.29893
    39B -> 42B       -0.30957
    39B -> 43B        0.12933
    40B -> 41B       -0.16212
    40B -> 42B       -0.43330
    40B -> 43B        0.12820

Excited State   7:  3.332-A  4.6437 eV  266.99 nm  f=0.0021  <S**2>=2.525
    40A -> 44A       -0.24051
    41A -> 43A       -0.22185
    38B -> 41B        0.11078
    38B -> 42B        0.18643
    39B -> 42B        0.80801
    39B -> 44B        0.23800
    40B -> 42B       -0.18665
    40B -> 43B       -0.14860

Excited State   8:  3.267-A  4.7258 eV  262.35 nm  f=0.0002  <S**2>=2.419
    40A -> 43A        0.16210
    37B -> 41B        0.89920
    37B -> 42B       -0.19206
    37B -> 52B       -0.15555

Excited State   9:  3.687-A 4.8409 eV  256.12 nm  f=0.0030  <S**2>=3.149
    40A -> 43A        0.50234
    40A -> 44A       -0.26990
    40A -> 48A        0.11295
    41A -> 44A       -0.17851
    42A -> 43A       -0.14752
    37B -> 41B       -0.16567
    38B -> 41B        0.30575
    38B -> 44B       -0.11876
    39B -> 41B       -0.29523
    39B -> 42B       -0.17305
    39B -> 43B        0.18451
    39B -> 44B        0.24082
```

```
     40B -> 44B        0.42210

 Excited State  10:  3.728-A 4.9387 eV  251.05 nm  f=0.0076  <S**2>=3.224
     40A -> 43A        0.34487
     40A -> 44A        0.45987
     41A -> 43A        0.20766
     38B -> 42B        0.10652
     39B -> 41B       -0.24339
     39B -> 42B        0.38737
     39B -> 44B       -0.44777
     40B -> 43B        0.21517
     40B -> 44B        0.21868

 Excited State  11:  3.299-A 5.0477 eV  245.62 nm  f=0.0328  <S**2>=2.471
     40A -> 43A       -0.29587
     40A -> 44A        0.11400
     41A -> 43A        0.26931
     42A -> 43A       -0.19996
     31B -> 42B       -0.12185
     32B -> 41B        0.12587
     34B -> 41B        0.13601
     38B -> 41B        0.68470
     38B -> 42B       -0.17803
     39B -> 41B        0.12786
     39B -> 42B        0.11089
     39B -> 43B       -0.12976
     39B -> 44B       -0.14659
     40B -> 41B       -0.20649
     40B -> 43B        0.14053

 Excited State  12:  3.967-A  5.1483 eV  240.83 nm  f=0.0036  <S**2>=3.684
     38A -> 44A        0.10530
     40A -> 43A        0.40542
     41A -> 44A        0.60937
     38B -> 41B        0.17524
     38B -> 44B        0.15801
     39B -> 41B       -0.11060
     39B -> 43B        0.12434
     40B -> 44B       -0.51674

 Excited State  13:  3.295-A 5.3350 eV  232.40 nm  f=0.0141  <S**2>=2.465
     38A -> 43A       -0.13912
     41A -> 43A       -0.15354
     25B -> 42B        0.10864
     29B -> 42B        0.11796
     31B -> 42B        0.53915
     32B -> 41B       -0.14435
     34B -> 41B        0.44272
     34B -> 42B       -0.13982
     35B -> 41B        0.13294
     35B -> 42B        0.22447
     37B -> 42B        0.10534
     38B -> 43B        0.17704
     38B -> 52B       -0.11664
     40B -> 43B        0.14341
     40B -> 52B       -0.15942
```

```
 Excited State  14:  3.159-A5.4370 eV  228.04 nm  f=0.0030  <S**2>=2.244
     39A -> 43A        0.67247
     39A -> 48A       -0.24744
     42A -> 44A        0.12905
     42A -> 45A       -0.17175
     42A -> 46A       -0.13365
     42A -> 52A       -0.18832
     42A -> 53A        0.14648
     42A -> 54A       -0.22809
     42A -> 55A       -0.13802
     31B -> 41B       -0.22197
     31B -> 42B        0.18574
     35B -> 41B       -0.12263
     38B -> 42B       -0.14508

 Excited State  15:  3.181-A 5.5125 eV  224.91 nm  f=0.0013  <S**2>=2.280
     40A -> 43A       -0.11750
     41A -> 44A       -0.36320
     42A -> 44A       -0.11933
     42A -> 54A        0.10003
     31B -> 41B       -0.23914
     35B -> 41B       -0.16394
     38B -> 42B       -0.20214
     39B -> 41B       -0.18810
     39B -> 43B        0.59810
     39B -> 52B        0.10281
     40B -> 44B       -0.38257

 Excited State  16:  3.147-A 5.5424 eV  223.70 nm  f=0.0006  <S**2>=2.226
     39A -> 43A        0.13520
     41A -> 44A       -0.27349
     42A -> 44A        0.11633
     42A -> 45A       -0.18814
     42A -> 46A       -0.13603
     42A -> 52A       -0.17589
     42A -> 53A        0.14069
     42A -> 54A       -0.20482
     42A -> 55A       -0.12362
     27B -> 42B        0.12937
     28B -> 42B        0.12115
     29B -> 41B        0.11766
     31B -> 41B        0.38000
     31B -> 42B       -0.15438
     31B -> 43B       -0.14255
     31B -> 52B        0.10098
     32B -> 41B       -0.11921
     34B -> 41B        0.14692
     34B -> 42B        0.10257
     35B -> 41B        0.26567
     38B -> 42B        0.29809
     39B -> 41B       -0.13467
     39B -> 43B        0.33005
     40B -> 44B       -0.14530

 Excited State  17:  3.185-A  5.5553 eV  223.18 nm  f=0.0023  <S**2>=2.286
     37A -> 43A       -0.10376
     39A -> 43A        0.55820
```

```
     39A -> 48A        -0.18838
     42A -> 44A        -0.20927
     42A -> 45A         0.28045
     42A -> 46A         0.20846
     42A -> 48A        -0.12544
     42A -> 52A         0.25477
     42A -> 53A        -0.18281
     42A -> 54A         0.29539
     42A -> 55A         0.17217
     31B -> 41B         0.11849
     31B -> 42B        -0.10715
     34B -> 42B         0.12304
     37B -> 42B         0.11705
     38B -> 42B         0.21067

 Excited State  18:  3.192-A  5.6753 eV  218.46 nm  f=0.2340  <S**2>=2.298
     38A -> 43A         0.10674
     40A -> 44A         0.12996
     41A -> 43A        -0.52588
     41A -> 48A         0.10189
     42A -> 44A        -0.14358
     31B -> 41B         0.11836
     34B -> 41B        -0.14908
     37B -> 42B        -0.17540
     38B -> 41B         0.15480
     39B -> 44B         0.11313
     40B -> 43B         0.66724

 Excited State  19:  3.260-A 5.7537 eV  215.48 nm  f=0.1026  <S**2>=2.407
     37A -> 54A         0.10907
     41A -> 43A        -0.18693
     42A -> 44A        -0.14785
     42A -> 45A        -0.11535
     42A -> 48A        -0.12356
     31B -> 41B        -0.19365
     31B -> 42B        -0.35347
     33B -> 41B        -0.11397
     34B -> 41B         0.28695
     35B -> 41B        -0.19506
     35B -> 42B        -0.22843
     36B -> 41B         0.12077
     37B -> 42B         0.45003
     37B -> 55B        -0.12435
     38B -> 41B        -0.16617
     40B -> 43B         0.17500

 Excited State  20:  3.093-A  5.8234 eV  212.91 nm  f=0.0474  <S**2>=2.141
     41A -> 43A        -0.14137
     42A -> 44A         0.79709
     42A -> 45A         0.35534
     42A -> 46A         0.25374
     42A -> 47A        -0.19165
     42A -> 50A         0.13959
     40B -> 43B         0.15582

 Excited State  21:  3.283-A  5.9012 eV  210.10 nm  f=0.0060  <S**2>=2.444
     33B -> 41B         0.63669
```

```
     33B -> 42B        -0.13177
     33B -> 52B        -0.13657
     34B -> 41B        -0.28638
     35B -> 41B         0.38619
     37B -> 42B         0.36742
     38B -> 42B        -0.13823

 Excited State  22:  3.154-A 5.9539 eV  208.24 nm  f=0.0337  <S**2>=2.237
     42A -> 43A        -0.15382
     42A -> 44A         0.10281
     27B -> 42B         0.13413
     31B -> 42B         0.19205
     32B -> 42B        -0.12248
     33B -> 41B        -0.24704
     34B -> 41B        -0.37602
     34B -> 42B         0.19969
     35B -> 41B        -0.18885
     35B -> 42B         0.27297
     37B -> 41B         0.13222
     37B -> 42B         0.56471
     38B -> 41B         0.21235
     38B -> 42B         0.14273

 Excited State  23:  3.246-A 6.1071 eV  203.02 nm  f=0.0066  <S**2>=2.383
     38A -> 43A        -0.17584
     41A -> 48A        -0.12850
     42A -> 44A         0.10917
     42A -> 45A        -0.15561
     26B -> 42B         0.10374
     27B -> 42B         0.19111
     28B -> 42B         0.20058
     31B -> 41B        -0.42327
     31B -> 43B         0.11872
     32B -> 42B        -0.11727
     33B -> 41B         0.31241
     34B -> 42B         0.43541
     37B -> 42B        -0.15954
     38B -> 42B         0.27549
     38B -> 43B         0.14476
     40B -> 51B        -0.11385
     40B -> 52B        -0.13848

 Excited State  24:  3.481-A  6.1160 eV  202.72 nm  f=0.0015  <S**2>=2.779
     38A -> 43A        -0.29416
     41A -> 48A        -0.21097
     42A -> 44A        -0.33407
     42A -> 45A         0.43910
     42A -> 46A         0.18784
     42A -> 47A        -0.17730
     42A -> 48A         0.11105
     42A -> 52A        -0.20615
     42A -> 53A         0.14439
     42A -> 54A        -0.25349
     42A -> 55A        -0.14888
     34B -> 41B        -0.20806
     38B -> 43B         0.26188
     40B -> 52B        -0.23545
```

```
 Excited State  25:  3.467-A   6.1266 eV  202.37 nm  f=0.0025  <S**2>=2.755
      38A -> 43A         0.30229
      41A -> 48A         0.22598
      41A -> 50A        -0.10344
      42A -> 44A        -0.24359
      42A -> 45A         0.30801
      42A -> 46A         0.11773
      42A -> 47A        -0.13366
      42A -> 48A         0.18454
      42A -> 52A        -0.16525
      42A -> 53A         0.10850
      42A -> 54A        -0.19428
      42A -> 55A        -0.12251
      27B -> 42B         0.13771
      28B -> 42B         0.11003
      31B -> 41B        -0.14409
      31B -> 42B         0.10542
      32B -> 42B        -0.10028
      33B -> 41B         0.11875
      34B -> 41B         0.23593
      34B -> 42B         0.19586
      35B -> 42B         0.14476
      38B -> 42B         0.16257
      38B -> 43B        -0.27132
      40B -> 43B        -0.13035
      40B -> 52B         0.24613
***************************************************************************
```

13. TD-DFT calculation of nitrosoalkene ^{1}3A in gas phase

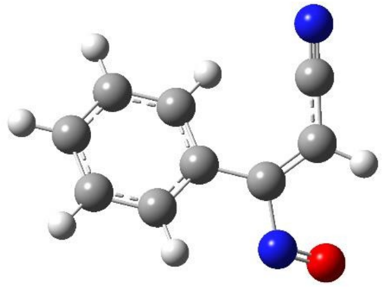

```
Excitation energies and oscillator strengths:

 Excited State   1: Singlet-A   0.8856 eV 1399.97 nm  f=0.0001  <S**2>=0.000
      39 -> 42         0.62928
      39 -> 43         0.25837
      40 -> 42         0.24010
      39 <- 42        -0.21755
      39 <- 43        -0.10942
 This state for optimization and/or second-order correction.
 Total Energy, E(TD-HF/TD-DFT) =  -530.940009141
 Copying the excited state density for this state as the 1-particle RhoCI
density.

 Excited State   2:      Singlet-A      3.4840 eV  355.87 nm  f=0.1323
<S**2>=0.000
      41 -> 42         0.69747
```

```
 Excited State   3: Singlet-A 4.0175 eV  308.61 nm  f=0.0072  <S**2>=0.000
      39 -> 42        -0.23865
      40 -> 42         0.65332

 Excited State   4: Singlet-A 4.8460 eV  255.85 nm  f=0.0273  <S**2>=0.000
      37 -> 42        -0.32318
      38 -> 42         0.23658
      39 -> 42        -0.24689
      39 -> 43         0.39844
      39 -> 48        -0.11552
      40 -> 43         0.14098

 Excited State   5: Singlet-A   5.0931 eV  243.44 nm  f=0.0703  <S**2>=0.000
      37 -> 42        -0.11528
      38 -> 42         0.49389
      39 -> 43        -0.31487
      39 -> 48         0.10101
      40 -> 43        -0.19929
      41 -> 43        -0.20424

 Excited State   6:      Singlet-A  5.2176 eV  237.63 nm  f=0.0752
<S**2>=0.000
      35 -> 42        -0.10620
      36 -> 42         0.11907
      37 -> 42         0.50943
      37 -> 43        -0.11549
      38 -> 42         0.22117
      39 -> 42        -0.13109
      39 -> 43         0.18745
      41 -> 43        -0.18468

 Excited State   7: Singlet-A 5.4096 eV  229.19 nm  f=0.0923  <S**2>=0.000
      38 -> 42        -0.10405
      39 -> 43        -0.16345
      40 -> 42         0.11264
      40 -> 43         0.37937
      40 -> 48         0.10670
      41 -> 43        -0.37222
      41 -> 44         0.34049

 Excited State   8: Singlet-A  5.4910 eV  225.80 nm  f=0.1529  <S**2>=0.000
      38 -> 42         0.28466
      40 -> 43         0.23023
      41 -> 43         0.48923
      41 -> 44         0.27885

 Excited State   9: Singlet-A 6.2550 eV  198.21 nm  f=0.0118  <S**2>=0.000
      26 -> 42         0.12725
      28 -> 42         0.18025
      31 -> 42         0.27566
      35 -> 42         0.22971
      36 -> 42         0.19281
      40 -> 43        -0.15650
      40 -> 44         0.13216
      41 -> 44         0.11636
      41 -> 45        -0.14692
```

```
     41 -> 46        -0.13790
     41 -> 49         0.12022
     41 -> 53        -0.12608

Excited State  10: Singlet-A  6.3927 eV  193.95 nm  f=0.0377  <S**2>=0.000
     31 -> 42         0.11535
     36 -> 42         0.50396
     37 -> 42        -0.18015
     39 -> 43        -0.10296
     40 -> 43         0.18156
     41 -> 44        -0.19767
     41 -> 45         0.11706
     41 -> 46         0.10588

Excited State  11: Singlet-A 6.4666 eV  191.73 nm  f=0.1621  <S**2>=0.000
     35 -> 42        -0.15616
     36 -> 42         0.14436
     39 -> 43         0.16252
     40 -> 43        -0.34796
     41 -> 44         0.41602
     41 -> 45         0.14821
     41 -> 48        -0.10109

Excited State  12:   Singlet-A  6.5675 eV  188.79 nm  f=0.0538  <S**2>=0.000
     28 -> 42         0.10779
     31 -> 42         0.15340
     35 -> 42         0.24972
     36 -> 42        -0.16354
     40 -> 44        -0.29052
     41 -> 44         0.11050
     41 -> 45         0.38766
     41 -> 46         0.14465
     41 -> 48         0.11575

Excited State  13:  Singlet-A 6.6053 eV  187.70 nm  f=0.1273  <S**2>=0.000
     28 -> 42         0.10375
     36 -> 42        -0.25311
     38 -> 43        -0.11482
     39 -> 44        -0.14144
     40 -> 44         0.35703
     41 -> 44        -0.11760
     41 -> 46         0.10535
     41 -> 48        -0.27934
     41 -> 52         0.11342
     41 -> 53         0.14403

Excited State  14: Singlet-A  6.7252 eV  184.36 nm  f=0.0292  <S**2>=0.000
     26 -> 42        -0.17798
     28 -> 42        -0.15023
     29 -> 42        -0.10060
     32 -> 42         0.12377
     33 -> 42        -0.22339
     34 -> 42         0.12711
     35 -> 42         0.41064
     35 -> 43        -0.10290
     38 -> 43         0.12273
     40 -> 44         0.18555
```

```
     41 -> 48        -0.10562

 Excited State  15: Singlet-A 6.8041 eV  182.22 nm  f=0.0900  <S**2>=0.000
     35 -> 42        -0.11561
     40 -> 43         0.12639
     40 -> 44         0.23498
     41 -> 45         0.47117
     41 -> 46        -0.29731
     41 -> 49         0.13688
     41 -> 53        -0.13808

 Excited State  16: Singlet-A 6.9561 eV  178.24 nm  f=0.0302  <S**2>=0.000
     33 -> 42         0.25718
     39 -> 44         0.41201
     39 -> 45         0.16155
     39 -> 46         0.14381
     40 -> 44         0.26946
     40 -> 45         0.14515
     41 -> 46         0.16697
     41 -> 48         0.15184

 Excited State  17: Singlet-A  6.9643 eV  178.03 nm   f=0.0100   <S**2>=0.000
     33 -> 42         0.51511
     33 -> 43        -0.11341
     35 -> 42         0.15572
     39 -> 44        -0.29010

 Excited State  18: Singlet-A 7.0217 eV  176.57 nm  f=0.0323  <S**2>=0.000
     33 -> 42         0.18296
     38 -> 43         0.16065
     39 -> 43        -0.11035
     39 -> 44         0.32031
     39 -> 45        -0.13754
     39 -> 46        -0.11960
     40 -> 45        -0.29794
     41 -> 46        -0.17464
     41 -> 48        -0.24222

 Excited State  19: Singlet-A  7.0818 eV  175.07 nm   f=0.0390   <S**2>=0.000
     38 -> 43         0.14473
     39 -> 45        -0.23076
     40 -> 45         0.47085
     40 -> 46        -0.29250
     41 -> 46        -0.18520

 Excited State  20: Singlet-A 7.1062 eV  174.47 nm  f=0.1008  <S**2>=0.000
     39 -> 43         0.10261
     39 -> 45         0.36338
     39 -> 46         0.19302
     39 -> 50        -0.10505
     40 -> 44        -0.17577
     40 -> 45         0.12729
     40 -> 46         0.17227
     41 -> 46        -0.34371
     41 -> 48        -0.16367

 Excited State  21: Singlet-A 7.1770 eV  172.75 nm  f=0.0717  <S**2>=0.000
```

```
      26 -> 42         0.11867
      31 -> 42         0.10740
      32 -> 42         0.13400
      34 -> 42         0.38192
      37 -> 43         0.10237
      40 -> 44         0.12266
      41 -> 46        -0.15969
      41 -> 47        -0.10986
      41 -> 48         0.24374
      41 -> 49        -0.25336

 Excited State  22: Singlet-A 7.2146 eV  171.85 nm  f=0.0176  <S**2>=0.000
      31 -> 42         0.10116
      32 -> 42         0.11283
      34 -> 42         0.25083
      35 -> 42        -0.11365
      38 -> 43         0.22985
      39 -> 45         0.19848
      41 -> 46         0.22704
      41 -> 47         0.22700
      41 -> 49         0.27643

 Excited State  23:      Singlet-A      7.3431 eV  168.84 nm  f=0.0726
<S**2>=0.000
      34 -> 42         0.21402
      36 -> 43        -0.11649
      38 -> 43        -0.16507
      39 -> 43         0.12424
      39 -> 44         0.20962
      39 -> 45        -0.13879
      39 -> 48         0.32463
      39 -> 49        -0.24558
      39 -> 50         0.12132
      40 -> 43         0.10058
      40 -> 46        -0.11367
      41 -> 49         0.12750

 Excited State  24: Singlet-A 7.3977 eV  167.60 nm  f=0.0064  <S**2>=0.000
      41 -> 47         0.60049
      41 -> 49        -0.27387

 Excited State  25: Singlet-A  7.4351 eV  166.76 nm  f=0.1020  <S**2>=0.000
      39 -> 45        -0.11735
      39 -> 46        -0.17450
      40 -> 45         0.12259
      40 -> 46         0.31055
      40 -> 47        -0.15253
      40 -> 48         0.43217
      40 -> 49        -0.14454
      41 -> 44        -0.14019
      41 -> 45         0.10518
**********************************************************************
```

14. TD-DFT calculation of nitrosoalkene ^{1}3A in IEFPCM

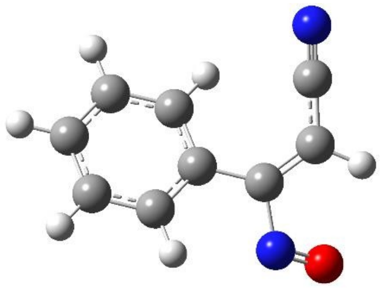

```
Excited State   1:  Singlet-A 0.9205 eV 1346.99 nm  f=0.0002  <S**2>=0.000
      38 -> 42         -0.10528
      39 -> 42          0.65430
      39 -> 43          0.26587
      40 -> 42         -0.15076
      39 <- 42         -0.21765
      39 <- 43         -0.10901
 This state for optimization and/or second-order correction.
 Total Energy, E(TD-HF/TD-DFT) =  -530.945317147
 Copying the excited state density for this state as the 1-particle RhoCI
density.

 Excited State   2: Singlet-A 3.3611 eV  368.89 nm  f=0.1648  <S**2>=0.000
      41 -> 42          0.69836

 Excited State   3: Singlet-A 3.9307 eV  315.43 nm  f=0.0091  <S**2>=0.000
      39 -> 42          0.14751
      40 -> 42          0.68204

 Excited State   4: Singlet-A 4.8763 eV  254.26 nm  f=0.0853  <S**2>=0.000
      37 -> 42         -0.26624
      38 -> 42          0.39056
      39 -> 42          0.23983
      39 -> 43         -0.33149
      41 -> 43         -0.14362

 Excited State   5: Singlet-A 5.0766 eV  244.23 nm  f=0.0997  <S**2>=0.000
      37 -> 42          0.10968
      38 -> 42          0.43650
      39 -> 42         -0.11108
      39 -> 43          0.38893
      39 -> 48         -0.10638
      40 -> 43         -0.15629
      41 -> 43         -0.23808

 Excited State   6: Singlet-A5.2505 eV  236.14 nm  f=0.0569  <S**2>=0.000
      35 -> 42         -0.14214
      36 -> 42          0.15826
      37 -> 42          0.51044
      37 -> 43         -0.11599
      39 -> 42          0.13345
      39 -> 43         -0.25269
      41 -> 43         -0.16982

 Excited State   7: Singlet-A 5.3672 eV  231.01 nm  f=0.2042  <S**2>=0.000
      38 -> 42          0.21848
      40 -> 43         -0.28742
```

```
      41 -> 43         0.50595
      41 -> 44        -0.22513

 Excited State   8: Singlet-A 5.4433 eV  227.77 nm  f=0.0941  <S**2>=0.000
      38 -> 42         0.22498
      39 -> 43         0.11366
      40 -> 43         0.37798
      41 -> 43         0.32728
      41 -> 44         0.36617

 Excited State   9:      Singlet-A      6.2139 eV  199.53 nm  f=0.0351
<S**2>=0.000
      26 -> 42         0.11255
      28 -> 42         0.16904
      31 -> 42         0.23675
      35 -> 42         0.20350
      36 -> 42         0.23905
      40 -> 43        -0.20716
      40 -> 44         0.10626
      41 -> 44         0.19110
      41 -> 45        -0.15816
      41 -> 46         0.15749
      41 -> 49         0.12060
      41 -> 52        -0.12883

 Excited State  10:      Singlet-A      6.2926 eV  197.03 nm  f=0.0450
<S**2>=0.000
      31 -> 42         0.10388
      36 -> 42         0.52047
      37 -> 42        -0.21916
      40 -> 43         0.18438
      41 -> 44        -0.21382

 Excited State  11:      Singlet-A      6.3904 eV  194.02 nm  f=0.2264
<S**2>=0.000
      35 -> 42        -0.21211
      36 -> 42         0.14281
      37 -> 42        -0.13496
      39 -> 43        -0.10248
      40 -> 43        -0.34612
      41 -> 44         0.39459
      41 -> 46        -0.10441
      41 -> 48        -0.11609

 Excited State  12:      Singlet-A      6.5373 eV  189.66 nm  f=0.2127
<S**2>=0.000
      38 -> 43        -0.11224
      40 -> 44         0.53175
      41 -> 44        -0.15436
      41 -> 45        -0.19985
      41 -> 47         0.12454
      41 -> 48        -0.25381

 Excited State  13:      Singlet-A      6.5469 eV  189.38 nm  f=0.0123
<S**2>=0.000
      26 -> 42         0.11473
      28 -> 42         0.15265
```

```
      31 -> 42         0.18612
      35 -> 42         0.31078
      36 -> 42        -0.16921
      41 -> 45         0.31073
      41 -> 46        -0.18800
      41 -> 48        -0.15978
      41 -> 52         0.15961

 Excited State  14:      Singlet-A      6.7211 eV  184.47 nm  f=0.0447
<S**2>=0.000
      26 -> 42        -0.21954
      27 -> 42        -0.10616
      28 -> 42        -0.18283
      29 -> 42        -0.11042
      31 -> 42        -0.11928
      32 -> 42        -0.13739
      33 -> 42        -0.14340
      34 -> 42         0.11341
      35 -> 42         0.36416
      35 -> 43        -0.10270
      36 -> 42         0.15679
      38 -> 43         0.14799
      40 -> 44         0.16071

 Excited State  15:      Singlet-A      6.7730 eV  183.06 nm  f=0.1152
<S**2>=0.000
      35 -> 42        -0.12733
      40 -> 43         0.10439
      40 -> 44         0.22747
      41 -> 45         0.49634
      41 -> 46         0.27838
      41 -> 49         0.10736
      41 -> 53        -0.13630

 Excited State  16:      Singlet-A      6.9946 eV  177.26 nm  f=0.1757
<S**2>=0.000
      38 -> 43        -0.11239
      39 -> 45        -0.12614
      40 -> 44         0.25643
      40 -> 45         0.14719
      41 -> 46        -0.28360
      41 -> 47        -0.17484
      41 -> 48         0.39587
      41 -> 49        -0.11702
      41 -> 50        -0.15738

 Excited State  17:      Singlet-A      7.0794 eV  175.13 nm  f=0.0078
<S**2>=0.000
      33 -> 42         0.10831
      36 -> 44         0.13087
      39 -> 44         0.58086
      39 -> 48        -0.10782
      39 -> 49         0.11166

 Excited State  18:      Singlet-A      7.1017 eV  174.58 nm  f=0.0804
<S**2>=0.000
      40 -> 45         0.55319
```

```
     40 -> 46         0.26323
     40 -> 47        -0.15128
     41 -> 46         0.15379
     41 -> 48        -0.11625

 Excited State  19: Singlet-A 7.1446 eV  173.53 nm  f=0.0119  <S**2>=0.000
     32 -> 42        -0.19585
     33 -> 42         0.54472
     33 -> 43        -0.12004
     34 -> 42         0.18173
     38 -> 43         0.17860
     39 -> 44        -0.10223

 Excited State  20: Singlet-A 7.1512 eV  173.38 nm  f=0.0696  <S**2>=0.000
     31 -> 42         0.10467
     33 -> 42        -0.12688
     34 -> 42         0.16933
     36 -> 43         0.13349
     38 -> 43         0.14895
     39 -> 43         0.12226
     39 -> 45         0.38851
     39 -> 46        -0.14284
     39 -> 47        -0.18653
     39 -> 48         0.13295
     40 -> 44         0.12729
     40 -> 46         0.14941
     41 -> 48         0.11657

 Excited State  21: Singlet-A 7.1892 eV  172.46 nm  f=0.0095  <S**2>=0.000
     31 -> 42         0.13228
     32 -> 42        -0.12471
     33 -> 42        -0.20330
     34 -> 42         0.38760
     37 -> 43         0.11685
     39 -> 45        -0.27255
     39 -> 47         0.10743
     40 -> 46        -0.13251
     41 -> 46         0.12813

 Excited State  22: Singlet-A 7.2620 eV  170.73 nm  f=0.0121  <S**2>=0.000
     38 -> 43         0.16161
     39 -> 44         0.11837
     39 -> 45        -0.11517
     41 -> 46        -0.36263
     41 -> 47         0.38465
     41 -> 49         0.27503

 Excited State  23: Singlet-A 7.3532 eV  168.61 nm  f=0.0664  <S**2>=0.000
     34 -> 42         0.29125
     38 -> 43        -0.28052
     39 -> 44        -0.22297
     39 -> 45         0.19462
     39 -> 48        -0.18306
     39 -> 49         0.16341
     39 -> 50         0.10431
     40 -> 48         0.10614
     41 -> 49         0.15051
```

```
      41 -> 50         0.10336

 Excited State  24:  Singlet-A  7.3696 eV  168.24 nm  f=0.1189  <S**2>=0.000
      39 -> 46        -0.11651
      39 -> 48         0.11975
      40 -> 46        -0.34371
      40 -> 47        -0.24934
      40 -> 48         0.39830
      40 -> 49        -0.11209
      41 -> 44        -0.11589

 Excited State  25: Singlet-A 7.4415 eV  166.61 nm  f=0.0105  <S**2>=0.000
      41 -> 46         0.12722
      41 -> 47         0.46316
      41 -> 48         0.22904
      41 -> 49        -0.37586
      41 -> 53         0.12000

*****************************************************************************
```

15. TD-DFT calculation of nitrosoalkene ^{1}3A in SMD

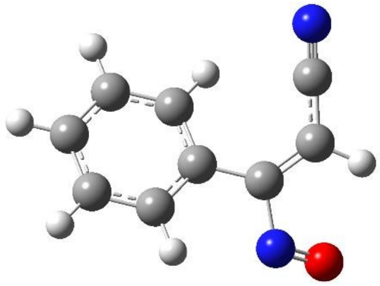

```
Excitation energies and oscillator strengths:

 Excited State   1:      Singlet-A      0.9209 eV 1346.26 nm  f=0.0002
<S**2>=0.000
      38 -> 42        -0.10742
      39 -> 42         0.65715
      39 -> 43         0.26433
      40 -> 42        -0.14082
      39 <- 42        -0.21858
      39 <- 43        -0.10827
 This state for optimization and/or second-order correction.
 Total Energy, E(TD-HF/TD-DFT) =  -530.951049571
 Copying the excited state density for this state as the 1-particle RhoCI
density.

 Excited State   2: Singlet-A   3.3291 eV  372.43 nm  f=0.1677  <S**2>=0.000
      41 -> 42         0.69850

 Excited State   3: Singlet-A  3.9164 eV  316.57 nm  f=0.0096  <S**2>=0.000
      39 -> 42         0.13719
      40 -> 42         0.68450

 Excited State   4: Singlet-A  4.8802 eV  254.06 nm  f=0.0978  <S**2>=0.000
      37 -> 42        -0.25229
      38 -> 42         0.42594
```

```
      39 -> 42         0.23028
      39 -> 43        -0.29984
      41 -> 43        -0.16206

 Excited State   5: Singlet-A  5.0799 eV  244.07 nm  f=0.0942  <S**2>=0.000
      37 -> 42         0.14614
      38 -> 42         0.39642
      39 -> 42        -0.12383
      39 -> 43         0.40811
      39 -> 48        -0.10910
      40 -> 43        -0.15312
      41 -> 43        -0.24693

 Excited State   6:   Singlet-A   5.2563  eV  235.88 nm  f=0.0606 <S**2>=0.000
      35 -> 42         0.15147
      36 -> 42         0.17099
      37 -> 42         0.48947
      37 -> 43        -0.11105
      39 -> 42         0.13593
      39 -> 43        -0.26584
      41 -> 43        -0.19479

 Excited State   7: Singlet-A 5.3460 eV  231.92 nm  f=0.2202  <S**2>=0.000
      37 -> 42         0.13203
      38 -> 42         0.24416
      40 -> 43        -0.25587
      41 -> 43         0.51572
      41 -> 44        -0.19219

 Excited State   8:  Singlet-A   5.4307 eV  228.30 nm  f=0.0784  <S**2>=0.000
      38 -> 42         0.21220
      39 -> 43         0.12118
      40 -> 43         0.40150
      41 -> 43         0.28258
      41 -> 44         0.37014
      41 -> 45        -0.11362

 Excited State   9: Singlet-A   6.1921 eV  200.23 nm  f=0.0432  <S**2>=0.000
      26 -> 42         0.10865
      28 -> 42         0.16836
      31 -> 42         0.22816
      35 -> 42        -0.18281
      36 -> 42         0.30745
      40 -> 43        -0.21646
      40 -> 44         0.10367
      41 -> 44         0.19207
      41 -> 45        -0.16503
      41 -> 46         0.13660
      41 -> 49         0.11358
      41 -> 52        -0.11765

 Excited State  10:      Singlet-A      6.2429 eV  198.60 nm  f=0.0482
<S**2>=0.000
      36 -> 42         0.49863
      37 -> 42        -0.24874
      40 -> 43         0.19577
      41 -> 44        -0.21816
```

```
      41 -> 45         0.11404

 Excited State  11:   Singlet-A  6.3533 eV  195.15 nm  f=0.2330  <S**2>=0.000
      31 -> 42        -0.10756
      35 -> 42         0.22755
      36 -> 42         0.10416
      37 -> 42        -0.14222
      40 -> 43        -0.33300
      41 -> 44         0.40070
      41 -> 46        -0.11571
      41 -> 48        -0.10339
      41 -> 52         0.10772

 Excited State  12: Singlet-A    6.5020 eV  190.69 nm  f=0.1951  <S**2>=0.000
      35 -> 42         0.12274
      38 -> 43        -0.10251
      40 -> 44         0.50834
      40 -> 45        -0.10507
      41 -> 44        -0.16996
      41 -> 45        -0.25065
      41 -> 47         0.11792
      41 -> 48        -0.22206

 Excited State  13:      Singlet-A      6.5230 eV  190.07 nm  f=0.0384
<S**2>=0.000
      26 -> 42        -0.10639
      28 -> 42        -0.14442
      31 -> 42        -0.17346
      35 -> 42         0.30486
      36 -> 42         0.13687
      40 -> 44        -0.15853
      41 -> 45        -0.29879
      41 -> 46         0.17094
      41 -> 48         0.19692
      41 -> 50        -0.10668
      41 -> 52        -0.16350

 Excited State  14: Singlet-A  6.7060 eV  184.88 nm  f=0.0726  <S**2>=0.000
      26 -> 42         0.22510
      27 -> 42         0.12177
      28 -> 42         0.18753
      29 -> 42         0.10980
      31 -> 42         0.14564
      33 -> 42         0.14617
      34 -> 42        -0.10101
      35 -> 42         0.29413
      36 -> 42        -0.16498
      38 -> 43        -0.14249
      40 -> 44        -0.20791
      41 -> 45        -0.15057

 Excited State  15: Singlet-A 6.7254 eV  184.35 nm  f=0.0991  <S**2>=0.000
      35 -> 42         0.21888
      40 -> 43         0.11413
      40 -> 44         0.18171
      41 -> 45         0.43906
      41 -> 46         0.29327
```

```
     41 -> 49         0.11023
     41 -> 53        -0.14438

Excited State  16: Singlet-A 6.9627 eV  178.07 nm  f=0.1698  <S**2>=0.000
     39 -> 45        -0.12973
     40 -> 44         0.26545
     40 -> 45         0.14745
     41 -> 46        -0.25657
     41 -> 47        -0.14146
     41 -> 48         0.40055
     41 -> 49        -0.15752
     41 -> 50        -0.17305

Excited State  17: Singlet-A 7.0565 eV  175.70 nm  f=0.1024  <S**2>=0.000
     39 -> 44        -0.15470
     40 -> 45         0.50298
     40 -> 46         0.23626
     40 -> 47        -0.14144
     41 -> 46         0.15269
     41 -> 48        -0.16991

Excited State  18: Singlet-A  7.0686 eV  175.40 nm  f=0.0060  <S**2>=0.000
     36 -> 44         0.10977
     39 -> 44         0.53139
     39 -> 45         0.16360
     39 -> 47        -0.11417
     39 -> 49         0.11716
     40 -> 45         0.19742
     40 -> 46         0.12566

Excited State  19: Singlet-A 7.1252 eV  174.01 nm  f=0.0583  <S**2>=0.000
     36 -> 43         0.15918
     38 -> 43         0.17973
     39 -> 43         0.14447
     39 -> 44        -0.12746
     39 -> 45         0.40392
     39 -> 46        -0.11361
     39 -> 47        -0.18698
     39 -> 48         0.16299
     40 -> 44         0.13938
     40 -> 46         0.16157

Excited State  20: Singlet-A  7.1667 eV  173.00 nm  f=0.0045  <S**2>=0.000
     26 -> 42         0.11163
     31 -> 42         0.11149
     32 -> 42        -0.26733
     34 -> 42         0.42363
     38 -> 43         0.17455
     39 -> 45        -0.14630
     41 -> 46         0.15939

Excited State  21: Singlet-A 7.1937 eV  172.35 nm  f=0.0236  <S**2>=0.000
     32 -> 42        -0.21663
     33 -> 42         0.52022
     33 -> 43        -0.11998
     37 -> 43        -0.10769
     41 -> 47         0.16642
```

```
 Excited State   22: Singlet-A  7.2231 eV  171.65 nm  f=0.0110  <S**2>=0.000
      33 -> 42        -0.12248
      38 -> 43         0.13161
      39 -> 44         0.12814
      39 -> 45        -0.11407
      41 -> 45         0.10398
      41 -> 46        -0.35024
      41 -> 47         0.37812
      41 -> 49         0.25977

 Excited State   23: Singlet-A 7.3237 eV  169.29 nm  f=0.0800  <S**2>=0.000
      34 -> 42         0.27716
      37 -> 43         0.11074
      38 -> 43        -0.27250
      39 -> 44        -0.24557
      39 -> 45         0.21419
      39 -> 48        -0.12198
      39 -> 49         0.14336
      40 -> 48         0.19388
      40 -> 49        -0.10507
      41 -> 49         0.12344

 Excited State   24: Singlet-A 7.3403 eV  168.91 nm  f=0.0903  <S**2>=0.000
      34 -> 42        -0.14057
      38 -> 43         0.13073
      39 -> 48         0.14206
      40 -> 43        -0.10492
      40 -> 46        -0.31646
      40 -> 47        -0.19374
      40 -> 48         0.38216
      40 -> 49        -0.13899
      41 -> 49        -0.10551

 Excited State   25: Singlet-A   7.3840 eV  167.91 nm  f=0.0179  <S**2>=0.000
      38 -> 43        -0.11075
      41 -> 46         0.15130
      41 -> 47         0.46609
      41 -> 48         0.15524
      41 -> 49        -0.37589
      41 -> 53         0.11943

 **********************************************************************
```

16. TD-DFT calculation of nitrosoalkene $^{1}3A$ in I-PCM

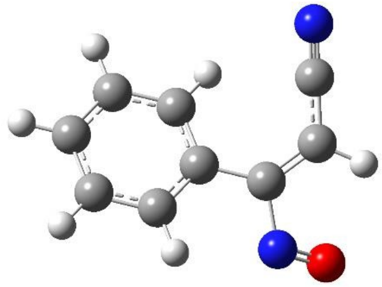

```
Excitation energies and oscillator strengths:
```

```
 Excited State   1: Singlet-A 0.8358 eV 1483.47 nm  f=0.0001  <S**2>=0.000
      39 -> 42        -0.64998
      39 -> 43        -0.27677
      40 -> 42         0.18891
      39 <- 42         0.23519
      39 <- 43         0.12203
 This state for optimization and/or second-order correction.
 Total Energy, E(TD-HF/TD-DFT) =  -530.949099630
 Copying the excited state density for this state as the 1-particle RhoCI
density.

 Excited State   2: Singlet-A 3.4217 eV  362.35 nm  f=0.1371  <S**2>=0.000
      41 -> 42         0.69742

 Excited State   3: Singlet-A  3.8964 eV  318.20 nm   f=0.0071   <S**2>=0.000
      39 -> 42         0.18298
      40 -> 42         0.67333

 Excited State   4: Singlet-A 4.8527 eV  255.49 nm  f=0.0348  <S**2>=0.000
      37 -> 42        -0.17435
      38 -> 42         0.23354
      39 -> 42         0.28244
      39 -> 43        -0.47193
      39 -> 48         0.12579
      40 -> 43         0.13045
      41 -> 43        -0.10523

 Excited State   5: Singlet-A  5.0781 eV  244.15 nm   f=0.0833   <S**2>=0.000
      38 -> 42         0.52428
      39 -> 43         0.28605
      40 -> 43        -0.15838
      41 -> 43        -0.22414

 Excited State   6: Singlet-A 5.3523 eV  231.65 nm  f=0.0661  <S**2>=0.000
      35 -> 42         0.19790
      36 -> 42         0.16392
      37 -> 42         0.49329
      37 -> 43        -0.10420
      39 -> 43        -0.11024
      41 -> 43        -0.28236
      41 -> 44        -0.11069

 Excited State   7:   Singlet-A  5.4275 eV  228.44 nm  f=0.0696  <S**2>=0.000
      39 -> 43         0.14976
      40 -> 42         0.10305
      40 -> 43         0.41899
      40 -> 48         0.13009
      41 -> 43        -0.31533
      41 -> 44         0.35672

 Excited State   8: Singlet-A  5.4954 eV  225.61 nm  f=0.1517  <S**2>=0.000
      37 -> 42         0.20797
      38 -> 42         0.31340
      40 -> 43         0.20385
      41 -> 43         0.47047
      41 -> 44         0.22172
```

```
Excited State   9: Singlet-A  6.1251 eV  202.42 nm  f=0.0216  <S**2>=0.000
     28 -> 42         0.13125
     31 -> 42         0.18683
     35 -> 42        -0.12598
     36 -> 42         0.16910
     37 -> 42         0.13348
     38 -> 46        -0.12853
     40 -> 43        -0.14053
     41 -> 44         0.13916
     41 -> 45        -0.24369
     41 -> 46         0.28627
     41 -> 49         0.14384
     41 -> 50        -0.12952

Excited State  10: Singlet-A  6.2277 eV  199.08 nm  f=0.0031  <S**2>=0.000
     31 -> 42         0.14480
     36 -> 42         0.54631
     37 -> 42        -0.25479
     41 -> 46        -0.10614

Excited State  11: Singlet-A 6.4444 eV  192.39 nm  f=0.1310  <S**2>=0.000
     39 -> 43        -0.15089
     40 -> 43        -0.39116
     40 -> 44         0.12857
     41 -> 44         0.41749
     41 -> 45         0.12469
     41 -> 46        -0.14760
     41 -> 48        -0.15169

Excited State  12: Singlet-A 6.5034 eV  190.65 nm  f=0.0557  <S**2>=0.000
     26 -> 42        -0.13153
     28 -> 42         0.18985
     31 -> 42         0.24701
     35 -> 42        -0.36699
     36 -> 42        -0.17649
     37 -> 42         0.15631
     40 -> 43         0.11458
     41 -> 44        -0.13127
     41 -> 45         0.18157
     41 -> 46        -0.13776

Excited State  13: Singlet-A  6.5735 eV  188.61 nm  f=0.1044  <S**2>=0.000
     39 -> 44        -0.10727
     40 -> 44        -0.44062
     41 -> 44         0.17838
     41 -> 45         0.31437
     41 -> 47         0.15077
     41 -> 48         0.27577

Excited State  14: Singlet-A  6.7144 eV  184.66 nm  f=0.0286  <S**2>=0.000
     26 -> 42         0.22092
     28 -> 42        -0.18405
     29 -> 42        -0.12176
     31 -> 42        -0.14394
     33 -> 42         0.12755
     35 -> 42        -0.35911
     36 -> 42         0.18605
```

```
     37 -> 42         0.12077
     38 -> 43         0.11045
     40 -> 44         0.18764
     41 -> 45         0.10608

Excited State  15: Singlet-A 6.7508 eV  183.66 nm  f=0.1039  <S**2>=0.000
     35 -> 42         0.16247
     40 -> 43         0.13400
     40 -> 44         0.24625
     41 -> 45         0.42747
     41 -> 46         0.35152
     41 -> 49         0.11536

Excited State  16: Singlet-A 7.0039 eV  177.02 nm  f=0.0717  <S**2>=0.000
     39 -> 44        -0.22591
     39 -> 45        -0.28267
     39 -> 46         0.17105
     39 -> 47        -0.11778
     39 -> 49        -0.12144
     40 -> 44         0.26316
     40 -> 45         0.24523
     40 -> 47         0.12972
     41 -> 48         0.27635

Excited State  17: Singlet-A 7.0521 eV  175.81 nm  f=0.0240  <S**2>=0.000
     36 -> 44        -0.12094
     38 -> 43         0.11859
     39 -> 43         0.11196
     39 -> 44        -0.55332
     39 -> 48         0.11814
     40 -> 45        -0.13357
     40 -> 47        -0.11024
     41 -> 48        -0.16564

Excited State  18: Singlet-A 7.0917 eV  174.83 nm  f=0.0290  <S**2>=0.000
     38 -> 43         0.13314
     39 -> 45         0.25808
     39 -> 47         0.11730
     40 -> 45         0.37520
     40 -> 46         0.35670
     40 -> 47         0.19551
     41 -> 48        -0.10565

Excited State  19: Singlet-A7.1067 eV  174.46 nm  f=0.2020  <S**2>=0.000
     39 -> 45        -0.33788
     39 -> 46         0.11309
     39 -> 47        -0.11155
     39 -> 49        -0.10561
     40 -> 44        -0.24083
     40 -> 45         0.20463
     41 -> 46         0.23121
     41 -> 48        -0.32027

Excited State  20: Singlet-A  7.2187 eV  171.75 nm  f=0.0334  <S**2>=0.000
     33 -> 42         0.18246
     34 -> 42         0.38047
     38 -> 43         0.12092
```

```
     39 -> 48         0.11306
     41 -> 46         0.15977
     41 -> 47         0.28876
     41 -> 49        -0.17331

Excited State   21: Singlet-A  7.2396 eV  171.26 nm  f=0.0087  <S**2>=0.000
     33 -> 42         0.11526
     34 -> 42         0.18020
     38 -> 43         0.22340
     39 -> 44         0.12277
     39 -> 45        -0.11088
     39 -> 48         0.11137
     41 -> 45         0.11398
     41 -> 46        -0.13658
     41 -> 47        -0.44393
     41 -> 48         0.11450
     41 -> 49         0.13682

Excited State   22: Singlet-A 7.3140 eV  169.52 nm  f=0.0342  <S**2>=0.000
     32 -> 42         0.47183
     34 -> 42         0.16934
     36 -> 43        -0.11794
     39 -> 44        -0.14308
     39 -> 48        -0.18208
     39 -> 49         0.12978
     41 -> 47        -0.18425

Excited State   23: Singlet-A  7.3442 eV  168.82 nm  f=0.0944  <S**2>=0.000
     32 -> 42        -0.13824
     34 -> 42         0.10637
     39 -> 44        -0.14003
     39 -> 45         0.14847
     39 -> 46        -0.13745
     40 -> 45         0.14281
     40 -> 46        -0.31147
     40 -> 47         0.20837
     40 -> 48         0.38007
     40 -> 49        -0.11570
     41 -> 44        -0.15430
     41 -> 45         0.10814

Excited State   24:   Singlet-A  7.3693 eV  168.24 nm  f=0.0400  <S**2>=0.000
     32 -> 42        -0.29636
     34 -> 42         0.25976
     37 -> 43         0.15081
     38 -> 43        -0.15369
     39 -> 44        -0.10050
     39 -> 48        -0.26832
     39 -> 49         0.13366
     40 -> 46         0.20065
     41 -> 46        -0.10032
     41 -> 49         0.15301

Excited State   25: Singlet-A 7.4277 eV  166.92 nm  f=0.0079  <S**2>=0.000
     32 -> 42        -0.17470
     38 -> 43        -0.18317
     39 -> 46         0.11965
```

```
      40 -> 48          -0.11576
      41 -> 46           0.23475
      41 -> 47          -0.30533
      41 -> 48           0.15407
      41 -> 49          -0.34950
      41 -> 50           0.10297
      41 -> 53           0.13172
```

 ****

17. TD-DFT calculation of nitrosoalkene [1]3A in C-PCM

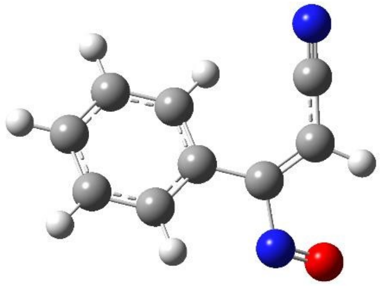

```
Excitation energies and oscillator strengths:

 Excited State   1: Singlet-A  0.9236 eV 1342.45 nm  f=0.0002  <S**2>=0.000
      38 -> 42         -0.10516
      39 -> 42          0.65317
      39 -> 43          0.26542
      40 -> 42         -0.15306
      39 <- 42         -0.21582
      39 <- 43         -0.10807
 This state for optimization and/or second-order correction.
 Total Energy, E(TD-HF/TD-DFT) =  -530.945678683
 Copying the excited state density for this state as the 1-particle RhoCI
density.

 Excited State   2: Singlet-A 3.3562 eV  369.41 nm  f=0.1698  <S**2>=0.000
      41 -> 42          0.69832

 Excited State   3: Singlet-A 3.9385 eV  314.80 nm  f=0.0096  <S**2>=0.000
      39 -> 42          0.15004
      40 -> 42          0.68138

 Excited State   4: Singlet-A 4.8761 eV  254.27 nm  f=0.0927  <S**2>=0.000
      37 -> 42         -0.26064
      38 -> 42          0.39934
      39 -> 42          0.23868
      39 -> 43         -0.32608
      41 -> 43         -0.14670

 Excited State   5: Singlet-A  5.0753 eV  244.29 nm  f=0.1031  <S**2>=0.000
      37 -> 42          0.12063
      38 -> 42          0.42972
      39 -> 42         -0.11347
      39 -> 43          0.39021
      39 -> 48         -0.10539
      40 -> 43         -0.15619
      41 -> 43         -0.24071
```

```
Excited State   6: Singlet-A  5.2515 eV  236.09 nm  f=0.0564  <S**2>=0.000
     35 -> 42        -0.14222
     36 -> 42         0.15888
     37 -> 42         0.51031
     37 -> 43        -0.11582
     39 -> 42         0.13379
     39 -> 43        -0.25539
     41 -> 43        -0.16567

Excited State   7: Singlet-A 5.3633 eV  231.17 nm  f=0.2173  <S**2>=0.000
     38 -> 42         0.22626
     40 -> 43        -0.27452
     41 -> 43         0.51562
     41 -> 44        -0.21571

Excited State   8: Singlet-A  5.4414 eV  227.85 nm  f=0.0889  <S**2>=0.000
     38 -> 42         0.21749
     39 -> 43         0.11982
     40 -> 43         0.38649
     41 -> 43         0.31176
     41 -> 44         0.37293

Excited State   9: Singlet-A 6.2111 eV  199.62 nm  f=0.0479  <S**2>=0.000
     26 -> 42        -0.10949
     28 -> 42        -0.16325
     31 -> 42        -0.22737
     35 -> 42        -0.19566
     36 -> 42        -0.21825
     40 -> 43         0.22972
     40 -> 44        -0.10663
     41 -> 44        -0.21986
     41 -> 45         0.15615
     41 -> 46        -0.16063
     41 -> 49        -0.12035
     41 -> 52         0.12649

Excited State  10: Singlet-A 6.2913 eV  197.07 nm  f=0.0609  <S**2>=0.000
     31 -> 42         0.12304
     36 -> 42         0.51918
     37 -> 42        -0.20445
     40 -> 43         0.19773
     41 -> 44        -0.23389

Excited State  11: Singlet-A  6.3796 eV  194.35 nm  f=0.2249  <S**2>=0.000
     35 -> 42        -0.21667
     36 -> 42         0.17453
     37 -> 42        -0.15330
     40 -> 43        -0.32429
     41 -> 44         0.37222
     41 -> 46        -0.12095
     41 -> 48        -0.12015
     41 -> 52         0.10731

Excited State  12: Singlet-A 6.5293 eV  189.89 nm  f=0.2299  <S**2>=0.000
     36 -> 42        -0.10210
     38 -> 43        -0.11282
```

```
     39 -> 44         0.10275
     40 -> 44         0.54338
     41 -> 44        -0.14196
     41 -> 45        -0.16744
     41 -> 47         0.12577
     41 -> 48        -0.25590

Excited State  13: Singlet-A  6.5483 eV  189.34 nm  f=0.0101  <S**2>=0.000
     26 -> 42         0.11469
     28 -> 42         0.15239
     31 -> 42         0.18707
     35 -> 42         0.31322
     36 -> 42        -0.16252
     41 -> 45         0.32062
     41 -> 46        -0.19276
     41 -> 48        -0.14656
     41 -> 49        -0.10221
     41 -> 52         0.15639

Excited State  14: Singlet-A 6.7209 eV  184.47 nm  f=0.0535  <S**2>=0.000
     26 -> 42        -0.21838
     27 -> 42        -0.10461
     28 -> 42        -0.18153
     29 -> 42        -0.10945
     31 -> 42        -0.11878
     32 -> 42        -0.13601
     33 -> 42        -0.13997
     34 -> 42         0.11267
     35 -> 42         0.35691
     35 -> 43        -0.10183
     36 -> 42         0.15745
     38 -> 43         0.14973
     40 -> 44         0.17235

Excited State  15: Singlet-A 6.7666 eV  183.23 nm  f=0.1111  <S**2>=0.000
     35 -> 42        -0.14971
     40 -> 43         0.10013
     40 -> 44         0.21266
     41 -> 45         0.49937
     41 -> 46         0.26806
     41 -> 49         0.10279
     41 -> 53        -0.14012

Excited State  16: Singlet-A 6.9862 eV  177.47 nm  f=0.1856  <S**2>=0.000
     38 -> 43        -0.10255
     39 -> 45        -0.11200
     40 -> 44         0.24884
     40 -> 45         0.13102
     41 -> 46        -0.29642
     41 -> 47        -0.18767
     41 -> 48         0.40125
     41 -> 49        -0.12492
     41 -> 50        -0.16302

Excited State  17: Singlet-A 7.0789 eV  175.15 nm  f=0.0094  <S**2>=0.000
     36 -> 44         0.13621
     39 -> 43        -0.10245
```

```
     39 -> 44         0.58350
     39 -> 48        -0.10925
     39 -> 49         0.10644

Excited State  18: Singlet-A 7.0990 eV  174.65 nm  f=0.0778  <S**2>=0.000
     40 -> 45         0.56003
     40 -> 46         0.24819
     40 -> 47        -0.16259
     41 -> 46         0.13998
     41 -> 48        -0.11025

Excited State  19: Singlet-A 7.1473 eV  173.47 nm  f=0.0148  <S**2>=0.000
     32 -> 42        -0.22066
     33 -> 42         0.31841
     34 -> 42         0.25714
     38 -> 43         0.24391
     39 -> 45         0.24687
     39 -> 47        -0.11766
     40 -> 46         0.10732

Excited State  20: Singlet-A
     33 -> 42         0.45039
     33 -> 43        -0.10017
     36 -> 43        -0.10802
     39 -> 45        -0.29368
     39 -> 46         0.11551
     39 -> 47         0.15196
     40 -> 46        -0.10975
     41 -> 47         0.12350

Excited State  21: Singlet-A  7.1898 eV  172.44 nm  f=0.0099  <S**2>=0.000
     31 -> 42         0.13331
     32 -> 42        -0.11817
     33 -> 42        -0.22557
     34 -> 42         0.37450
     37 -> 43         0.11915
     39 -> 45        -0.27808
     39 -> 47         0.11106
     40 -> 46        -0.13456
     41 -> 46         0.11689

Excited State  22: Singlet-A 7.2668 eV  170.62 nm  f=0.0117  <S**2>=0.000
     38 -> 43         0.16770
     39 -> 44         0.12140
     39 -> 45        -0.11213
     41 -> 46        -0.35553
     41 -> 47         0.39051
     41 -> 49         0.26855

Excited State  23: Singlet-A 7.3458 eV  168.78 nm  f=0.0712  <S**2>=0.000
     34 -> 42         0.28769
     38 -> 43        -0.27057
     39 -> 44        -0.22194
     39 -> 45         0.20245
     39 -> 48        -0.15670
     39 -> 49         0.15551
     39 -> 50         0.10141
```

```
      40 -> 47        -0.10208
      40 -> 48         0.14662
      41 -> 46        -0.10448
      41 -> 47         0.10613
      41 -> 49         0.15672

 Excited State  24: Singlet-A  7.3649 eV  168.34 nm  f=0.1095  <S**2>=0.000
      34 -> 42        -0.10086
      38 -> 43         0.11944
      39 -> 46        -0.11905
      39 -> 48         0.13800
      40 -> 43        -0.10255
      40 -> 46        -0.34858
      40 -> 47        -0.24613
      40 -> 48         0.37761
      40 -> 49        -0.10475
      41 -> 44        -0.10417

 Excited State  25:      Singlet-A      7.4445 eV  166.54 nm  f=0.0101
<S**2>=0.000
      41 -> 46         0.13608
      41 -> 47         0.45331
      41 -> 48         0.23454
      41 -> 49        -0.38209
      41 -> 53         0.12220
*********************************************************************
```
